# Analysis of *Streptomyces* Volatilomes Using Global Molecular Networking Reveals the Presence of Metabolites with Diverse Biological Activities

**DOI:** 10.1128/spectrum.00552-22

**Published:** 2022-07-28

**Authors:** Jingyu Liu, Jody-Ann Clarke, Sean McCann, N. Kirk Hillier, Kapil Tahlan

**Affiliations:** a Department of Biology, Memorial University of Newfoundland, St. John’s, Newfoundland and Labrador, Canada; b Department of Biology, Acadia University, Wolfville, Nova Scotia, Canada; University of Mississippi

**Keywords:** *Streptomyces*, molecular networking, MSHub/GNPS, volatilomes

## Abstract

*Streptomyces* species produce a wide variety of specialized metabolites, some of which are used for communication or competition for resources in their natural environments. In addition, many natural products used in medicine and industry are derived from *Streptomyces*, and there has been interest in their capacity to produce volatile organic compounds (VOCs) for different industrial and agricultural applications. Recently, a machine-learning workflow called MSHub/GNPS was developed, which enables auto-deconvolution of gas chromatography-mass spectrometry (GC-MS) data, molecular networking, and library search capabilities, but it has not been applied to *Streptomyces* volatilomes. In this study, 131 *Streptomyces* isolates from the island of Newfoundland were phylogenetically typed, and 37 were selected based on their phylogeny and growth characteristics for VOC analysis using both a user-guided (conventional) and an MSHub/GNPS-based approach. More VOCs were annotated by MSHub/GNPS than by the conventional method. The number of unknown VOCs detected by the two methods was higher than those annotated, suggesting that many novel compounds remain to be identified. The molecular network generated by GNPS can be used to guide the annotation of such unknown VOCs in future studies. However, the number of overlapping VOCs annotated by the two methods is relatively small, suggesting that a combination of analysis methods might be required for robust volatilome analysis. More than half of the VOCs annotated with high confidence by the two approaches are plant-associated, many with reported bioactivities such as insect behavior modulation. Details regarding the properties and reported functions of such VOCs are described.

**IMPORTANCE** This study represents the first detailed analysis of *Streptomyces* volatilomes using MSHub/GNPS, which in combination with a routinely used conventional method led to many annotations. More VOCs could be annotated using MSHub/GNPS as compared to the conventional method, many of which have known antimicrobial, anticancer, and insect behavior-modulating activities. The identification of numerous plant-associated VOCs by both approaches in the current study suggests that their production could be a more widespread phenomenon by members of the genus, highlighting opportunities for their large-scale production using *Streptomyces.* Plant-associated VOCs with antimicrobial activities, such as 1-octen-3-ol, octanol, and phenylethyl alcohol, have potential applications as fumigants. Furthermore, many of the annotated VOCs are reported to influence insect behavior, alluding to a possible explanation for their production based on the functions of other recently described *Streptomyces* VOCs in dispersal and nutrient acquisition.

## INTRODUCTION

Volatile organic compounds (VOCs) are small odorous molecules (containing up to 20 carbon atoms) with low molecular mass (100 to 500 daltons), high vapor pressure, and low boiling point, and also containing a lipophilic moiety (1). These characteristics aid in the volatility of such molecules, making them good candidates for mediating interactions among different species in the environment by acting as diffusible signals (2, 3). Various naturally occurring VOCs spanning multiple chemical classes have been reported in bacteria, fungi, plants, animals, and humans (4–8). Due to their desirable aromas, many VOCs are also manufactured synthetically for use in the food, cosmetic, chemical, and pharmaceutical industries. However, there has been interest in using microbes as sources for the engineered production of such relevant compounds (9).

Bacteria from the genus *Streptomyces* are a promising source of VOCs due to their known ability to produce beneficial natural products (or specialized metabolites), many of which have applications in industry, medicine, and agriculture. *Streptomyces* species display complex cellular characteristics, which include mycelial growth and the ability to produce spores, they are ubiquitous soil dwellers occupying various habitats such as the rhizosphere, and some are also symbionts of plants and animals (10). Numerous specialized metabolites of *Streptomyces* origin have led to the development of currently used antifungal, anticancer, immunosuppressant, antibacterial, and anthelmintic agents (11), more common than those of complete synthetic origin (12). Genome mining techniques have revealed that almost every *Streptomyces* species contains an impressive number of biosynthetic gene clusters (BGCs) associated with specialized metabolite production (13, 14), which include VOCs such as geosmin and 2-methylisoborneol (15, 16). Many studies to date have focused on *Streptomyces* species for antibiotic discovery, but VOCs produced by them are predicted to be just as bountiful, with potential applications in agriculture and industry (17–19). For example, microbially produced VOCs with antifungal activities have been applied in agriculture as fumigants to control plant disease (20); they have also been used in the animal feed industry to control molds (21).

In nature, many VOCs produced by *Streptomyces* have roles in cellular proliferation, defense, inter- and intraspecies communication, and competition for resources. For example, Streptomyces venezuelae produces the VOC trimethylamine (TMA), which has been shown to promote a specialized mode of growth in other members of the genus and to reduce iron availability for competitors (22). The VOCs geosmin and 2-methylisoborneol, which are responsible for the characteristic earthy odor of soil are also produced by *Streptomyces* and are thought to be involved in attracting invertebrates such as arthropods so that *Streptomyces* spores can adhere to them for dispersal (23). It has also been reported that fruit flies are attracted by low levels of 2-methylisoborneol produced by certain *Streptomyces* species, which then use another specialized metabolite to kill and utilize the flies, possibly for nutritional purposes (24). Concomitantly, some insects avoid geosmin and higher levels of 2-methylisoborneol, which possibly signal danger due to the presence of *Streptomyces* in the vicinity (25, 26). VOCs possessing antimicrobial activities have the added advantage of inhibiting competitors at a distance. For example, the production of butanone, 1,3,5-trichloro-2-methoxy benzene, and dimethyl disulfide by some *Streptomyces* species was demonstrated to inhibit the germination or growth of certain saprophytic and pathogenic fungi (17, 27, 28). In addition, VOCs produced by free-living and symbiotic *Streptomyces* might play still unrecognized roles in modulating the behaviors of insects and animals. For example, dodecanol/dodecanal, known to be produced by *Streptomyces* (29, 30), is also found in the pheromone gland of the codling moth (Cydia pomonella) and male ring-tailed lemurs (31, 32).

Due to the importance of VOCs produced by bacteria and other organisms, there has been considerable interest in their study recently. VOCs from different sources are often detected or identified using electron ionization gas chromatography-mass spectrometry (GC-MS), which employs vendor-specific software or publicly available resources for data analysis (33–37). This requires a certain level of user expertise for defining parameters during data analysis, which sometimes leads to variations in the annotation and quantification of reported VOCs between studies (38, 39). Recently, a machine-learning workflow called MSHub/GNPS was developed, which not only enables auto-deconvolution of GC-MS data, but also allows for molecular networking and library searching (38). However, there are no additional reports on the application of MSHub/GNPS on *Streptomyces* volatilome profiling or its comparison to other so-called conventional or traditional methods. Therefore, we applied the MSHub/GNPS pipeline to analyze the volatilomes (the full suite of VOCs released) of environmental *Streptomyces* isolates belonging to 30 different operational taxonomic units (OTUs), which were cultured under different conditions. For comparison, GC-MS-based VOC analysis was also performed using a method relying on user-guided annotation that we have used routinely in the past (18), and the results of the two analyses are presented to highlight important findings.

## RESULTS AND DISCUSSION

### Collection and molecular typing of *Streptomyces* isolates.

**FIG 1:**
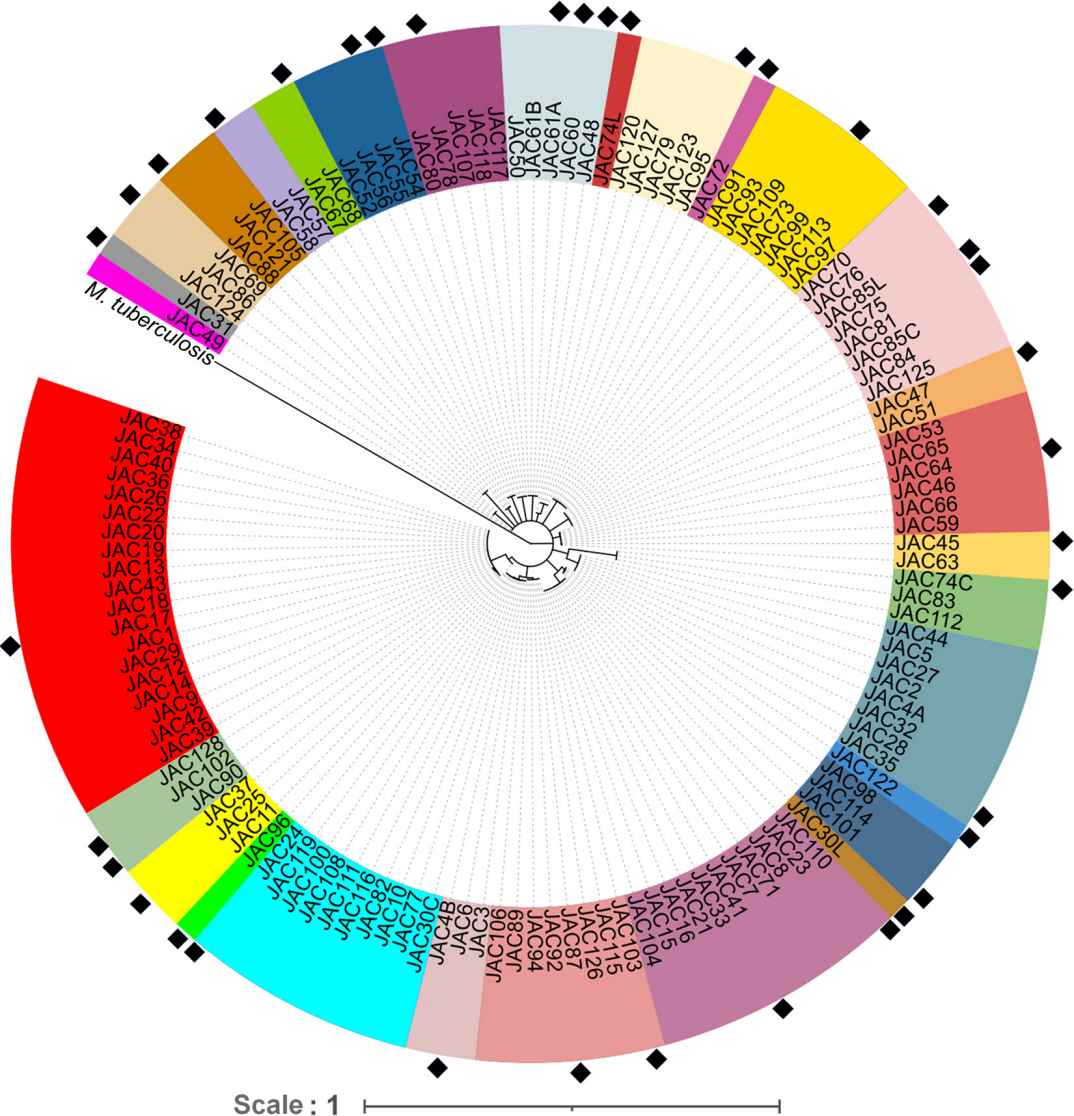
Phylogenetic relationship between the different *Streptomyces* isolates from the current study. The tree consists of 131 *rpoB* gene sequences from the respective isolates, which clustered into 30 OTUs. Each OTU is colored differently to highlight diversity. The tree was constructed using 880-bp sequences with 100 bootstrap replicates, and a minimum 50% consensus cutoff value was used to resolve the branches. The equivalent sequence of M. tuberculosis *rpoB* served as an outgroup, and the isolates selected for VOC analysis are indicated (♦). The scale bar indicates the number of nucleotide substitutions per site.

All 131 *Streptomyces* isolates used in the current study were obtained from soil samples collected from the island of Newfoundland, Canada, using selective isolation procedures described for members of the genus (40). They were identified and typed by sequencing a specific region of the *rpoB* gene, which is known to provide good discrimination for phylogenetic analysis (see Table S1 in the supplemental material) (41, 42). The different isolates clustered into 30 OTUs (Fig. 1 and Fig. S1), with 23 dominant OTUs containing members related to S. pratensis (19 isolates); S. mirabilis (11 isolates), S. sanglieri (10 isolates), S. hygroscopicus (8 isolates), S. sampsonii (8 isolates), S. aureus (7 isolates), S. antibioticus (6 isolates), S. praecox (5 isolates), S. murinus (5 isolates), S. ficellus (5 isolates), S. nojiriensis (4 isolates), S. albogriseolus (4 isolates), S. xiamenensis (3 isolates), S. fagopyri (3 isolates), S. cinereoruber (3 isolates), and S. scopuliridis (3 isolates) (Fig. 1 and Fig. S1). The remaining seven OTUs only contained one isolate each, which were found to be closely related to S. niveus, S. atratus, S. alboniger, *Streptomyces* sp. strain10ZA5, *Streptomyces* sp. strain 09VY61, *Streptomyces* sp. strain 10NE7, and *Streptomyces* sp. strain RPA4-2, respectively (Fig. 1 and Fig. S1).

Of the 131 *Streptomyces* isolates, 37 were chosen for VOC analysis (Fig. 1 and Fig. S1), which was based on their position in the phylogenetic tree and the ability of each isolate to grow on the three fermentation media used in the current study. In certain cases, up to three isolates were selected from dominant OTUs (Fig. 1), as bacteria with similar *rpoB* gene sequences can sometimes display differences in specialized metabolite production profiles (43). In addition, all isolates from OTUs containing single members were also included irrespective of their growth characteristics, which should allow for the examination of VOCs that are produced by phylogenetically distinct members of the genus (Fig. 1 and Fig. S1).

### Screening of *Streptomyces* isolates for VOC production.

It is well documented that nutritional and growth conditions influence microbial specialized metabolism (44–46), which has led to the concept of one strain many compounds (OSMAC). Therefore, the 37 selected *Streptomyces* isolates were grown in three *Streptomyces* fermentation media: SFM, which supports diverse VOC and specialized metabolite production (15, 30, 47–49), a synthetic medium previously used to promote geosmin production (16, 50), and YMS, a general specialized metabolite production medium (51). Cultures of each isolate grown in the three media were pooled before VOC trapping/collection, to reduce the number of samples that had to be processed for GC-MS analysis during the screening phase (18).

**FIG 2:**
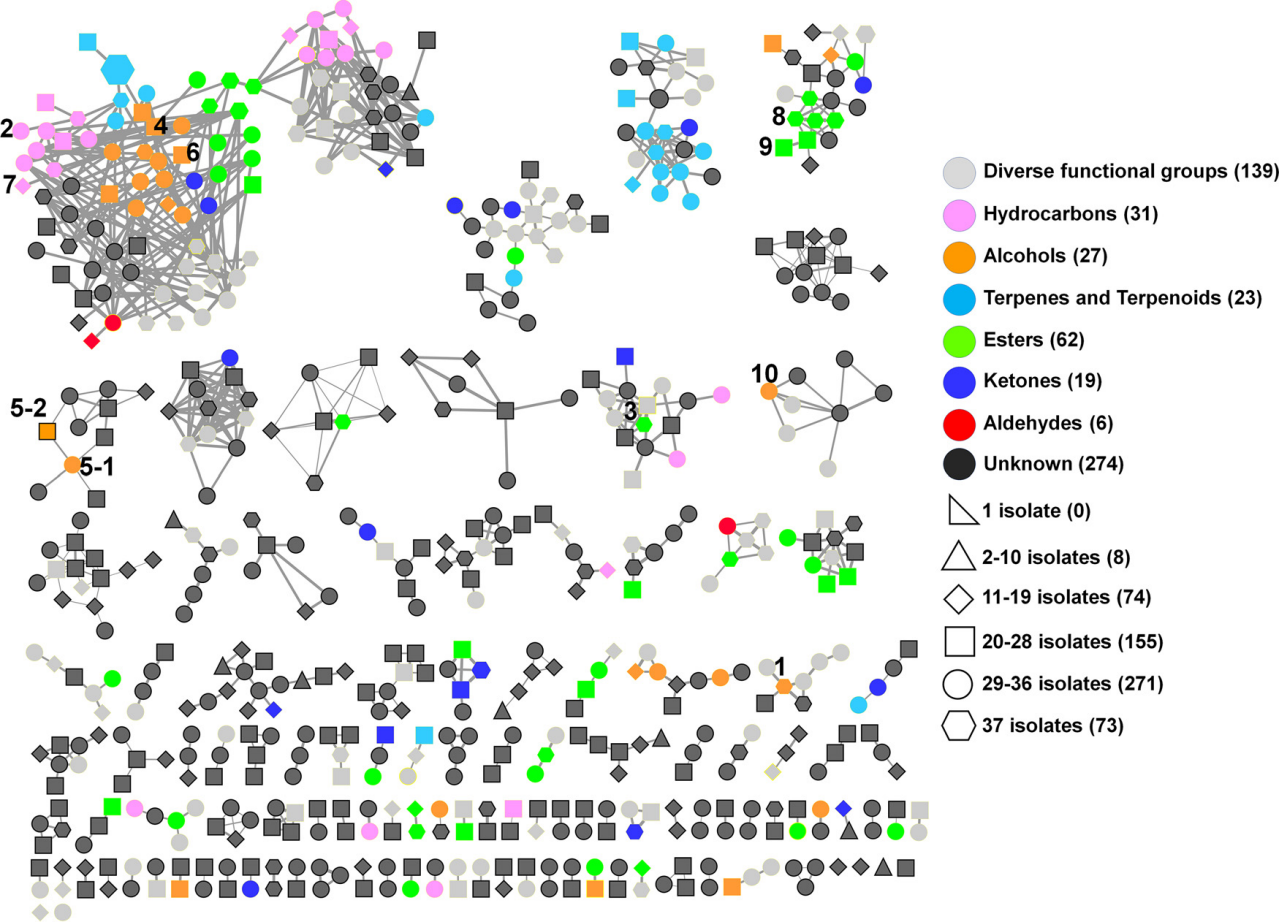
Molecular network generated by MSHub/GNPS using spectra of VOCs collected from cultures of the 37 *Streptomyces* isolates. Each node represents one fragmentation spectrum corresponding to a VOC, and node size represents the summed intensity (peak area) of the respective VOCs from all 37 isolates. The colors of the nodes are based on chemical class, and their shapes indicate the distribution of the VOC among the 37 *Streptomyces* isolates. The total number of VOCs from each chemical class (node colors) and their distribution (node shapes) are indicated in the legend. VOCs annotated by both MSHub/GNPS and the conventional method are labeled with numbers, which correspond to their identities in Fig. 4.

**FIG 3:**
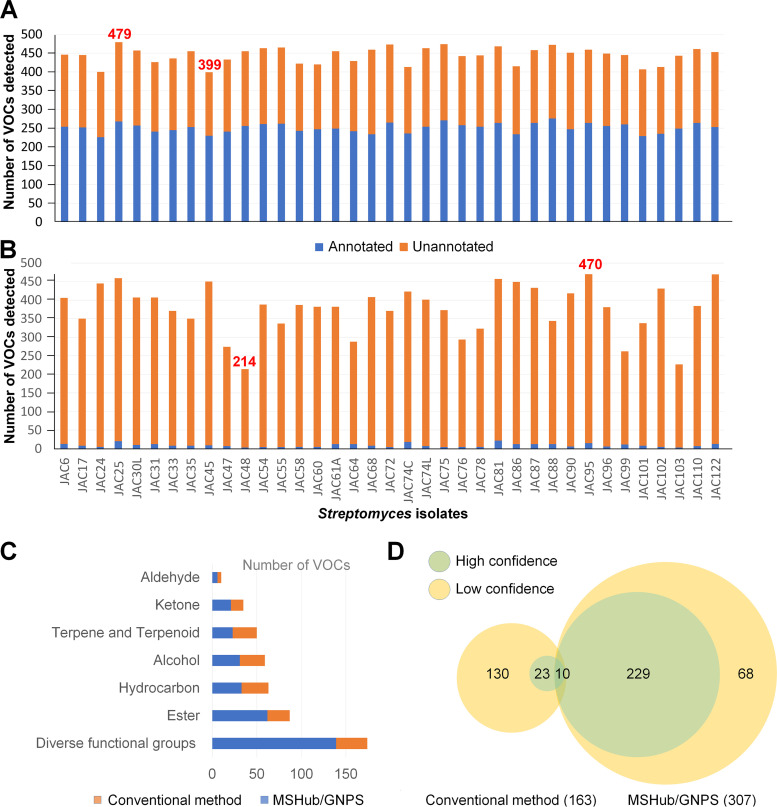
(A and B) Numbers of VOCs detected and annotated from pooled *Streptomyces* isolate cultures using (A) MSHub/GNPS molecular networking and (B) the conventional method. (C) Groups of VOCs from different chemical classes detected by the two methods. (D) Numbers of VOCs annotated with high (green) and low (yellow) confidence using the conventional method and MSHub/GNPS.

The GC-MS data obtained were then analyzed using MSHub/GNPS (38), which led to 1,120 spectra after deconvolution. Molecular networks were built in GNPS using spectra with a minimum of six shared peaks and cosine similarity scores of ≥0.60. The molecular network from the 37 *Streptomyces* isolates contained 581 nodes (each node represents one spectrum or VOC) after manual removal of nodes also present in control samples (empty collection jars and pooled uninoculated media). Of the annotated VOCs, 73 were produced by all 37 isolates (the core volatilome; Table S2; discussed in more detail in the following section), 271 by 29 to 36 isolates, 155 by 20 to 28 isolates, 74 by 11 to 19 isolates and 8 by 2 to 10 isolates, but no VOC could be attributed to a single isolate only (Table S3 and Fig. 2). Results showed that JAC25 and JAC45 isolates contained the highest (*n* = 479) and lowest (*n* = 399) number of VOCs, respectively (Fig. 3A).

Of the 581 VOCs detected using MSHub/GNPS, 307 were annotated by GNPS library matching (default balance score, 50%) (Tables S2 and S3), and could be grouped into seven major chemical classes (Fig. 3C). This included one category labeled as compounds with diverse functional groups (DFG) comprising VOCs with multiple functional groups or not classified under the other six classes. DFG and the esters were the most common chemical classes represented among the 307 annotated VOCs (Fig. 3C and Table S3), which were further grouped based on confidence in their identities. Compounds with balance scores of ≥65% (*n* = 239) and <65% (*n* = 68) were considered high and low confidence matches, respectively (Fig. 3D and Tables S3 and S4). Of the 239 VOCs annotated with high confidence, more than half of them (*n* = 156) are plant-associated and/or have not been reported from *Streptomyces* previously (*n* = 132) (Table S4). The production of some plant-associated metabolites and VOCs in *Streptomyces* has been observed previously (18, 52), highlighting the potential for using bacteria to produce such compounds. In addition, VOCs annotated with high confidence using MSHub/GNPS are reported to display a variety of activities, including antibacterial [1-tridecanol, 3-pentadecanol, and bis(2-ethylhexyl) phthalate] (53–62), antifungal [cis-9-hexadecenal, (Z)-12-octadecenoic acid methyl ester; hexanedioic acid; bis(2-ethylhexyl) ester and octadecyl 3-(3,5-di-tert-butyl-4-hydroxyphenyl) propionate] (63–69), and anticancer [6,10-dimethyl-4-undecanol and 1,2-benzenedicarboxylic acid bis(2-ethylhexyl) ester] (70, 71). Bis(2-ethylhexyl) phthalate is often considered a background artifact during the analysis of biologically produced volatiles but has been reported to be produced by many organisms, and there have been some discussions regarding it being classified as a natural product (62). In this study, we found bis(2-ethylhexyl) phthalate in samples from all 37 isolates, with JAC90 extracts containing the largest amount (Fig. S2). Some of the VOCs annotated using MSHub/GNPS have also been shown to influence insect behavior by acting as pheromones [(6Z,9Z)-6,9-tricosadiene; cis-9-hexadecenal and (Z)-11-hexadecenal] (68, 69, 72–74).

In addition to MSHub/GNPS, the same GC-MS data were also analyzed using a manually guided (conventional) method. The Bruker MS Workstation (version 8.0.1; Bruker Daltonics, UK) was used for peak detection, and the National Institute of Standard and Technology (NIST) Mass Spectral Search Program (NMSS) for the NIST/EPA/NIH Mass Spectral Library (version 2.0g, built in 2011, Scion Instruments, UK) was used for VOC annotation (18), which showed that the JAC95 and JAC48 isolates contained the highest (*n* = 470) and lowest numbers (*n* = 214) of VOCs, respectively (Fig. 3B). The conventional method used the NIST library for matching (75), leading to the annotation of 163 VOCs, with the hydrocarbons and those grouped as DFG being the most common (Fig. 3C). In addition, 33 VOCs were annotated with high confidence using the conventional method, with half (*n* = 16) of them being plant associated and 18 that had not been reported from *Streptomyces* previously (Fig. 3D and Tables S5 and S6). A range of interesting bioactivities have also been reported for the VOCs annotated with high confidence using this method, for example, 2-methylisoborneol has antifungal activity (45–47); benzyl alcohol, 1*H*-benzocycloheptene; 4a*S*-cis-2,4a,5,6,7,8,9,9a-octahydro-3,5,5-trimethyl-9-methylene, cubenol, and geosmin have insect repellent activities (48–53); geosmin and 2-methylisoborneol also have insect attractant activities (17, 54, 55); and cyclohexanemethanol, 4-ethenyl-α,α,4-trimethyl-3-(1-methylethenyl)-, [1R-(1α,3α,4β)] and hexanenitrile influence other insect signaling or communication systems (56, 57).

### VOCs annotated by both MSHub/GNPS and the conventional method.

**FIG 4:**
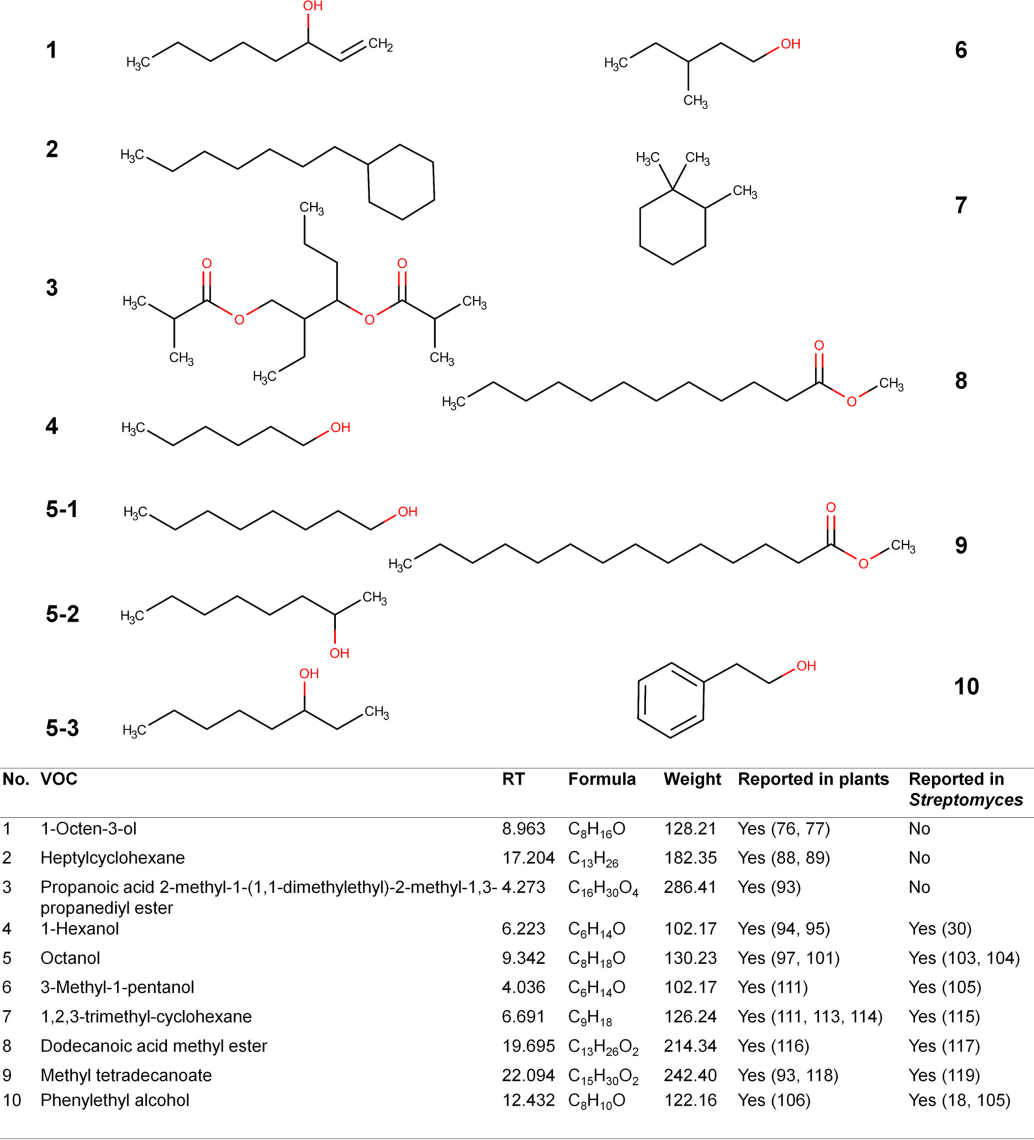
VOCs detected and annotated by both MSHub/GNPS and the conventional method during pooled culture analysis of 37 *Streptomyces* isolates.

Ten VOCs were annotated by both methods, and they are all plant associated (Fig. 2 and 4 and Fig. S2). The production of two of these metabolites (1-hexanol and phenylethyl alcohol) was recently noted in certain industrially important *Streptomyces* species (18), whereas 1-octen-3-ol, heptylcyclohexane, and propanoic acid 2-methyl-1-(1,1-dimethylethyl)-2-methyl-1,3-propanediyl ester have not been reported from members of the genus before. 1-Octen-3-ol, also known as mushroom alcohol, has been reported in plants (orange essential oil and the herb Aster scaber) (76, 77), fungi (Metarhizium brunneum) (78–80), bacteria (rhizospheric isolate L255) (81), and arthropods (Ahasverus advena and Amblyomma variegatum) (82, 83), but not in *Streptomyces*. It has been shown to have antibacterial, antifungal (84), nematode repellent, nematicidal (78–80), and insect attractant activities (85, 86); it also inhibits the germination and growth of some pathogenic fungi (84, 87). In addition, low-level production of 1-octen-3-ol by an entomopathogenic fungus (Metarhizium brunneum) repels slugs, snails, and nematodes but causes their killing at higher doses (78–80). In combination with carbon dioxide, 1-octen-3-ol can also function as an attractant for horse flies (Tabanus sulcifrons), and mosquitoes (85, 86). Heptylcyclohexane has been reported in medicinal plants (Gnidia glauca and Dioscorea bulbifera) and several strawberry varieties (88, 89). It is also produced by insects such as shield or stink bugs (*Pentatomidae*), where it functions as a pheromone, but it has not been reported in *Streptomyces* prior to the current study (90–92). Propanoic acid 2-methyl-1-(1,1-dimethylethyl)-2-methyl-1,3-propanediyl ester is a plant-associated metabolite (from Leonotis nepetifolia) that has also not been reported in *Streptomyces* previously (93), but not much is known about its biological activity.

Both data analysis methods were also able to annotate plant-associated VOCs that have been reported in *Streptomyces* before (some of which are discussed in the previous section, and others are described below). 1-Hexanol has been reported in plants (94, 95), fungi (96, 97), and bacteria (30, 98), and it has various bioactivities, including plant growth modulation (98), suppression of mosquito CO_2_ sensing receptors (99), and attraction of the banded elm bark beetle (Scolytus schevyrewi), a pest of apricot trees (100). Octanol has been reported to be produced by plants (97, 101), fungi (96, 102), and bacteria, including *Streptomyces* (103, 104), and it displays diverse activities depending on the specific isomer. 1-Octanol and 3-octanol have been detected in wounded or infected plants (97), where 1-octanol also promotes cell division and indole-3-acetic acid production in the phylloplane Pseudomonas sp. strain NEEL19 (101). In addition, 2-octanol produced by soil bacteria (including *Bacillus*, *Microbacterium*, *Stenotrophomonas*, *Streptomyces*, and *Serratia*) displays potent nematocidal activities against a free-living nematode (Panagrellus redivivus) and the pinewood nematode (Bursaphelenchus xylophilus) (104). Another compound detected was phenylethyl alcohol, which is synthesized by multiple *Streptomyces* species (19, 105) and plants (106) and is known to repel certain insects (107, 108). It also has antifungal activities against pathogens of peanuts (109) and sweet potato (110). 3-Methyl-1-pentanol is a plant-associated metabolite (from Triadica sebifera) (111) that has also been found in Streptomyces citreus (105) and is an attractant for owlet moths (Noctuinae) (112). 1,2,3-Trimethyl-cyclohexane has been described in certain flowering plants and fruit trees (111, 113, 114) and was also reported as one of the major components of *Streptomyces* extracts possessing anti-methicillin-resistant Staphylococcus aureus (MRSA) activities (115). Dodecanoic acid methyl ester is also widely found in plant oils (116), and its production in *Streptomyces* has been reported (117), but currently there is no information on its function or biological activity. Methyl tetradecanoate has been reported in multiple plant species (93, 118), *Streptomyces* (119), and insects (120–122). It has repellent activity toward ants (122) and is involved in different insect signaling and communication systems in nature, including aggregation (or attraction) when produced in fruit flies (*Drosophila*) (90). Interestingly, certain pathogens lead to increased production of methyl tetradecanoate at the site of infection in fruit flies, which serves to lure more flies for spreading the infection to other members (123).

For the bioactive plant-associated VOCs annotated in the current study, it might be possible to use *Streptomyces* for producing them for large-scale applications. For example, VOCs with antimicrobial or nematocidal activities (such as 1-octen-3-ol, octanol, phenylethyl alcohol, and 1,2,3-trimethyl-cyclohexane) could potentially function as fumigants for use in agriculture. VOCs with insect repellent, attractant, or behavior-modulating activities (such as 1-octen-3-ol, heptylcyclohexane, 1-hexanol, phenylethyl alcohol, 3-methyl-1-pentanol, and methyl tetradecanoate) might have application in managing pests in agricultural and other settings.

### Analysis of VOC production in replicate cultures of select *Streptomyces* isolates.

**FIG 5:**
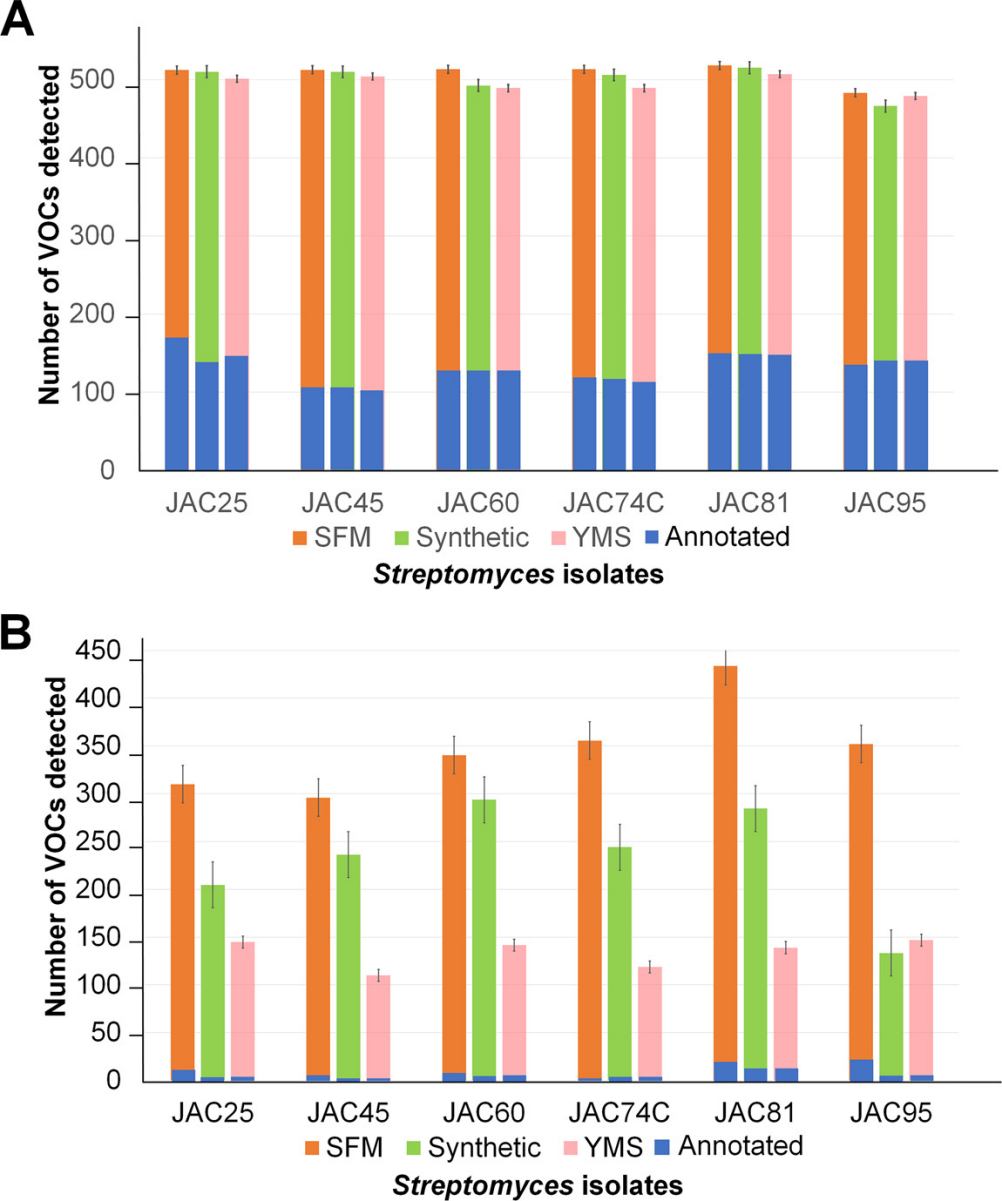
(A and B) The number of VOCs detected and annotated in replicate cultures of six selected *Streptomyces* isolates using (A) MSHub/GNPS molecular networking and (B) the conventional method. VOCs were counted if they were present in at least two replicate cultures of a given isolate grown in each respective medium. The mean and standard deviation in numbers of VOCs produced in three replicate cultures of each isolate (black lines) and proportion of VOCs annotated in each case (blue bars) are also indicated.

To confirm the results of pooled culture screens (Fig. 2 and 3), 6 of the 37 *Streptomyces* isolates were selected and subjected to repeated culturing in each medium, followed by VOC analysis. Selection of the specific isolates (JAC25, 45, 60, 74C, 81, and 95) was based on the numbers, types, and reported insect behavior-modulating activities of the VOCs produced by them during the screening stage described above. The six *Streptomyces* isolates were cultured in triplicate in the three fermentation media, and each culture was individually used for collecting VOCs for GC-MS analysis. VOCs detected in at least two of three replicate cultures were used to confirm the production of a metabolite by each isolate grown on a specific fermentation medium and were compared to results from the pooled analysis (Fig. 5; Tables S3, S5, S7, and S8). Analysis of the data obtained using MSHub/GNPS and the conventional method showed that highest (518 and 434) and lowest (466 and 110) numbers of VOCs were produced in SFM and YMS media, respectively (Fig. 5, Tables S7 and S8). In addition, some VOCs were only produced by certain isolates when cultured in a specific fermentation medium (Tables S7 and S8), for example, more than one third (*n* = 36) of the annotated VOCs were only produced in SFM (Table S8), which was expected due to the known effects of nutritional and growth conditions on *Streptomyces* specialized metabolism (44–46).

Analysis of GC-MS data using MSHub/GNPS showed that the average numbers of VOCs detected were 509 (±13), 500 (±18), and 495 (±11) (mean standard deviations indicated in parenthesis) for SFM, synthetic, and YMS media, respectively (Fig. 5A), and a total of 242 VOCs were annotated using this strategy (Table S7). Of these 242 VOCs, 75 were also annotated during the pooled culture screens by MSHub/GNPS (Tables S3 and S7), further increasing the confidence in their annotation. In addition, the production of VOCs identified during the preliminary analysis with reported bioactivities, such as hexanol, phenylethyl alcohol, 2-methylisoborneol, 1-octen-3-ol, 1-octanol, 2-octanol, 1,1,4-trimethylcyclohexane, heptylcyclohexane, methyl tetradecanoate, dodecane, and undecane was also confirmed (Table S7).

In comparison to MSHub/GNPS, the average numbers of VOCs detected per isolate using the conventional method of analysis were 348 (±48), 233 (±59), and 133 (±15) (mean standard deviations indicated in parenthesis) for SFM, synthetic, and YMS medium, respectively (Fig. 5B), with a total of 95 annotations (Table S8). Compared to pooled culture screens (Table S5), the production of 28 VOCs was confirmed during replicate culture analysis using the conventional method (Table S8). In addition, VOCs with bioactivities detected during initial screening, such as geosmin, hexanol, phenylethyl alcohol, and 2-methylisoborneol were again detected and annotated during the analysis of replicate cultures using the methodology (Table S8).

Overall, results from replicate culture analysis corroborated those obtained earlier using pooled cultures and identified the specific growth media in which each of the annotated VOCs were produced by the respective isolates (Fig. 5, Tables S3 to S8). However, the overlap between the high-confidence matches annotated by MSHub/GNPS and the conventional method is less than 10, and MSHub/GNPS seems to remove the volatilome variability between media and isolates (Table S2 to S8, Fig. 3 and 5), suggesting that it performs better in such situations. Another point to note is that 68 to 69% of VOCs annotated by the two methods during replicate culture analyses were not detected during the initial screens. It is possible that some of these VOCs are produced at low levels in specific media and are diluted out during the pooled culture screen due to nonproducing cultures, which would make it challenging to detect them. Therefore, a combination of pooled and replicate culture analysis maybe necessary to characterize *Streptomyces* volatilomes effectively.

### Conclusions and perspectives.

In the current study, we applied the recently developed MSHub/GNPS molecular networking approach along with a conventional method routinely used by our group to analyze the volatilomes of select environmental *Streptomyces* isolates. More VOCs could be detected using MSHub/GNPS than using the conventional method (Fig. 3 and 5; Tables S3, S5, S7, and S8), which might be due to the ability of MSHub/GNPS to use machine learning for conducting user-independent, auto-estimated deconvolution. Furthermore, MSHub/GNPS seems to remove the volatilome variability between media and isolates (Tables S2 to S8, Fig. 3 and 5), is freely available, and provides access to large publicly available data sets for comparison. The number of overlapping VOCs annotated by both methods is relatively small (Fig. 4), but the conventional approach annotated some compounds that MSHub/GNPS missed, which included geosmin, cubenol, benzyl alcohol, 1*H*-benzocycloheptene, and 4a*S*-cis-2,4a,5,6,7,8,9,9a-octahydro-3,5,5-trimethyl-9-methylene, all of which have insect repellent activities (51–54, 63, 64). In comparison, MSHub/GNPS was able to annotate some VOCs with antibacterial [1-tridecanol, 3-pentadecanol, and bis(2-ethylhexyl) phthalate] (53, 54), antifungal [cis-9-hexadecenal, (Z)-12-octadecenoic acid methyl ester, hexanedioic acid, bis(2-ethylhexyl) ester, and octadecyl 3-(3,5-di-tert-butyl-4-hydroxyphenyl)propionate] (63–69), and anticancer [6,10-dimethyl-4-undecanol and 1,2-benzenedicarboxylic acid bis(2-ethylhexyl) ester] (70, 71) activities, which were missed by the conventional method. Results suggest that MSHub/GNPS and the conventional method are complementary due to obvious differences in design and workflow, and that the use of both methods is required for thorough volatilome characterization. In addition, the total number of VOCs detected by the two approaches was higher than those which could be assigned annotations; so many potentially novel VOCs with unknown bioactivities remain to be identified. With machine learning approaches becoming more prevalent and with an increase in publicly available libraries, the repertoire of known VOCs for future applications is expected to increase greatly.

Plant-associated VOCs and metabolites are widely used in different products and applications. In this study, more than half of the VOCs annotated by both MSHub/GNPS and the conventional method are plant associated (Tables S4 and S6). In some of our previous work, we described the ability of certain industrially important *Streptomyces* species to produce secreted specialized metabolites (52, 124) and VOCs normally associated with plants (18). The ability of two distinct analysis methods to detect such VOCs from phylogenetically distinct *Streptomyces* species isolated from the environment in the current study suggests that the phenomenon might be more widespread. However, since most information on such metabolites comes from previous work on plants, it is possible that plant-associated VOCs might also be produced by other organisms, a question that will be answered over time as future studies analyze other diverse sources using accessible technologies. Many VOCs are synthetically manufactured for use in industry and agriculture, and the ability of diverse *Streptomyces* strains to make such compounds might provide an alternate route for their large-scale production.

The exact reason for the production of plant-associated metabolites by *Streptomyces* is currently not clear, but since these bacteria inhabit the rhizosphere (125), it is possible that such metabolites (including VOCs) have roles in eliciting interactions with plants and other organisms in the environment (2). It was recently reported that certain *Streptomyces* strains produce VOCs to attract arthropods for the purposes of spore dispersal or to cause their killing for nutrient acquisition (23, 24). In addition, some insects sense specific bacterial VOCs and are repelled by them as a possible mechanism of avoidance (25, 26). Many of the plant-associated VOCs detected in the current study have been reported to modulate arthropod behavior (126), so it is possible that their production by *Streptomyces* is a form of mimicry to influence such interactions. These possibilities need to be examined in more detail and in their appropriate natural context, which are part of long-term studies currently being conducted by our group.

## MATERIALS AND METHODS

### Media, reagents, and culture conditions.

The *Streptomyces* isolates used in this study were isolated from soil using previously described procedures (127) and were cultured at 28°C on International *Streptomyces* Project (ISP) medium 4 plates (ISP-4; BD Biosciences, Canada) or in Trypticase soy broth (TSB; BD Biosciences, Canada). All liquid cultures were grown in flasks containing stainless steel springs and were agitated by shaking at 200 rpm. Cultures for DNA isolation were grown for 3 to 5 days in nutrient broth (BD Biosciences, Canada). For VOC collection, 1% (vol/vol) of 2-day-old seed cultures grown in TSB were used to inoculate 25 mL of SFM (15, 30, 47–49), synthetic (16, 50), or YMS (51) medium in 250-mL flasks, which were then incubated for 5 days.

### Molecular procedures, typing of isolates, VOC collection, and GC-MS analysis.

All procedures using kits were carried out as per instructions provided by the manufacturer. Genomic DNA was isolated using the Presto mini genomic DNA (gDNA) bacterial kit (Geneaid Biotech Ltd., Taiwan), and an 880-bp region of *rpoB* was PCR amplified using the Phusion high-fidelity PCR kit (New England Biolabs [NEB], Canada). PCR products were purified using the EZ-10 spin column PCR product purification kit (Bio Basic, Inc., Canada) and were sequenced at The Centre for Applied Genomics (TCAG) (University of Toronto, Canada). All primers used in the current study are listed in Table S1.

Phylogenetic trees were prepared using *rpoB* gene sequences of all *Streptomyces* isolates from the current study and those extracted from *Streptomyces* genome assemblies available in the NCBI database (*n* = 282, as of 8 April 2021). The online Benchling software (https://www.benchling.com/) MAFFT function was used to prepare multiple-sequence alignments, which were downloaded in FASTA format for analyses in MEGA X (128). Evolutionary history was inferred using the maximum likelihood method and general time reversible model (129) using trimmed sequences and 100 bootstrap replicates. The phylogenetic tree was visualized and edited using the Interactive Tree of Life (iTOL) version 5 (130).

For pooled culture VOC analysis, the 37 selected *Streptomyces* isolates were grown in the three fermentation media described above. Cultures of each isolate grown in the three media were mixed before VOC collection following the method described by Cheng et al. (18). The samples were then analyzed using a Scion 456 gas chromatograph-single quad mass spectrometer (GC-MS; SCION Instruments, UK) with an electron ionization (EI) energy of 70 eV and scanning *m/z* of 40 to 350. Separations were carried out using a nonpolar capillary column Rxi-5silms (30 m by 0.25 mm; film thickness, 0.25 mm; Restek Corporation, USA) linked to a Bruker mass spectrometer (Bruker Daltonics, UK) following the program described by Cheng et al. (18). The exact same procedure was also used during replicate culture analysis, except that individual 20- mL cultures of each isolate grown in a single medium were used for VOC collection. An empty jar and uninoculated media subjected to the same conditions of volatile trapping, sampling, and analysis served as controls.

### User-guided identification of VOCs.

For the conventional method, peaks from GC-MS analysis were detected using the Bruker MS Workstation (version 8.0.1; Bruker Daltonics Ltd., UK) using a detection threshold of 2,000 counts per second, a maximum peak width of 6.0 s, a slope sensitivity of 20, and a tangent of 10%. VOCs were annotated using the NIST Mass Spectral Search Program (NMSS) for the NIST/EPA/NIH Mass Spectral Library (version 2.0g, built in 2011; Scion Instruments, UK), as described previously (18). To summarize, spectrum lists of VOCs were created for each chromatogram in the Bruker MS Workstation, and VOC matches suggested by NMSS were then evaluated based on spectral similarity and comparison of their Kovats retention indices (RI) to published values. Spectra obtained by analyzing control samples from empty jars and uninoculated media were also annotated using the NIST Mass Spectral Search Program (NMSS) to generate a list of VOCs that were present in them. The VOCs annotated in the control samples were manually removed from the list of test samples. For replicate culture VOC analysis using both methods (MSHub/GNPS and conventional), VOCs were counted if they were present in at least two replicate cultures of a given isolate. The VOCs that were only present in one replicate were removed. Compounds from bacterial cultures also detected in control samples were considered medium-derived or artifacts of the collection and sampling procedure and thus not of bacterial origin. Compounds with good spectral matches to NMSS references and authentic standards or supported by published RI data (absolute difference, ≤10) (75) were considered high-confidence matches. The annotated VOCs were then classified using ClassyFire (131). If no authentic standards were available and the published RI could not support an NMSS-suggested compound ID or there was no NMSS-suggested compound ID, the VOC was deemed a low-confidence match or an unknown, respectively.

### Annotation of VOCs using MSHub/GNPS.

GC-MS data were converted from .xml (vendor-specific format) to .CDF or .mzML using OpenChrom Lablicate Edition (version 1.4.0) (132) and were uploaded to MassIVE (https://massive.ucsd.edu). The data were then processed using MSHub deconvolution workflow (38) to generate a spectrum file (.mgf) and a quantification table (.csv), which were directly used as the input for molecular-library search-GC workflow. A spectrum similarity cosine score of ≥0.60, and at least six matched peaks were used to generate molecular networks using GNPS (version 30). VOCs were annotated by library search using a balance score of 65% to guarantee that only spectra with high quality were searched against the library (considered high-confidence matches, ≥65%), and were listed separately (Tables S4 and S6) (38). A default balance score of 50% was also used for the library search, and these annotations (≥50%, ≤65%) were deemed low-confidence matches. The web links for the MSHub/GNPS jobs generated in this study can be found in Table S9. The resulting network file (.graphml) was visualized in Cytoscape (version 3.7.2) (133), and the nodes corresponding to the control (≥50%) were removed manually. The library search results were retrieved from GNPS, and compounds with annotations were classified using ClassyFire (131).

### Data availability.

All MS data are publicly available in MassIVE (https://massive.ucsd.edu) under the accession numbers MSV000087409 and MSV000088131. Web links for MSHub/GNPS jobs generated in this study are included in Table S9.
