# Supplementary material for: Analysis of Streptomyces Volatilomes Using Global Molecular Networking Reveals the Presence of Metabolites with Diverse Biological Activities
Source: Microbiol Spectr. 2022 Jul 28;10(4):e00552-22. doi: 10.1128/spectrum.00552-22 (PMC9431705; doi:10.1128/spectrum.00552-22)
Supplement: Supplemental file 1 — Fig. S1-S3 and Tables S1-S9. Download spectrum.00552-22-s0001.pdf, PDF file, 4.7 MB [file spectrum.00552-22-s0001.pdf]

Analysis of *Streptomyces* volatilomes using global molecular networking reveals the presence of metabolites with diverse biological activities

Jingyu Liu[1Δ], Jody-Ann Clarke[1Δ], Sean McCann[2], N. Kirk Hillier[2] and Kapil Tahlan[1#]

[1] Department of Biology, Memorial University of Newfoundland, St. John's, NL A1C 5S7 Canada

[2] Department of Biology, Acadia University, Wolfville, NS B4P 2R6, Canada

[Δ] These authors contributed equally to the work.

# Correspondence: ktahlan@mun.ca

SUPPORTING INFORMATION LIST

Figure S1. Phylogenetic relationship between *Streptomyces* isolates from Newfoundland and other *Streptomyces* whose complete genome sequences are available from NCBI for download (n=211). 4

Figure S2. Molecular network generated using data from cultures of 37 *Streptomyces* isolates (cosine 0.6). 6

Figure S3. Molecular network generated using replicate cultures of the six selected *Streptomyces* isolates (cosine 0.6). 8

Table S1. Oligonucleotide primers used for PCR amplification and DNA sequencing in the current study. 9

Table S2. Core VOCs annotated in all the 37 *Streptomyces* isolates using the MSHub/GNPS. 10

Table S3. Bacterially derived compounds annotated in pooled culture VOC analysis of the 37 *Streptomyces* isolates using MSHub/GNPS. 13

Table S4. VOCs annotated with high confidence in the pooled culture screen using MSHub/GNPS. 75

Table S5. Bacterially derived compounds annotated in pooled culture VOC analysis of the 37 *Streptomyces* isolates using the conventional method. 116

Table S6. VOCs annotated with high confidence in the pooled culture screen using conventional method. 126

Table S7. Bacterially derived compounds annotated in replicate VOC analysis of the six selceted *Streptomyces* isolates using the MSHub/GNPS 129

Table S8. Bacterially derived compounds annotated in replicate VOC analysis of the six selceted *Streptomyces* isolates using the conventional method.. 140

Table S9. Web links for MSHub/GNPS jobs generated in this study. 145

REFERENCES 146

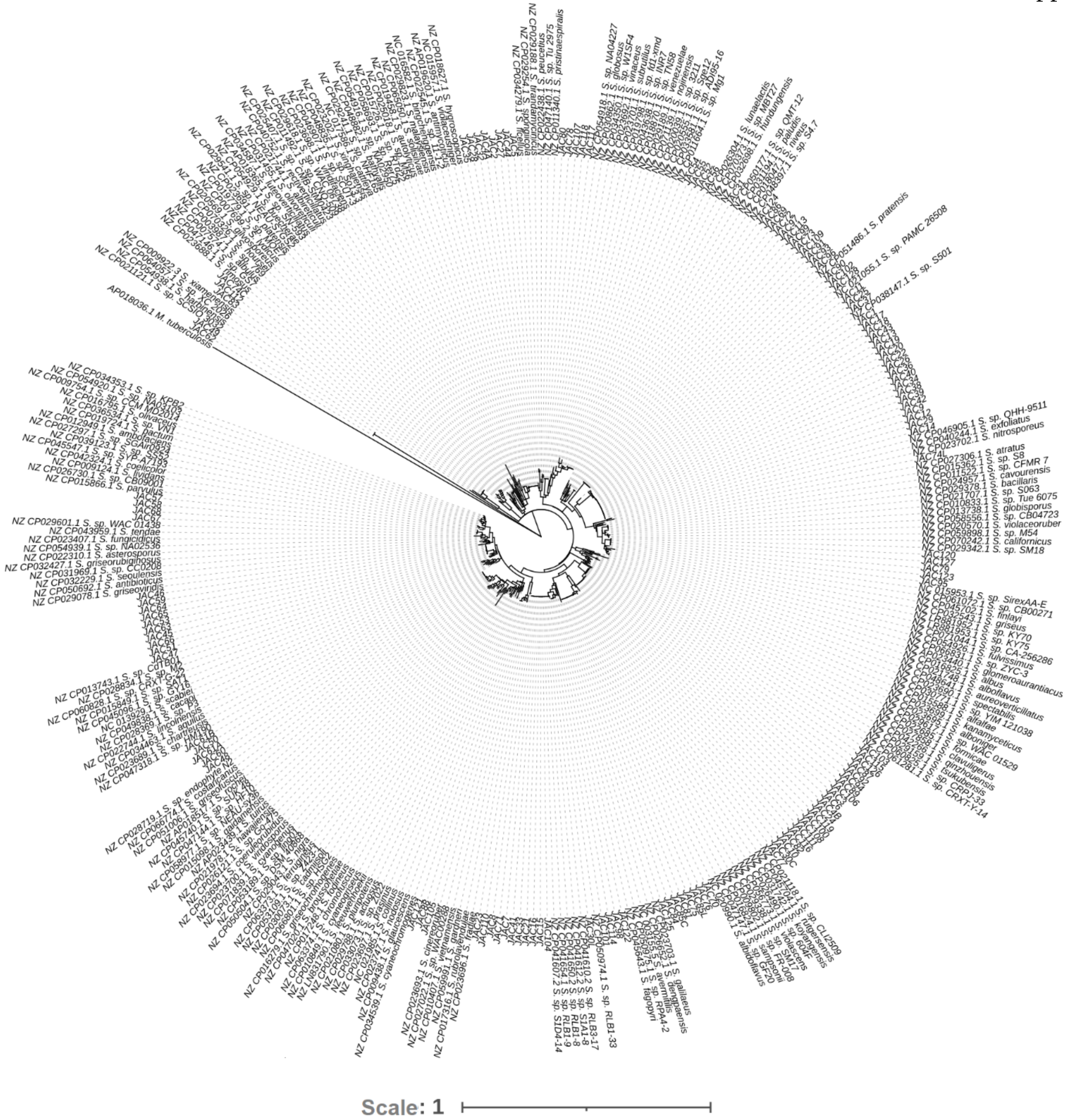
Scale: 1

Figure S1. Phylogenetic relationship between *Streptomyces* isolates from Newfoundland and other *Streptomyces* whose complete genome sequences are available from NCBI for download (n=211). The tree consists of 345 *rpoB* gene sequences, including 131 sequences from this study. The tree was constructed using 880 bp nucleotide sequence lengths with 100 bootstrap replicates. A minimum of 50% consensus cut-off value was used to create the branches of the tree. The scale bar indicates the number of nucleotide substitutions per site and *M. tuberculosis* served as an outgroup.

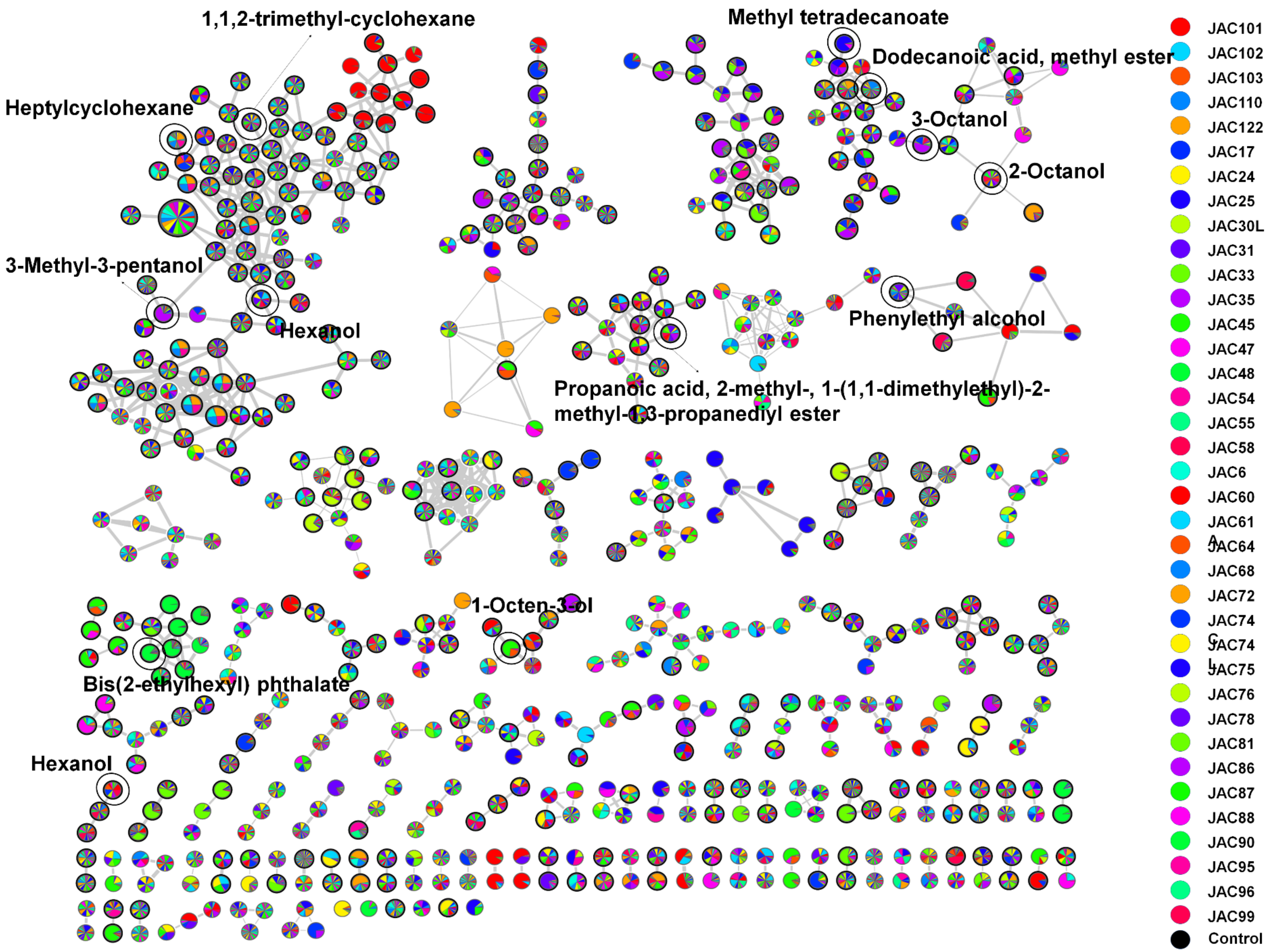
1,1,2-trimethyl-cyclohexane
Methyl tetradecanoate
Dodecanoic acid, methyl ester
Heptylcyclohexane
3-Octanol
2-Octanol
3-Methyl-3-pentanol
Hexanol
Phenylethyl alcohol
Propanoic acid, 2-methyl-, 1-(1,1-dimethylethyl)-2-methyl-1,3-propanediyl ester
1-Octen-3-ol
Bis(2-ethylhexyl) phthalate
Hexanol
JAC101
JAC102
JAC103
JAC110
JAC122
JAC17
JAC24
JAC25
JAC30L
JAC31
JAC33
JAC35
JAC45
JAC47
JAC48
JAC54
JAC55
JAC58
JAC6
JAC60
JAC61
JAC64
JAC68
JAC72
JAC74
JAC74
JAC75
JAC76
JAC78
JAC81
JAC86
JAC87
JAC88
JAC90
JAC95
JAC96
JAC99
Control

Figure S2. Molecular network generated using data from cultures of 37 *Streptomyces* isolates (cosine 0.6). Each node represents one fragmentation spectrum and node size represents the summed intensity (peak area) of the ion from all samples. Nodes that have matches in GNPS spectral libraries are outlined in black. The pie charts indicate the relative abundance of each compound in the different isolate samples. The VOCs annotated by both methods (and bis(2-ethylhexyl) phthalate) are labelled and indicated with circles.

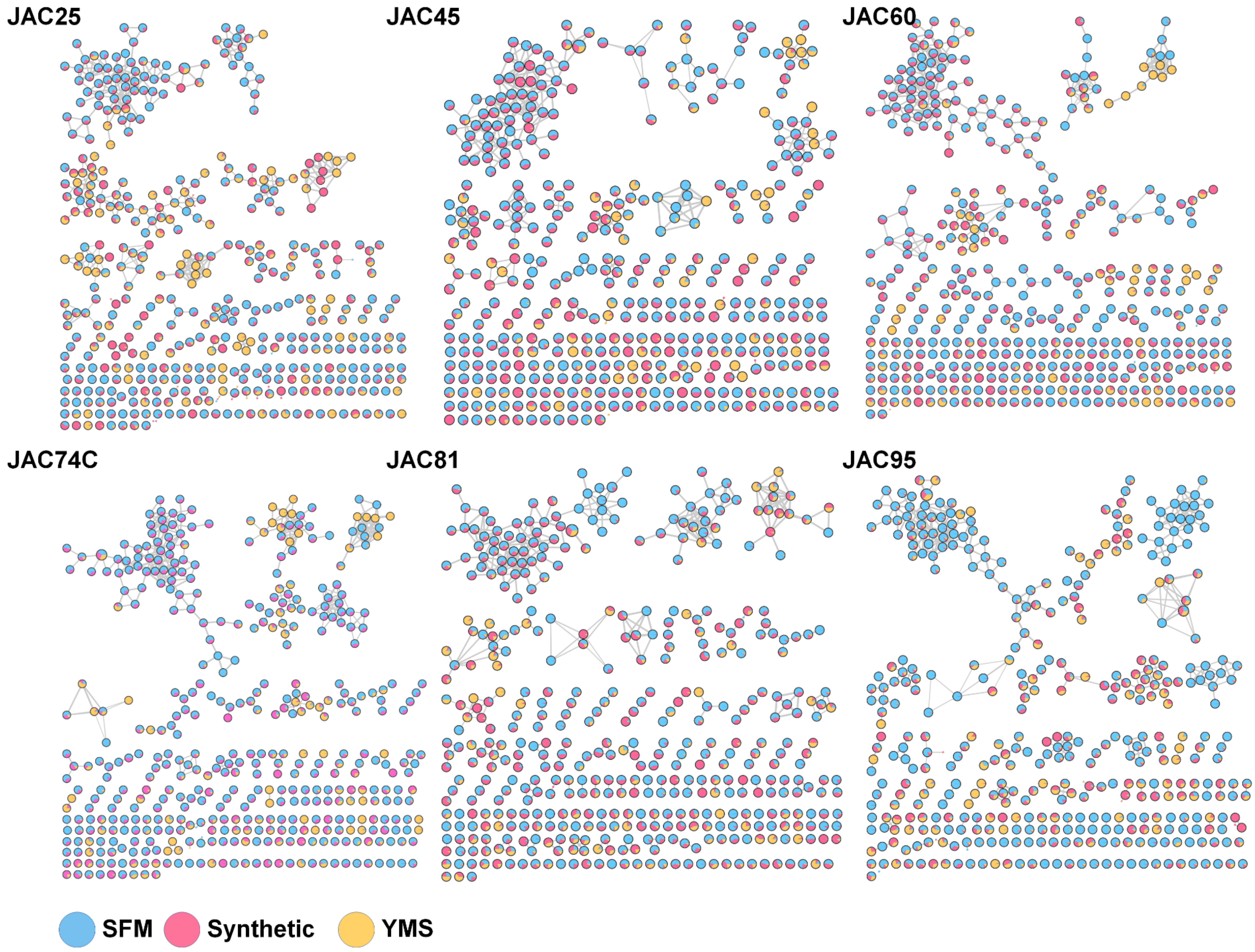
JAC25
JAC45
JAC60
JAC74C
JAC81
JAC95
SFM
Synthetic
YMS

Figure S3. Molecular network generated using replicate cultures of the six selected *Streptomyces* isolates (cosine 0.6). Each node represents one fragmentation spectrum and node size represents the summed intensity (peak area) of the ion from all samples. Nodes that have matches in GNPS spectral libraries are outlined in black. The pie charts indicate the relative abundance of each compound in the cultures of the respective isolates grown in the different media.

Table S1. Oligonucleotide primers used for PCR amplification and DNA sequencing in the current study.

Primer name	Sequence (5'- 3')	Annealing temperature +	Purpose	Reference
27F	AGAGTTTGATCCTGGCTCAG	59	Amplifying 1465 bp of the 16s rDNA gene and sequencing for molecular identification	(1)
1492R	ACGGCTACCTTGTTACGACT			
516F	TGCCAGCAGCCGCGGTA	-	Sequencing 16s rDNA gene amplicons for molecular identification	(2)
rpoBF1	TTCATGGACCAGAACAACC	58	Amplifying 880 bp of the *rpoB* gene for use in phylogeny	(3)
rpoBR1	CGTAGTTGTGACCCTCCC			
rpoBF2	CCAGAACAACCCGCT	-	Sequencing *rpoB* gene amplicons for phylogeny	This Study
rpoBR2	CTCCCACGGCATGAA			

+ Annealing temperatures were calculated using the NEB online Tm calculator (https://tmcalculator.neb.com/#!/main) for the Phusion system.

- means not applicable.

Table S2. Core VOCs annotated in all the 37 *Streptomyces* isolates using the MSHub/GNPS.

Compound/VOC	Retention Time (min)	Molecular Formula	Molecular Weight
**Alcohols**			
1-Octen-3-ol	5.46	$C_8H_{16}O$	128.21
Cis-1,2-Cyclohexanediol	11.52	$C_6H_{12}O_2$	116.16
**Esters**			
Ethyl Undecanoate	8.54	$C_{13}H_{26}O_2$	214.34
Carbonic Acid, Butyl 2-Ethylhexyl Ester	10.15	$C_{13}H_{26}O_3$	230.34
Dodecyl Formate	11.72	$C_{13}H_{26}O_2$	214.34
Methyl Para Toluate	13.72	$C_9H_{10}O_2$	150.17
2-Butenedioic Acid (*Z*)-, Monododecyl Ester	19.06	$C_{16}H_{28}O_4$	284.39
Carbonic Acid, 2,2,2-Trichloroethyl 2-Ethylhexyl Ester	19.42	$C_{11}H_{19}Cl_3O_3$	305.6
Dodecanoic Acid, Methyl Ester	19.7	$C_{13}H_{26}O_2$	214.34
Hexanoic Acid, 2-Hexenyl Ester, (*E*)-	20.46	$C_{12}H_{22}O_2$	198.3
3-Octyl Isovalerate	21.22	$C_{13}H_{26}O_2$	214.34
13-Methyltetradecanoic Acid Methyl Ester	22.1	$C_{16}H_{32}O_2$	256.42
Allyl Hexanoate	22.37	$C_9H_{16}O_2$	156.22
Methyl Decanoate	24.22	$C_{11}H_{22}O_2$	186.29
Bis(2-Ethylhexyl) Phthalate	28.42	$C_{24}H_{38}O_4$	390.6
**Hydrocarbons**			

N-Octadecane	13.22	$C_{18}H_{38}$	254.5
Cyclohexane, 1,2,3-Trimethyl-	14.56	C_9H_{18}	126.24
Cyclopentadecane	17.2	$C_{15}H_{30}$	210.4
Cyclohexane, Tetradecyl-	21.76	$C_{20}H_{40}$	280.5
**Ketones**			
Pentanone (4-OH-4-Me-2-)	5.57	$C_6H_{12}O_2$	116.16
2-Tert-Butylcyclohexanone	11.47	$C_{10}H_{18}O$	154.25
Ethanone, 2-Chloro-1-(2,4-Dimethylphenyl)-	11.57	$C_{10}H_{11}ClO$	182.64
**Terpenes and Terpenoids**			
Cyclohexanol, 5-Methyl-2-(1-Methylethyl)-	12.48	$C_{10}H_{20}O$	156.26
1,1-Bis(4,4-Dimethyl-2,6-Dioxocyclohexyl)Ethane	14.07	$C_{18}H_{26}O_4$	306.4
Beta-Gurjunene	17.77	$C_{15}H_{24}$	204.35
Cubebene (Alpha-)	21.55	$C_{15}H_{24}$	204.35
**Diverse functional groups**			
(*Z*,*Z*)-12,15-Octadecadienoic Acid Methyl Ester	6.04	$C_{19}H_{34}O_2$	294.5
Para-Bromo toluene	6.25	C_7H_7Br	171.03
(+)-*N*-Benzyl-.Alpha.-Phenethylamine	8.46	$C_{15}H_{17}N$	211.3
Oleic Acid	8.74	$C_{18}H_{34}O_2$	282.5
3-Methyl-P-Anisaldehyde	10.29	$C_9H_{10}O_2$	150.17
1,2-Benzenedicarboxaldehyde	11.05	$C_8H_6O_2$	134.13
Docosyl Pentyl Ether	11.96	$C_{27}H_{56}O$	396.7
Docosanoic Anhydride	12.19	$C_{44}H_{86}O_3$	663.2

3Beta-Acetoxy-20-Hydroxy-5Alpha-Cevan-6-One	13.97	$C_{29}H_{45}NO_4$	471.7
Heptane, 3-[(Ethenyloxy)Methyl]-	16.45	$C_{10}H_{20}O$	156.26
Heptyl Tetradecyl Ether	17.04	$C_{21}H_{44}O$	312.6
Behenyl Chloride	18.2	$C_{22}H_{45}Cl$	345
N-((Methylphenylamino)Methyl)Benzamide	18.38	$C_{15}H_{16}N_2O$	240.3
4-Benzylaminoindole	20.3	$C_{15}H_{14}N_2$	222.28
Cis-1-Chloro-9-Octadecene	24.92	$C_{18}H_{35}Cl$	286.9
Carbonochloridic Acid, Heptyl Ester	26.67	$C_8H_{15}ClO_2$	178.65
Benzoic Acid Mono-Tms	26.8	$C_{10}H_{14}O_2Si$	194.3

Table S3. Bacterially derived compounds annotated in pooled culture VOC analysis of the 37 *Streptomyces* isolates using MSHub/GNPS.

Compound/VOC	Confidence in annotation	Retention time (min)	Annotated in (isolates)
**Alcohols (27)**			
Dihydromyrcenol	High	4.7	JAC33,JAC60,JAC102,JAC99,JAC47,JAC45,JAC64,JAC103,JAC74C,JAC48,JAC17,JAC78,JAC76,JAC86
12-Bromo-1-Dodecanol	Low	5.42	JAC30L,JAC99,JAC90,JAC81,JAC75,JAC78,JAC96,JAC86,JAC95,JAC35,JAC88,JAC68,JAC110,JAC74L,JAC54,JAC76,JAC60,JAC31,JAC61A,JAC122,JAC72,JAC25,JAC102,JAC87,JAC17
1-Octen-3-ol	High	5.46	JAC33,JAC60,JAC87,JAC102,JAC96,JAC88,JAC55,JAC99,JAC75,JAC47,JAC54,JAC31,JAC61A,JAC45,JAC64,JAC103,JAC74C,JAC48,JAC95,JAC25,JAC101,JAC122,JAC17,JAC78,JAC68,JAC72,JAC81,JAC30L,JAC76,JAC58,JAC86,JAC90,JAC74L,JAC6,JAC110,JAC24,JAC35
Octanol (2-)	High	6.35	JAC33,JAC60,JAC87,JAC102,JAC96,JAC88,JAC55,JAC99,JAC75,JAC47,JAC54,JAC31,JAC61A,JAC45,JAC64,JAC103,JAC74C,JAC48,JAC95,JAC25,JAC101,JAC122,JAC17,JAC78,JAC68,JAC72,JAC81,JAC30L,JAC76,JAC58,JAC86,JAC90,JAC74L,JAC6,JAC110,JAC24,JAC35
6,10-Dimethyl-2-Undecanol	High	6.62	JAC33,JAC60,JAC102,JAC99,JAC47,JAC31,JAC45,JAC64,JAC103,JAC74C,JAC48,JAC25,JAC101,JAC17,JAC78,JAC68,JAC72,JAC30L,JAC76,JAC58,JAC86,JAC24
3-Hexanol, 3,5-Dimethyl-	High	8.03	JAC33,JAC60,JAC102,JAC88,JAC55,JAC99,JAC47,JAC54,JAC31,JAC61A,JAC103,JAC74C,JAC48,JAC2

			5,JAC101,JAC17,JAC78,JAC72,JAC30L,JAC76,JAC58,JAC86,JAC74L,JAC6,JAC110,JAC24
2-Decanol	High	8.26	JAC33,JAC87,JAC88,JAC55,JAC99,JAC61A,JAC45,JAC103,JAC74C,JAC95,JAC101,JAC122,JAC68,JAC81,JAC30L,JAC76,JAC58,JAC86,JAC6,JAC110,JAC35
Octanol (3-)	High	9.39	JAC33,JAC60,JAC99,JAC75,JAC47,JAC45,JAC64,JAC103,JAC74C,JAC48,JAC17,JAC78,JAC81,JAC76,JAC58,JAC86,JAC6
1-Phenylethyl Alcohol	High	10.29	JAC33,JAC60,JAC87,JAC102,JAC96,JAC88,JAC55,JAC99,JAC75,JAC47,JAC54,JAC31,JAC61A,JAC64,JAC103,JAC74C,JAC48,JAC95,JAC25,JAC101,JAC122,JAC17,JAC78,JAC68,JAC72,JAC81,JAC30L,JAC76,JAC58,JAC86,JAC90,JAC74L,JAC6,JAC110,JAC24,JAC35
2-Hexanol, 2,5-Dimethyl-, (*S*)-	High	10.63	JAC33,JAC60,JAC87,JAC102,JAC96,JAC88,JAC55,JAC99,JAC75,JAC47,JAC54,JAC31,JAC61A,JAC45,JAC103,JAC48,JAC95,JAC25,JAC122,JAC17,JAC78,JAC68,JAC72,JAC81,JAC30L,JAC76,JAC58,JAC90,JAC74L,JAC6,JAC110,JAC24,JAC35
1-Tetradecanol	High	11.29	JAC33,JAC60,JAC87,JAC102,JAC88,JAC55,JAC99,JAC75,JAC61A,JAC45,JAC103,JAC48,JAC95,JAC101,JAC78,JAC72,JAC81,JAC30L,JAC76,JAC58,JAC90,JAC74L,JAC110
Cis-1,2-Cyclohexanediol	High	11.52	JAC33,JAC60,JAC87,JAC102,JAC96,JAC88,JAC55,JAC99,JAC75,JAC47,JAC54,JAC31,JAC61A,JAC45,JAC64,JAC103,JAC74C,JAC48,JAC95,JAC25,JAC101,JAC122,JAC17,JAC78,JAC68,JAC72,JAC30L,JAC76,JAC58,JAC86,JAC90,JAC6,JAC110,JAC24,JAC35
1,3-Propanediol, 2-Butyl-2-Ethyl-	High	12.58	JAC33,JAC60,JAC87,JAC102,JAC96,JAC88,JAC55,JAC99,JAC75,JAC47,JAC54,JAC31,JAC61A,JAC45,JAC64,JAC103,JAC74C,JAC48,JAC95,JAC25,JAC101

			,JAC122,JAC17,JAC78,JAC68,JAC81,JAC30L,JAC76,JAC86,JAC90,JAC74L,JAC6,JAC110,JAC24,JAC35
1-Undecanol, 11-Bromo-	High	12.58	JAC33,JAC60,JAC87,JAC102,JAC96,JAC88,JAC55,JAC99,JAC75,JAC47,JAC54,JAC31,JAC61A,JAC64,JAC103,JAC74C,JAC48,JAC95,JAC25,JAC101,JAC122,JAC17,JAC78,JAC68,JAC72,JAC81,JAC30L,JAC76,JAC58,JAC86,JAC90,JAC74L,JAC6,JAC110,JAC35
Hexanol	High	12.99	JAC60,JAC87,JAC102,JAC96,JAC88,JAC55,JAC99,JAC75,JAC47,JAC54,JAC31,JAC61A,JAC45,JAC103,JAC48,JAC95,JAC25,JAC101,JAC122,JAC17,JAC78,JAC68,JAC72,JAC81,JAC30L,JAC76,JAC58,JAC90,JAC74L,JAC6,JAC110,JAC35
(*S*)-(+)-3-Methyl-1-Pentanol	High	13.09	JAC33,JAC60,JAC102,JAC99,JAC75,JAC47,JAC54,JAC31,JAC45,JAC64,JAC103,JAC74C,JAC48,JAC25,JAC101,JAC17,JAC78,JAC72,JAC81,JAC30L,JAC76,JAC58,JAC86,JAC24,JAC35
2-Isopropyl-5-Methyl-1-Heptanol	High	13.66	JAC33,JAC60,JAC87,JAC102,JAC88,JAC99,JAC75,JAC47,JAC54,JAC31,JAC45,JAC64,JAC74C,JAC95,JAC25,JAC101,JAC17,JAC78,JAC72,JAC76,JAC58,JAC86,JAC24,JAC35
Tetrahydrolavandulol	High	15.41	JAC60,JAC87,JAC102,JAC88,JAC55,JAC99,JAC75,JAC47,JAC31,JAC64,JAC103,JAC74C,JAC48,JAC25,JAC101,JAC122,JAC68,JAC72,JAC81,JAC76,JAC58,JAC86,JAC6,JAC35
10-Undecen-1-ol	High	17.86	JAC33,JAC87,JAC102,JAC96,JAC88,JAC55,JAC99,JAC75,JAC47,JAC54,JAC31,JAC61A,JAC64,JAC103,JAC74C,JAC48,JAC95,JAC122,JAC17,JAC78,JAC68,JAC72,JAC81,JAC30L,JAC76,JAC58,JAC86,JAC90,JAC74L,JAC6,JAC110,JAC24,JAC35
12-Methyl-1-Tridecanol	High	17.91	JAC33,JAC60,JAC87,JAC102,JAC96,JAC88,JAC55,JAC99,JAC75,JAC47,JAC54,JAC31,JAC61A,JAC45,J

			AC64,JAC103,JAC74C,JAC48,JAC95,JAC25,JAC101,JAC122,JAC17,JAC78,JAC68,JAC72,JAC81,JAC30L,JAC76,JAC58,JAC86,JAC90,JAC74L,JAC6,JAC110,JAC24,JAC35
1-Pentadecanol	High	19.1	JAC33,JAC60,JAC87,JAC102,JAC96,JAC88,JAC55,JAC99,JAC75,JAC47,JAC54,JAC31,JAC61A,JAC103,JAC74C,JAC48,JAC95,JAC25,JAC122,JAC17,JAC78,JAC68,JAC72,JAC81,JAC30L,JAC58,JAC86,JAC90,JAC74L,JAC6,JAC110,JAC24,JAC35
Tridecanol	High	19.8	JAC33,JAC60,JAC87,JAC102,JAC96,JAC88,JAC55,JAC99,JAC75,JAC47,JAC54,JAC31,JAC61A,JAC45,JAC103,JAC74C,JAC95,JAC122,JAC17,JAC78,JAC68,JAC72,JAC81,JAC30L,JAC76,JAC58,JAC90,JAC74L,JAC6,JAC110,JAC24,JAC35
Methyl 2-Hydroxydodecanoate	Low	20.76	JAC30L,JAC74C,JAC90,JAC47,JAC78,JAC96,JAC86,JAC48,JAC103,JAC35,JAC68,JAC110,JAC45,JAC33,JAC64,JAC74L,JAC76,JAC60,JAC61A,JAC122,JAC58,JAC72,JAC102,JAC87,JAC17,JAC24
Methyl (5*Z*,8*Z*,11*Z*,14*Z*)-Eicosatetraenoate	Low	21.85	JAC30L,JAC74C,JAC90,JAC81,JAC75,JAC47,JAC78,JAC96,JAC48,JAC35,JAC88,JAC101,JAC68,JAC110,JAC45,JAC33,JAC64,JAC74L,JAC54,JAC6,JAC60,JAC31,JAC61A,JAC58,JAC72,JAC25,JAC87,JAC24,JAC55
3-Pentadecanol	High	22.14	JAC87,JAC102,JAC96,JAC88,JAC55,JAC75,JAC54,JAC31,JAC61A,JAC74C,JAC101,JAC68,JAC72,JAC81,JAC30L,JAC58,JAC86,JAC90,JAC74L,JAC110,JAC24,JAC35
10-Methyl-1-Dodecanol	High	22.87	JAC33,JAC60,JAC87,JAC102,JAC96,JAC88,JAC55,JAC99,JAC75,JAC47,JAC54,JAC31,JAC61A,JAC64,JAC103,JAC74C,JAC48,JAC95,JAC25,JAC101,JAC122,JAC17,JAC78,JAC68,JAC72,JAC81,JAC30L,JAC76

			,JAC58,JAC86,JAC90,JAC74L,JAC6,JAC110,JAC24,JAC35
7-Ethyl-2-Methylundecan-1-ol	Low	28.04	JAC30L,JAC99,JAC74C,JAC90,JAC81,JAC75,JAC47,JAC78,JAC96,JAC86,JAC48,JAC95,JAC103,JAC35,JAC88,JAC101,JAC68,JAC110,JAC45,JAC33,JAC64,JAC74L,JAC54,JAC6,JAC76,JAC60,JAC31,JAC61A,JAC122,JAC58,JAC72,JAC25,JAC102,JAC87,JAC17,JAC24,JAC55
**Aldehydes (6)**			
Benzeneacetaldehyde	High	12.44	JAC33,JAC60,JAC87,JAC96,JAC88,JAC55,JAC99,JAC75,JAC47,JAC54,JAC61A,JAC45,JAC64,JAC103,JAC74C,JAC48,JAC95,JAC25,JAC101,JAC122,JAC68,JAC72,JAC76,JAC58,JAC86,JAC90,JAC110,JAC24,JAC35
Perillyl Aldehyde	Low	13.53	JAC30L,JAC81,JAC75,JAC47,JAC96,JAC86,JAC95,JAC35,JAC88,JAC101,JAC45,JAC33,JAC64,JAC74L,JAC76,JAC60,JAC31,JAC61A,JAC58,JAC72,JAC102,JAC24,JAC55
(*Z*)-11-Hexadecenal	High	15.71	JAC60,JAC102,JAC99,JAC47,JAC31,JAC45,JAC64,JAC25,JAC101,JAC17,JAC78,JAC72,JAC81,JAC58,JAC24
Cis-9-Hexadecenal	High	23.94	JAC33,JAC60,JAC87,JAC102,JAC88,JAC99,JAC75,JAC47,JAC31,JAC61A,JAC45,JAC64,JAC103,JAC74C,JAC48,JAC95,JAC101,JAC122,JAC17,JAC78,JAC68,JAC72,JAC76,JAC58,JAC86,JAC6,JAC110,JAC24,JAC35
Nonadecanoic Acid	Low	24.26	JAC30L,JAC99,JAC74C,JAC90,JAC81,JAC75,JAC47,JAC78,JAC96,JAC86,JAC48,JAC95,JAC103,JAC35,JAC88,JAC101,JAC68,JAC110,JAC45,JAC33,JAC64,JAC74L,JAC54,JAC6,JAC76,JAC60,JAC31,JAC61A,J

			AC122,JAC58,JAC72,JAC102,JAC87,JAC17,JAC24,JAC55
Nonanal	Low	28.42	JAC30L,JAC99,JAC74C,JAC81,JAC75,JAC47,JAC78,JAC96,JAC86,JAC48,JAC95,JAC103,JAC35,JAC88,JAC101,JAC68,JAC110,JAC45,JAC33,JAC64,JAC74L,JAC54,JAC6,JAC76,JAC60,JAC31,JAC61A,JAC122,JAC58,JAC72,JAC25,JAC102,JAC87,JAC17,JAC24,JAC55
**Esters (62)**			
Cyclohexanecarboxylic Acid, 4-Methoxy-, Heptyl Ester	Low	7.83	JAC99,JAC74C,JAC90,JAC47,JAC78,JAC96,JAC48,JAC103,JAC35,JAC88,JAC101,JAC110,JAC45,JAC33,JAC64,JAC74L,JAC6,JAC76,JAC60,JAC31,JAC61A,JAC58,JAC72,JAC102,JAC87,JAC17,JAC24,JAC55
Ethyl Undecanoate	High	8.54	JAC33,JAC60,JAC87,JAC102,JAC96,JAC88,JAC55,JAC99,JAC75,JAC47,JAC54,JAC31,JAC61A,JAC45,JAC64,JAC103,JAC74C,JAC48,JAC95,JAC25,JAC101,JAC122,JAC17,JAC78,JAC68,JAC72,JAC81,JAC30L,JAC76,JAC58,JAC86,JAC90,JAC74L,JAC6,JAC110,JAC24,JAC35
5-Hydroxy-3-Methoxy-2,6-Dimethyl-(6*R*)-((2*R*)-2-Methylbutyryloxy)-2,4-Cyclohexadien-1-One	Low	9.18	JAC30L,JAC99,JAC74C,JAC81,JAC75,JAC47,JAC78,JAC86,JAC48,JAC95,JAC103,JAC35,JAC101,JAC68,JAC45,JAC64,JAC6,JAC76,JAC60,JAC31,JAC122,JAC58,JAC72,JAC25,JAC102,JAC87,JAC24
Methacrylic Acid 2-Ethylhexyl Ester	High	9.61	JAC60,JAC87,JAC96,JAC88,JAC55,JAC99,JAC75,JAC47,JAC54,JAC45,JAC64,JAC48,JAC95,JAC25,JAC101,JAC17,JAC78,JAC68,JAC72,JAC81,JAC76,JAC86,JAC90,JAC74L,JAC6,JAC110
Carbonic Acid, Butyl 2-Ethylhexyl Ester	High	10.15	JAC33,JAC60,JAC87,JAC102,JAC96,JAC88,JAC55,JAC99,JAC75,JAC54,JAC31,JAC61A,JAC45,JAC64,JAC103,JAC74C,JAC48,JAC95,JAC101,JAC122,JAC1

			7,JAC78,JAC68,JAC72,JAC81,JAC30L,JAC76,JAC58,JAC86,JAC90,JAC74L,JAC6,JAC110,JAC24,JAC35
Octadecyl 3-(3,5-Di-Tert-Butyl-4-Hydroxyphenyl)Propionate	High	10.21	JAC33,JAC60,JAC87,JAC102,JAC96,JAC88,JAC55,JAC99,JAC75,JAC54,JAC31,JAC61A,JAC64,JAC103,JAC74C,JAC48,JAC95,JAC25,JAC101,JAC122,JAC17,JAC78,JAC72,JAC81,JAC76,JAC58,JAC86,JAC90,JAC74L,JAC110,JAC24,JAC35
2-Acetoxyisobutyryl Chloride	High	10.63	JAC33,JAC60,JAC87,JAC102,JAC88,JAC55,JAC99,JAC75,JAC47,JAC54,JAC31,JAC45,JAC64,JAC103,JAC74C,JAC48,JAC95,JAC25,JAC101,JAC17,JAC78,JAC68,JAC72,JAC30L,JAC76,JAC58,JAC86,JAC74L,JAC24
Hexyl Acetate	High	11.29	JAC33,JAC60,JAC87,JAC102,JAC96,JAC88,JAC55,JAC99,JAC75,JAC47,JAC54,JAC31,JAC61A,JAC45,JAC64,JAC103,JAC74C,JAC48,JAC95,JAC25,JAC101,JAC122,JAC17,JAC78,JAC68,JAC72,JAC81,JAC30L,JAC76,JAC58,JAC86,JAC90,JAC74L,JAC6,JAC110,JAC24,JAC35
Dodecyl Formate	High	11.72	JAC33,JAC60,JAC87,JAC102,JAC96,JAC88,JAC55,JAC99,JAC75,JAC47,JAC54,JAC31,JAC61A,JAC45,JAC64,JAC103,JAC48,JAC95,JAC25,JAC122,JAC17,JAC78,JAC68,JAC72,JAC81,JAC30L,JAC76,JAC58,JAC90,JAC74L,JAC6,JAC110,JAC24,JAC35
Hexyl 10-Undecenoate	High	11.85	JAC33,JAC60,JAC87,JAC102,JAC96,JAC88,JAC55,JAC99,JAC75,JAC47,JAC54,JAC31,JAC61A,JAC45,JAC64,JAC103,JAC74C,JAC48,JAC95,JAC25,JAC101,JAC122,JAC17,JAC78,JAC68,JAC72,JAC81,JAC30L,JAC76,JAC58,JAC86,JAC90,JAC74L,JAC6,JAC110,JAC24,JAC35
9-Octadecenoic Acid, Methyl Ester, (*E*)-	Low	12.94	JAC30L,JAC99,JAC74C,JAC90,JAC81,JAC75,JAC47,JAC78,JAC96,JAC86,JAC48,JAC95,JAC103,JAC35,JAC101,JAC110,JAC45,JAC33,JAC64,JAC74L,JAC54,

			JAC76,JAC60,JAC31,JAC61A,JAC122,JAC58,JAC72,JAC25,JAC102,JAC87,JAC17,JAC24,JAC55
(*Z*)-12-Octadecenoic Acid Methyl Ester	High	13.33	JAC33,JAC60,JAC87,JAC102,JAC96,JAC88,JAC55,JAC99,JAC75,JAC47,JAC54,JAC31,JAC61A,JAC45,JAC64,JAC103,JAC74C,JAC48,JAC95,JAC25,JAC101,JAC122,JAC17,JAC78,JAC68,JAC72,JAC81,JAC30L,JAC76,JAC58,JAC86,JAC90,JAC74L,JAC6,JAC110,JAC24,JAC35
Methyl Para Toluate	High	13.72	JAC33,JAC102,JAC88,JAC99,JAC75,JAC47,JAC54,JAC31,JAC61A,JAC64,JAC103,JAC74C,JAC48,JAC95,JAC25,JAC101,JAC122,JAC17,JAC78,JAC81,JAC76,JAC58,JAC86,JAC74L,JAC6,JAC24
Fumaric Acid, Dodecyl Tetradec-3-Enyl Ester	Low	14.65	JAC30L,JAC99,JAC74C,JAC90,JAC81,JAC75,JAC47,JAC78,JAC96,JAC86,JAC48,JAC95,JAC103,JAC35,JAC88,JAC68,JAC110,JAC45,JAC33,JAC64,JAC74L,JAC54,JAC6,JAC76,JAC60,JAC31,JAC61A,JAC122,JAC58,JAC72,JAC25,JAC102,JAC87,JAC17,JAC24,JAC55
(*Z*,*Z*)-7,11-Hexadecadienyl Acetate	Low	14.73	JAC30L,JAC99,JAC74C,JAC90,JAC81,JAC75,JAC47,JAC78,JAC96,JAC86,JAC48,JAC95,JAC35,JAC88,JAC101,JAC68,JAC110,JAC45,JAC33,JAC64,JAC74L,JAC54,JAC76,JAC60,JAC31,JAC61A,JAC122,JAC58,JAC72,JAC25,JAC102,JAC87,JAC17,JAC24,JAC55
Glutaric Acid, Dec-2-Yl Dec-4-Enyl Ester	High	15.41	JAC33,JAC60,JAC87,JAC102,JAC96,JAC88,JAC55,JAC99,JAC75,JAC47,JAC54,JAC31,JAC61A,JAC45,JAC64,JAC103,JAC74C,JAC48,JAC95,JAC25,JAC101,JAC122,JAC17,JAC78,JAC68,JAC72,JAC81,JAC30L,JAC76,JAC58,JAC86,JAC90,JAC74L,JAC6,JAC110,JAC24,JAC35

1,2-Dibutyroxy-1-Ethoxyethane	High	16.85	JAC33,JAC60,JAC102,JAC99,JAC75,JAC47,JAC31,JAC45,JAC64,JAC103,JAC74C,JAC48,JAC25,JAC101,JAC17,JAC78,JAC76,JAC58,JAC86,JAC24
2-Butenedioic Acid (*Z*)-, Monododecyl Ester	High	19.06	JAC33,JAC60,JAC87,JAC102,JAC96,JAC88,JAC55,JAC99,JAC75,JAC47,JAC54,JAC31,JAC61A,JAC45,JAC64,JAC103,JAC74C,JAC48,JAC95,JAC25,JAC122,JAC17,JAC78,JAC68,JAC72,JAC81,JAC30L,JAC76,JAC58,JAC86,JAC90,JAC74L,JAC6,JAC110,JAC24
Decan-1,10-Diol Dimethacrylate	High	19.13	JAC33,JAC60,JAC87,JAC102,JAC96,JAC55,JAC99,JAC47,JAC54,JAC31,JAC61A,JAC45,JAC64,JAC103,JAC74C,JAC48,JAC25,JAC101,JAC122,JAC17,JAC78,JAC68,JAC81,JAC76,JAC58,JAC86,JAC90,JAC74L,JAC6,JAC110,JAC35
Carbonic Acid, 2,2,2-Trichloroethyl 2-Ethylhexyl Ester	High	19.42	JAC60,JAC87,JAC102,JAC96,JAC75,JAC47,JAC31,JAC45,JAC103,JAC74C,JAC48,JAC95,JAC25,JAC101,JAC122,JAC17,JAC78,JAC68,JAC72,JAC81,JAC30L,JAC76,JAC58,JAC86,JAC90,JAC74L,JAC6,JAC110,JAC24,JAC35
2-Ethylhexyl Mercaptoacetate	High	19.46	JAC33,JAC60,JAC102,JAC99,JAC47,JAC31,JAC45,JAC64,JAC103,JAC74C,JAC48,JAC101,JAC17,JAC78,JAC72,JAC76,JAC58,JAC86,JAC24,JAC35
Dodecanoic Acid, Methyl Ester	High	19.7	JAC33,JAC60,JAC87,JAC102,JAC88,JAC55,JAC99,JAC75,JAC47,JAC54,JAC31,JAC61A,JAC45,JAC64,JAC103,JAC74C,JAC48,JAC95,JAC25,JAC101,JAC17,JAC78,JAC68,JAC81,JAC30L,JAC76,JAC58,JAC86,JAC90,JAC6,JAC110,JAC24
Cyanoacetic Acid, Nonyl Ester	High	19.8	JAC33,JAC60,JAC87,JAC102,JAC96,JAC88,JAC55,JAC99,JAC75,JAC47,JAC54,JAC31,JAC61A,JAC45,JAC64,JAC103,JAC48,JAC95,JAC25,JAC122,JAC17,JAC78,JAC68,JAC72,JAC81,JAC30L,JAC76,JAC58,JAC90,JAC74L,JAC6,JAC110,JAC24,JAC35

Carbonic Acid, Monoamide, *N*-Propyl-*N*-Butyl-, 2-Ethylhexyl Ester	High	20.09	JAC87,JAC102,JAC96,JAC55,JAC75,JAC47,JAC54,JAC31,JAC61A,JAC45,JAC68,JAC81,JAC30L,JAC58,JAC86,JAC24
Hexanoic Acid, 2-Hexenyl Ester, (*E*)-	High	20.46	JAC33,JAC60,JAC87,JAC102,JAC96,JAC88,JAC55,JAC99,JAC47,JAC54,JAC31,JAC61A,JAC64,JAC95,JAC25,JAC101,JAC122,JAC68,JAC72,JAC30L,JAC86,JAC90,JAC74L,JAC6,JAC110,JAC24,JAC35
Vinyl 2-Ethylhexanoate	High	20.68	JAC33,JAC60,JAC87,JAC102,JAC96,JAC88,JAC55,JAC99,JAC75,JAC47,JAC54,JAC61A,JAC45,JAC64,JAC103,JAC74C,JAC48,JAC95,JAC25,JAC101,JAC122,JAC17,JAC78,JAC68,JAC72,JAC81,JAC30L,JAC76,JAC58,JAC86,JAC90,JAC74L,JAC6,JAC110,JAC24,JAC35
3-Octyl Isovalerate	High	21.22	JAC33,JAC60,JAC87,JAC102,JAC96,JAC88,JAC55,JAC99,JAC75,JAC47,JAC54,JAC31,JAC61A,JAC45,JAC103,JAC74C,JAC48,JAC95,JAC25,JAC101,JAC122,JAC17,JAC68,JAC72,JAC81,JAC30L,JAC76,JAC86,JAC90,JAC74L,JAC6,JAC110,JAC35
Lauryl Acetate	High	21.41	JAC33,JAC60,JAC102,JAC96,JAC88,JAC99,JAC75,JAC47,JAC54,JAC31,JAC61A,JAC45,JAC64,JAC103,JAC48,JAC95,JAC25,JAC122,JAC17,JAC78,JAC68,JAC72,JAC81,JAC30L,JAC76,JAC58,JAC86,JAC90,JAC110,JAC24,JAC35
Carbonic Acid, Isobutyl Cyclohexyl Ester	High	21.91	JAC33,JAC60,JAC87,JAC102,JAC96,JAC88,JAC55,JAC99,JAC75,JAC47,JAC54,JAC31,JAC61A,JAC45,JAC64,JAC103,JAC74C,JAC48,JAC95,JAC25,JAC101,JAC122,JAC17,JAC78,JAC68,JAC72,JAC81,JAC30L,JAC76,JAC58,JAC86,JAC90,JAC74L,JAC6,JAC110,JAC24,JAC35

Hexanoic Acid, 5-Oxo-, Ethyl Ester	High	21.91	JAC60,JAC87,JAC96,JAC88,JAC55,JAC75,JAC54,JAC74C,JAC95,JAC25,JAC101,JAC122,JAC72,JAC30L,JAC90,JAC74L,JAC110,JAC24,JAC35
Eicosane	Low	21.91	JAC30L,JAC99,JAC74C,JAC90,JAC81,JAC75,JAC96,JAC48,JAC95,JAC35,JAC88,JAC101,JAC68,JAC110,JAC45,JAC33,JAC64,JAC74L,JAC54,JAC6,JAC76,JAC60,JAC31,JAC61A,JAC122,JAC72,JAC25,JAC102,JAC87,JAC17,JAC24,JAC55
13-Methyltetradecanoic Acid Methyl Ester	High	22.1	JAC33,JAC87,JAC102,JAC99,JAC47,JAC31,JAC45,JAC74C,JAC78,JAC76,JAC58,JAC74L,JAC24
Ethyl Tetradecanoate	High	22.14	JAC33,JAC60,JAC87,JAC102,JAC96,JAC88,JAC55,JAC99,JAC75,JAC47,JAC54,JAC31,JAC61A,JAC45,JAC64,JAC103,JAC74C,JAC48,JAC95,JAC25,JAC101,JAC122,JAC17,JAC78,JAC68,JAC72,JAC81,JAC30L,JAC76,JAC58,JAC86,JAC90,JAC74L,JAC6,JAC110,JAC24,JAC35
Methyl Tetradecanoate	High	22.14	JAC33,JAC60,JAC87,JAC102,JAC96,JAC88,JAC55,JAC99,JAC75,JAC47,JAC54,JAC31,JAC61A,JAC45,JAC64,JAC103,JAC74C,JAC48,JAC95,JAC25,JAC101,JAC122,JAC17,JAC78,JAC68,JAC72,JAC30L,JAC76,JAC58,JAC86,JAC90,JAC6,JAC110,JAC24,JAC35
2-(2-Methylpiperidino)Ethyl P-Chlorobenzoate	High	22.28	JAC60,JAC87,JAC102,JAC96,JAC88,JAC55,JAC75,JAC47,JAC54,JAC31,JAC61A,JAC103,JAC48,JAC95,JAC25,JAC101,JAC122,JAC78,JAC68,JAC72,JAC81,JAC58,JAC86,JAC6,JAC24,JAC35
Allyl Hexanoate	High	22.37	JAC33,JAC60,JAC87,JAC102,JAC96,JAC88,JAC55,JAC99,JAC75,JAC47,JAC54,JAC31,JAC61A,JAC45,JAC64,JAC103,JAC74C,JAC48,JAC95,JAC25,JAC101,JAC122,JAC17,JAC78,JAC68,JAC72,JAC81,JAC30L,JAC76,JAC58,JAC86,JAC90,JAC74L,JAC6,JAC110,JAC24,JAC35

Ethyl 3-Oxoheptanoate	Low	22.41	JAC30L,JAC81,JAC47,JAC78,JAC86,JAC68,JAC110,JAC33,JAC6,JAC60,JAC31,JAC122,JAC25,JAC102,JAC17
2-Methylvaleric Acid, 2-Ethylhexyl Ester	High	22.46	JAC33,JAC60,JAC87,JAC102,JAC96,JAC88,JAC55,JAC99,JAC75,JAC47,JAC54,JAC31,JAC61A,JAC45,JAC64,JAC103,JAC74C,JAC48,JAC95,JAC25,JAC101,JAC122,JAC17,JAC78,JAC68,JAC72,JAC81,JAC30L,JAC76,JAC58,JAC86,JAC90,JAC74L,JAC6,JAC110,JAC24,JAC35
Cis-3-Hexenyl 2-Ethylbutyrate	High	22.65	JAC60,JAC87,JAC102,JAC96,JAC88,JAC55,JAC99,JAC75,JAC47,JAC54,JAC61A,JAC64,JAC103,JAC74C,JAC48,JAC95,JAC25,JAC101,JAC122,JAC17,JAC78,JAC68,JAC72,JAC81,JAC30L,JAC76,JAC58,JAC86,JAC90,JAC74L,JAC6,JAC110,JAC24,JAC35
Benzyl Benzoate	High	22.71	JAC33,JAC60,JAC87,JAC102,JAC96,JAC88,JAC55,JAC99,JAC75,JAC47,JAC54,JAC31,JAC61A,JAC45,JAC64,JAC103,JAC74C,JAC48,JAC95,JAC25,JAC101,JAC122,JAC17,JAC78,JAC68,JAC72,JAC81,JAC30L,JAC76,JAC58,JAC86,JAC90,JAC74L,JAC6,JAC110,JAC24,JAC35
Methyl Dodecanoate	High	23.22	JAC87,JAC96,JAC55,JAC99,JAC75,JAC47,JAC54,JAC61A,JAC45,JAC64,JAC74C,JAC95,JAC122,JAC68,JAC72,JAC81,JAC30L,JAC76,JAC90,JAC74L,JAC6,JAC110,JAC35
Fumaric Acid, 2-Ethylhexyl 2,2,3,3-Tetrafluoropropyl Ester	Low	23.78	JAC30L,JAC99,JAC74C,JAC90,JAC81,JAC75,JAC47,JAC78,JAC96,JAC86,JAC48,JAC95,JAC103,JAC35,JAC88,JAC68,JAC110,JAC45,JAC33,JAC74L,JAC54,JAC6,JAC76,JAC31,JAC61A,JAC122,JAC58,JAC72,JAC25,JAC17,JAC24,JAC55
Heptadecane, 1-Bromo-	Low	24.1	JAC30L,JAC99,JAC74C,JAC90,JAC81,JAC75,JAC78,JAC96,JAC86,JAC48,JAC95,JAC103,JAC35,JAC88,JAC101,JAC68,JAC110,JAC45,JAC33,JAC64,JAC74L,

			JAC54,JAC6,JAC76,JAC60,JAC31,JAC61A,JAC122,JAC58,JAC72,JAC25,JAC102,JAC87,JAC17,JAC24,JAC55
Trans-2-Hexenyl 2-Ethylbutyrate	High	24.14	JAC33,JAC60,JAC87,JAC102,JAC96,JAC88,JAC55,JAC99,JAC75,JAC47,JAC54,JAC31,JAC61A,JAC45,JAC103,JAC74C,JAC48,JAC95,JAC25,JAC101,JAC122,JAC17,JAC78,JAC68,JAC72,JAC81,JAC30L,JAC76,JAC86,JAC90,JAC74L,JAC6,JAC110,JAC35
Methyl Decanoate	High	24.22	JAC87,JAC102,JAC55,JAC99,JAC47,JAC61A,JAC45,JAC64,JAC103,JAC74C,JAC95,JAC25,JAC122,JAC17,JAC78,JAC68,JAC72,JAC58,JAC86,JAC90,JAC74L,JAC110,JAC35
Methyl Palmitate	High	24.26	JAC33,JAC87,JAC96,JAC88,JAC55,JAC75,JAC47,JAC54,JAC61A,JAC45,JAC95,JAC25,JAC122,JAC68,JAC72,JAC81,JAC30L,JAC90,JAC74L,JAC6,JAC110,JAC35
Isopropyl Hexadecanoate	High	25.21	JAC33,JAC87,JAC102,JAC96,JAC88,JAC55,JAC99,JAC75,JAC47,JAC54,JAC31,JAC61A,JAC64,JAC103,JAC74C,JAC48,JAC95,JAC25,JAC101,JAC122,JAC17,JAC78,JAC68,JAC72,JAC81,JAC30L,JAC76,JAC58,JAC86,JAC90,JAC74L,JAC6,JAC110,JAC24,JAC35
Isopropyl Palmitate	Low	25.21	JAC99,JAC74C,JAC75,JAC47,JAC78,JAC86,JAC48,JAC103,JAC101,JAC110,JAC45,JAC33,JAC64,JAC6,JAC76,JAC60,JAC31,JAC61A,JAC58,JAC72,JAC102,JAC17,JAC24
(*Z*)-6-Octadecenoic Acid Methyl Ester	Low	25.76	JAC30L,JAC99,JAC90,JAC81,JAC75,JAC78,JAC96,JAC86,JAC48,JAC103,JAC35,JAC88,JAC101,JAC68,JAC110,JAC33,JAC74L,JAC6,JAC76,JAC60,JAC31,JAC61A,JAC122,JAC58,JAC72,JAC25,JAC87,JAC17,JAC24,JAC55

Carbonic Acid, Propargyl 2-Ethylhexyl Ester	High	26.14	JAC33,JAC60,JAC87,JAC102,JAC96,JAC88,JAC55,JAC99,JAC75,JAC47,JAC54,JAC31,JAC61A,JAC45,JAC64,JAC103,JAC74C,JAC48,JAC95,JAC25,JAC101,JAC122,JAC17,JAC78,JAC68,JAC72,JAC81,JAC30L,JAC76,JAC58,JAC86,JAC90,JAC74L,JAC6,JAC110,JAC24,JAC35
1,2-Benzenedicarboxylic Acid Bis(2-Ethylhexyl) Ester	High	26.52	JAC33,JAC60,JAC87,JAC102,JAC96,JAC88,JAC55,JAC99,JAC75,JAC47,JAC54,JAC31,JAC61A,JAC45,JAC64,JAC103,JAC74C,JAC48,JAC95,JAC25,JAC101,JAC122,JAC17,JAC78,JAC68,JAC72,JAC81,JAC30L,JAC76,JAC58,JAC86,JAC90,JAC74L,JAC6,JAC110,JAC24,JAC35
Eicosanebioic Acid, Dimethyl Ester	Low	27.58	JAC30L,JAC99,JAC74C,JAC90,JAC81,JAC75,JAC47,JAC78,JAC96,JAC86,JAC48,JAC95,JAC103,JAC35,JAC88,JAC101,JAC68,JAC110,JAC45,JAC33,JAC64,JAC74L,JAC54,JAC6,JAC76,JAC60,JAC31,JAC61A,JAC122,JAC58,JAC72,JAC25,JAC102,JAC87,JAC17,JAC24,JAC55
Bis(2-Ethylhexyl) Phthalate	High	28.42	JAC60,JAC87,JAC102,JAC96,JAC88,JAC55,JAC99,JAC75,JAC47,JAC54,JAC31,JAC61A,JAC45,JAC64,JAC103,JAC74C,JAC48,JAC95,JAC25,JAC101,JAC122,JAC17,JAC78,JAC68,JAC72,JAC81,JAC30L,JAC76,JAC58,JAC90,JAC74L,JAC6,JAC110,JAC24
1,2-Benzenedicarboxylic Acid Decyl Octyl Ester	High	28.49	JAC60,JAC87,JAC102,JAC96,JAC88,JAC55,JAC99,JAC75,JAC47,JAC54,JAC61A,JAC45,JAC64,JAC74C,JAC48,JAC95,JAC25,JAC122,JAC17,JAC68,JAC72,JAC81,JAC30L,JAC76,JAC90,JAC74L,JAC6,JAC110,JAC24,JAC35
Hexanedioic Acid, Bis(2-Ethylhexyl) Ester	High	28.71	JAC87,JAC88,JAC47,JAC54,JAC31,JAC61A,JAC95,JAC25,JAC101,JAC122,JAC78,JAC81,JAC86,JAC90,JAC74L,JAC6,JAC110,JAC24,JAC35

Succinic Acid, Monochloride 2-Ethylbutyl Ester	High	29.13	JAC33,JAC60,JAC87,JAC102,JAC96,JAC88,JAC55,JAC99,JAC75,JAC47,JAC54,JAC31,JAC61A,JAC45,JAC64,JAC103,JAC74C,JAC48,JAC95,JAC25,JAC101,JAC122,JAC17,JAC78,JAC68,JAC72,JAC81,JAC30L,JAC76,JAC58,JAC86,JAC90,JAC74L,JAC6,JAC110,JAC24,JAC35
Butyramide, 4-Chloro-*N*-Hept-2-Yl-	Low	29.13	JAC99,JAC74C,JAC90,JAC81,JAC75,JAC47,JAC78,JAC96,JAC86,JAC48,JAC95,JAC103,JAC35,JAC88,JAC101,JAC68,JAC110,JAC45,JAC33,JAC64,JAC74L,JAC54,JAC6,JAC76,JAC60,JAC122,JAC58,JAC72,JAC25,JAC102,JAC87,JAC17,JAC24,JAC55
Ethyl 3-Acetoxybutyrate	High	29.51	JAC33,JAC60,JAC87,JAC102,JAC96,JAC88,JAC55,JAC99,JAC75,JAC47,JAC54,JAC31,JAC61A,JAC45,JAC64,JAC103,JAC74C,JAC48,JAC95,JAC25,JAC101,JAC122,JAC17,JAC78,JAC68,JAC72,JAC81,JAC30L,JAC76,JAC58,JAC86,JAC90,JAC74L,JAC6,JAC110,JAC24,JAC35
1,2-Benzenedicarboxylic Acid Hexyl Octyl Ester	High	30.29	JAC60,JAC87,JAC96,JAC88,JAC55,JAC99,JAC75,JAC47,JAC54,JAC31,JAC61A,JAC64,JAC103,JAC74C,JAC48,JAC95,JAC25,JAC101,JAC122,JAC17,JAC68,JAC72,JAC81,JAC30L,JAC76,JAC58,JAC86,JAC90,JAC74L,JAC6,JAC110,JAC35
Dibutyl Itaconate	Low	31.47	JAC30L,JAC90,JAC81,JAC75,JAC96,JAC88,JAC68,JAC110,JAC74L,JAC54,JAC6,JAC122,JAC72,JAC25,JAC87
**Hydrocarbons (31)**			
Hexane, 3-Ethyl-	High	5.2	JAC33,JAC60,JAC87,JAC102,JAC96,JAC88,JAC55,JAC99,JAC75,JAC47,JAC54,JAC31,JAC61A,JAC45,JAC64,JAC103,JAC74C,JAC48,JAC95,JAC25,JAC101,JAC122,JAC17,JAC78,JAC68,JAC72,JAC81,JAC30L

			,JAC76,JAC58,JAC86,JAC90,JAC74L,JAC6,JAC110,JAC24,JAC35
Diisodecyl 4-Cyclohexene-1,2-Dicarboxylate	Low	6.1	JAC30L,JAC99,JAC90,JAC81,JAC75,JAC47,JAC78,JAC96,JAC86,JAC48,JAC95,JAC103,JAC35,JAC88,JAC101,JAC68,JAC110,JAC45,JAC33,JAC64,JAC74L,JAC54,JAC6,JAC76,JAC31,JAC122,JAC58,JAC72,JAC25,JAC102,JAC87,JAC17,JAC24,JAC55
Hexadecane	High	12.1	JAC33,JAC99,JAC75,JAC47,JAC31,JAC45,JAC64,JAC74C,JAC48,JAC25,JAC17,JAC78,JAC68,JAC81,JAC76,JAC58,JAC86,JAC24
1,1,4-Trimethylcyclohexane	High	12.62	JAC87,JAC96,JAC88,JAC55,JAC54,JAC61A,JAC95,JAC25,JAC122,JAC68,JAC76,JAC90,JAC74L,JAC6,JAC110,JAC35
1-Decene	High	12.94	JAC33,JAC60,JAC87,JAC102,JAC96,JAC88,JAC55,JAC99,JAC75,JAC47,JAC54,JAC31,JAC61A,JAC45,JAC64,JAC103,JAC74C,JAC48,JAC95,JAC25,JAC101,JAC122,JAC17,JAC78,JAC68,JAC72,JAC81,JAC30L,JAC76,JAC58,JAC86,JAC74L,JAC6,JAC110,JAC24,JAC35
Cyclohexane, 1,2,3-Trimethyl-	High	13.22	JAC33,JAC60,JAC87,JAC102,JAC96,JAC88,JAC55,JAC99,JAC75,JAC54,JAC31,JAC61A,JAC45,JAC103,JAC48,JAC95,JAC25,JAC122,JAC78,JAC68,JAC72,JAC81,JAC30L,JAC76,JAC58,JAC86,JAC90,JAC74L,JAC6,JAC110,JAC24,JAC35
2,4,4-Trimethyl-1-Hexene	High	13.57	JAC33,JAC60,JAC87,JAC102,JAC96,JAC88,JAC55,JAC99,JAC75,JAC47,JAC54,JAC31,JAC61A,JAC45,JAC64,JAC103,JAC74C,JAC48,JAC95,JAC25,JAC101,JAC122,JAC17,JAC78,JAC68,JAC72,JAC81,JAC30L,JAC76,JAC58,JAC86,JAC90,JAC74L,JAC6,JAC110,JAC35

N-Octadecane	High	14.56	JAC33,JAC87,JAC102,JAC96,JAC88,JAC55,JAC75,JAC54,JAC61A,JAC48,JAC95,JAC25,JAC122,JAC17,JAC78,JAC68,JAC72,JAC81,JAC30L,JAC58,JAC90,JAC74L,JAC6,JAC110,JAC35
Tetracosane	High	16.01	JAC33,JAC87,JAC102,JAC96,JAC88,JAC55,JAC99,JAC75,JAC47,JAC54,JAC31,JAC61A,JAC45,JAC64,JAC103,JAC74C,JAC48,JAC95,JAC25,JAC101,JAC122,JAC17,JAC68,JAC72,JAC81,JAC30L,JAC76,JAC58,JAC86,JAC90,JAC74L,JAC6,JAC110,JAC24,JAC35
1-Hexene, 3,5,5-Trimethyl-	High	16.3	JAC33,JAC60,JAC87,JAC96,JAC88,JAC55,JAC99,JAC75,JAC47,JAC54,JAC31,JAC61A,JAC64,JAC103,JAC48,JAC95,JAC25,JAC101,JAC122,JAC17,JAC78,JAC68,JAC72,JAC81,JAC30L,JAC76,JAC86,JAC90,JAC74L,JAC6,JAC110,JAC35
Pentacosane	High	16.81	JAC33,JAC60,JAC87,JAC102,JAC96,JAC88,JAC55,JAC99,JAC75,JAC47,JAC54,JAC31,JAC61A,JAC45,JAC64,JAC103,JAC48,JAC95,JAC25,JAC101,JAC122,JAC17,JAC78,JAC68,JAC72,JAC81,JAC30L,JAC58,JAC86,JAC90,JAC74L,JAC6,JAC110,JAC24,JAC35
Cyclohexane, Tetradecyl-	High	17.2	JAC33,JAC60,JAC87,JAC102,JAC96,JAC88,JAC55,JAC99,JAC75,JAC47,JAC54,JAC31,JAC61A,JAC64,JAC103,JAC74C,JAC48,JAC95,JAC25,JAC101,JAC122,JAC17,JAC78,JAC68,JAC72,JAC81,JAC30L,JAC76,JAC58,JAC86,JAC90,JAC74L,JAC6,JAC110,JAC24,JAC35
Pentane, 2,2,3,4-Tetramethyl-	High	17.57	JAC60,JAC87,JAC96,JAC88,JAC55,JAC75,JAC47,JAC54,JAC61A,JAC64,JAC103,JAC74C,JAC48,JAC95,JAC25,JAC101,JAC122,JAC17,JAC78,JAC68,JAC72,JAC81,JAC30L,JAC76,JAC86,JAC90,JAC74L,JAC6,JAC110,JAC35

(6Z,9Z)-6,9-Tricosadiene	High	17.86	JAC87,JAC96,JAC88,JAC55,JAC75,JAC54,JAC61A,JAC64,JAC95,JAC25,JAC101,JAC122,JAC68,JAC72,JAC81,JAC30L,JAC90,JAC74L,JAC6,JAC110,JAC35
Cyclohexane, 1,2-Dimethyl-(Cis/Trans)	High	17.91	JAC87,JAC96,JAC88,JAC55,JAC99,JAC75,JAC54,JAC61A,JAC103,JAC48,JAC95,JAC25,JAC122,JAC68,JAC81,JAC74L,JAC6,JAC110
Tridecane	High	18.03	JAC87,JAC96,JAC88,JAC55,JAC75,JAC61A,JAC95,JAC25,JAC122,JAC68,JAC72,JAC81,JAC30L,JAC74L,JAC6,JAC110,JAC35
1,19-Eicosadiene	High	18.76	JAC33,JAC60,JAC87,JAC102,JAC96,JAC88,JAC55,JAC99,JAC75,JAC47,JAC54,JAC31,JAC61A,JAC45,JAC64,JAC103,JAC74C,JAC48,JAC95,JAC25,JAC101,JAC122,JAC17,JAC78,JAC68,JAC72,JAC81,JAC30L,JAC76,JAC58,JAC86,JAC90,JAC74L,JAC6,JAC110,JAC24,JAC35
Octacosane	Low	19.13	JAC30L,JAC90,JAC81,JAC75,JAC96,JAC95,JAC35,JAC88,JAC101,JAC110,JAC74L,JAC54,JAC6,JAC122,JAC72,JAC87,JAC55
1-Heptadecene	High	19.3	JAC33,JAC60,JAC87,JAC102,JAC96,JAC88,JAC55,JAC99,JAC75,JAC47,JAC54,JAC31,JAC61A,JAC45,JAC64,JAC103,JAC74C,JAC48,JAC95,JAC122,JAC17,JAC78,JAC72,JAC81,JAC30L,JAC76,JAC58,JAC86,JAC90,JAC74L,JAC6,JAC110,JAC24,JAC35
Heptylcyclohexane	High	20.14	JAC33,JAC87,JAC102,JAC96,JAC88,JAC55,JAC99,JAC75,JAC47,JAC54,JAC31,JAC61A,JAC45,JAC64,JAC103,JAC74C,JAC48,JAC95,JAC25,JAC101,JAC122,JAC17,JAC78,JAC68,JAC72,JAC81,JAC30L,JAC76,JAC58,JAC86,JAC90,JAC74L,JAC6,JAC110,JAC24,JAC35
1-Eicosene	High	20.61	JAC33,JAC87,JAC96,JAC88,JAC55,JAC99,JAC75,JAC47,JAC54,JAC61A,JAC45,JAC64,JAC103,JAC74C,J

			AC48,JAC95,JAC25,JAC101,JAC122,JAC17,JAC68,JAC72,JAC81,JAC30L,JAC76,JAC58,JAC90,JAC74L,JAC6,JAC110,JAC35
Cyclopentadecane	High	21.76	JAC33,JAC87,JAC96,JAC88,JAC55,JAC99,JAC75,JAC47,JAC54,JAC31,JAC61A,JAC45,JAC103,JAC48,JAC95,JAC25,JAC122,JAC17,JAC68,JAC72,JAC81,JAC30L,JAC76,JAC58,JAC86,JAC90,JAC74L,JAC6,JAC110,JAC24,JAC35
Cyclohexane, Undecyl-	High	22.56	JAC60,JAC87,JAC96,JAC88,JAC55,JAC99,JAC75,JAC47,JAC54,JAC61A,JAC45,JAC64,JAC103,JAC74C,JAC48,JAC95,JAC25,JAC101,JAC122,JAC68,JAC72,JAC30L,JAC76,JAC90,JAC74L,JAC6,JAC110,JAC35
Octadecane	High	22.94	JAC33,JAC60,JAC87,JAC102,JAC96,JAC88,JAC55,JAC99,JAC75,JAC47,JAC54,JAC31,JAC61A,JAC45,JAC64,JAC103,JAC74C,JAC48,JAC95,JAC25,JAC101,JAC122,JAC17,JAC78,JAC68,JAC72,JAC81,JAC30L,JAC76,JAC58,JAC86,JAC90,JAC74L,JAC6,JAC110,JAC24,JAC35
Pentadecane	High	23.02	JAC33,JAC60,JAC87,JAC102,JAC96,JAC88,JAC55,JAC99,JAC75,JAC47,JAC54,JAC31,JAC61A,JAC64,JAC103,JAC74C,JAC48,JAC95,JAC25,JAC101,JAC122,JAC17,JAC78,JAC68,JAC72,JAC81,JAC30L,JAC76,JAC58,JAC90,JAC74L,JAC6,JAC110,JAC35
Octadecane	High	25.01	JAC87,JAC96,JAC88,JAC55,JAC75,JAC54,JAC61A,JAC103,JAC48,JAC101,JAC72,JAC81,JAC30L,JAC76,JAC58,JAC90,JAC74L,JAC6,JAC110,JAC24,JAC35
N-Acetyl-Alanyl-Phenylalanyl-Glycine Methylester	Low	28.17	JAC99,JAC81,JAC75,JAC47,JAC78,JAC96,JAC48,JAC95,JAC103,JAC35,JAC88,JAC110,JAC33,JAC64,JAC6,JAC76,JAC60,JAC31,JAC61A,JAC58,JAC72,JAC25,JAC102,JAC87,JAC17,JAC55

Nonadecane	Low	28.55	JAC30L,JAC99,JAC74C,JAC90,JAC81,JAC75,JAC47,JAC96,JAC86,JAC48,JAC95,JAC103,JAC35,JAC88,JAC101,JAC68,JAC110,JAC45,JAC33,JAC64,JAC74L,JAC54,JAC6,JAC76,JAC60,JAC31,JAC61A,JAC122,JAC58,JAC72,JAC25,JAC102,JAC87,JAC17,JAC24,JAC55
Nonacosane	High	29.22	JAC33,JAC60,JAC87,JAC102,JAC96,JAC88,JAC55,JAC99,JAC75,JAC47,JAC54,JAC31,JAC61A,JAC45,JAC64,JAC103,JAC74C,JAC48,JAC95,JAC25,JAC101,JAC122,JAC17,JAC78,JAC68,JAC72,JAC81,JAC30L,JAC76,JAC58,JAC86,JAC90,JAC74L,JAC6,JAC110,JAC24,JAC35
Oct-3-Enoylamide, *N*-Methyl-*N*-(2-Ethylhexyl)-	Low	29.51	JAC30L,JAC99,JAC90,JAC75,JAC78,JAC96,JAC86,JAC95,JAC103,JAC35,JAC88,JAC101,JAC68,JAC110,JAC33,JAC74L,JAC54,JAC76,JAC31,JAC61A,JAC122,JAC58,JAC72,JAC25,JAC87,JAC17
Trans-2-Nonene	High	30.1	JAC33,JAC60,JAC87,JAC102,JAC96,JAC88,JAC55,JAC99,JAC75,JAC54,JAC31,JAC61A,JAC64,JAC103,JAC74C,JAC48,JAC95,JAC25,JAC101,JAC122,JAC17,JAC78,JAC68,JAC72,JAC81,JAC30L,JAC76,JAC58,JAC86,JAC90,JAC74L,JAC6,JAC110,JAC24,JAC35
**Ketones (19)**			
Pentanone (4-OH-4-Me-2-)	High	5.57	JAC33,JAC60,JAC87,JAC102,JAC96,JAC88,JAC55,JAC99,JAC75,JAC47,JAC54,JAC31,JAC61A,JAC45,JAC64,JAC103,JAC74C,JAC48,JAC95,JAC25,JAC101,JAC122,JAC17,JAC78,JAC68,JAC72,JAC81,JAC30L,JAC76,JAC58,JAC86,JAC90,JAC74L,JAC6,JAC110,JAC24,JAC35
2-Tridecanone	High	5.66	JAC33,JAC60,JAC102,JAC99,JAC75,JAC47,JAC31,JAC45,JAC64,JAC103,JAC74C,JAC48,JAC101,JAC12

			2,JAC17,JAC78,JAC68,JAC30L,JAC76,JAC58,JAC86,JAC6,JAC24
2-Butylcyclopenanone	High	9.67	JAC60,JAC87,JAC102,JAC96,JAC88,JAC55,JAC99,JAC75,JAC47,JAC54,JAC31,JAC61A,JAC45,JAC64,JAC103,JAC48,JAC95,JAC25,JAC122,JAC17,JAC78,JAC68,JAC72,JAC81,JAC30L,JAC76,JAC58,JAC90,JAC74L,JAC6,JAC110,JAC24,JAC35
2-Propyl-5,5-Dimethyl-1,3-Cyclohexanedione	High	10.65	JAC33,JAC87,JAC96,JAC88,JAC55,JAC99,JAC75,JAC47,JAC54,JAC31,JAC61A,JAC64,JAC103,JAC74C,JAC48,JAC95,JAC25,JAC101,JAC122,JAC17,JAC78,JAC68,JAC72,JAC81,JAC30L,JAC76,JAC58,JAC86,JAC90,JAC74L,JAC6,JAC110,JAC24,JAC35
1,2-Cyclopentanedione, 3-Methyl-	High	11.15	JAC33,JAC60,JAC87,JAC96,JAC88,JAC55,JAC99,JAC75,JAC47,JAC54,JAC31,JAC61A,JAC64,JAC103,JAC74C,JAC95,JAC25,JAC101,JAC122,JAC17,JAC78,JAC68,JAC72,JAC81,JAC30L,JAC76,JAC58,JAC90,JAC74L,JAC6,JAC110,JAC24,JAC35
2-Tert-Butylcyclohexanone	High	11.57	JAC33,JAC60,JAC87,JAC102,JAC96,JAC88,JAC55,JAC99,JAC75,JAC47,JAC54,JAC31,JAC61A,JAC45,JAC64,JAC103,JAC74C,JAC48,JAC95,JAC25,JAC101,JAC122,JAC17,JAC78,JAC68,JAC72,JAC81,JAC30L,JAC76,JAC58,JAC86,JAC90,JAC74L,JAC6,JAC110,JAC24,JAC35
Cyclohexanone, 4-(1,1-Dimethylethyl)-	High	12.32	JAC33,JAC60,JAC87,JAC102,JAC96,JAC88,JAC55,JAC99,JAC75,JAC47,JAC54,JAC31,JAC61A,JAC45,JAC64,JAC103,JAC74C,JAC48,JAC95,JAC25,JAC101,JAC122,JAC17,JAC78,JAC68,JAC72,JAC81,JAC30L,JAC76,JAC86,JAC90,JAC74L,JAC6,JAC110,JAC24,JAC35
Ethanone, 2-Chloro-1-(2,4-Dimethylphenyl)-	High	16.16	JAC33,JAC60,JAC87,JAC102,JAC96,JAC88,JAC55,JAC99,JAC75,JAC47,JAC54,JAC31,JAC61A,JAC45,JAC64,JAC103,JAC74C,JAC48,JAC95,JAC25,JAC101

			,JAC122,JAC17,JAC78,JAC68,JAC72,JAC81,JAC30L,JAC76,JAC58,JAC86,JAC90,JAC74L,JAC6,JAC110,JAC24,JAC35
4'-Butoxyacetophenone	High	17.31	JAC33,JAC60,JAC87,JAC102,JAC96,JAC88,JAC55,JAC99,JAC47,JAC54,JAC61A,JAC45,JAC64,JAC103,JAC74C,JAC48,JAC95,JAC25,JAC101,JAC122,JAC17,JAC78,JAC72,JAC81,JAC30L,JAC76,JAC58,JAC86,JAC90,JAC74L,JAC110,JAC35
Cyclododecanone	High	17.94	JAC33,JAC60,JAC102,JAC99,JAC47,JAC31,JAC45,JAC64,JAC103,JAC74C,JAC48,JAC25,JAC101,JAC17,JAC78,JAC72,JAC76,JAC58,JAC86,JAC24
2-(1-Cyclohexenyl)Cyclohexanone	High	18.93	JAC33,JAC60,JAC87,JAC96,JAC88,JAC55,JAC99,JAC75,JAC47,JAC54,JAC31,JAC61A,JAC45,JAC64,JAC103,JAC74C,JAC48,JAC95,JAC25,JAC101,JAC122,JAC17,JAC78,JAC68,JAC72,JAC81,JAC30L,JAC76,JAC58,JAC90,JAC74L,JAC6,JAC110,JAC24,JAC35
Trans-3-Nonen-2-One	High	20.57	JAC33,JAC60,JAC87,JAC102,JAC96,JAC88,JAC55,JAC99,JAC75,JAC47,JAC54,JAC31,JAC61A,JAC45,JAC64,JAC103,JAC74C,JAC48,JAC95,JAC25,JAC101,JAC122,JAC17,JAC78,JAC68,JAC72,JAC81,JAC30L,JAC76,JAC58,JAC86,JAC90,JAC74L,JAC6,JAC110,JAC35
3-Methoxyheptanophenone	High	22.02	JAC33,JAC60,JAC87,JAC102,JAC96,JAC88,JAC55,JAC99,JAC75,JAC47,JAC54,JAC61A,JAC64,JAC103,JAC48,JAC95,JAC25,JAC101,JAC17,JAC78,JAC68,JAC72,JAC81,JAC30L,JAC76,JAC58,JAC86,JAC90,JAC6,JAC110,JAC35
5,5-Dimethyl-1,3-Cyclohexanedione	High	22.02	JAC60,JAC87,JAC96,JAC88,JAC55,JAC75,JAC47,JAC54,JAC31,JAC61A,JAC45,JAC64,JAC103,JAC74C,JAC48,JAC95,JAC25,JAC101,JAC122,JAC17,JAC78,J

			AC68,JAC72,JAC81,JAC30L,JAC76,JAC58,JAC90,JAC74L,JAC6,JAC110,JAC35
Tetradecanophenone	High	23.05	JAC60,JAC87,JAC102,JAC96,JAC88,JAC55,JAC99,JAC75,JAC47,JAC54,JAC31,JAC61A,JAC45,JAC64,JAC103,JAC74C,JAC48,JAC95,JAC25,JAC101,JAC122,JAC78,JAC68,JAC72,JAC81,JAC30L,JAC76,JAC90,JAC74L,JAC6,JAC110,JAC35
Pyrimidine, 6-Oxo-5-Acetyl-4-Hydroxy-1,6-Dihydro-	High	27.4	JAC33,JAC60,JAC87,JAC102,JAC96,JAC88,JAC55,JAC99,JAC75,JAC47,JAC54,JAC45,JAC64,JAC103,JAC74C,JAC48,JAC95,JAC25,JAC101,JAC122,JAC17,JAC78,JAC72,JAC81,JAC30L,JAC58,JAC86,JAC90,JAC74L,JAC6,JAC110,JAC24,JAC35
2-Propanone, 1,1,1-Trichloro-	High	29.4	JAC33,JAC60,JAC102,JAC96,JAC88,JAC99,JAC75,JAC47,JAC31,JAC45,JAC64,JAC48,JAC95,JAC25,JAC122,JAC17,JAC78,JAC68,JAC72,JAC58,JAC86,JAC90,JAC74L,JAC6,JAC110
(4a*S*,5*S*,8a*S*)-8Abeta-Formyl-5Beta-Methyl-5Alpha-(4-Methyl-3-Pentenyl)-3,4,4*A*,5,6,7,8,8A-Octahydronaphthalen-1(2*H*)-One	High	30.87	JAC96,JAC88,JAC55,JAC75,JAC54,JAC61A,JAC25,JAC122,JAC68,JAC72,JAC81,JAC30L,JAC74L,JAC6,JAC35
Hexanal (Si-Contam)_Adms	High	31.2	JAC88,JAC75,JAC61A,JAC95,JAC25,JAC68,JAC81,JAC30L,JAC90,JAC74L,JAC6,JAC35
**Terpenes and Terpenoids (23)**			
2-Octene, 2,6-Dimethyl-	High	7.95	JAC33,JAC60,JAC87,JAC102,JAC96,JAC88,JAC55,JAC99,JAC75,JAC47,JAC54,JAC31,JAC61A,JAC45,JAC64,JAC103,JAC74C,JAC48,JAC95,JAC25,JAC101,JAC122,JAC17,JAC78,JAC68,JAC72,JAC81,JAC30L,JAC76,JAC86,JAC90,JAC74L,JAC6,JAC110,JAC24,JAC35

Perillyl Isobutyrate	High	10.08	JAC33,JAC60,JAC87,JAC102,JAC96,JAC88,JAC55,JAC99,JAC75,JAC47,JAC54,JAC31,JAC61A,JAC45,JAC64,JAC103,JAC74C,JAC48,JAC95,JAC25,JAC101,JAC17,JAC78,JAC68,JAC72,JAC81,JAC30L,JAC76,JAC58,JAC86,JAC90,JAC74L,JAC6,JAC110,JAC24,JAC35
Isopulegol(Equatorial)	High	11.82	JAC33,JAC60,JAC87,JAC102,JAC96,JAC88,JAC55,JAC99,JAC75,JAC47,JAC54,JAC31,JAC61A,JAC45,JAC64,JAC103,JAC74C,JAC48,JAC95,JAC25,JAC101,JAC122,JAC17,JAC78,JAC68,JAC72,JAC81,JAC30L,JAC76,JAC58,JAC86,JAC90,JAC74L,JAC6,JAC110,JAC24,JAC35
1,1-Bis(4,4-Dimethyl-2,6-Dioxocyclohexyl)Ethane	High	12.48	JAC60,JAC87,JAC102,JAC96,JAC88,JAC55,JAC75,JAC47,JAC54,JAC61A,JAC45,JAC95,JAC25,JAC101,JAC122,JAC17,JAC78,JAC68,JAC72,JAC81,JAC30L,JAC58,JAC90,JAC74L,JAC6,JAC110,JAC35
Cyclohexanol, 5-Methyl-2-(1-Methylethyl)-	High	14.07	JAC33,JAC60,JAC87,JAC102,JAC96,JAC88,JAC55,JAC99,JAC75,JAC47,JAC54,JAC31,JAC61A,JAC45,JAC64,JAC103,JAC74C,JAC95,JAC25,JAC101,JAC122,JAC17,JAC78,JAC68,JAC72,JAC81,JAC30L,JAC76,JAC58,JAC86,JAC74L,JAC6,JAC110,JAC24,JAC35
L-Menthol	High	14.16	JAC60,JAC87,JAC102,JAC96,JAC88,JAC55,JAC99,JAC75,JAC47,JAC54,JAC61A,JAC45,JAC64,JAC48,JAC25,JAC122,JAC17,JAC68,JAC72,JAC81,JAC30L,JAC76,JAC86,JAC90,JAC74L,JAC6,JAC110,JAC24
(4-Isopropyl-Trans-6-Methyl-3-Cyclohexenyl)Formaldehyde 2,4-Dinitrophenylhydrazone	Low	15.32	JAC30L,JAC99,JAC74C,JAC90,JAC81,JAC75,JAC47,JAC78,JAC96,JAC86,JAC48,JAC95,JAC103,JAC35,JAC88,JAC101,JAC68,JAC110,JAC45,JAC33,JAC64,JAC74L,JAC54,JAC6,JAC76,JAC60,JAC31,JAC61A,JAC122,JAC58,JAC72,JAC25,JAC102,JAC87,JAC17,JAC24,JAC55

(7S)-(-)-10,10-Di-Me-5-Thia-4-Azatricyclo[5.2.1.0-3,7]Dec-3-Ene-5,5-Dioxide	High	15.71	JAC33,JAC60,JAC87,JAC102,JAC96,JAC88,JAC55,JAC99,JAC75,JAC47,JAC54,JAC31,JAC45,JAC64,JAC103,JAC74C,JAC48,JAC95,JAC25,JAC101,JAC122,JAC17,JAC78,JAC72,JAC81,JAC30L,JAC76,JAC58,JAC86,JAC90,JAC74L,JAC6,JAC110,JAC24,JAC35
Ylangene (Alpha-)	High	17.69	JAC33,JAC60,JAC87,JAC102,JAC96,JAC88,JAC55,JAC99,JAC75,JAC47,JAC54,JAC31,JAC61A,JAC45,JAC64,JAC103,JAC74C,JAC48,JAC95,JAC25,JAC101,JAC122,JAC17,JAC78,JAC68,JAC72,JAC81,JAC30L,JAC76,JAC58,JAC86,JAC90,JAC74L,JAC6,JAC110,JAC24,JAC35
Cubebene (Alpha-)	High	17.77	JAC33,JAC60,JAC87,JAC102,JAC96,JAC88,JAC55,JAC99,JAC75,JAC47,JAC54,JAC31,JAC61A,JAC45,JAC64,JAC103,JAC74C,JAC48,JAC95,JAC25,JAC101,JAC122,JAC17,JAC78,JAC68,JAC72,JAC81,JAC30L,JAC76,JAC58,JAC86,JAC90,JAC74L,JAC6,JAC110,JAC24,JAC35
Acetic Acid, 1,7,7-Trimethyl-Bicyclo[2.2.1]Hept-2-Yl Ester	High	18.23	JAC33,JAC60,JAC102,JAC96,JAC88,JAC55,JAC99,JAC75,JAC47,JAC31,JAC61A,JAC45,JAC103,JAC74C,JAC48,JAC95,JAC101,JAC122,JAC17,JAC78,JAC68,JAC72,JAC30L,JAC76,JAC58,JAC86,JAC90,JAC74L,JAC6,JAC110,JAC24
Aristolochene (4,5-Di-Epi)	High	18.3	JAC33,JAC60,JAC87,JAC102,JAC96,JAC88,JAC55,JAC99,JAC75,JAC47,JAC54,JAC31,JAC61A,JAC45,JAC64,JAC103,JAC48,JAC95,JAC25,JAC101,JAC122,JAC17,JAC78,JAC68,JAC72,JAC81,JAC30L,JAC76,JAC58,JAC86,JAC90,JAC74L,JAC6,JAC110,JAC24,JAC35
Sabinene	High	18.3	JAC87,JAC102,JAC88,JAC75,JAC47,JAC45,JAC64,JAC103,JAC48,JAC95,JAC25,JAC17,JAC72,JAC81,JAC58,JAC86,JAC90,JAC74L,JAC24

Sesquiphellandrene (Beta-)	High	18.51	JAC33,JAC60,JAC87,JAC102,JAC96,JAC88,JAC55,JAC99,JAC75,JAC47,JAC54,JAC31,JAC61A,JAC45,JAC64,JAC103,JAC48,JAC95,JAC25,JAC122,JAC17,JAC78,JAC68,JAC72,JAC81,JAC30L,JAC76,JAC58,JAC86,JAC90,JAC74L,JAC6,JAC110,JAC24,JAC35
(+)-Alpha-Muurolene	High	18.59	JAC33,JAC60,JAC87,JAC102,JAC96,JAC88,JAC55,JAC99,JAC75,JAC47,JAC54,JAC31,JAC61A,JAC45,JAC64,JAC103,JAC74C,JAC48,JAC95,JAC25,JAC101,JAC122,JAC17,JAC78,JAC68,JAC72,JAC81,JAC30L,JAC76,JAC58,JAC86,JAC90,JAC74L,JAC6,JAC110,JAC24,JAC35
7-Isopropenyl-1,4A-Dimethyl-3,4,5,6,7,8-Hexahydro-2-Naphthalenone	High	19.95	JAC96,JAC99,JAC31,JAC61A,JAC64,JAC48,JAC122,JAC68,JAC72,JAC30L,JAC90,JAC74L,JAC6,JAC24,JAC35
4,10-Dimethyl-7-Isopropyl-Bicyclo(4.4.0)Deca-1,4-Diene	High	21.13	JAC33,JAC60,JAC87,JAC102,JAC88,JAC55,JAC99,JAC75,JAC54,JAC64,JAC103,JAC74C,JAC95,JAC25,JAC122,JAC17,JAC78,JAC68,JAC72,JAC81,JAC76,JAC6
Germacrone	High	21.18	JAC33,JAC87,JAC88,JAC55,JAC99,JAC75,JAC47,JAC54,JAC61A,JAC45,JAC64,JAC74C,JAC48,JAC95,JAC25,JAC122,JAC72,JAC81,JAC76,JAC86,JAC90,JAC74L,JAC110,JAC35
Caryophyllene (*Z*-)	High	21.27	JAC33,JAC60,JAC87,JAC102,JAC88,JAC55,JAC99,JAC75,JAC47,JAC54,JAC31,JAC61A,JAC45,JAC64,JAC103,JAC74C,JAC48,JAC95,JAC25,JAC101,JAC122,JAC17,JAC78,JAC68,JAC72,JAC81,JAC30L,JAC76,JAC58,JAC90,JAC74L,JAC6,JAC110,JAC24,JAC35
Eremophilene	High	21.27	JAC33,JAC60,JAC87,JAC102,JAC96,JAC88,JAC55,JAC99,JAC75,JAC47,JAC54,JAC31,JAC61A,JAC45,JAC64,JAC103,JAC74C,JAC48,JAC95,JAC25,JAC101,JAC122,JAC17,JAC78,JAC68,JAC72,JAC81,JAC30L

			,JAC76,JAC58,JAC86,JAC90,JAC74L,JAC6,JAC110,JAC35
Beta-Gurjunene	High	21.55	JAC33,JAC60,JAC87,JAC96,JAC88,JAC55,JAC99,JAC75,JAC47,JAC54,JAC31,JAC61A,JAC45,JAC64,JAC103,JAC74C,JAC48,JAC95,JAC25,JAC101,JAC122,JAC17,JAC78,JAC68,JAC72,JAC81,JAC30L,JAC76,JAC58,JAC86,JAC90,JAC74L,JAC6,JAC35
3-Isopropyl-6,10-Dimethylundecane-2-ol	High	28.79	JAC33,JAC87,JAC102,JAC96,JAC88,JAC55,JAC99,JAC75,JAC47,JAC54,JAC31,JAC61A,JAC45,JAC64,JAC103,JAC48,JAC95,JAC25,JAC122,JAC17,JAC78,JAC68,JAC72,JAC81,JAC30L,JAC76,JAC58,JAC86,JAC90,JAC74L,JAC6,JAC110,JAC24,JAC35
Perfluorononanoic Acid	Low	31.69	JAC81,JAC75,JAC96,JAC95,JAC88,JAC68,JAC74L,JAC54,JAC61A,JAC122,JAC25
**Diverse functional groups (139)**			
Octadecyl Bromide	High	4.33	JAC33,JAC60,JAC87,JAC102,JAC96,JAC88,JAC55,JAC99,JAC75,JAC54,JAC31,JAC61A,JAC64,JAC103,JAC74C,JAC48,JAC95,JAC25,JAC101,JAC122,JAC17,JAC78,JAC68,JAC72,JAC81,JAC30L,JAC76,JAC58,JAC90,JAC74L,JAC6,JAC110,JAC35
Dramamine	High	4.53	JAC33,JAC60,JAC87,JAC102,JAC96,JAC88,JAC99,JAC75,JAC47,JAC54,JAC31,JAC61A,JAC45,JAC64,JAC103,JAC74C,JAC48,JAC95,JAC25,JAC101,JAC122,JAC17,JAC78,JAC68,JAC72,JAC81,JAC30L,JAC76,JAC58,JAC86,JAC90,JAC6,JAC110,JAC24,JAC35
Bis(2-Ethylhexyl) Hydrogen Phosphite	Low	4.7	JAC30L,JAC99,JAC74C,JAC90,JAC81,JAC75,JAC47,JAC78,JAC96,JAC86,JAC48,JAC95,JAC103,JAC35,JAC101,JAC68,JAC110,JAC45,JAC33,JAC64,JAC74L,JAC54,JAC6,JAC76,JAC60,JAC31,JAC61A,JAC122,J

			AC58,JAC72,JAC25,JAC102,JAC87,JAC17,JAC24,JAC55
Behenic Amide	High	4.86	JAC33,JAC60,JAC87,JAC102,JAC96,JAC88,JAC55,JAC54,JAC31,JAC61A,JAC74C,JAC48,JAC95,JAC25,JAC101,JAC122,JAC17,JAC78,JAC68,JAC72,JAC81,JAC30L,JAC58,JAC86,JAC74L,JAC110,JAC24,JAC35
5-Dimethylaminopyrimidine	Low	5.25	JAC30L,JAC99,JAC74C,JAC90,JAC81,JAC78,JAC86,JAC48,JAC103,JAC88,JAC110,JAC33,JAC74L,JAC54,JAC76,JAC61A,JAC122,JAC58,JAC25,JAC102,JAC87,JAC17,JAC24,JAC55
4-Chloro-2-Aminopyrimidine	High	5.33	JAC33,JAC60,JAC102,JAC96,JAC88,JAC75,JAC54,JAC61A,JAC64,JAC103,JAC95,JAC25,JAC17,JAC78,JAC68,JAC72,JAC81,JAC30L,JAC76,JAC86,JAC90,JAC74L,JAC110
Pentadecafluorooctanoic Acid, Pentyl Ester	High	5.33	JAC33,JAC60,JAC87,JAC102,JAC88,JAC55,JAC99,JAC47,JAC54,JAC31,JAC45,JAC64,JAC103,JAC74C,JAC48,JAC25,JAC101,JAC122,JAC17,JAC78,JAC68,JAC72,JAC81,JAC30L,JAC76,JAC58,JAC86,JAC90,JAC6,JAC110,JAC24
Oleic Anhydride	Low	5.54	JAC30L,JAC99,JAC74C,JAC81,JAC75,JAC47,JAC78,JAC96,JAC86,JAC48,JAC95,JAC103,JAC35,JAC88,JAC101,JAC68,JAC45,JAC33,JAC64,JAC54,JAC6,JAC76,JAC60,JAC61A,JAC58,JAC72,JAC25,JAC102,JAC87,JAC17,JAC24,JAC55
Hexenyl 3-Methyl Butanoate (*3Z*-)	High	5.66	JAC33,JAC60,JAC87,JAC102,JAC96,JAC88,JAC55,JAC99,JAC75,JAC54,JAC31,JAC61A,JAC45,JAC64,JAC103,JAC48,JAC95,JAC25,JAC122,JAC78,JAC68,JAC72,JAC81,JAC30L,JAC76,JAC58,JAC86,JAC90,JAC74L,JAC6,JAC110,JAC24,JAC35

Heptanone (2-)	High	5.94	JAC33,JAC60,JAC87,JAC102,JAC96,JAC88,JAC55,JAC75,JAC47,JAC54,JAC31,JAC61A,JAC45,JAC64,JAC103,JAC74C,JAC48,JAC95,JAC25,JAC17,JAC78,JAC68,JAC72,JAC81,JAC30L,JAC58,JAC86,JAC90,JAC74L,JAC6,JAC110,JAC24,JAC35
3-Methyl-P-Anisaldehyde	High	6.04	JAC33,JAC60,JAC87,JAC102,JAC96,JAC88,JAC55,JAC99,JAC75,JAC47,JAC54,JAC31,JAC61A,JAC45,JAC64,JAC103,JAC74C,JAC48,JAC95,JAC25,JAC101,JAC122,JAC17,JAC78,JAC68,JAC72,JAC81,JAC30L,JAC76,JAC58,JAC86,JAC90,JAC74L,JAC6,JAC110,JAC24,JAC35
(*S*)-3-Hydroxyisobutyric Acid	Low	6.23	JAC30L,JAC99,JAC74C,JAC90,JAC81,JAC75,JAC47,JAC78,JAC96,JAC86,JAC48,JAC95,JAC103,JAC35,JAC88,JAC101,JAC68,JAC110,JAC45,JAC33,JAC64,JAC74L,JAC54,JAC6,JAC76,JAC60,JAC31,JAC61A,JAC122,JAC58,JAC72,JAC25,JAC102,JAC87,JAC17,JAC24,JAC55
(+)-*N*-Benzyl-.Alpha.-Phenethylamine	High	6.25	JAC33,JAC60,JAC87,JAC102,JAC96,JAC88,JAC55,JAC99,JAC75,JAC47,JAC54,JAC31,JAC61A,JAC45,JAC64,JAC103,JAC74C,JAC48,JAC95,JAC25,JAC101,JAC122,JAC17,JAC78,JAC68,JAC72,JAC81,JAC30L,JAC76,JAC58,JAC86,JAC90,JAC74L,JAC6,JAC110,JAC24,JAC35
4-Piperidinemethanamine	High	6.31	JAC33,JAC87,JAC102,JAC96,JAC88,JAC55,JAC99,JAC75,JAC47,JAC54,JAC31,JAC45,JAC64,JAC103,JAC74C,JAC48,JAC95,JAC25,JAC101,JAC17,JAC78,JAC68,JAC81,JAC30L,JAC76,JAC58,JAC86,JAC90,JAC74L,JAC6,JAC110,JAC24,JAC35
Acetamide, 2-(Phenylthio)-*N*-Butyl-*N*-Ethyl-	Low	6.59	JAC30L,JAC99,JAC74C,JAC90,JAC81,JAC75,JAC47,JAC78,JAC96,JAC86,JAC48,JAC95,JAC103,JAC35,JAC88,JAC101,JAC68,JAC110,JAC45,JAC33,JAC64,JAC74L,JAC54,JAC6,JAC76,JAC60,JAC31,JAC61A,J

			AC122,JAC58,JAC72,JAC25,JAC102,JAC87,JAC17,JAC24,JAC55
1,1,3-Trimethyl-1-Silacyclo-3-Pentene	Low	6.85	JAC30L,JAC99,JAC74C,JAC90,JAC81,JAC75,JAC47,JAC78,JAC96,JAC86,JAC48,JAC95,JAC103,JAC35,JAC88,JAC101,JAC68,JAC110,JAC45,JAC33,JAC64,JAC74L,JAC54,JAC6,JAC76,JAC60,JAC31,JAC61A,JAC122,JAC58,JAC72,JAC25,JAC102,JAC87,JAC17,JAC24,JAC55
Pentadecafluorooctanoic Acid, Isobutyl Ester	High	7.11	JAC60,JAC87,JAC102,JAC96,JAC88,JAC55,JAC99,JAC75,JAC47,JAC31,JAC61A,JAC45,JAC64,JAC103,JAC74C,JAC95,JAC25,JAC101,JAC122,JAC17,JAC78,JAC72,JAC81,JAC30L,JAC76,JAC58,JAC90,JAC6,JAC110,JAC35
6-Iodo-2-Picolin-5-ol	High	7.25	JAC87,JAC96,JAC88,JAC55,JAC75,JAC54,JAC31,JAC61A,JAC103,JAC48,JAC95,JAC25,JAC122,JAC68,JAC72,JAC81,JAC30L,JAC86,JAC90,JAC74L,JAC6,JAC110,JAC24,JAC35
Pyrazine, Methoxy-, 4-Oxide	Low	7.27	JAC30L,JAC99,JAC74C,JAC81,JAC75,JAC47,JAC78,JAC48,JAC95,JAC103,JAC35,JAC88,JAC101,JAC68,JAC110,JAC33,JAC64,JAC74L,JAC6,JAC76,JAC60,JAC31,JAC58,JAC72,JAC25,JAC102,JAC17,JAC24,JAC55
2-(4-Hydroxyphenyl)Ethanoic Acid	High	7.32	JAC33,JAC60,JAC102,JAC96,JAC99,JAC47,JAC31,JAC45,JAC64,JAC74C,JAC48,JAC101,JAC17,JAC78,JAC76,JAC58,JAC86,JAC24
Pentadecafluorooctanoic Acid, 4-Methyl-2-Pentyl Ester	High	7.4	JAC33,JAC60,JAC102,JAC88,JAC55,JAC99,JAC75,JAC54,JAC45,JAC64,JAC74C,JAC95,JAC25,JAC101,JAC122,JAC17,JAC78,JAC68,JAC72,JAC81,JAC74L,JAC110,JAC24,JAC35

Acetamide, 2-(Thiophen-2-Yl)-*N*-Methyl-*N*-(3-Methylbutyl)-	Low	7.46	JAC30L,JAC74C,JAC90,JAC81,JAC75,JAC47,JAC96,JAC86,JAC95,JAC88,JAC110,JAC33,JAC74L,JAC54,JAC76,JAC60,JAC31,JAC122,JAC58,JAC72,JAC25,JAC102,JAC17,JAC24,JAC55
Pentadecafluorooctanoic Acid, 3-Methylbut-2-En-1-Yl Ester	Low	7.46	JAC30L,JAC99,JAC74C,JAC90,JAC81,JAC75,JAC47,JAC78,JAC96,JAC48,JAC103,JAC45,JAC64,JAC74L,JAC76,JAC60,JAC122,JAC58,JAC72,JAC102,JAC17,JAC24,JAC55
Pentadecafluorooctanoic Acid, Undec-2-En-1-Yl Ester	High	7.61	JAC33,JAC60,JAC87,JAC102,JAC96,JAC88,JAC55,JAC99,JAC75,JAC47,JAC54,JAC31,JAC61A,JAC45,JAC64,JAC103,JAC74C,JAC48,JAC95,JAC25,JAC101,JAC122,JAC17,JAC78,JAC68,JAC72,JAC81,JAC30L,JAC76,JAC58,JAC90,JAC74L,JAC6,JAC110,JAC24,JAC35
Pentadecafluorooctanoic Acid, Dodecyl Ester	High	7.79	JAC33,JAC60,JAC102,JAC96,JAC88,JAC55,JAC99,JAC75,JAC47,JAC54,JAC31,JAC45,JAC64,JAC103,JAC74C,JAC48,JAC25,JAC101,JAC122,JAC17,JAC78,JAC68,JAC72,JAC81,JAC30L,JAC76,JAC58,JAC86,JAC90,JAC74L,JAC6,JAC110,JAC24,JAC35
Pentadecafluorooctanoic Acid, Hexyl Ester	High	7.79	JAC33,JAC87,JAC102,JAC88,JAC75,JAC47,JAC31,JAC61A,JAC45,JAC64,JAC103,JAC74C,JAC48,JAC95,JAC25,JAC17,JAC78,JAC72,JAC30L,JAC76,JAC58,JAC90,JAC24,JAC35
Levoglucosan	High	7.95	JAC33,JAC60,JAC87,JAC102,JAC96,JAC88,JAC55,JAC99,JAC54,JAC31,JAC61A,JAC45,JAC64,JAC103,JAC74C,JAC48,JAC95,JAC25,JAC101,JAC122,JAC17,JAC78,JAC68,JAC81,JAC30L,JAC76,JAC58,JAC86,JAC90,JAC74L,JAC6,JAC110,JAC24,JAC35
Dotriacontyl Isobutyl Ether	High	8.36	JAC60,JAC87,JAC102,JAC96,JAC88,JAC55,JAC99,JAC75,JAC47,JAC54,JAC31,JAC61A,JAC64,JAC103,JAC74C,JAC48,JAC95,JAC101,JAC122,JAC78,JAC72

			,JAC81,JAC30L,JAC76,JAC58,JAC86,JAC90,JAC74L,JAC110,JAC24,JAC35
1,2-Benzenedicarboxaldehyde	High	8.46	JAC33,JAC60,JAC87,JAC102,JAC96,JAC88,JAC55,JAC99,JAC75,JAC47,JAC54,JAC31,JAC61A,JAC45,JAC64,JAC103,JAC74C,JAC48,JAC95,JAC25,JAC101,JAC122,JAC17,JAC78,JAC68,JAC72,JAC81,JAC30L,JAC76,JAC58,JAC86,JAC90,JAC74L,JAC6,JAC110,JAC24,JAC35
(2*S*)-*N*-Tert-Butoxycarbonyl-2-(1,4-Dimethanesulfonyloxybutyl)Pyrrolidine	Low	8.69	JAC30L,JAC99,JAC74C,JAC90,JAC81,JAC75,JAC47,JAC78,JAC96,JAC86,JAC48,JAC95,JAC103,JAC35,JAC88,JAC101,JAC110,JAC45,JAC33,JAC64,JAC74L,JAC54,JAC76,JAC60,JAC31,JAC61A,JAC122,JAC58,JAC72,JAC25,JAC87,JAC17,JAC24,JAC55
(*Z*,*Z*)-12,15-Octadecadienoic Acid Methyl Ester	High	8.74	JAC33,JAC60,JAC87,JAC102,JAC96,JAC88,JAC55,JAC99,JAC75,JAC47,JAC54,JAC31,JAC61A,JAC45,JAC64,JAC103,JAC74C,JAC48,JAC95,JAC25,JAC101,JAC122,JAC17,JAC78,JAC68,JAC72,JAC81,JAC30L,JAC76,JAC58,JAC86,JAC90,JAC74L,JAC6,JAC110,JAC24,JAC35
Arachidonic Acid	High	8.77	JAC87,JAC102,JAC96,JAC88,JAC55,JAC99,JAC75,JAC47,JAC54,JAC31,JAC45,JAC64,JAC103,JAC74C,JAC48,JAC95,JAC25,JAC122,JAC17,JAC78,JAC68,JAC72,JAC81,JAC30L,JAC76,JAC58,JAC90,JAC74L,JAC6,JAC110,JAC24
Perfluoro(Methylcyclohexane)	High	8.77	JAC33,JAC60,JAC87,JAC102,JAC96,JAC88,JAC55,JAC99,JAC75,JAC47,JAC54,JAC31,JAC61A,JAC64,JAC103,JAC74C,JAC48,JAC95,JAC25,JAC101,JAC122,JAC17,JAC78,JAC68,JAC72,JAC81,JAC30L,JAC76,JAC58,JAC90,JAC74L,JAC6,JAC110,JAC24,JAC35
Acetaldehyde Hexyl Isobutyl Acetal	High	8.82	JAC33,JAC60,JAC87,JAC102,JAC88,JAC55,JAC99,JAC75,JAC54,JAC31,JAC61A,JAC45,JAC103,JAC48,JAC95,JAC25,JAC122,JAC17,JAC78,JAC72,JAC81,JA

			C30L,JAC76,JAC58,JAC86,JAC90,JAC74L,JAC6,JAC110,JAC35
(5Alpha)-7,8-Didehydro-4,5-Epoxy-3-Methoxy-17-Methylmorphinan-14-ol	Low	9.21	JAC30L,JAC99,JAC74C,JAC90,JAC81,JAC75,JAC47,JAC78,JAC96,JAC86,JAC48,JAC95,JAC103,JAC35,JAC88,JAC68,JAC110,JAC45,JAC33,JAC74L,JAC54,JAC6,JAC76,JAC60,JAC61A,JAC122,JAC72,JAC25,JAC87,JAC17,JAC55
3,3-Diethylglutaric Acid	High	9.39	JAC33,JAC60,JAC87,JAC102,JAC96,JAC88,JAC55,JAC99,JAC75,JAC47,JAC54,JAC31,JAC61A,JAC45,JAC64,JAC103,JAC48,JAC95,JAC25,JAC101,JAC122,JAC17,JAC78,JAC68,JAC72,JAC81,JAC30L,JAC76,JAC58,JAC86,JAC90,JAC74L,JAC6,JAC110,JAC24,JAC35
Pentadecafluorooctanoic Acid, Propyl Ester	High	9.5	JAC33,JAC87,JAC102,JAC96,JAC88,JAC55,JAC99,JAC75,JAC47,JAC54,JAC61A,JAC45,JAC103,JAC74C,JAC48,JAC25,JAC78,JAC72,JAC81,JAC30L,JAC76,JAC58,JAC86,JAC90,JAC74L,JAC6,JAC110,JAC24,JAC35
Tremorine	Low	9.5	JAC30L,JAC90,JAC81,JAC75,JAC47,JAC78,JAC96,JAC86,JAC95,JAC35,JAC88,JAC110,JAC45,JAC33,JAC64,JAC74L,JAC54,JAC60,JAC31,JAC61A,JAC122,JAC58,JAC72,JAC25,JAC102,JAC87,JAC17,JAC24,JAC55
Cresol (Ortho-)	High	9.61	JAC33,JAC60,JAC87,JAC102,JAC96,JAC55,JAC99,JAC75,JAC47,JAC54,JAC31,JAC45,JAC64,JAC103,JAC74C,JAC48,JAC95,JAC25,JAC101,JAC122,JAC17,JAC78,JAC68,JAC81,JAC76,JAC58,JAC86,JAC74L,JAC6,JAC24
3,6-Dimethyl-5-Oxo-1,2,3,5-Tetrahydroimidazo[1,2-A]Pyrimidine	Low	9.86	JAC30L,JAC99,JAC74C,JAC90,JAC81,JAC75,JAC47,JAC78,JAC96,JAC86,JAC48,JAC95,JAC103,JAC35,JAC88,JAC101,JAC68,JAC110,JAC45,JAC33,JAC64,JAC74L,JAC54,JAC6,JAC76,JAC60,JAC31,JAC61A,J

			AC122,JAC58,JAC72,JAC25,JAC102,JAC87,JAC17,JAC24,JAC55
4-Amino-2,2,5,5-Tetramethyl-3-Imidazoline-1-Oxyl	High	10.03	JAC96,JAC88,JAC55,JAC99,JAC47,JAC54,JAC31,JAC61A,JAC64,JAC103,JAC74C,JAC95,JAC101,JAC17,JAC68,JAC76,JAC74L,JAC110,JAC24,JAC35
Benzenemethanol, 4-(1,1-Dimethylethyl)-	High	10.08	JAC33,JAC60,JAC87,JAC102,JAC96,JAC88,JAC55,JAC99,JAC75,JAC54,JAC31,JAC61A,JAC45,JAC103,JAC74C,JAC95,JAC101,JAC122,JAC17,JAC78,JAC68,JAC72,JAC81,JAC30L,JAC76,JAC58,JAC86,JAC90,JAC74L,JAC6,JAC110,JAC24,JAC35
N-((Methylphenylamino)Methyl)Benzamide	High	10.29	JAC33,JAC60,JAC87,JAC102,JAC96,JAC88,JAC55,JAC99,JAC75,JAC47,JAC54,JAC31,JAC61A,JAC45,JAC64,JAC103,JAC74C,JAC48,JAC95,JAC25,JAC101,JAC122,JAC17,JAC78,JAC68,JAC72,JAC81,JAC30L,JAC76,JAC58,JAC86,JAC90,JAC74L,JAC6,JAC110,JAC24,JAC35
Ethyl 3-Methyl-5-Methylpyrrole-2-Carboxylate	High	10.55	JAC33,JAC60,JAC87,JAC96,JAC88,JAC55,JAC99,JAC75,JAC54,JAC31,JAC61A,JAC45,JAC64,JAC74C,JAC48,JAC95,JAC25,JAC101,JAC122,JAC68,JAC72,JAC81,JAC76,JAC58,JAC86,JAC90,JAC74L,JAC110,JAC24,JAC35
2-Butanone, (1-Methyl-2-Propenyl)Hydrazone	Low	10.71	JAC30L,JAC99,JAC74C,JAC90,JAC81,JAC75,JAC47,JAC78,JAC96,JAC86,JAC48,JAC95,JAC103,JAC35,JAC88,JAC101,JAC68,JAC110,JAC45,JAC33,JAC64,JAC74L,JAC54,JAC6,JAC76,JAC60,JAC31,JAC61A,JAC122,JAC58,JAC72,JAC25,JAC102,JAC87,JAC17,JAC24,JAC55
Suberoyl Chloride	High	10.77	JAC33,JAC60,JAC87,JAC102,JAC96,JAC88,JAC55,JAC99,JAC75,JAC54,JAC31,JAC61A,JAC64,JAC95,JAC25,JAC101,JAC78,JAC68,JAC72,JAC81,JAC30L,JAC76,JAC86,JAC90,JAC74L,JAC110,JAC24,JAC35

Pentadecafluorooctanoic Acid, Tridec-2-Yn-1-Yl Ester	Low	10.97	JAC30L,JAC99,JAC81,JAC75,JAC78,JAC95,JAC103,JAC35,JAC101,JAC68,JAC45,JAC33,JAC64,JAC54,JAC6,JAC76,JAC60,JAC122,JAC58,JAC72,JAC25,JAC17,JAC55
Oleic Acid	High	11.05	JAC33,JAC60,JAC87,JAC102,JAC96,JAC88,JAC55,JAC99,JAC75,JAC47,JAC54,JAC31,JAC61A,JAC45,JAC64,JAC103,JAC74C,JAC48,JAC95,JAC25,JAC101,JAC122,JAC17,JAC78,JAC68,JAC72,JAC81,JAC30L,JAC76,JAC58,JAC86,JAC90,JAC74L,JAC6,JAC110,JAC24,JAC35
1,3-Dibenzoyl-4-Oxo-2-Thioxoimidazolidine	High	11.11	JAC33,JAC60,JAC87,JAC102,JAC96,JAC88,JAC55,JAC99,JAC75,JAC47,JAC54,JAC31,JAC61A,JAC45,JAC64,JAC103,JAC74C,JAC48,JAC95,JAC25,JAC101,JAC122,JAC17,JAC78,JAC68,JAC72,JAC81,JAC30L,JAC76,JAC58,JAC86,JAC90,JAC74L,JAC6,JAC110,JAC24
N-.Alpha.-Benzoyl-L-Arginine	High	11.11	JAC60,JAC87,JAC96,JAC88,JAC55,JAC99,JAC75,JAC47,JAC54,JAC31,JAC61A,JAC45,JAC64,JAC103,JAC74C,JAC48,JAC95,JAC25,JAC101,JAC122,JAC78,JAC68,JAC72,JAC81,JAC30L,JAC76,JAC58,JAC86,JAC90,JAC74L,JAC6,JAC110,JAC24,JAC35
1H,1H,2H-Perfluoro-1-Octene	High	11.15	JAC33,JAC60,JAC87,JAC102,JAC88,JAC55,JAC99,JAC75,JAC47,JAC54,JAC31,JAC45,JAC64,JAC103,JAC74C,JAC48,JAC95,JAC25,JAC101,JAC17,JAC78,JAC68,JAC81,JAC76,JAC58,JAC86,JAC6,JAC110,JAC24
Decanedioyl Dichloride	High	11.78	JAC102,JAC47,JAC45,JAC64,JAC103,JAC74C,JAC48,JAC25,JAC101,JAC17,JAC78,JAC68,JAC81,JAC76,JAC58,JAC86,JAC90,JAC6
Benzeneacetic Acid, .Alpha.-Oxo-, Methyl Ester	High	11.91	JAC33,JAC87,JAC96,JAC88,JAC55,JAC99,JAC75,JAC54,JAC61A,JAC45,JAC64,JAC74C,JAC48,JAC95,J

			AC25,JAC101,JAC122,JAC78,JAC68,JAC72,JAC81,JAC30L,JAC76,JAC90,JAC74L,JAC6,JAC110,JAC35
Benzoic Acid Mono-Tms	High	11.96	JAC33,JAC60,JAC87,JAC102,JAC96,JAC88,JAC55,JAC99,JAC75,JAC47,JAC54,JAC31,JAC61A,JAC45,JAC64,JAC103,JAC74C,JAC48,JAC95,JAC25,JAC101,JAC122,JAC17,JAC78,JAC68,JAC72,JAC81,JAC30L,JAC76,JAC58,JAC86,JAC90,JAC74L,JAC6,JAC110,JAC24,JAC35
Carbonochloridic Acid, Heptyl Ester	High	12.19	JAC33,JAC60,JAC87,JAC102,JAC96,JAC88,JAC55,JAC99,JAC75,JAC47,JAC54,JAC31,JAC61A,JAC45,JAC64,JAC103,JAC74C,JAC48,JAC95,JAC25,JAC101,JAC122,JAC17,JAC78,JAC68,JAC72,JAC81,JAC30L,JAC76,JAC58,JAC86,JAC90,JAC74L,JAC6,JAC110,JAC24,JAC35
5-Trimethylsilyl-1,2,3,4-Tetrahydropyrimidine-2,4-Dione	Low	12.72	JAC30L,JAC74C,JAC90,JAC81,JAC75,JAC47,JAC78,JAC96,JAC48,JAC95,JAC103,JAC35,JAC88,JAC101,JAC68,JAC110,JAC45,JAC33,JAC74L,JAC54,JAC6,JAC76,JAC60,JAC31,JAC61A,JAC122,JAC58,JAC72,JAC25,JAC102,JAC87,JAC17,JAC24,JAC55
Pentadecafluorooctanoic Acid, Octyl Ester	Low	12.72	JAC99,JAC74C,JAC47,JAC78,JAC96,JAC86,JAC48,JAC103,JAC101,JAC68,JAC45,JAC33,JAC76,JAC60,JAC31,JAC58,JAC102,JAC17,JAC24
Benzenamine, 2,4-Dimethoxy-	High	13.66	JAC33,JAC60,JAC87,JAC96,JAC99,JAC47,JAC31,JAC45,JAC64,JAC103,JAC74C,JAC48,JAC95,JAC25,JAC101,JAC122,JAC17,JAC78,JAC68,JAC81,JAC30L,JAC76,JAC58,JAC86,JAC110,JAC35
2,5-Dimethylbenzophenone	High	13.72	JAC33,JAC60,JAC87,JAC96,JAC88,JAC55,JAC99,JAC75,JAC47,JAC54,JAC31,JAC61A,JAC103,JAC74C,JAC48,JAC95,JAC101,JAC122,JAC68,JAC72,JAC81,JAC30L,JAC76,JAC86,JAC90,JAC74L,JAC6,JAC110

O-Decylhydroxylamine	High	13.9	JAC33,JAC60,JAC87,JAC102,JAC96,JAC88,JAC55,JAC99,JAC75,JAC54,JAC31,JAC61A,JAC103,JAC48,JAC95,JAC25,JAC122,JAC17,JAC78,JAC68,JAC72,JAC81,JAC30L,JAC76,JAC58,JAC86,JAC90,JAC74L,JAC6,JAC110,JAC35
Para-Bromotoluene	High	13.97	JAC33,JAC60,JAC87,JAC102,JAC96,JAC88,JAC55,JAC99,JAC75,JAC47,JAC54,JAC31,JAC61A,JAC45,JAC64,JAC103,JAC74C,JAC48,JAC95,JAC25,JAC101,JAC122,JAC17,JAC78,JAC68,JAC72,JAC81,JAC30L,JAC76,JAC58,JAC86,JAC90,JAC74L,JAC6,JAC110,JAC24,JAC35
2'-Ethylpropiophenone	High	14.16	JAC33,JAC60,JAC87,JAC102,JAC96,JAC88,JAC55,JAC99,JAC75,JAC47,JAC54,JAC31,JAC45,JAC64,JAC103,JAC74C,JAC48,JAC25,JAC101,JAC17,JAC78,JAC72,JAC81,JAC30L,JAC76,JAC58,JAC86,JAC6,JAC110,JAC24
Diazene, Bis[4-(Hexyloxy)Phenyl]-, 1-Oxide	High	14.45	JAC33,JAC60,JAC87,JAC102,JAC96,JAC88,JAC55,JAC99,JAC75,JAC54,JAC31,JAC61A,JAC45,JAC64,JAC103,JAC74C,JAC48,JAC95,JAC25,JAC101,JAC122,JAC17,JAC78,JAC68,JAC72,JAC81,JAC30L,JAC76,JAC58,JAC86,JAC90,JAC74L,JAC6,JAC110,JAC35
Pentadecafluorooctanoic Acid, 2-Ethylhexyl Ester	High	14.65	JAC33,JAC60,JAC87,JAC96,JAC88,JAC55,JAC99,JAC75,JAC54,JAC61A,JAC45,JAC103,JAC74C,JAC48,JAC95,JAC25,JAC101,JAC122,JAC17,JAC78,JAC68,JAC72,JAC81,JAC30L,JAC76,JAC58,JAC86,JAC90,JAC74L,JAC6,JAC110,JAC35
Pentadecafluorooctanoic Acid, Heptyl Ester	High	14.84	JAC33,JAC60,JAC87,JAC102,JAC96,JAC88,JAC55,JAC99,JAC75,JAC47,JAC54,JAC31,JAC61A,JAC64,JAC103,JAC74C,JAC48,JAC95,JAC25,JAC101,JAC122,JAC17,JAC78,JAC68,JAC72,JAC81,JAC30L,JAC76,JAC58,JAC86,JAC90,JAC74L,JAC6,JAC110,JAC24,JAC35

Beta-Cyclocitral	High	15.24	JAC33,JAC87,JAC96,JAC88,JAC55,JAC99,JAC75,JAC47,JAC54,JAC31,JAC61A,JAC45,JAC64,JAC103,JAC74C,JAC48,JAC95,JAC25,JAC101,JAC122,JAC68,JAC72,JAC81,JAC30L,JAC76,JAC86,JAC90,JAC74L,JAC6,JAC110,JAC35
1-Phenyl-1-Nonyne	High	15.5	JAC33,JAC60,JAC87,JAC96,JAC88,JAC55,JAC99,JAC47,JAC54,JAC31,JAC61A,JAC45,JAC64,JAC103,JAC74C,JAC48,JAC95,JAC25,JAC101,JAC122,JAC17,JAC78,JAC68,JAC72,JAC81,JAC30L,JAC76,JAC58,JAC86,JAC90,JAC74L,JAC6,JAC110,JAC24,JAC35
Benzene, 1,4-Bis(1-Methylethenyl)-	Low	15.5	JAC99,JAC74C,JAC81,JAC75,JAC47,JAC78,JAC96,JAC86,JAC48,JAC95,JAC103,JAC68,JAC45,JAC33,JAC64,JAC74L,JAC54,JAC6,JAC76,JAC60,JAC31,JAC122,JAC72,JAC102,JAC24
Allylbenzene	High	15.75	JAC33,JAC60,JAC87,JAC102,JAC88,JAC55,JAC99,JAC75,JAC47,JAC54,JAC31,JAC61A,JAC45,JAC64,JAC103,JAC74C,JAC48,JAC95,JAC25,JAC101,JAC122,JAC17,JAC78,JAC68,JAC72,JAC30L,JAC76,JAC58,JAC86,JAC6,JAC24,JAC35
Benzoic Acid, 2-Acetylhydrazide	High	15.75	JAC60,JAC87,JAC102,JAC96,JAC88,JAC55,JAC99,JAC75,JAC54,JAC61A,JAC45,JAC64,JAC74C,JAC48,JAC95,JAC25,JAC101,JAC122,JAC78,JAC68,JAC72,JAC30L,JAC76,JAC90,JAC74L,JAC6,JAC110,JAC35
Ethyl Acetophenone (P-)	High	15.75	JAC87,JAC96,JAC88,JAC55,JAC54,JAC61A,JAC103,JAC74C,JAC95,JAC25,JAC122,JAC78,JAC68,JAC81,JAC90,JAC74L,JAC6,JAC110,JAC35
Benzenamine, 4-Bromo-3-Methyl-	Low	15.84	JAC30L,JAC99,JAC74C,JAC90,JAC81,JAC75,JAC78,JAC96,JAC86,JAC48,JAC95,JAC103,JAC35,JAC88,JAC101,JAC68,JAC110,JAC33,JAC74L,JAC54,JAC6,JAC76,JAC60,JAC31,JAC61A,JAC122,JAC58,JAC72,JAC25,JAC102,JAC87,JAC17,JAC24,JAC55

Behenyl Chloride	High	16.45	JAC33,JAC60,JAC87,JAC102,JAC96,JAC88,JAC55,JAC99,JAC75,JAC47,JAC54,JAC31,JAC61A,JAC45,JAC64,JAC103,JAC74C,JAC48,JAC95,JAC25,JAC101,JAC122,JAC17,JAC78,JAC68,JAC72,JAC81,JAC30L,JAC76,JAC58,JAC86,JAC90,JAC74L,JAC6,JAC110,JAC24,JAC35
Nonyl Tetradecyl Ether	High	16.58	JAC33,JAC60,JAC87,JAC102,JAC96,JAC88,JAC55,JAC75,JAC54,JAC31,JAC61A,JAC45,JAC64,JAC48,JAC95,JAC25,JAC122,JAC17,JAC78,JAC68,JAC81,JAC30L,JAC58,JAC90,JAC74L,JAC6,JAC110,JAC24,JAC35
1-Bromo-8-Tetrahydropyranyloxyoctane	High	16.81	JAC33,JAC60,JAC87,JAC102,JAC96,JAC88,JAC55,JAC99,JAC75,JAC54,JAC31,JAC61A,JAC45,JAC64,JAC103,JAC48,JAC95,JAC25,JAC101,JAC122,JAC17,JAC78,JAC68,JAC72,JAC81,JAC30L,JAC76,JAC58,JAC86,JAC90,JAC74L,JAC6,JAC110,JAC24,JAC35
Benzene, 1,4-Bis(1,1-Dimethylethyl)-	Low	16.97	JAC30L,JAC99,JAC74C,JAC90,JAC81,JAC75,JAC47,JAC78,JAC96,JAC86,JAC48,JAC95,JAC103,JAC35,JAC88,JAC101,JAC68,JAC110,JAC45,JAC33,JAC64,JAC74L,JAC54,JAC6,JAC76,JAC60,JAC31,JAC61A,JAC122,JAC58,JAC72,JAC25,JAC102,JAC87,JAC17,JAC24,JAC55
4-Benzylaminoindole	High	17.04	JAC33,JAC60,JAC87,JAC102,JAC96,JAC88,JAC55,JAC99,JAC75,JAC47,JAC54,JAC31,JAC61A,JAC45,JAC64,JAC103,JAC74C,JAC48,JAC95,JAC25,JAC101,JAC122,JAC17,JAC78,JAC68,JAC72,JAC81,JAC30L,JAC76,JAC58,JAC86,JAC90,JAC74L,JAC6,JAC110,JAC24,JAC35
Methyl 6-Methyl-3-Pyridyl Ketone 4-Cyclohexylthiosemicarbazone	Low	17.26	JAC30L,JAC99,JAC74C,JAC90,JAC81,JAC75,JAC78,JAC96,JAC86,JAC95,JAC35,JAC88,JAC101,JAC68,JAC110,JAC45,JAC33,JAC64,JAC54,JAC6,JAC60,JA

			C31,JAC61A,JAC122,JAC58,JAC72,JAC25,JAC102,JAC87,JAC17,JAC24,JAC55
Nonalactone (Gamma-)	High	17.41	JAC33,JAC60,JAC87,JAC102,JAC96,JAC88,JAC55,JAC99,JAC75,JAC54,JAC31,JAC61A,JAC64,JAC103,JAC48,JAC95,JAC25,JAC122,JAC17,JAC78,JAC68,JAC72,JAC81,JAC30L,JAC76,JAC58,JAC86,JAC90,JAC74L,JAC6,JAC110,JAC24,JAC35
Guanidine, N,N'-Bis(2-Methylphenyl)-	High	17.86	JAC33,JAC60,JAC87,JAC102,JAC96,JAC88,JAC55,JAC99,JAC75,JAC47,JAC54,JAC31,JAC61A,JAC45,JAC64,JAC103,JAC74C,JAC48,JAC95,JAC25,JAC122,JAC17,JAC78,JAC68,JAC72,JAC81,JAC30L,JAC76,JAC58,JAC86,JAC90,JAC74L,JAC6,JAC110,JAC35
Heptane, 3-[(Ethenyloxy)Methyl]-	High	18.2	JAC33,JAC60,JAC87,JAC102,JAC96,JAC88,JAC55,JAC99,JAC75,JAC47,JAC54,JAC31,JAC61A,JAC45,JAC64,JAC103,JAC74C,JAC48,JAC95,JAC25,JAC101,JAC122,JAC17,JAC78,JAC68,JAC72,JAC81,JAC30L,JAC76,JAC58,JAC86,JAC90,JAC74L,JAC6,JAC110,JAC24,JAC35
(*Z*,*Z*,*Z*)-6,9,15-Octadecatrienoic Acid Methyl Ester	High	18.33	JAC33,JAC87,JAC88,JAC55,JAC75,JAC54,JAC61A,JAC64,JAC103,JAC74C,JAC48,JAC95,JAC25,JAC81,JAC76,JAC58,JAC86,JAC74L,JAC110,JAC35
3-Ethenyl-2,5-Dibutyl-3-(4-Methyl-3-Pentenyl)Tetrahydrofuran	Low	18.33	JAC30L,JAC99,JAC90,JAC81,JAC75,JAC47,JAC78,JAC96,JAC48,JAC95,JAC103,JAC35,JAC88,JAC101,JAC68,JAC110,JAC45,JAC33,JAC64,JAC74L,JAC54,JAC6,JAC76,JAC60,JAC31,JAC61A,JAC122,JAC58,JAC72,JAC25,JAC102,JAC87,JAC17,JAC24,JAC55
3Beta-Acetoxy-20-Hydroxy-5Alpha-Cevan-6-One	High	18.38	JAC33,JAC60,JAC87,JAC102,JAC96,JAC88,JAC55,JAC99,JAC75,JAC47,JAC54,JAC31,JAC61A,JAC45,JAC64,JAC103,JAC74C,JAC48,JAC95,JAC25,JAC101,JAC122,JAC17,JAC78,JAC68,JAC72,JAC81,JAC30L

			,JAC76,JAC58,JAC86,JAC90,JAC74L,JAC6,JAC110,JAC24,JAC35
(*Z*,*Z*,*Z*)-9,12,15-Octadecatrienoic Acid Methyl Ester	High	18.59	JAC33,JAC87,JAC96,JAC88,JAC55,JAC99,JAC75,JAC47,JAC54,JAC31,JAC61A,JAC45,JAC64,JAC103,JAC74C,JAC95,JAC25,JAC122,JAC17,JAC68,JAC72,JAC81,JAC30L,JAC76,JAC58,JAC86,JAC90,JAC6,JAC110,JAC24,JAC35
Butanal, 3-Methyl-2-Methylene-, (1-Methylethyl)Hydrazone	High	19.1	JAC33,JAC87,JAC102,JAC88,JAC55,JAC99,JAC75,JAC47,JAC54,JAC31,JAC61A,JAC45,JAC64,JAC103,JAC48,JAC95,JAC25,JAC101,JAC122,JAC17,JAC78,JAC81,JAC76,JAC58,JAC86,JAC90,JAC74L,JAC110,JAC24,JAC35
Octyltrichlorosilane	Low	19.19	JAC30L,JAC99,JAC90,JAC81,JAC75,JAC47,JAC78,JAC96,JAC48,JAC95,JAC103,JAC35,JAC88,JAC101,JAC68,JAC110,JAC45,JAC33,JAC64,JAC74L,JAC54,JAC6,JAC76,JAC60,JAC31,JAC61A,JAC122,JAC58,JAC72,JAC102,JAC87,JAC17,JAC24,JAC55
1-Chloroeicosane	High	19.3	JAC33,JAC60,JAC102,JAC96,JAC88,JAC99,JAC75,JAC47,JAC54,JAC31,JAC61A,JAC45,JAC64,JAC103,JAC74C,JAC48,JAC95,JAC25,JAC101,JAC122,JAC17,JAC78,JAC68,JAC72,JAC81,JAC30L,JAC76,JAC58,JAC86,JAC6,JAC110,JAC24,JAC35
Propanoic Acid, 2-Methyl-, 1-(1,1-Dimethylethyl)-2-Methyl-1,3-Propanediyl Ester	High	19.46	JAC33,JAC60,JAC87,JAC102,JAC96,JAC88,JAC55,JAC99,JAC75,JAC54,JAC31,JAC61A,JAC45,JAC64,JAC103,JAC74C,JAC48,JAC95,JAC25,JAC101,JAC122,JAC17,JAC78,JAC68,JAC72,JAC81,JAC30L,JAC76,JAC58,JAC86,JAC90,JAC74L,JAC6,JAC110,JAC24,JAC35
10-Hexyl-9-Anthracenecarbaldehyde	Low	19.54	JAC30L,JAC99,JAC74C,JAC90,JAC81,JAC75,JAC47,JAC78,JAC96,JAC86,JAC48,JAC95,JAC103,JAC35,JAC88,JAC101,JAC68,JAC110,JAC45,JAC33,JAC64,JAC74L,JAC54,JAC6,JAC76,JAC60,JAC31,JAC61A,J

			AC122,JAC58,JAC72,JAC25,JAC102,JAC87,JAC17,JAC24,JAC55
Benztropine	Low	19.8	JAC30L,JAC99,JAC74C,JAC90,JAC81,JAC75,JAC47,JAC78,JAC96,JAC48,JAC95,JAC103,JAC35,JAC88,JAC68,JAC110,JAC45,JAC33,JAC64,JAC74L,JAC54,JAC6,JAC76,JAC60,JAC31,JAC61A,JAC58,JAC72,JAC25,JAC102,JAC87,JAC17,JAC24,JAC55
Malonic Acid, Di(4-Heptyl) Ester	High	19.95	JAC33,JAC60,JAC87,JAC96,JAC88,JAC55,JAC99,JAC75,JAC47,JAC54,JAC31,JAC61A,JAC45,JAC64,JAC103,JAC74C,JAC48,JAC95,JAC25,JAC101,JAC122,JAC17,JAC78,JAC68,JAC72,JAC81,JAC30L,JAC58,JAC90,JAC74L,JAC6,JAC110
4-(1-Methylethenyl)-2-Phenyl-1,3-Dioxolane	Low	19.95	JAC30L,JAC99,JAC74C,JAC90,JAC81,JAC75,JAC47,JAC78,JAC96,JAC86,JAC48,JAC95,JAC103,JAC88,JAC101,JAC68,JAC45,JAC33,JAC64,JAC74L,JAC54,JAC6,JAC76,JAC60,JAC31,JAC61A,JAC122,JAC58,JAC72,JAC25,JAC102,JAC87,JAC17,JAC24,JAC55
Heptyl Tetradecyl Ether	High	20.3	JAC33,JAC60,JAC87,JAC102,JAC96,JAC88,JAC55,JAC99,JAC75,JAC47,JAC54,JAC31,JAC61A,JAC45,JAC64,JAC103,JAC74C,JAC48,JAC95,JAC25,JAC101,JAC122,JAC17,JAC78,JAC68,JAC72,JAC81,JAC30L,JAC76,JAC58,JAC86,JAC90,JAC74L,JAC6,JAC110,JAC24,JAC35
(*Z*,*Z*,*Z*)-6,9,12-Octadecatrienoic Acid Methyl Ester	High	20.35	JAC33,JAC102,JAC99,JAC47,JAC45,JAC64,JAC103,JAC48,JAC101,JAC17,JAC72,JAC76,JAC86,JAC110,JAC24
2(3H)-Phenanthrenone, 4,4A,9,10-Tetrahydro-4A-Methyl-	Low	20.35	JAC30L,JAC99,JAC74C,JAC90,JAC81,JAC75,JAC47,JAC78,JAC96,JAC86,JAC48,JAC95,JAC103,JAC35,JAC88,JAC68,JAC110,JAC45,JAC33,JAC74L,JAC54,JAC6,JAC76,JAC31,JAC61A,JAC122,JAC58,JAC72,JAC25,JAC87,JAC17,JAC24,JAC55

(*Z*,*Z*,*Z*,*Z*)-6,9,12,15-Octadecatetraenoic Acid Methyl Ester	High	20.38	JAC60,JAC102,JAC88,JAC55,JAC75,JAC74C,JAC25,JAC101,JAC122,JAC78,JAC68,JAC81,JAC90,JAC6,JAC110,JAC35
4-Tert-Butylpyrocatechol	High	20.57	JAC60,JAC88,JAC55,JAC54,JAC61A,JAC74C,JAC48,JAC95,JAC17,JAC72,JAC74L,JAC110,JAC24
4-(Decyloxy)Benzaldehyde	High	20.87	JAC60,JAC87,JAC96,JAC88,JAC55,JAC99,JAC75,JAC47,JAC54,JAC61A,JAC45,JAC64,JAC103,JAC74C,JAC48,JAC95,JAC122,JAC68,JAC72,JAC30L,JAC76,JAC86,JAC90,JAC74L,JAC6,JAC110,JAC24,JAC35
4-Hydroxy-3-Methoxyphenylacetic Acid (2,5,6-D3,Alpha,Alpha-D2)	Low	20.87	JAC74C,JAC90,JAC47,JAC78,JAC96,JAC86,JAC48,JAC95,JAC88,JAC101,JAC68,JAC110,JAC45,JAC33,JAC64,JAC74L,JAC54,JAC60,JAC31,JAC58,JAC102,JAC87,JAC17,JAC24
1-Chloromethyl-3,5-Bis(1,1-Dimethylethyl)Benzene	High	20.98	JAC33,JAC60,JAC87,JAC102,JAC96,JAC88,JAC55,JAC99,JAC75,JAC47,JAC54,JAC31,JAC61A,JAC45,JAC64,JAC103,JAC74C,JAC48,JAC95,JAC101,JAC122,JAC17,JAC78,JAC68,JAC72,JAC81,JAC30L,JAC76,JAC58,JAC86,JAC90,JAC74L,JAC6,JAC110,JAC24,JAC35
Methyl 3,4-O-Isopropylidene-Beta-D-Fucopyranoside	High	21.06	JAC33,JAC60,JAC87,JAC96,JAC88,JAC55,JAC99,JAC75,JAC31,JAC45,JAC64,JAC103,JAC74C,JAC48,JAC95,JAC25,JAC122,JAC17,JAC78,JAC68,JAC72,JAC81,JAC76,JAC58,JAC86,JAC90,JAC74L,JAC6,JAC110,JAC24,JAC35
9,12-Octadecadienoyl Chloride, (*Z*,*Z*)-	High	21.34	JAC33,JAC60,JAC87,JAC88,JAC99,JAC75,JAC47,JAC54,JAC45,JAC64,JAC103,JAC95,JAC17,JAC78,JAC76,JAC86,JAC6,JAC24
5Alpha-Androst-16-En-3-One	Low	21.34	JAC30L,JAC99,JAC74C,JAC81,JAC75,JAC47,JAC78,JAC96,JAC86,JAC48,JAC95,JAC103,JAC35,JAC88,JAC101,JAC110,JAC33,JAC64,JAC54,JAC6,JAC76,JA

			C60,JAC31,JAC61A,JAC122,JAC58,JAC72,JAC25,JAC17,JAC24,JAC55
Decyl Octyl Ether	High	21.41	JAC33,JAC60,JAC87,JAC102,JAC96,JAC88,JAC55,JAC99,JAC75,JAC47,JAC54,JAC61A,JAC64,JAC103,JAC48,JAC95,JAC25,JAC101,JAC122,JAC17,JAC78,JAC68,JAC72,JAC81,JAC30L,JAC76,JAC58,JAC86,JAC90,JAC74L,JAC6,JAC110,JAC24,JAC35
1,4-Dibenzoyl-2,Trans-5-Diethylpiperazine	Low	21.98	JAC30L,JAC99,JAC74C,JAC90,JAC81,JAC75,JAC47,JAC78,JAC96,JAC86,JAC48,JAC95,JAC103,JAC35,JAC88,JAC101,JAC68,JAC110,JAC45,JAC33,JAC64,JAC74L,JAC54,JAC76,JAC60,JAC31,JAC61A,JAC122,JAC58,JAC72,JAC25,JAC102,JAC87,JAC17,JAC24,JAC55
1,9-Dichlorononane	High	22.19	JAC33,JAC60,JAC87,JAC102,JAC96,JAC88,JAC55,JAC99,JAC75,JAC47,JAC54,JAC31,JAC45,JAC64,JAC103,JAC48,JAC25,JAC101,JAC122,JAC17,JAC78,JAC72,JAC30L,JAC76,JAC58,JAC86,JAC90,JAC74L,JAC6,JAC24
5-Bromo-2,6-Dimethyl-4-Pyrimidinamine	Low	22.28	JAC99,JAC74C,JAC47,JAC78,JAC96,JAC86,JAC48,JAC103,JAC88,JAC45,JAC33,JAC76,JAC60,JAC31,JAC58,JAC25,JAC102,JAC17,JAC24
Cyclohexasiloxane, Dodecamethyl-	High	22.71	JAC60,JAC87,JAC96,JAC88,JAC55,JAC99,JAC75,JAC54,JAC61A,JAC64,JAC103,JAC74C,JAC48,JAC95,JAC25,JAC122,JAC68,JAC72,JAC81,JAC30L,JAC76,JAC86,JAC90,JAC74L,JAC6,JAC110,JAC35
9,12-Dioxo-5,6,7,9,12,14,15,16-Octahydro-6,15-Methanobenzo(A)Naphtho(2,3-F)Cyclodecene	Low	23.44	JAC30L,JAC99,JAC74C,JAC90,JAC81,JAC75,JAC47,JAC78,JAC96,JAC86,JAC48,JAC95,JAC103,JAC35,JAC88,JAC101,JAC68,JAC110,JAC33,JAC64,JAC74L,JAC54,JAC6,JAC76,JAC60,JAC31,JAC61A,JAC122,JAC58,JAC72,JAC25,JAC87,JAC17,JAC55

Mevinoline	Low	23.44	JAC30L,JAC99,JAC74C,JAC81,JAC75,JAC48,JAC103,JAC88,JAC68,JAC45,JAC6,JAC76,JAC61A,JAC72,JAC87,JAC55
(6-Bromo-(*E*)-1-Hexenyl)Benzene	Low	23.74	JAC30L,JAC99,JAC74C,JAC90,JAC81,JAC75,JAC47,JAC78,JAC96,JAC86,JAC48,JAC95,JAC103,JAC35,JAC88,JAC101,JAC68,JAC110,JAC45,JAC33,JAC64,JAC74L,JAC54,JAC6,JAC60,JAC31,JAC61A,JAC122,JAC58,JAC72,JAC25,JAC102,JAC87,JAC17,JAC24,JAC55
5'-Chloro-2'-Methoxyacetanilide	Low	23.78	JAC30L,JAC99,JAC74C,JAC90,JAC47,JAC78,JAC96,JAC48,JAC95,JAC35,JAC88,JAC101,JAC68,JAC110,JAC74L,JAC54,JAC6,JAC60,JAC122,JAC25,JAC102,JAC87,JAC17,JAC24,JAC55
(Alpha,Alpha,Alpha-Trifluoro-Para-Tolyl)Acetic Acid	High	23.89	JAC33,JAC60,JAC87,JAC96,JAC88,JAC55,JAC99,JAC75,JAC47,JAC54,JAC31,JAC61A,JAC45,JAC64,JAC103,JAC74C,JAC48,JAC95,JAC25,JAC101,JAC122,JAC17,JAC78,JAC68,JAC72,JAC81,JAC30L,JAC76,JAC86,JAC90,JAC74L,JAC6,JAC110,JAC24,JAC35
Eicosane, 1-Iodo-	High	24.1	JAC60,JAC87,JAC102,JAC96,JAC88,JAC55,JAC99,JAC75,JAC47,JAC54,JAC61A,JAC103,JAC48,JAC95,JAC25,JAC101,JAC122,JAC17,JAC78,JAC68,JAC72,JAC81,JAC30L,JAC76,JAC58,JAC90,JAC74L,JAC6,JAC110,JAC35
1-Adamantaneacetic Acid	High	24.38	JAC33,JAC60,JAC87,JAC102,JAC96,JAC88,JAC55,JAC99,JAC75,JAC47,JAC54,JAC31,JAC61A,JAC45,JAC64,JAC103,JAC74C,JAC48,JAC95,JAC25,JAC101,JAC122,JAC17,JAC78,JAC68,JAC72,JAC81,JAC30L,JAC58,JAC86,JAC90,JAC74L,JAC6,JAC110,JAC24,JAC35
Butane, 1-Iodo-3-Methyl-	High	24.56	JAC33,JAC60,JAC87,JAC102,JAC96,JAC55,JAC99,JAC75,JAC54,JAC31,JAC61A,JAC45,JAC64,JAC103,JAC74C,JAC48,JAC95,JAC25,JAC101,JAC122,JAC17

			,JAC78,JAC68,JAC72,JAC81,JAC30L,JAC76,JAC58,JAC86,JAC90,JAC74L,JAC6,JAC110,JAC24,JAC35
1-Iodooctane	High	24.74	JAC33,JAC60,JAC87,JAC102,JAC99,JAC75,JAC47,JAC31,JAC45,JAC64,JAC103,JAC74C,JAC48,JAC95,JAC25,JAC101,JAC122,JAC17,JAC78,JAC68,JAC30L,JAC76,JAC58,JAC86,JAC6,JAC24,JAC35
Cis-1-Chloro-9-Octadecene	High	24.92	JAC33,JAC60,JAC87,JAC102,JAC96,JAC88,JAC55,JAC99,JAC75,JAC47,JAC54,JAC31,JAC61A,JAC45,JAC64,JAC103,JAC74C,JAC48,JAC95,JAC25,JAC101,JAC122,JAC17,JAC78,JAC68,JAC72,JAC81,JAC30L,JAC76,JAC58,JAC86,JAC90,JAC74L,JAC6,JAC110,JAC24,JAC35
Distearyl Thiodipropionate	High	25.76	JAC33,JAC60,JAC87,JAC102,JAC96,JAC55,JAC99,JAC75,JAC47,JAC31,JAC45,JAC64,JAC103,JAC74C,JAC101,JAC122,JAC17,JAC78,JAC68,JAC76,JAC86,JAC90,JAC6,JAC110,JAC24,JAC35
Acetamide, 2-(Thiophen-2-Yl)-*N*-Methyl-*N*-Decyl-	Low	26.27	JAC30L,JAC99,JAC74C,JAC90,JAC81,JAC75,JAC47,JAC78,JAC95,JAC35,JAC88,JAC101,JAC68,JAC110,JAC33,JAC64,JAC74L,JAC6,JAC76,JAC60,JAC31,JAC61A,JAC122,JAC58,JAC72,JAC25,JAC87,JAC17,JAC24,JAC55
Docosanoic Anhydride	High	26.67	JAC33,JAC60,JAC87,JAC102,JAC96,JAC88,JAC55,JAC99,JAC75,JAC47,JAC54,JAC31,JAC61A,JAC45,JAC64,JAC103,JAC74C,JAC48,JAC95,JAC25,JAC101,JAC122,JAC17,JAC78,JAC68,JAC72,JAC81,JAC30L,JAC76,JAC58,JAC86,JAC90,JAC74L,JAC6,JAC110,JAC24,JAC35
Docosyl Pentyl Ether	High	26.8	JAC33,JAC60,JAC87,JAC102,JAC96,JAC88,JAC55,JAC99,JAC75,JAC47,JAC54,JAC31,JAC61A,JAC45,JAC64,JAC103,JAC74C,JAC48,JAC95,JAC25,JAC101,JAC122,JAC17,JAC78,JAC68,JAC72,JAC81,JAC30L

			,JAC76,JAC58,JAC86,JAC90,JAC74L,JAC6,JAC110,JAC24,JAC35
Pentadecafluorooctanoic Acid, Undecyl Ester	High	26.93	JAC33,JAC60,JAC87,JAC96,JAC88,JAC55,JAC99,JAC75,JAC47,JAC54,JAC31,JAC61A,JAC45,JAC64,JAC103,JAC74C,JAC48,JAC95,JAC25,JAC101,JAC122,JAC17,JAC78,JAC68,JAC72,JAC81,JAC30L,JAC76,JAC58,JAC86,JAC74L,JAC6,JAC110,JAC24,JAC35
11-Bromoundecanoic Acid	Low	27.49	JAC30L,JAC99,JAC74C,JAC75,JAC47,JAC48,JAC103,JAC35,JAC88,JAC101,JAC45,JAC33,JAC64,JAC6,JAC76,JAC31,JAC61A,JAC58,JAC72,JAC25,JAC17,JAC55
2-Bromo Dodecane	High	27.71	JAC87,JAC96,JAC88,JAC55,JAC99,JAC75,JAC54,JAC61A,JAC45,JAC95,JAC25,JAC101,JAC122,JAC78,JAC68,JAC72,JAC81,JAC30L,JAC76,JAC86,JAC90,JAC74L,JAC6,JAC110,JAC35
Maltitol	High	28.08	JAC33,JAC60,JAC87,JAC102,JAC88,JAC55,JAC99,JAC47,JAC54,JAC31,JAC61A,JAC45,JAC64,JAC103,JAC74C,JAC48,JAC25,JAC101,JAC17,JAC78,JAC81,JAC30L,JAC76,JAC58,JAC86,JAC110,JAC24
Cyclobutanecarboxamide, *N*-Octyl-	High	28.71	JAC33,JAC88,JAC99,JAC47,JAC31,JAC64,JAC103,JAC74C,JAC48,JAC25,JAC122,JAC17,JAC72,JAC76,JAC86,JAC90,JAC6,JAC24
2-(3-Benzoylphenyl)Propionic Acid Trimethylsilyl Ester	High	29.04	JAC60,JAC87,JAC102,JAC96,JAC88,JAC99,JAC54,JAC64,JAC103,JAC95,JAC101,JAC17,JAC78,JAC68,JAC30L,JAC76,JAC90,JAC35
Oct-3-Enoylamide, *N*-Methyl-*N*-Pentyl-	High	29.42	JAC60,JAC87,JAC102,JAC96,JAC88,JAC55,JAC99,JAC75,JAC47,JAC54,JAC31,JAC61A,JAC45,JAC64,JAC74C,JAC48,JAC95,JAC25,JAC101,JAC122,JAC17,JAC78,JAC68,JAC72,JAC81,JAC30L,JAC76,JAC58,JAC86,JAC90,JAC74L,JAC6,JAC110,JAC24,JAC35

2-Hydroxy-3-Isopropyl-5-Piperidinomethyl-2,4,6-Cycloheptatrien-1-One	Low	29.76	JAC30L,JAC99,JAC74C,JAC90,JAC81,JAC75,JAC78,JAC86,JAC48,JAC95,JAC103,JAC35,JAC88,JAC101,JAC68,JAC110,JAC54,JAC31,JAC58,JAC25,JAC55
Perfluorotributylamine	High	30.36	JAC87,JAC96,JAC88,JAC55,JAC54,JAC61A,JAC95,JAC25,JAC122,JAC68,JAC72,JAC81,JAC30L,JAC6,JAC110,JAC35
Violuric Acid	High	30.72	JAC87,JAC96,JAC88,JAC55,JAC75,JAC61A,JAC95,JAC25,JAC122,JAC68,JAC72,JAC90,JAC74L,JAC6,JAC110,JAC35
Uric Acid	Low	31.24	JAC30L,JAC81,JAC75,JAC96,JAC95,JAC35,JAC88,JAC68,JAC110,JAC74L,JAC54,JAC6,JAC61A,JAC122,JAC72,JAC25,JAC87,JAC55
1-Octanamine, *N*-Methyl-	High	31.53	JAC88,JAC55,JAC75,JAC54,JAC61A,JAC122,JAC68,JAC72,JAC81,JAC30L,JAC74L,JAC6,JAC110,JAC35
Perfluoro(2-Methylpentane)	High	31.53	JAC87,JAC96,JAC88,JAC75,JAC54,JAC61A,JAC95,JAC25,JAC122,JAC68,JAC72,JAC30L,JAC74L,JAC6,JAC110
Perfluoro-1,1-Dimethylcyclopentane	High	31.66	JAC87,JAC96,JAC88,JAC55,JAC61A,JAC95,JAC25,JAC122,JAC72,JAC81,JAC30L,JAC74L,JAC6,JAC110
1-(P-Bromobenzenesulfonyl)Piperidine	Low	31.66	JAC30L,JAC90,JAC81,JAC95,JAC88,JAC68,JAC110,JAC54,JAC72,JAC87
Disulfide, Di-Tert-Dodecyl	High	31.77	JAC87,JAC96,JAC88,JAC55,JAC75,JAC54,JAC61A,JAC95,JAC25,JAC122,JAC68,JAC72,JAC81,JAC30L,JAC90,JAC74L,JAC6,JAC110,JAC35
**Annotated as VocBinbase Bin Numbers (93)**			
Fiehn VocBinbase Bin #371	Low	4.08	JAC30L,JAC99,JAC74C,JAC90,JAC81,JAC47,JAC78,JAC96,JAC86,JAC48,JAC95,JAC103,JAC35,JAC88,JAC101,JAC68,JAC110,JAC45,JAC33,JAC64,JAC74L,

			JAC54,JAC76,JAC60,JAC31,JAC61A,JAC122,JAC58,JAC102,JAC87,JAC17,JAC24,JAC55
Fiehn VocBinbase Bin #176	High	4.64	JAC33,JAC60,JAC87,JAC102,JAC96,JAC88,JAC55,JAC99,JAC75,JAC47,JAC54,JAC31,JAC61A,JAC45,JAC64,JAC103,JAC74C,JAC48,JAC95,JAC25,JAC101,JAC122,JAC17,JAC78,JAC68,JAC72,JAC81,JAC30L,JAC76,JAC58,JAC86,JAC90,JAC74L,JAC6,JAC110,JAC24,JAC35
Fiehn VocBinbase Bin #847	High	4.77	JAC33,JAC60,JAC87,JAC102,JAC96,JAC55,JAC99,JAC75,JAC47,JAC54,JAC31,JAC61A,JAC45,JAC64,JAC103,JAC74C,JAC48,JAC95,JAC25,JAC101,JAC122,JAC17,JAC78,JAC68,JAC72,JAC81,JAC30L,JAC76,JAC58,JAC86,JAC90,JAC74L,JAC6,JAC110,JAC24,JAC35
Fiehn VocBinbase Bin #1498	High	4.86	JAC33,JAC60,JAC87,JAC102,JAC99,JAC75,JAC47,JAC54,JAC31,JAC61A,JAC45,JAC64,JAC103,JAC74C,JAC48,JAC95,JAC25,JAC101,JAC122,JAC17,JAC78,JAC68,JAC72,JAC81,JAC30L,JAC76,JAC58,JAC86,JAC90,JAC6,JAC110,JAC24,JAC35
Fiehn VocBinbase Bin #1063	High	4.95	JAC33,JAC60,JAC102,JAC88,JAC47,JAC31,JAC61A,JAC45,JAC64,JAC74C,JAC95,JAC25,JAC122,JAC17,JAC78,JAC72,JAC30L,JAC76,JAC58,JAC86,JAC90,JAC74L,JAC6,JAC110,JAC24,JAC35
Fiehn VocBinbase Bin #387	Low	4.95	JAC99,JAC90,JAC75,JAC47,JAC78,JAC86,JAC48,JAC95,JAC103,JAC35,JAC88,JAC110,JAC45,JAC64,JAC74L,JAC6,JAC76,JAC60,JAC61A,JAC122,JAC58,JAC72,JAC102,JAC87,JAC17,JAC55
Fiehn VocBinbase Bin #780	Low	4.95	JAC30L,JAC99,JAC74C,JAC90,JAC81,JAC75,JAC78,JAC96,JAC86,JAC48,JAC95,JAC35,JAC88,JAC68,JAC110,JAC45,JAC33,JAC64,JAC74L,JAC54,JAC6,J

			AC76,JAC60,JAC31,JAC61A,JAC122,JAC72,JAC25,JAC102,JAC87,JAC17,JAC24,JAC55
Fiehn VocBinbase Bin #1375	High	5.07	JAC33,JAC60,JAC87,JAC102,JAC96,JAC88,JAC55,JAC99,JAC75,JAC47,JAC54,JAC31,JAC61A,JAC45,JAC64,JAC103,JAC74C,JAC48,JAC95,JAC25,JAC101,JAC122,JAC17,JAC78,JAC68,JAC72,JAC81,JAC30L,JAC76,JAC58,JAC86,JAC90,JAC74L,JAC6,JAC110,JAC24,JAC35
Fiehn VocBinbase Bin #838	High	5.2	JAC33,JAC60,JAC102,JAC96,JAC88,JAC55,JAC99,JAC75,JAC47,JAC54,JAC31,JAC61A,JAC45,JAC64,JAC103,JAC74C,JAC48,JAC95,JAC25,JAC101,JAC122,JAC17,JAC78,JAC68,JAC72,JAC81,JAC30L,JAC76,JAC58,JAC86,JAC90,JAC74L,JAC6,JAC110,JAC24,JAC35
Fiehn VocBinbase Bin #754	High	5.54	JAC33,JAC60,JAC102,JAC88,JAC55,JAC99,JAC75,JAC47,JAC31,JAC45,JAC64,JAC103,JAC48,JAC25,JAC17,JAC78,JAC72,JAC81,JAC30L,JAC76,JAC58,JAC90,JAC74L,JAC110,JAC24
Fiehn VocBinbase Bin #509	High	5.84	JAC60,JAC87,JAC88,JAC55,JAC99,JAC75,JAC54,JAC61A,JAC103,JAC74C,JAC48,JAC25,JAC101,JAC122,JAC17,JAC78,JAC72,JAC81,JAC30L,JAC76,JAC58,JAC86,JAC90,JAC74L,JAC6,JAC35
Fiehn VocBinbase Bin #37	High	5.84	JAC33,JAC60,JAC87,JAC102,JAC96,JAC88,JAC55,JAC99,JAC47,JAC31,JAC61A,JAC45,JAC64,JAC103,JAC74C,JAC48,JAC95,JAC25,JAC101,JAC122,JAC17,JAC78,JAC68,JAC72,JAC81,JAC30L,JAC76,JAC58,JAC86,JAC90,JAC74L,JAC6,JAC110,JAC24,JAC35
Fiehn VocBinbase Bin #39	High	6.1	JAC87,JAC96,JAC88,JAC55,JAC99,JAC75,JAC54,JAC61A,JAC103,JAC95,JAC25,JAC101,JAC122,JAC78,JAC68,JAC72,JAC30L,JAC90,JAC74L,JAC6,JAC110,JAC35

Fiehn VocBinbase Bin #1081	High	6.14	JAC33,JAC60,JAC102,JAC88,JAC99,JAC47,JAC31,JAC45,JAC64,JAC103,JAC48,JAC95,JAC101,JAC122,JAC17,JAC78,JAC72,JAC81,JAC30L,JAC76,JAC58,JAC86,JAC6,JAC24
Fiehn VocBinbase Bin #454	High	6.31	JAC87,JAC96,JAC88,JAC55,JAC75,JAC47,JAC54,JAC61A,JAC64,JAC74C,JAC95,JAC25,JAC101,JAC78,JAC68,JAC72,JAC81,JAC30L,JAC76,JAC90,JAC74L,JAC6,JAC110,JAC35
Fiehn VocBinbase Bin #467	Low	6.31	JAC30L,JAC90,JAC81,JAC75,JAC78,JAC96,JAC86,JAC48,JAC95,JAC103,JAC68,JAC110,JAC45,JAC33,JAC64,JAC74L,JAC6,JAC60,JAC31,JAC58,JAC72,JAC25,JAC102,JAC87,JAC17,JAC24
Fiehn VocBinbase Bin #888	High	6.75	JAC33,JAC60,JAC87,JAC102,JAC96,JAC88,JAC55,JAC54,JAC31,JAC61A,JAC45,JAC64,JAC74C,JAC95,JAC25,JAC122,JAC17,JAC78,JAC30L,JAC90,JAC74L,JAC110,JAC24,JAC35
Fiehn VocBinbase Bin #829	Low	7.14	JAC30L,JAC99,JAC74C,JAC90,JAC81,JAC75,JAC47,JAC78,JAC96,JAC86,JAC48,JAC95,JAC103,JAC35,JAC88,JAC101,JAC68,JAC110,JAC45,JAC33,JAC64,JAC74L,JAC54,JAC6,JAC60,JAC31,JAC61A,JAC122,JAC58,JAC72,JAC25,JAC102,JAC87,JAC17,JAC55
Fiehn VocBinbase Bin #1442	Low	7.53	JAC30L,JAC99,JAC74C,JAC90,JAC81,JAC75,JAC47,JAC78,JAC96,JAC86,JAC48,JAC95,JAC103,JAC35,JAC88,JAC101,JAC68,JAC45,JAC33,JAC64,JAC74L,JAC54,JAC6,JAC76,JAC60,JAC31,JAC61A,JAC122,JAC58,JAC72,JAC25,JAC102,JAC87,JAC17,JAC24,JAC55
Fiehn VocBinbase Bin #212	High	7.65	JAC33,JAC60,JAC87,JAC102,JAC96,JAC88,JAC55,JAC99,JAC75,JAC47,JAC54,JAC31,JAC61A,JAC74C,JAC48,JAC95,JAC25,JAC122,JAC17,JAC78,JAC68,J

			AC72,JAC81,JAC30L,JAC76,JAC58,JAC86,JAC90,JAC74L,JAC6,JAC110,JAC24,JAC35
Fiehn VocBinbase Bin #518	Low	7.79	JAC30L,JAC99,JAC81,JAC75,JAC47,JAC78,JAC96,JAC86,JAC48,JAC95,JAC103,JAC35,JAC88,JAC101,JAC68,JAC110,JAC45,JAC33,JAC64,JAC74L,JAC54,JAC6,JAC76,JAC60,JAC31,JAC61A,JAC122,JAC58,JAC72,JAC25,JAC87,JAC55
Fiehn VocBinbase Bin #113	Low	7.83	JAC30L,JAC99,JAC90,JAC81,JAC47,JAC78,JAC96,JAC86,JAC48,JAC95,JAC103,JAC88,JAC68,JAC110,JAC45,JAC33,JAC64,JAC74L,JAC54,JAC6,JAC76,JAC60,JAC31,JAC61A,JAC72,JAC25,JAC102,JAC87,JAC24,JAC55
Fiehn VocBinbase Bin #493	High	8.38	JAC33,JAC60,JAC87,JAC96,JAC88,JAC55,JAC99,JAC54,JAC31,JAC61A,JAC48,JAC95,JAC25,JAC122,JAC17,JAC78,JAC68,JAC58,JAC90,JAC74L,JAC6,JAC110,JAC24
Fiehn VocBinbase Bin #1417	Low	8.58	JAC30L,JAC99,JAC74C,JAC90,JAC81,JAC75,JAC47,JAC78,JAC96,JAC86,JAC48,JAC95,JAC103,JAC35,JAC88,JAC68,JAC110,JAC45,JAC33,JAC64,JAC74L,JAC54,JAC6,JAC76,JAC60,JAC31,JAC61A,JAC122,JAC58,JAC72,JAC25,JAC102,JAC87,JAC17,JAC24,JAC55
Fiehn VocBinbase Bin #542	Low	8.58	JAC99,JAC90,JAC81,JAC47,JAC78,JAC96,JAC95,JAC103,JAC35,JAC101,JAC68,JAC110,JAC45,JAC33,JAC64,JAC6,JAC76,JAC60,JAC61A,JAC122,JAC58,JAC25,JAC102,JAC17
Fiehn VocBinbase Bin #1007	High	8.82	JAC33,JAC87,JAC96,JAC88,JAC55,JAC99,JAC54,JAC31,JAC64,JAC48,JAC95,JAC25,JAC122,JAC78,JAC58,JAC90,JAC6,JAC110,JAC24,JAC35
Fiehn VocBinbase Bin #896	Low	8.88	JAC30L,JAC99,JAC74C,JAC90,JAC81,JAC75,JAC47,JAC78,JAC96,JAC48,JAC95,JAC103,JAC35,JAC88,J

			AC101,JAC68,JAC110,JAC33,JAC64,JAC74L,JAC54,JAC6,JAC76,JAC60,JAC31,JAC61A,JAC58,JAC72,JAC25,JAC87,JAC17,JAC24,JAC55
Fiehn VocBinbase Bin #580	High	9.21	JAC33,JAC60,JAC102,JAC55,JAC99,JAC64,JAC103,JAC74C,JAC48,JAC95,JAC25,JAC122,JAC17,JAC78,JAC76,JAC86,JAC6,JAC24,JAC35
Fiehn VocBinbase Bin #579	High	9.29	JAC33,JAC87,JAC96,JAC88,JAC55,JAC99,JAC75,JAC47,JAC54,JAC31,JAC61A,JAC45,JAC64,JAC103,JAC48,JAC95,JAC25,JAC122,JAC17,JAC78,JAC68,JAC72,JAC81,JAC30L,JAC76,JAC58,JAC86,JAC90,JAC74L,JAC6,JAC110,JAC24,JAC35
Fiehn VocBinbase Bin #226	High	9.83	JAC33,JAC60,JAC87,JAC102,JAC96,JAC88,JAC55,JAC99,JAC75,JAC47,JAC54,JAC31,JAC61A,JAC45,JAC64,JAC103,JAC74C,JAC48,JAC95,JAC25,JAC101,JAC122,JAC17,JAC78,JAC68,JAC72,JAC81,JAC30L,JAC76,JAC58,JAC86,JAC90,JAC74L,JAC6,JAC110,JAC24,JAC35
Fiehn VocBinbase Bin #917	High	10.55	JAC33,JAC60,JAC102,JAC88,JAC99,JAC75,JAC47,JAC54,JAC31,JAC61A,JAC45,JAC64,JAC103,JAC48,JAC25,JAC122,JAC17,JAC78,JAC72,JAC81,JAC30L,JAC76,JAC58,JAC90,JAC74L,JAC110,JAC35
Fiehn VocBinbase Bin #1372	High	10.65	JAC60,JAC87,JAC102,JAC96,JAC88,JAC55,JAC47,JAC45,JAC64,JAC74C,JAC95,JAC25,JAC101,JAC122,JAC68,JAC72,JAC90,JAC74L,JAC6
Fiehn VocBinbase Bin #568	High	10.89	JAC33,JAC60,JAC102,JAC99,JAC75,JAC47,JAC54,JAC31,JAC45,JAC64,JAC103,JAC74C,JAC48,JAC25,JAC101,JAC17,JAC78,JAC68,JAC72,JAC81,JAC30L,JAC76,JAC58,JAC86,JAC6,JAC24,JAC35
Fiehn VocBinbase Bin #50	High	10.89	JAC33,JAC87,JAC96,JAC88,JAC55,JAC99,JAC75,JAC47,JAC54,JAC31,JAC61A,JAC45,JAC103,JAC48,JAC95,JAC25,JAC122,JAC17,JAC78,JAC68,JAC72,JAC

			81,JAC30L,JAC76,JAC58,JAC86,JAC90,JAC74L,JAC6,JAC110,JAC24,JAC35
Fiehn VocBinbase Bin #680	High	12.67	JAC33,JAC60,JAC87,JAC102,JAC96,JAC88,JAC55,JAC99,JAC75,JAC47,JAC54,JAC31,JAC61A,JAC45,JAC64,JAC103,JAC74C,JAC48,JAC95,JAC25,JAC101,JAC122,JAC17,JAC78,JAC68,JAC72,JAC81,JAC30L,JAC76,JAC58,JAC86,JAC90,JAC74L,JAC6,JAC110,JAC24,JAC35
Fiehn VocBinbase Bin #846	Low	13.09	JAC30L,JAC90,JAC81,JAC75,JAC47,JAC78,JAC96,JAC48,JAC95,JAC103,JAC35,JAC88,JAC101,JAC68,JAC110,JAC45,JAC74L,JAC54,JAC6,JAC60,JAC61A,JAC122,JAC72,JAC25,JAC102,JAC87,JAC17,JAC55
Fiehn VocBinbase Bin #402	High	13.14	JAC33,JAC60,JAC87,JAC102,JAC88,JAC99,JAC75,JAC47,JAC31,JAC45,JAC64,JAC103,JAC74C,JAC48,JAC25,JAC101,JAC122,JAC17,JAC78,JAC72,JAC81,JAC30L,JAC76,JAC58,JAC86,JAC90,JAC74L,JAC24
Fiehn VocBinbase Bin #191	High	13.72	JAC60,JAC87,JAC102,JAC96,JAC88,JAC55,JAC99,JAC75,JAC47,JAC54,JAC103,JAC74C,JAC48,JAC95,JAC25,JAC101,JAC122,JAC17,JAC68,JAC72,JAC81,JAC30L,JAC90,JAC74L,JAC6,JAC110,JAC35
Fiehn VocBinbase Bin #352	High	14.24	JAC33,JAC60,JAC102,JAC96,JAC88,JAC55,JAC99,JAC75,JAC47,JAC31,JAC45,JAC64,JAC103,JAC74C,JAC48,JAC25,JAC101,JAC122,JAC17,JAC78,JAC72,JAC81,JAC30L,JAC76,JAC58,JAC86,JAC74L,JAC24
Fiehn VocBinbase Bin #1475	High	14.92	JAC33,JAC87,JAC102,JAC96,JAC88,JAC55,JAC99,JAC75,JAC54,JAC31,JAC61A,JAC103,JAC48,JAC95,JAC101,JAC122,JAC17,JAC78,JAC68,JAC72,JAC81,JAC30L,JAC76,JAC58,JAC86,JAC90,JAC74L,JAC6,JAC110,JAC24,JAC35
Fiehn VocBinbase Bin #993	Low	15.71	JAC30L,JAC90,JAC47,JAC78,JAC96,JAC48,JAC95,JAC103,JAC35,JAC88,JAC101,JAC68,JAC33,JAC74L,

			JAC54,JAC6,JAC61A,JAC122,JAC58,JAC72,JAC25,JAC102,JAC24,JAC55
Fiehn VocBinbase Bin #545	High	16.03	JAC33,JAC60,JAC87,JAC102,JAC96,JAC88,JAC55,JAC99,JAC75,JAC47,JAC54,JAC31,JAC61A,JAC45,JAC64,JAC103,JAC74C,JAC48,JAC95,JAC25,JAC101,JAC122,JAC17,JAC78,JAC68,JAC72,JAC81,JAC30L,JAC76,JAC58,JAC86,JAC90,JAC74L,JAC6,JAC110,JAC24,JAC35
Fiehn VocBinbase Bin #1022	High	16.48	JAC33,JAC60,JAC102,JAC99,JAC75,JAC47,JAC31,JAC45,JAC64,JAC103,JAC74C,JAC48,JAC25,JAC101,JAC122,JAC17,JAC78,JAC72,JAC30L,JAC76,JAC58,JAC86,JAC24,JAC35
Fiehn VocBinbase Bin #1406	Low	16.52	JAC30L,JAC99,JAC74C,JAC90,JAC81,JAC75,JAC47,JAC78,JAC96,JAC86,JAC48,JAC103,JAC35,JAC88,JAC101,JAC68,JAC110,JAC45,JAC33,JAC64,JAC74L,JAC54,JAC6,JAC76,JAC60,JAC31,JAC61A,JAC122,JAC58,JAC72,JAC25,JAC102,JAC87,JAC17,JAC24,JAC55
Fiehn VocBinbase Bin #921	High	16.85	JAC33,JAC60,JAC87,JAC96,JAC88,JAC55,JAC99,JAC75,JAC47,JAC54,JAC61A,JAC45,JAC64,JAC103,JAC74C,JAC48,JAC95,JAC25,JAC101,JAC122,JAC17,JAC78,JAC68,JAC72,JAC81,JAC30L,JAC76,JAC58,JAC86,JAC90,JAC74L,JAC6,JAC110,JAC35
Fiehn VocBinbase Bin #524	High	17.48	JAC60,JAC87,JAC96,JAC88,JAC55,JAC99,JAC75,JAC47,JAC54,JAC61A,JAC103,JAC48,JAC95,JAC101,JAC122,JAC17,JAC68,JAC72,JAC81,JAC30L,JAC76,JAC90,JAC74L,JAC6,JAC110,JAC35
Fiehn VocBinbase Bin #1039	High	17.52	JAC33,JAC60,JAC87,JAC102,JAC96,JAC88,JAC55,JAC99,JAC75,JAC47,JAC54,JAC31,JAC61A,JAC45,JAC64,JAC103,JAC74C,JAC48,JAC95,JAC25,JAC101,JAC122,JAC17,JAC78,JAC68,JAC72,JAC81,JAC30L

			,JAC76,JAC86,JAC90,JAC74L,JAC6,JAC110,JAC24,JAC35
Fiehn VocBinbase Bin #1115	High	17.86	JAC33,JAC60,JAC102,JAC88,JAC55,JAC99,JAC47,JAC54,JAC61A,JAC45,JAC64,JAC103,JAC74C,JAC48,JAC95,JAC122,JAC17,JAC78,JAC68,JAC72,JAC81,JAC30L,JAC76,JAC58,JAC86,JAC90,JAC74L,JAC110,JAC24,JAC35
Fiehn VocBinbase Bin #487	High	18.06	JAC33,JAC60,JAC87,JAC102,JAC96,JAC88,JAC55,JAC99,JAC75,JAC47,JAC54,JAC31,JAC61A,JAC45,JAC64,JAC103,JAC74C,JAC48,JAC95,JAC101,JAC122,JAC17,JAC78,JAC68,JAC72,JAC81,JAC30L,JAC76,JAC58,JAC86,JAC90,JAC74L,JAC6,JAC110,JAC24,JAC35
Fiehn VocBinbase Bin #491	High	18.15	JAC33,JAC60,JAC87,JAC96,JAC88,JAC55,JAC99,JAC75,JAC54,JAC61A,JAC103,JAC74C,JAC48,JAC95,JAC25,JAC101,JAC122,JAC78,JAC68,JAC72,JAC81,JAC30L,JAC76,JAC58,JAC86,JAC90,JAC74L,JAC6,JAC110,JAC35
Fiehn VocBinbase Bin #799	High	18.99	JAC33,JAC75,JAC47,JAC45,JAC64,JAC103,JAC74C,JAC101,JAC17,JAC76
Fiehn VocBinbase Bin #1210	Low	18.99	JAC30L,JAC99,JAC90,JAC81,JAC75,JAC47,JAC78,JAC96,JAC86,JAC48,JAC95,JAC103,JAC35,JAC88,JAC101,JAC68,JAC110,JAC45,JAC33,JAC64,JAC74L,JAC54,JAC6,JAC76,JAC31,JAC61A,JAC122,JAC58,JAC72,JAC25,JAC102,JAC87,JAC17,JAC24,JAC55
Fiehn VocBinbase Bin #1202	Low	19.1	JAC30L,JAC99,JAC74C,JAC90,JAC75,JAC47,JAC78,JAC96,JAC86,JAC48,JAC103,JAC35,JAC101,JAC68,JAC45,JAC33,JAC64,JAC74L,JAC6,JAC76,JAC60,JAC31,JAC61A,JAC122,JAC58,JAC72,JAC102,JAC17,JAC24

Fiehn VocBinbase Bin #357	Low	19.37	JAC30L,JAC99,JAC74C,JAC90,JAC81,JAC75,JAC47,JAC78,JAC96,JAC86,JAC48,JAC103,JAC35,JAC88,JAC101,JAC68,JAC110,JAC33,JAC64,JAC74L,JAC54,JAC6,JAC76,JAC60,JAC31,JAC61A,JAC122,JAC58,JAC72,JAC102,JAC87,JAC17,JAC55
Fiehn VocBinbase Bin #363	High	19.49	JAC33,JAC60,JAC87,JAC96,JAC88,JAC55,JAC75,JAC47,JAC54,JAC31,JAC61A,JAC64,JAC74C,JAC48,JAC95,JAC25,JAC17,JAC72,JAC81,JAC30L,JAC76,JAC90,JAC74L,JAC110,JAC35
Fiehn VocBinbase Bin #1345	High	19.73	JAC33,JAC60,JAC102,JAC96,JAC99,JAC75,JAC47,JAC31,JAC45,JAC64,JAC103,JAC74C,JAC48,JAC95,JAC25,JAC101,JAC122,JAC17,JAC78,JAC72,JAC81,JAC30L,JAC76,JAC58,JAC86,JAC110,JAC24
Fiehn VocBinbase Bin #1266	Low	19.73	JAC30L,JAC90,JAC75,JAC96,JAC95,JAC35,JAC88,JAC101,JAC74L,JAC54,JAC61A,JAC25,JAC102,JAC87,JAC17,JAC24
Fiehn VocBinbase Bin #1225	High	19.87	JAC33,JAC60,JAC87,JAC102,JAC96,JAC88,JAC55,JAC99,JAC75,JAC47,JAC54,JAC31,JAC61A,JAC45,JAC64,JAC103,JAC74C,JAC48,JAC95,JAC25,JAC101,JAC122,JAC17,JAC78,JAC68,JAC72,JAC81,JAC30L,JAC76,JAC58,JAC86,JAC90,JAC74L,JAC6,JAC110,JAC24,JAC35
Fiehn VocBinbase Bin #77	High	20.14	JAC33,JAC102,JAC96,JAC55,JAC99,JAC75,JAC47,JAC54,JAC31,JAC45,JAC64,JAC103,JAC74C,JAC48,JAC95,JAC25,JAC101,JAC122,JAC17,JAC78,JAC68,JAC30L,JAC76,JAC58,JAC86,JAC90,JAC74L,JAC24
Fiehn VocBinbase Bin #737	High	20.76	JAC33,JAC87,JAC102,JAC96,JAC88,JAC55,JAC99,JAC75,JAC47,JAC54,JAC61A,JAC64,JAC103,JAC74C,JAC48,JAC95,JAC25,JAC101,JAC122,JAC17,JAC78,JAC68,JAC72,JAC81,JAC30L,JAC76,JAC58,JAC86,JAC90,JAC74L,JAC6,JAC110,JAC35

Fiehn VocBinbase Bin #1336	Low	21.31	JAC30L,JAC99,JAC74C,JAC90,JAC81,JAC75,JAC47,JAC78,JAC96,JAC86,JAC48,JAC95,JAC103,JAC35,JAC88,JAC101,JAC68,JAC110,JAC45,JAC33,JAC64,JAC74L,JAC54,JAC6,JAC76,JAC60,JAC31,JAC61A,JAC122,JAC58,JAC72,JAC25,JAC102,JAC87,JAC17,JAC24,JAC55
Fiehn VocBinbase Bin #606	High	21.62	JAC33,JAC60,JAC87,JAC88,JAC55,JAC99,JAC47,JAC54,JAC31,JAC45,JAC64,JAC103,JAC74C,JAC48,JAC95,JAC25,JAC101,JAC17,JAC78,JAC68,JAC72,JAC76,JAC58,JAC86,JAC74L,JAC6,JAC24
Fiehn VocBinbase Bin #1297	High	21.8	JAC33,JAC60,JAC88,JAC99,JAC75,JAC47,JAC45,JAC64,JAC103,JAC74C,JAC101,JAC17,JAC78,JAC68,JAC76,JAC58,JAC86,JAC24,JAC35
Fiehn VocBinbase Bin #1091	High	22.49	JAC33,JAC60,JAC102,JAC99,JAC47,JAC31,JAC45,JAC64,JAC103,JAC74C,JAC48,JAC101,JAC122,JAC17,JAC78,JAC72,JAC81,JAC30L,JAC76,JAC58,JAC86,JAC6,JAC24,JAC35
Fiehn VocBinbase Bin #749	High	23.05	JAC33,JAC60,JAC87,JAC102,JAC55,JAC99,JAC75,JAC47,JAC31,JAC45,JAC64,JAC103,JAC74C,JAC48,JAC25,JAC101,JAC17,JAC78,JAC72,JAC30L,JAC76,JAC58,JAC86,JAC74L,JAC24
Fiehn VocBinbase Bin #53	High	24	JAC33,JAC60,JAC102,JAC88,JAC99,JAC31,JAC45,JAC64,JAC103,JAC74C,JAC48,JAC101,JAC122,JAC17,JAC78,JAC68,JAC76,JAC58,JAC86,JAC90,JAC24
Fiehn VocBinbase Bin #413	High	24.14	JAC33,JAC60,JAC87,JAC102,JAC96,JAC88,JAC55,JAC99,JAC75,JAC47,JAC54,JAC31,JAC61A,JAC64,JAC103,JAC74C,JAC48,JAC95,JAC25,JAC101,JAC122,JAC17,JAC78,JAC68,JAC72,JAC81,JAC30L,JAC76,JAC58,JAC86,JAC90,JAC74L,JAC6,JAC110,JAC24,JAC35

Fiehn VocBinbase Bin #7	High	24.33	JAC33,JAC60,JAC102,JAC96,JAC88,JAC55,JAC99,JAC75,JAC47,JAC54,JAC31,JAC61A,JAC45,JAC64,JAC74C,JAC48,JAC95,JAC25,JAC101,JAC122,JAC17,JAC78,JAC72,JAC81,JAC30L,JAC76,JAC86,JAC6,JAC110,JAC24
Fiehn VocBinbase Bin #1017	Low	25.13	JAC30L,JAC99,JAC74C,JAC90,JAC81,JAC75,JAC47,JAC78,JAC96,JAC86,JAC48,JAC95,JAC103,JAC35,JAC88,JAC101,JAC68,JAC110,JAC45,JAC33,JAC64,JAC74L,JAC54,JAC6,JAC76,JAC60,JAC31,JAC61A,JAC122,JAC58,JAC72,JAC25,JAC102,JAC87,JAC17,JAC24,JAC55
Fiehn VocBinbase Bin #1512	High	25.81	JAC33,JAC60,JAC87,JAC102,JAC75,JAC47,JAC31,JAC45,JAC64,JAC103,JAC74C,JAC48,JAC95,JAC25,JAC101,JAC122,JAC17,JAC78,JAC68,JAC72,JAC81,JAC30L,JAC76,JAC58,JAC86,JAC6,JAC24
Fiehn VocBinbase Bin #361	Low	25.91	JAC30L,JAC99,JAC74C,JAC81,JAC75,JAC47,JAC78,JAC96,JAC86,JAC48,JAC95,JAC103,JAC35,JAC88,JAC101,JAC68,JAC110,JAC45,JAC33,JAC64,JAC74L,JAC54,JAC6,JAC76,JAC60,JAC31,JAC61A,JAC122,JAC58,JAC72,JAC25,JAC102,JAC87,JAC17,JAC24,JAC55
Fiehn VocBinbase Bin #1089	Low	26.58	JAC30L,JAC99,JAC74C,JAC90,JAC81,JAC75,JAC47,JAC78,JAC96,JAC86,JAC48,JAC103,JAC35,JAC101,JAC68,JAC110,JAC45,JAC33,JAC64,JAC74L,JAC54,JAC6,JAC76,JAC60,JAC31,JAC122,JAC58,JAC72,JAC25,JAC102,JAC17,JAC24,JAC55
Fiehn VocBinbase Bin #1461	High	26.75	JAC87,JAC55,JAC99,JAC61A,JAC48,JAC25,JAC101,JAC78,JAC68,JAC81,JAC30L,JAC86,JAC90,JAC74L,JAC6,JAC110,JAC24,JAC35
Fiehn VocBinbase Bin #1510	High	26.85	JAC33,JAC60,JAC87,JAC102,JAC96,JAC99,JAC47,JAC31,JAC61A,JAC45,JAC64,JAC103,JAC74C,JAC4

			8,JAC25,JAC101,JAC122,JAC17,JAC81,JAC30L,JAC76,JAC86,JAC90,JAC74L,JAC6,JAC110,JAC24
Fiehn VocBinbase Bin #1057	High	26.85	JAC33,JAC102,JAC96,JAC99,JAC54,JAC31,JAC61A,JAC45,JAC64,JAC103,JAC74C,JAC48,JAC25,JAC101,JAC17,JAC78,JAC72,JAC81,JAC30L,JAC58,JAC86,JAC90,JAC74L,JAC6,JAC24,JAC35
Fiehn VocBinbase Bin #1182	Low	27.42	JAC30L,JAC99,JAC74C,JAC90,JAC81,JAC75,JAC78,JAC96,JAC86,JAC48,JAC103,JAC35,JAC101,JAC68,JAC45,JAC33,JAC74L,JAC76,JAC31,JAC61A,JAC122,JAC58,JAC72,JAC25,JAC87
Fiehn VocBinbase Bin #766	Low	27.49	JAC75,JAC47,JAC78,JAC96,JAC86,JAC48,JAC103,JAC68,JAC45,JAC64,JAC54,JAC6,JAC76,JAC31,JAC122,JAC58,JAC72,JAC24
Fiehn VocBinbase Bin #1355	Low	27.53	JAC99,JAC74C,JAC75,JAC47,JAC78,JAC86,JAC103,JAC35,JAC101,JAC68,JAC45,JAC64,JAC74L,JAC54,JAC31,JAC58,JAC72,JAC102,JAC17
Fiehn VocBinbase Bin #839	High	28.1	JAC33,JAC60,JAC87,JAC102,JAC88,JAC55,JAC99,JAC47,JAC31,JAC61A,JAC64,JAC103,JAC48,JAC95,JAC101,JAC17,JAC81,JAC76,JAC58,JAC74L
Fiehn VocBinbase Bin #1403	High	28.1	JAC33,JAC60,JAC102,JAC96,JAC55,JAC75,JAC47,JAC31,JAC61A,JAC64,JAC74C,JAC48,JAC95,JAC122,JAC78,JAC68,JAC72,JAC30L,JAC90,JAC74L,JAC110,JAC24,JAC35
Fiehn VocBinbase Bin #729	High	28.71	JAC60,JAC102,JAC96,JAC47,JAC54,JAC45,JAC64,JAC74C,JAC101,JAC68,JAC81,JAC76,JAC86,JAC74L,JAC110,JAC35
Fiehn VocBinbase Bin #1398	Low	29.09	JAC74C,JAC90,JAC81,JAC75,JAC78,JAC86,JAC48,JAC95,JAC103,JAC35,JAC88,JAC101,JAC68,JAC45,JAC33,JAC76,JAC60,JAC61A,JAC122,JAC58,JAC72,JAC102,JAC17,JAC24,JAC55

Fiehn VocBinbase Bin #1494	High	29.26	JAC102,JAC88,JAC55,JAC99,JAC75,JAC31,JAC45,JAC103,JAC74C,JAC48,JAC101,JAC122,JAC17,JAC68,JAC30L,JAC76,JAC58,JAC86,JAC90,JAC6,JAC110
Fiehn VocBinbase Bin #899	Low	29.26	JAC90,JAC81,JAC75,JAC47,JAC78,JAC96,JAC86,JAC48,JAC103,JAC35,JAC88,JAC101,JAC68,JAC110,JAC45,JAC33,JAC64,JAC74L,JAC54,JAC6,JAC76,JAC31,JAC58,JAC87,JAC17,JAC24,JAC55
Fiehn VocBinbase Bin #1224	High	29.35	JAC33,JAC60,JAC87,JAC102,JAC96,JAC88,JAC55,JAC99,JAC75,JAC47,JAC54,JAC31,JAC61A,JAC45,JAC64,JAC103,JAC74C,JAC48,JAC95,JAC25,JAC101,JAC122,JAC17,JAC78,JAC68,JAC72,JAC81,JAC30L,JAC76,JAC58,JAC86,JAC90,JAC74L,JAC6,JAC110,JAC24,JAC35
Fiehn VocBinbase Bin #1168	High	29.58	JAC33,JAC60,JAC87,JAC102,JAC96,JAC88,JAC55,JAC99,JAC75,JAC47,JAC54,JAC31,JAC61A,JAC45,JAC64,JAC103,JAC74C,JAC48,JAC95,JAC25,JAC101,JAC122,JAC17,JAC78,JAC68,JAC72,JAC81,JAC30L,JAC76,JAC58,JAC86,JAC90,JAC74L,JAC6,JAC110,JAC24,JAC35
Fiehn VocBinbase Bin #863	High	29.76	JAC33,JAC60,JAC102,JAC96,JAC88,JAC55,JAC99,JAC47,JAC31,JAC45,JAC64,JAC103,JAC74C,JAC48,JAC95,JAC25,JAC101,JAC122,JAC17,JAC78,JAC68,JAC72,JAC76,JAC58,JAC86,JAC24,JAC35
Fiehn VocBinbase Bin #1500	Low	29.76	JAC30L,JAC74C,JAC47,JAC78,JAC96,JAC48,JAC95,JAC103,JAC35,JAC88,JAC101,JAC68,JAC110,JAC45,JAC33,JAC64,JAC74L,JAC54,JAC31,JAC61A,JAC122,JAC72,JAC25,JAC17,JAC24,JAC55
Fiehn VocBinbase Bin #95	High	30.1	JAC87,JAC96,JAC54,JAC61A,JAC95,JAC68,JAC72,JAC81,JAC90,JAC74L,JAC6,JAC110,JAC35

Fiehn VocBinbase Bin #1506	High	30.59	JAC87,JAC96,JAC88,JAC55,JAC75,JAC54,JAC61A,JAC95,JAC25,JAC122,JAC68,JAC72,JAC81,JAC30L,JAC90,JAC74L,JAC6,JAC110,JAC35
Fiehn VocBinbase Bin #1038	High	30.69	JAC87,JAC96,JAC88,JAC75,JAC54,JAC61A,JAC95,JAC25,JAC122,JAC68,JAC72,JAC30L,JAC90,JAC74L,JAC110,JAC35
Fiehn VocBinbase Bin #494	High	31.49	JAC87,JAC96,JAC88,JAC55,JAC54,JAC61A,JAC95,JAC25,JAC122,JAC68,JAC72,JAC81,JAC30L,JAC90,JAC74L,JAC6,JAC110,JAC35
Fiehn VocBinbase Bin #809	High	31.88	JAC87,JAC96,JAC88,JAC75,JAC54,JAC61A,JAC95,JAC25,JAC122,JAC72,JAC81,JAC30L,JAC90,JAC74L,JAC6,JAC110

Table S4. VOCs annotated with high confidence in the pooled culture screen using MSHub/GNPS.

Compound/VOC*	Retention time (min)	Annotated in (isolates)	Reported in plants	Reported in *Streptomyces*
**Alcohols**				
Dihydromyrcenol	4.7	JAC33,JAC60,JAC102,JAC99,JAC47,JAC45,JAC64,JAC103,JAC74C,JAC48,JAC17,JAC78,JAC76,JAC86	Yes	Yes
1-Octen-3-ol	5.46	JAC87,JAC96,JAC88,JAC55,JAC54,JAC95,JAC101,JAC122,JAC68,JAC90,JAC74L,JAC6,JAC110,JAC35	Yes	Yes
Octanol (2-)	6.35	JAC33,JAC60,JAC87,JAC102,JAC96,JAC88,JAC55,JAC99,JAC75,JAC47,JAC54,JAC31,JAC61A,JAC45,JAC64,JAC103,JAC74C,JAC48,JAC95,JAC25,JAC101,JAC122,JAC17,JAC78,JAC68,JAC72,JAC81,JAC30L,JAC76,JAC58,JAC86,JAC90,JAC74L,JAC6,JAC110,JAC24,JAC35	Yes	Yes
**6,10-Dimethyl-2-Undecanol**	6.62	JAC33,JAC60,JAC102,JAC99,JAC47,JAC31,JAC45,JAC64,JAC103,JAC74C,JAC48,JAC25,JAC101,JAC17,JAC78,JAC68,JAC72,JAC30L,JAC76,JAC58,JAC86,JAC24	Yes	No
**3-Hexanol, 3,5-Dimethyl-**	8.03	JAC33,JAC60,JAC102,JAC88,JAC55,JAC99,JAC47,JAC54,JAC31,JAC61A,JAC103,JAC74C,JAC48,JAC25,JAC101,JAC17,JAC78,JAC72,JAC30L,JAC76,JAC58,JAC86,JAC74L,JAC6,JAC110,JAC24	Yes	No
2-Decanol	8.26	JAC33,JAC87,JAC88,JAC55,JAC99,JAC61A,JAC45,JAC103,JAC74C,JAC95,JAC101,JAC122,JAC68,JAC81,JAC30L,JAC76,JAC58,JAC86,JAC6,JAC110,JAC35	Yes	Yes
Octanol (3-)	9.39	JAC33,JAC60,JAC99,JAC75,JAC47,JAC45,JAC64,JAC103,JAC74C,JAC48,JAC17,JAC78,JAC81,JAC76,JAC58,JAC86,JAC6	Yes	Yes

1-Phenylethyl Alcohol	10.29	JAC33,JAC60,JAC87,JAC102,JAC96,JAC88,JAC55,JAC99,JAC75,JAC47,JAC54,JAC31,JAC61A,JAC64,JAC103,JAC74C,JAC48,JAC95,JAC25,JAC101,JAC122,JAC17,JAC78,JAC68,JAC72,JAC81,JAC30L,JAC76,JAC58,JAC86,JAC90,JAC74L,JAC6,JAC110,JAC24,JAC35	Yes	Yes
**2-Hexanol, 2,5-Dimethyl-, (*S*)-**	10.63	JAC33,JAC60,JAC87,JAC102,JAC96,JAC88,JAC55,JAC99,JAC75,JAC47,JAC54,JAC31,JAC61A,JAC45,JAC103,JAC48,JAC95,JAC25,JAC122,JAC17,JAC78,JAC68,JAC72,JAC81,JAC30L,JAC76,JAC58,JAC90,JAC74L,JAC6,JAC110,JAC24,JAC35	Yes	No
1-Tetradecanol	11.29	JAC33,JAC60,JAC87,JAC102,JAC88,JAC55,JAC99,JAC75,JAC61A,JAC45,JAC103,JAC48,JAC95,JAC101,JAC78,JAC72,JAC81,JAC30L,JAC76,JAC58,JAC90,JAC74L,JAC110	Yes	Yes
Cis-1,2-Cyclohexanediol	11.52	JAC33,JAC60,JAC87,JAC102,JAC96,JAC88,JAC55,JAC99,JAC75,JAC47,JAC54,JAC31,JAC61A,JAC45,JAC64,JAC103,JAC74C,JAC48,JAC95,JAC25,JAC101,JAC122,JAC17,JAC78,JAC68,JAC72,JAC30L,JAC76,JAC58,JAC86,JAC90,JAC6,JAC110,JAC24,JAC35	Yes	Yes
**1,3-Propanediol, 2-Butyl-2-Ethyl-**	12.58	JAC33,JAC60,JAC87,JAC102,JAC96,JAC88,JAC55,JAC99,JAC75,JAC47,JAC54,JAC31,JAC61A,JAC45,JAC64,JAC103,JAC74C,JAC48,JAC95,JAC25,JAC101,JAC122,JAC17,JAC78,JAC68,JAC81,JAC30L,JAC76,JAC86,JAC90,JAC74L,JAC6,JAC110,JAC24,JAC35	Yes	No
1-Undecanol, 11-Bromo-	12.58	JAC33,JAC60,JAC87,JAC102,JAC96,JAC88,JAC55,JAC99,JAC75,JAC47,JAC54,JAC31,JAC61A,JAC64,JAC103,JAC74C,JAC	Yes	Yes

		48,JAC95,JAC25,JAC101,JAC122,JAC17,JAC78,JAC68,JAC72,JAC81,JAC30L,JAC76,JAC58,JAC86,JAC90,JAC74L,JAC6,JAC110,JAC35		
Hexanol	12.99	JAC60,JAC87,JAC102,JAC96,JAC88,JAC55,JAC99,JAC75,JAC47,JAC54,JAC31,JAC61A,JAC45,JAC103,JAC48,JAC95,JAC25,JAC101,JAC122,JAC17,JAC78,JAC68,JAC72,JAC81,JAC30L,JAC76,JAC58,JAC90,JAC74L,JAC6,JAC110,JAC35	Yes	Yes
(*S*)-(+)-3-Methyl-1-Pentanol	13.09	JAC33,JAC60,JAC102,JAC99,JAC75,JAC47,JAC54,JAC31,JAC45,JAC64,JAC103,JAC74C,JAC48,JAC25,JAC101,JAC17,JAC78,JAC72,JAC81,JAC30L,JAC76,JAC58,JAC86,JAC24,JAC35	Yes	Yes
2-Isopropyl-5-Methyl-1-Heptanol	13.66	JAC33,JAC60,JAC87,JAC102,JAC88,JAC99,JAC75,JAC47,JAC54,JAC31,JAC45,JAC64,JAC74C,JAC95,JAC25,JAC101,JAC17,JAC78,JAC72,JAC76,JAC58,JAC86,JAC24,JAC35	Yes	Yes
Tetrahydrolavandulol	15.41	JAC60,JAC87,JAC102,JAC88,JAC55,JAC99,JAC75,JAC47,JAC31,JAC64,JAC103,JAC74C,JAC48,JAC25,JAC101,JAC122,JAC68,JAC72,JAC81,JAC76,JAC58,JAC86,JAC6,JAC35	Yes	Yes
**10-Undecen-1-ol**	17.86	JAC33,JAC87,JAC102,JAC96,JAC88,JAC55,JAC99,JAC75,JAC47,JAC54,JAC31,JAC61A,JAC64,JAC103,JAC74C,JAC48,JAC95,JAC122,JAC17,JAC78,JAC68,JAC72,JAC81,JAC30L,JAC76,JAC58,JAC86,JAC90,JAC74L,JAC6,JAC110,JAC24,JAC35	Yes	No
**12-Methyl-1-Tridecanol**	17.91	JAC33,JAC60,JAC87,JAC102,JAC96,JAC88,JAC55,JAC99,JAC75,JAC47,JAC54,JAC31,JAC61A,JAC45,JAC64,JAC103,JAC74C,JAC48,JAC95,JAC25,JAC101,JAC122,	Yes	No

		JAC17,JAC78,JAC68,JAC72,JAC81,JAC30L,JAC76,JAC58,JAC86,JAC90,JAC74L,JAC6,JAC110,JAC24,JAC35		
1-Pentadecanol	19.1	JAC33,JAC60,JAC87,JAC102,JAC96,JAC88,JAC55,JAC99,JAC75,JAC47,JAC54,JAC31,JAC61A,JAC103,JAC74C,JAC48,JAC95,JAC25,JAC122,JAC17,JAC78,JAC68,JAC72,JAC81,JAC30L,JAC58,JAC86,JAC90,JAC74L,JAC6,JAC110,JAC24,JAC35	Yes	Yes
Tridecanol	19.8	JAC33,JAC60,JAC87,JAC102,JAC96,JAC88,JAC55,JAC99,JAC75,JAC47,JAC54,JAC31,JAC61A,JAC45,JAC103,JAC74C,JAC95,JAC122,JAC17,JAC78,JAC68,JAC72,JAC81,JAC30L,JAC76,JAC58,JAC90,JAC74L,JAC6,JAC110,JAC24,JAC35	Yes	Yes
3-Pentadecanol	22.14	JAC87,JAC102,JAC96,JAC88,JAC55,JAC75,JAC54,JAC31,JAC61A,JAC74C,JAC101,JAC68,JAC72,JAC81,JAC30L,JAC58,JAC86,JAC90,JAC74L,JAC110,JAC24,JAC35	Yes	Yes
**10-Methyl-1-Dodecanol**	22.87	JAC33,JAC60,JAC87,JAC102,JAC96,JAC88,JAC55,JAC99,JAC75,JAC47,JAC54,JAC31,JAC61A,JAC64,JAC103,JAC74C,JAC48,JAC95,JAC25,JAC101,JAC122,JAC17,JAC78,JAC68,JAC72,JAC81,JAC30L,JAC76,JAC58,JAC86,JAC90,JAC74L,JAC6,JAC110,JAC24,JAC35	No	No
**Aldehydes**				
Benzeneacetaldehyde	12.44	JAC33,JAC60,JAC87,JAC96,JAC88,JAC55,JAC99,JAC75,JAC47,JAC54,JAC61A,JAC45,JAC64,JAC103,JAC74C,JAC48,JAC95,JAC25,JAC101,JAC122,JAC68,JAC72,JAC76,JAC58,JAC86,JAC90,JAC110,JAC24,JAC35	Yes	Yes

**(*Z*)-11-Hexadecenal**	15.71	JAC60,JAC102,JAC99,JAC47,JAC31,JAC45,JAC64,JAC25,JAC101,JAC17,JAC78,JAC72,JAC81,JAC58,JAC24	No	No
Cis-9-Hexadecenal	23.94	JAC33,JAC60,JAC87,JAC102,JAC88,JAC99,JAC75,JAC47,JAC31,JAC61A,JAC45,JAC64,JAC103,JAC74C,JAC48,JAC95,JAC101,JAC122,JAC17,JAC78,JAC68,JAC72,JAC76,JAC58,JAC86,JAC6,JAC110,JAC24,JAC35	Yes	Yes
**Esters**				
**Ethyl Undecanoate**	8.54	JAC33,JAC60,JAC87,JAC102,JAC96,JAC88,JAC55,JAC99,JAC75,JAC47,JAC54,JAC31,JAC61A,JAC45,JAC64,JAC103,JAC74C,JAC48,JAC95,JAC25,JAC101,JAC122,JAC17,JAC78,JAC68,JAC72,JAC81,JAC30L,JAC76,JAC58,JAC86,JAC90,JAC74L,JAC6,JAC110,JAC24,JAC35	No	No
**Methacrylic Acid 2-Ethylhexyl Ester**	9.61	JAC60,JAC87,JAC96,JAC88,JAC55,JAC99,JAC75,JAC47,JAC54,JAC45,JAC64,JAC48,JAC95,JAC25,JAC101,JAC17,JAC78,JAC68,JAC72,JAC81,JAC76,JAC86,JAC90,JAC74L,JAC6,JAC110	No	No
**Carbonic Acid, Butyl 2-Ethylhexyl Ester**	10.15	JAC33,JAC60,JAC87,JAC102,JAC96,JAC88,JAC55,JAC99,JAC75,JAC54,JAC31,JAC61A,JAC45,JAC64,JAC103,JAC74C,JAC48,JAC95,JAC101,JAC122,JAC17,JAC78,JAC68,JAC72,JAC81,JAC30L,JAC76,JAC58,JAC86,JAC90,JAC74L,JAC6,JAC110,JAC24,JAC35	No	No
**Octadecyl 3-(3,5-Di-Tert-Butyl-4-Hydroxyphenyl)Propionate**	10.21	JAC33,JAC60,JAC87,JAC102,JAC96,JAC88,JAC55,JAC99,JAC75,JAC54,JAC31,JAC61A,JAC64,JAC103,JAC74C,JAC48,JAC95,JAC25,JAC101,JAC122,JAC17,JAC78,JAC72,JAC81,JAC76,JAC58,JAC86,JAC90,JAC74L,JAC110,JAC24,JAC35	No	No

**2-Acetoxyisobutyryl Chloride**	10.63	JAC33,JAC60,JAC87,JAC102,JAC88,JAC55,JAC99,JAC75,JAC47,JAC54,JAC31,JAC45,JAC64,JAC103,JAC74C,JAC48,JAC95,JAC25,JAC101,JAC17,JAC78,JAC68,JAC72,JAC30L,JAC76,JAC58,JAC86,JAC74L,JAC24	Yes	No
**Hexyl Acetate**	11.29	JAC33,JAC60,JAC87,JAC102,JAC96,JAC88,JAC55,JAC99,JAC75,JAC47,JAC54,JAC31,JAC61A,JAC45,JAC64,JAC103,JAC74C,JAC48,JAC95,JAC25,JAC101,JAC122,JAC17,JAC78,JAC68,JAC72,JAC81,JAC30L,JAC76,JAC58,JAC86,JAC90,JAC74L,JAC6,JAC110,JAC24,JAC35	Yes	No
**Dodecyl Formate**	11.72	JAC33,JAC60,JAC87,JAC102,JAC96,JAC88,JAC55,JAC99,JAC75,JAC47,JAC54,JAC31,JAC61A,JAC45,JAC64,JAC103,JAC48,JAC95,JAC25,JAC122,JAC17,JAC78,JAC68,JAC72,JAC81,JAC30L,JAC76,JAC58,JAC90,JAC74L,JAC6,JAC110,JAC24,JAC35	Yes	No
**Hexyl 10-Undecenoate**	11.85	JAC33,JAC60,JAC87,JAC102,JAC96,JAC88,JAC55,JAC99,JAC75,JAC47,JAC54,JAC31,JAC61A,JAC45,JAC64,JAC103,JAC74C,JAC48,JAC95,JAC25,JAC101,JAC122,JAC17,JAC78,JAC68,JAC72,JAC81,JAC30L,JAC76,JAC58,JAC86,JAC90,JAC74L,JAC6,JAC110,JAC24,JAC35	No	No
(*Z*)-12-Octadecenoic Acid Methyl Ester	13.33	JAC33,JAC60,JAC87,JAC102,JAC96,JAC88,JAC55,JAC99,JAC75,JAC47,JAC54,JAC31,JAC61A,JAC45,JAC64,JAC103,JAC74C,JAC48,JAC95,JAC25,JAC101,JAC122,JAC17,JAC78,JAC68,JAC72,JAC81,JAC30L,JAC76,JAC58,JAC86,JAC90,JAC74L,JAC6,JAC110,JAC24,JAC35	Yes	Yes

**Methyl Para Toluate**	13.72	JAC33,JAC102,JAC88,JAC99,JAC75,JAC47,JAC54,JAC31,JAC61A,JAC64,JAC103,JAC74C,JAC48,JAC95,JAC25,JAC101,JAC122,JAC17,JAC78,JAC81,JAC76,JAC58,JAC86,JAC74L,JAC6,JAC24	No	No
**Glutaric Acid, Dec-2-Yl Dec-4-Enyl Ester**	15.41	JAC33,JAC60,JAC87,JAC102,JAC96,JAC88,JAC55,JAC99,JAC75,JAC47,JAC54,JAC31,JAC61A,JAC45,JAC64,JAC103,JAC74C,JAC48,JAC95,JAC25,JAC101,JAC122,JAC17,JAC78,JAC68,JAC72,JAC81,JAC30L,JAC76,JAC58,JAC86,JAC90,JAC74L,JAC6,JAC110,JAC24,JAC35	No	No
**1,2-Dibutyroxy-1-Ethoxyethane**	16.85	JAC33,JAC60,JAC102,JAC99,JAC75,JAC47,JAC31,JAC45,JAC64,JAC103,JAC74C,JAC48,JAC25,JAC101,JAC17,JAC78,JAC76,JAC58,JAC86,JAC24	No	No
**2-Butenedioic Acid (*Z*)-, Monododecyl Ester**	19.06	JAC33,JAC60,JAC87,JAC102,JAC96,JAC88,JAC55,JAC99,JAC75,JAC47,JAC54,JAC31,JAC61A,JAC45,JAC64,JAC103,JAC74C,JAC48,JAC95,JAC25,JAC122,JAC17,JAC78,JAC68,JAC72,JAC81,JAC30L,JAC76,JAC58,JAC86,JAC90,JAC74L,JAC6,JAC110,JAC24	Yes	No
**Decan-1,10-Diol Dimethacrylate**	19.13	JAC33,JAC60,JAC87,JAC102,JAC96,JAC55,JAC99,JAC47,JAC54,JAC31,JAC61A,JAC45,JAC64,JAC103,JAC74C,JAC48,JAC25,JAC101,JAC122,JAC17,JAC78,JAC68,JAC81,JAC76,JAC58,JAC86,JAC90,JAC74L,JAC6,JAC110,JAC35	No	No
**Carbonic Acid, 2,2,2-Trichloroethyl 2-Ethylhexyl Ester**	19.42	JAC60,JAC87,JAC102,JAC96,JAC75,JAC47,JAC31,JAC45,JAC103,JAC74C,JAC48,JAC95,JAC25,JAC101,JAC122,JAC17,JAC78,JAC68,JAC72,JAC81,JAC30L,JAC76,JAC58,JAC86,JAC90,JAC74L,JAC6,JAC110,JAC24,JAC35	No	No

**2-Ethylhexyl Mercaptoacetate**	19.46	JAC33,JAC60,JAC102,JAC99,JAC47,JAC31,JAC45,JAC64,JAC103,JAC74C,JAC48,JAC101,JAC17,JAC78,JAC72,JAC76,JAC58,JAC86,JAC24,JAC35	No	No
Dodecanoic Acid, Methyl Ester	19.7	JAC33,JAC60,JAC87,JAC102,JAC88,JAC55,JAC99,JAC75,JAC47,JAC54,JAC31,JAC61A,JAC45,JAC64,JAC103,JAC74C,JAC48,JAC95,JAC25,JAC101,JAC17,JAC78,JAC68,JAC81,JAC30L,JAC76,JAC58,JAC86,JAC90,JAC6,JAC110,JAC24	Yes	Yes
**Cyanoacetic Acid, Nonyl Ester**	19.8	JAC33,JAC60,JAC87,JAC102,JAC96,JAC88,JAC55,JAC99,JAC75,JAC47,JAC54,JAC31,JAC61A,JAC45,JAC64,JAC103,JAC48,JAC95,JAC25,JAC122,JAC17,JAC78,JAC68,JAC72,JAC81,JAC30L,JAC76,JAC58,JAC90,JAC74L,JAC6,JAC110,JAC24,JAC35	No	No
**Carbonic Acid, Monoamide, *N*-Propyl-*N*-Butyl-, 2-Ethylhexyl Ester**	20.09	JAC87,JAC102,JAC96,JAC55,JAC75,JAC47,JAC54,JAC31,JAC61A,JAC45,JAC68,JAC81,JAC30L,JAC58,JAC86,JAC24	Yes	No
**Hexanoic Acid, 2-Hexenyl Ester, (*E*)-**	20.46	JAC33,JAC60,JAC87,JAC102,JAC96,JAC88,JAC55,JAC99,JAC47,JAC54,JAC31,JAC61A,JAC64,JAC95,JAC25,JAC101,JAC122,JAC68,JAC72,JAC30L,JAC86,JAC90,JAC74L,JAC6,JAC110,JAC24,JAC35	Yes	No
**Vinyl 2-Ethylhexanoate**	20.68	JAC33,JAC60,JAC87,JAC102,JAC96,JAC88,JAC55,JAC99,JAC75,JAC47,JAC54,JAC61A,JAC45,JAC64,JAC103,JAC74C,JAC48,JAC95,JAC25,JAC101,JAC122,JAC17,JAC78,JAC68,JAC72,JAC81,JAC30L,JAC76,JAC58,JAC86,JAC90,JAC74L,JAC6,JAC110,JAC24,JAC35	No	No
**3-Octyl Isovalerate**	21.22	JAC33,JAC60,JAC87,JAC102,JAC96,JAC88,JAC55,JAC99,JAC75,JAC47,JAC54,JA	Yes	No

		C31,JAC61A,JAC45,JAC103,JAC74C,JAC48,JAC95,JAC25,JAC101,JAC122,JAC17,JAC68,JAC72,JAC81,JAC30L,JAC76,JAC86,JAC90,JAC74L,JAC6,JAC110,JAC35		
**Lauryl Acetate**	21.41	JAC33,JAC60,JAC102,JAC96,JAC88,JAC99,JAC75,JAC47,JAC54,JAC31,JAC61A,JAC45,JAC64,JAC103,JAC48,JAC95,JAC25,JAC122,JAC17,JAC78,JAC68,JAC72,JAC81,JAC30L,JAC76,JAC58,JAC86,JAC90,JAC110,JAC24,JAC35	Yes	No
**Carbonic Acid, Isobutyl Cyclohexyl Ester**	21.91	JAC33,JAC60,JAC87,JAC102,JAC96,JAC88,JAC55,JAC99,JAC75,JAC47,JAC54,JAC31,JAC61A,JAC45,JAC64,JAC103,JAC74C,JAC48,JAC95,JAC25,JAC101,JAC122,JAC17,JAC78,JAC68,JAC72,JAC81,JAC30L,JAC76,JAC58,JAC86,JAC90,JAC74L,JAC6,JAC110,JAC24,JAC35	No	No
**Hexanoic Acid, 5-Oxo-, Ethyl Ester**	21.91	JAC60,JAC87,JAC96,JAC88,JAC55,JAC75,JAC54,JAC74C,JAC95,JAC25,JAC101,JAC122,JAC72,JAC30L,JAC90,JAC74L,JAC110,JAC24,JAC35	No	No
**13-Methyltetradecanoic Acid Methyl Ester**	22.1	JAC33,JAC87,JAC102,JAC99,JAC47,JAC31,JAC45,JAC74C,JAC78,JAC76,JAC58,JAC74L,JAC24	No	No
**Ethyl Tetradecanoate**	22.14	JAC33,JAC60,JAC87,JAC102,JAC96,JAC88,JAC55,JAC99,JAC75,JAC47,JAC54,JAC31,JAC61A,JAC45,JAC64,JAC103,JAC74C,JAC48,JAC95,JAC25,JAC101,JAC122,JAC17,JAC78,JAC68,JAC72,JAC81,JAC30L,JAC76,JAC58,JAC86,JAC90,JAC74L,JAC6,JAC110,JAC24,JAC35	No	No
Methyl Tetradecanoate	22.14	JAC33,JAC60,JAC87,JAC102,JAC96,JAC88,JAC55,JAC99,JAC75,JAC47,JAC54,JAC31,JAC61A,JAC45,JAC64,JAC103,JAC74C,JAC48,JAC95,JAC25,JAC101,JAC122,	Yes	Yes

		JAC17,JAC78,JAC68,JAC72,JAC30L,JAC76,JAC58,JAC86,JAC90,JAC6,JAC110,JAC24,JAC35		
**2-(2-Methylpiperidino)Ethyl P-Chlorobenzoate**	22.28	JAC60,JAC87,JAC102,JAC96,JAC88,JAC55,JAC75,JAC47,JAC54,JAC31,JAC61A,JAC103,JAC48,JAC95,JAC25,JAC101,JAC122,JAC78,JAC68,JAC72,JAC81,JAC58,JAC86,JAC6,JAC24,JAC35	No	No
Allyl Hexanoate	22.37	JAC33,JAC60,JAC87,JAC102,JAC96,JAC88,JAC55,JAC99,JAC75,JAC47,JAC54,JAC31,JAC61A,JAC45,JAC64,JAC103,JAC74C,JAC48,JAC95,JAC25,JAC101,JAC122,JAC17,JAC78,JAC68,JAC72,JAC81,JAC30L,JAC76,JAC58,JAC86,JAC90,JAC74L,JAC6,JAC110,JAC24,JAC35	Yes	Yes
**2-Methylvaleric Acid, 2-Ethylhexyl Ester**	22.46	JAC33,JAC60,JAC87,JAC102,JAC96,JAC88,JAC55,JAC99,JAC75,JAC47,JAC54,JAC31,JAC61A,JAC45,JAC64,JAC103,JAC74C,JAC48,JAC95,JAC25,JAC101,JAC122,JAC17,JAC78,JAC68,JAC72,JAC81,JAC30L,JAC76,JAC58,JAC86,JAC90,JAC74L,JAC6,JAC110,JAC24,JAC35	No	No
**Cis-3-Hexenyl 2-Ethylbutyrate**	22.65	JAC60,JAC87,JAC102,JAC96,JAC88,JAC55,JAC99,JAC75,JAC47,JAC54,JAC61A,JAC64,JAC103,JAC74C,JAC48,JAC95,JAC25,JAC101,JAC122,JAC17,JAC78,JAC68,JAC72,JAC81,JAC30L,JAC76,JAC58,JAC86,JAC90,JAC74L,JAC6,JAC110,JAC24,JAC35	No	No
Benzyl Benzoate	22.71	JAC33,JAC60,JAC87,JAC102,JAC96,JAC88,JAC55,JAC99,JAC75,JAC47,JAC54,JAC31,JAC61A,JAC45,JAC64,JAC103,JAC74C,JAC48,JAC95,JAC25,JAC101,JAC122,JAC17,JAC78,JAC68,JAC72,JAC81,JAC3	Yes	Yes

		0L,JAC76,JAC58,JAC86,JAC90,JAC74L,JAC6,JAC110,JAC24,JAC35		
Methyl Dodecanoate_Repeat	23.22	JAC87,JAC96,JAC55,JAC99,JAC75,JAC47,JAC54,JAC61A,JAC45,JAC64,JAC74C,JAC95,JAC122,JAC68,JAC72,JAC81,JAC30L,JAC76,JAC90,JAC74L,JAC6,JAC110,JAC35	No	Yes
**Trans-2-Hexenyl 2-Ethylbutyrate**	24.14	JAC33,JAC60,JAC87,JAC102,JAC96,JAC88,JAC55,JAC99,JAC75,JAC47,JAC54,JAC31,JAC61A,JAC45,JAC103,JAC74C,JAC48,JAC95,JAC25,JAC101,JAC122,JAC17,JAC78,JAC68,JAC72,JAC81,JAC30L,JAC76,JAC86,JAC90,JAC74L,JAC6,JAC110,JAC35	No	No
Methyl Decanoate	24.22	JAC87,JAC102,JAC55,JAC99,JAC47,JAC61A,JAC45,JAC64,JAC103,JAC74C,JAC95,JAC25,JAC122,JAC17,JAC78,JAC68,JAC72,JAC58,JAC86,JAC90,JAC74L,JAC110,JAC35	No	Yes
Methyl Palmitate	24.26	JAC33,JAC87,JAC96,JAC88,JAC55,JAC75,JAC47,JAC54,JAC61A,JAC45,JAC95,JAC25,JAC122,JAC68,JAC72,JAC81,JAC30L,JAC90,JAC74L,JAC6,JAC110,JAC35	Yes	Yes
Isopropyl Hexadecanoate	25.21	JAC33,JAC87,JAC102,JAC96,JAC88,JAC55,JAC99,JAC75,JAC47,JAC54,JAC31,JAC61A,JAC64,JAC103,JAC74C,JAC48,JAC95,JAC25,JAC101,JAC122,JAC17,JAC78,JAC68,JAC72,JAC81,JAC30L,JAC76,JAC58,JAC86,JAC90,JAC74L,JAC6,JAC110,JAC24,JAC35	Yes	Yes
**Carbonic Acid, Propargyl 2-Ethylhexyl Ester**	26.14	JAC33,JAC60,JAC87,JAC102,JAC96,JAC88,JAC55,JAC99,JAC75,JAC47,JAC54,JAC31,JAC61A,JAC45,JAC64,JAC103,JAC74C,JAC48,JAC95,JAC25,JAC101,JAC122,JAC17,JAC78,JAC68,JAC72,JAC81,JAC3	No	No

		0L,JAC76,JAC58,JAC86,JAC90,JAC74L,JAC6,JAC110,JAC24,JAC35		
**1,2-Benzenedicarboxylic Acid Bis(2-Ethylhexyl) Ester**	26.52	JAC33,JAC60,JAC87,JAC102,JAC96,JAC88,JAC55,JAC99,JAC75,JAC47,JAC54,JAC31,JAC61A,JAC45,JAC64,JAC103,JAC74C,JAC48,JAC95,JAC25,JAC101,JAC122,JAC17,JAC78,JAC68,JAC72,JAC81,JAC30L,JAC76,JAC58,JAC86,JAC90,JAC74L,JAC6,JAC110,JAC24,JAC35	Yes	No
Bis(2-Ethylhexyl) Phthalate	28.42	JAC60,JAC87,JAC102,JAC96,JAC88,JAC55,JAC99,JAC75,JAC47,JAC54,JAC31,JAC61A,JAC45,JAC64,JAC103,JAC74C,JAC48,JAC95,JAC25,JAC101,JAC122,JAC17,JAC78,JAC68,JAC72,JAC81,JAC30L,JAC76,JAC58,JAC90,JAC74L,JAC6,JAC110,JAC24	Yes	Yes
**1,2-Benzenedicarboxylic Acid Decyl Octyl Ester**	28.49	JAC60,JAC87,JAC102,JAC96,JAC88,JAC55,JAC99,JAC75,JAC47,JAC54,JAC61A,JAC45,JAC64,JAC74C,JAC48,JAC95,JAC25,JAC122,JAC17,JAC68,JAC72,JAC81,JAC30L,JAC76,JAC90,JAC74L,JAC6,JAC110,JAC24,JAC35	No	No
Hexanedioic Acid, Bis(2-Ethylhexyl) Ester	28.71	JAC87,JAC88,JAC47,JAC54,JAC31,JAC61A,JAC95,JAC25,JAC101,JAC122,JAC78,JAC81,JAC86,JAC90,JAC74L,JAC6,JAC110,JAC24,JAC35	Yes	Yes
**Succinic Acid, Monochloride 2-Ethylbutyl Ester**	29.13	JAC33,JAC60,JAC87,JAC102,JAC96,JAC88,JAC55,JAC99,JAC75,JAC47,JAC54,JAC31,JAC61A,JAC45,JAC64,JAC103,JAC74C,JAC48,JAC95,JAC25,JAC101,JAC122,JAC17,JAC78,JAC68,JAC72,JAC81,JAC30L,JAC76,JAC58,JAC86,JAC90,JAC74L,JAC6,JAC110,JAC24,JAC35	No	No
Ethyl 3-Acetoxybutyrate	29.51	JAC33,JAC60,JAC87,JAC102,JAC96,JAC88,JAC55,JAC99,JAC75,JAC47,JAC54,JA	Yes	Yes

		C31,JAC61A,JAC45,JAC64,JAC103,JAC74C,JAC48,JAC95,JAC25,JAC101,JAC122,JAC17,JAC78,JAC68,JAC72,JAC81,JAC30L,JAC76,JAC58,JAC86,JAC90,JAC74L,JAC6,JAC110,JAC24,JAC35		
**1,2-Benzenedicarboxylic Acid Hexyl Octyl Ester**	30.29	JAC60,JAC87,JAC96,JAC88,JAC55,JAC99,JAC75,JAC47,JAC54,JAC31,JAC61A,JAC64,JAC103,JAC74C,JAC48,JAC95,JAC25,JAC101,JAC122,JAC17,JAC68,JAC72,JAC81,JAC30L,JAC76,JAC58,JAC86,JAC90,JAC74L,JAC6,JAC110,JAC35	No	No
**Hydrocarbons**				
**Hexane, 3-Ethyl-**	5.2	JAC33,JAC60,JAC87,JAC102,JAC96,JAC88,JAC55,JAC99,JAC75,JAC47,JAC54,JAC31,JAC61A,JAC45,JAC64,JAC103,JAC74C,JAC48,JAC95,JAC25,JAC101,JAC122,JAC17,JAC78,JAC68,JAC72,JAC81,JAC30L,JAC76,JAC58,JAC86,JAC90,JAC74L,JAC6,JAC110,JAC24,JAC35	No	No
**Hexadecane**	12.1	JAC33,JAC99,JAC75,JAC47,JAC31,JAC45,JAC64,JAC74C,JAC48,JAC25,JAC17,JAC78,JAC68,JAC81,JAC76,JAC58,JAC86,JAC24	Yes	No
1,1,4-Trimethylcyclohexane	12.62	JAC87,JAC96,JAC88,JAC55,JAC54,JAC61A,JAC95,JAC25,JAC122,JAC68,JAC76,JAC90,JAC74L,JAC6,JAC110,JAC35	Yes	Yes
1-Decene	12.94	JAC33,JAC60,JAC87,JAC102,JAC96,JAC88,JAC55,JAC99,JAC75,JAC47,JAC54,JAC31,JAC61A,JAC45,JAC64,JAC103,JAC74C,JAC48,JAC95,JAC25,JAC101,JAC122,JAC17,JAC78,JAC68,JAC72,JAC81,JAC30L,JAC76,JAC58,JAC86,JAC74L,JAC6,JAC110,JAC24,JAC35	Yes	Yes
Cyclohexane, 1,2,3-Trimethyl-	13.22	JAC33,JAC60,JAC87,JAC102,JAC96,JAC88,JAC55,JAC99,JAC75,JAC54,JAC31,JA	Yes	Yes

		C61A,JAC45,JAC103,JAC48,JAC95,JAC25,JAC122,JAC78,JAC68,JAC72,JAC81,JAC30L,JAC76,JAC58,JAC86,JAC90,JAC74L,JAC6,JAC110,JAC24,JAC35		
**2,4,4-Trimethyl-1-Hexene**	13.57	JAC33,JAC60,JAC87,JAC102,JAC96,JAC88,JAC55,JAC99,JAC75,JAC47,JAC54,JAC31,JAC61A,JAC45,JAC64,JAC103,JAC74C,JAC48,JAC95,JAC25,JAC101,JAC122,JAC17,JAC78,JAC68,JAC72,JAC81,JAC30L,JAC76,JAC58,JAC86,JAC90,JAC74L,JAC6,JAC110,JAC35	No	No
N-Octadecane	14.56	JAC33,JAC87,JAC102,JAC96,JAC88,JAC55,JAC75,JAC54,JAC61A,JAC48,JAC95,JAC25,JAC122,JAC17,JAC78,JAC68,JAC72,JAC81,JAC30L,JAC58,JAC90,JAC74L,JAC6,JAC110,JAC35	Yes	Yes
Tetracosane	16.01	JAC33,JAC87,JAC102,JAC96,JAC88,JAC55,JAC99,JAC75,JAC47,JAC54,JAC31,JAC61A,JAC45,JAC64,JAC103,JAC74C,JAC48,JAC95,JAC25,JAC101,JAC122,JAC17,JAC68,JAC72,JAC81,JAC30L,JAC76,JAC58,JAC86,JAC90,JAC74L,JAC6,JAC110,JAC24,JAC35	Yes	Yes
**1-Hexene, 3,5,5-Trimethyl-**	16.3	JAC33,JAC60,JAC87,JAC96,JAC88,JAC55,JAC99,JAC75,JAC47,JAC54,JAC31,JAC61A,JAC64,JAC103,JAC48,JAC95,JAC25,JAC101,JAC122,JAC17,JAC78,JAC68,JAC72,JAC81,JAC30L,JAC76,JAC86,JAC90,JAC74L,JAC6,JAC110,JAC35	No	No
Pentacosane	16.81	JAC33,JAC60,JAC87,JAC102,JAC96,JAC88,JAC55,JAC99,JAC75,JAC47,JAC54,JAC31,JAC61A,JAC45,JAC64,JAC103,JAC48,JAC95,JAC25,JAC101,JAC122,JAC17,JAC78,JAC68,JAC72,JAC81,JAC30L,JAC5	Yes	Yes

		8,JAC86,JAC90,JAC74L,JAC6,JAC110,JAC24,JAC35		
**Cyclohexane, Tetradecyl-**	17.2	JAC33,JAC60,JAC87,JAC102,JAC96,JAC88,JAC55,JAC99,JAC75,JAC47,JAC54,JAC31,JAC61A,JAC64,JAC103,JAC74C,JAC48,JAC95,JAC25,JAC101,JAC122,JAC17,JAC78,JAC68,JAC72,JAC81,JAC30L,JAC76,JAC58,JAC86,JAC90,JAC74L,JAC6,JAC110,JAC24,JAC35	Yes	No
**Pentane, 2,2,3,4-Tetramethyl-**	17.57	JAC60,JAC87,JAC96,JAC88,JAC55,JAC75,JAC47,JAC54,JAC61A,JAC64,JAC103,JAC74C,JAC48,JAC95,JAC25,JAC101,JAC122,JAC17,JAC78,JAC68,JAC72,JAC81,JAC30L,JAC76,JAC86,JAC90,JAC74L,JAC6,JAC110,JAC35	Yes	No
**(6*Z*,9*Z*)-6,9-Tricosadiene**	17.86	JAC87,JAC96,JAC88,JAC55,JAC75,JAC54,JAC61A,JAC64,JAC95,JAC25,JAC101,JAC122,JAC68,JAC72,JAC81,JAC30L,JAC90,JAC74L,JAC6,JAC110,JAC35	No	No
**Cyclohexane, 1,2-Dimethyl- (Cis/Trans)**	17.91	JAC87,JAC96,JAC88,JAC55,JAC99,JAC75,JAC54,JAC61A,JAC103,JAC48,JAC95,JAC25,JAC122,JAC68,JAC81,JAC74L,JAC6,JAC110	No	No
Tridecane	18.03	JAC87,JAC96,JAC88,JAC55,JAC75,JAC61A,JAC95,JAC25,JAC122,JAC68,JAC72,JAC81,JAC30L,JAC74L,JAC6,JAC110,JAC35	Yes	Yes
1,19-Eicosadiene	18.76	JAC33,JAC60,JAC87,JAC102,JAC96,JAC88,JAC55,JAC99,JAC75,JAC47,JAC54,JAC31,JAC61A,JAC45,JAC64,JAC103,JAC74C,JAC48,JAC95,JAC25,JAC101,JAC122,JAC17,JAC78,JAC68,JAC72,JAC81,JAC30L,JAC76,JAC58,JAC86,JAC90,JAC74L,JAC6,JAC110,JAC24,JAC35	Yes	Yes

1-Heptadecene	19.3	JAC33,JAC60,JAC87,JAC102,JAC96,JAC88,JAC55,JAC99,JAC75,JAC47,JAC54,JAC31,JAC61A,JAC45,JAC64,JAC103,JAC74C,JAC48,JAC95,JAC122,JAC17,JAC78,JAC72,JAC81,JAC30L,JAC76,JAC58,JAC86,JAC90,JAC74L,JAC6,JAC110,JAC24,JAC35	Yes	Yes
**Heptylcyclohexane**	20.14	JAC33,JAC87,JAC102,JAC96,JAC88,JAC55,JAC99,JAC75,JAC47,JAC54,JAC31,JAC61A,JAC45,JAC64,JAC103,JAC74C,JAC48,JAC95,JAC25,JAC101,JAC122,JAC17,JAC78,JAC68,JAC72,JAC81,JAC30L,JAC76,JAC58,JAC86,JAC90,JAC74L,JAC6,JAC110,JAC24,JAC35	Yes	No
1-Eicosene	20.61	JAC33,JAC87,JAC96,JAC88,JAC55,JAC99,JAC75,JAC47,JAC54,JAC61A,JAC45,JAC64,JAC103,JAC74C,JAC48,JAC95,JAC25,JAC101,JAC122,JAC17,JAC68,JAC72,JAC81,JAC30L,JAC76,JAC58,JAC90,JAC74L,JAC6,JAC110,JAC35	Yes	Yes
Cyclopentadecane	21.76	JAC33,JAC87,JAC96,JAC88,JAC55,JAC99,JAC75,JAC47,JAC54,JAC31,JAC61A,JAC45,JAC103,JAC48,JAC95,JAC25,JAC122,JAC17,JAC68,JAC72,JAC81,JAC30L,JAC76,JAC58,JAC86,JAC90,JAC74L,JAC6,JAC110,JAC24,JAC35	Yes	Yes
**Cyclohexane, Undecyl-**	22.56	JAC60,JAC87,JAC96,JAC88,JAC55,JAC99,JAC75,JAC47,JAC54,JAC61A,JAC45,JAC64,JAC103,JAC74C,JAC48,JAC95,JAC25,JAC101,JAC122,JAC68,JAC72,JAC30L,JAC76,JAC90,JAC74L,JAC6,JAC110,JAC35	Yes	No
Octadecane	22.94	JAC33,JAC60,JAC87,JAC102,JAC96,JAC88,JAC55,JAC99,JAC75,JAC47,JAC54,JAC31,JAC61A,JAC45,JAC64,JAC103,JAC7	Yes	Yes

		4C,JAC48,JAC95,JAC25,JAC101,JAC122,JAC17,JAC78,JAC68,JAC72,JAC81,JAC30L,JAC76,JAC58,JAC86,JAC90,JAC74L,JAC6,JAC110,JAC24,JAC35		
Pentadecane	23.02	JAC33,JAC60,JAC87,JAC102,JAC96,JAC88,JAC55,JAC99,JAC75,JAC47,JAC54,JAC31,JAC61A,JAC64,JAC103,JAC74C,JAC48,JAC95,JAC25,JAC101,JAC122,JAC17,JAC78,JAC68,JAC72,JAC81,JAC30L,JAC76,JAC58,JAC90,JAC74L,JAC6,JAC110,JAC35	Yes	Yes
Octadecane	25.01	JAC87,JAC96,JAC88,JAC55,JAC75,JAC54,JAC61A,JAC103,JAC48,JAC101,JAC72,JAC81,JAC30L,JAC76,JAC58,JAC90,JAC74L,JAC6,JAC110,JAC24,JAC35	Yes	Yes
Nonacosane	29.22	JAC33,JAC60,JAC87,JAC102,JAC96,JAC88,JAC55,JAC99,JAC75,JAC47,JAC54,JAC31,JAC61A,JAC45,JAC64,JAC103,JAC74C,JAC48,JAC95,JAC25,JAC101,JAC122,JAC17,JAC78,JAC68,JAC72,JAC81,JAC30L,JAC76,JAC58,JAC86,JAC90,JAC74L,JAC6,JAC110,JAC24,JAC35	Yes	Yes
**Trans-2-Nonene**	30.1	JAC33,JAC60,JAC87,JAC102,JAC96,JAC88,JAC55,JAC99,JAC75,JAC54,JAC31,JAC61A,JAC64,JAC103,JAC74C,JAC48,JAC95,JAC25,JAC101,JAC122,JAC17,JAC78,JAC68,JAC72,JAC81,JAC30L,JAC76,JAC58,JAC86,JAC90,JAC74L,JAC6,JAC110,JAC24,JAC35	No	No
**Ketones**				
Pentanone (4-OH-4-Me-2-)	5.57	JAC33,JAC60,JAC87,JAC102,JAC96,JAC88,JAC55,JAC99,JAC75,JAC47,JAC54,JAC31,JAC61A,JAC45,JAC64,JAC103,JAC74C,JAC48,JAC95,JAC25,JAC101,JAC122,JAC17,JAC78,JAC68,JAC72,JAC81,JAC3	Yes	Yes

		0L,JAC76,JAC58,JAC86,JAC90,JAC74L,JAC6,JAC110,JAC24,JAC35		
2-Tridecanone	5.66	JAC33,JAC60,JAC102,JAC99,JAC75,JAC47,JAC31,JAC45,JAC64,JAC103,JAC74C,JAC48,JAC101,JAC122,JAC17,JAC78,JAC68,JAC30L,JAC76,JAC58,JAC86,JAC6,JAC24	Yes	Yes
2-Butylcyclopenanone	9.67	JAC60,JAC87,JAC102,JAC96,JAC88,JAC55,JAC99,JAC75,JAC47,JAC54,JAC31,JAC61A,JAC45,JAC64,JAC103,JAC48,JAC95,JAC25,JAC122,JAC17,JAC78,JAC68,JAC72,JAC81,JAC30L,JAC76,JAC58,JAC90,JAC74L,JAC6,JAC110,JAC24,JAC35	Yes	Yes
**2-Propyl-5,5-Dimethyl-1,3-Cyclohexanedione**	10.65	JAC33,JAC87,JAC96,JAC88,JAC55,JAC99,JAC75,JAC47,JAC54,JAC31,JAC61A,JAC64,JAC103,JAC74C,JAC48,JAC95,JAC25,JAC101,JAC122,JAC17,JAC78,JAC68,JAC72,JAC81,JAC30L,JAC76,JAC58,JAC86,JAC90,JAC74L,JAC6,JAC110,JAC24,JAC35	No	No
1,2-Cyclopentanedione, 3-Methyl-	11.15	JAC33,JAC60,JAC87,JAC96,JAC88,JAC55,JAC99,JAC75,JAC47,JAC54,JAC31,JAC61A,JAC64,JAC103,JAC74C,JAC95,JAC25,JAC101,JAC122,JAC17,JAC78,JAC68,JAC72,JAC81,JAC30L,JAC76,JAC58,JAC90,JAC74L,JAC6,JAC110,JAC24,JAC35	Yes	Yes
**2-Tert-Butylcyclohexanone**	11.57	JAC33,JAC60,JAC87,JAC102,JAC96,JAC88,JAC55,JAC99,JAC75,JAC47,JAC54,JAC31,JAC61A,JAC45,JAC64,JAC103,JAC74C,JAC48,JAC95,JAC25,JAC101,JAC122,JAC17,JAC78,JAC68,JAC72,JAC81,JAC30L,JAC76,JAC58,JAC86,JAC90,JAC74L,JAC6,JAC110,JAC24,JAC35	No	No
**Cyclohexanone, 4-(1,1-Dimethylethyl)-**	12.32	JAC33,JAC60,JAC87,JAC102,JAC96,JAC88,JAC55,JAC99,JAC75,JAC47,JAC54,JA	No	No

		C31,JAC61A,JAC45,JAC64,JAC103,JAC74C,JAC48,JAC95,JAC25,JAC101,JAC122,JAC17,JAC78,JAC68,JAC72,JAC81,JAC30L,JAC76,JAC86,JAC90,JAC74L,JAC6,JAC110,JAC24,JAC35		
**Ethanone, 2-Chloro-1-(2,4-Dimethylphenyl)-**	16.16	JAC33,JAC60,JAC87,JAC102,JAC96,JAC88,JAC55,JAC99,JAC75,JAC47,JAC54,JAC31,JAC61A,JAC45,JAC64,JAC103,JAC74C,JAC48,JAC95,JAC25,JAC101,JAC122,JAC17,JAC78,JAC68,JAC72,JAC81,JAC30L,JAC76,JAC58,JAC86,JAC90,JAC74L,JAC6,JAC110,JAC24,JAC35	No	No
**4'-Butoxyacetophenone**	17.31	JAC33,JAC60,JAC87,JAC102,JAC96,JAC88,JAC55,JAC99,JAC47,JAC54,JAC61A,JAC45,JAC64,JAC103,JAC74C,JAC48,JAC95,JAC25,JAC101,JAC122,JAC17,JAC78,JAC72,JAC81,JAC30L,JAC76,JAC58,JAC86,JAC90,JAC74L,JAC110,JAC35	No	No
Cyclododecanone	17.94	JAC33,JAC60,JAC102,JAC99,JAC47,JAC31,JAC45,JAC64,JAC103,JAC74C,JAC48,JAC25,JAC101,JAC17,JAC78,JAC72,JAC76,JAC58,JAC86,JAC24	Yes	Yes
**2-(1-Cyclohexenyl)Cyclohexanone**	18.93	JAC33,JAC60,JAC87,JAC96,JAC88,JAC55,JAC99,JAC75,JAC47,JAC54,JAC31,JAC61A,JAC45,JAC64,JAC103,JAC74C,JAC48,JAC95,JAC25,JAC101,JAC122,JAC17,JAC78,JAC68,JAC72,JAC81,JAC30L,JAC76,JAC58,JAC90,JAC74L,JAC6,JAC110,JAC24,JAC35	Yes	No
Trans-3-Nonen-2-One	20.57	JAC33,JAC60,JAC87,JAC102,JAC96,JAC88,JAC55,JAC99,JAC75,JAC47,JAC54,JAC31,JAC61A,JAC45,JAC64,JAC103,JAC74C,JAC48,JAC95,JAC25,JAC101,JAC122,JAC17,JAC78,JAC68,JAC72,JAC81,JAC3	Yes	Yes

		0L,JAC76,JAC58,JAC86,JAC90,JAC74L,JAC6,JAC110,JAC35		
**3-Methoxyheptanophenone**	22.02	JAC33,JAC60,JAC87,JAC102,JAC96,JAC88,JAC55,JAC99,JAC75,JAC47,JAC54,JAC61A,JAC64,JAC103,JAC48,JAC95,JAC25,JAC101,JAC17,JAC78,JAC68,JAC72,JAC81,JAC30L,JAC76,JAC58,JAC86,JAC90,JAC6,JAC110,JAC35	Yes	No
**5,5-Dimethyl-1,3-Cyclohexanedione**	22.02	JAC60,JAC87,JAC96,JAC88,JAC55,JAC75,JAC47,JAC54,JAC31,JAC61A,JAC45,JAC64,JAC103,JAC74C,JAC48,JAC95,JAC25,JAC101,JAC122,JAC17,JAC78,JAC68,JAC72,JAC81,JAC30L,JAC76,JAC58,JAC90,JAC74L,JAC6,JAC110,JAC35	Yes	No
**Tetradecanophenone**	23.05	JAC60,JAC87,JAC102,JAC96,JAC88,JAC55,JAC99,JAC75,JAC47,JAC54,JAC31,JAC61A,JAC45,JAC64,JAC103,JAC74C,JAC48,JAC95,JAC25,JAC101,JAC122,JAC78,JAC68,JAC72,JAC81,JAC30L,JAC76,JAC90,JAC74L,JAC6,JAC110,JAC35	No	No
**Pyrimidine, 6-Oxo-5-Acetyl-4-Hydroxy-1,6-Dihydro-**	27.4	JAC33,JAC60,JAC87,JAC102,JAC96,JAC88,JAC55,JAC99,JAC75,JAC47,JAC54,JAC45,JAC64,JAC103,JAC74C,JAC48,JAC95,JAC25,JAC101,JAC122,JAC17,JAC78,JAC72,JAC81,JAC30L,JAC58,JAC86,JAC90,JAC74L,JAC6,JAC110,JAC24,JAC35	No	No
**2-Propanone, 1,1,1-Trichloro-**	29.4	JAC33,JAC60,JAC102,JAC96,JAC88,JAC99,JAC75,JAC47,JAC31,JAC45,JAC64,JAC48,JAC95,JAC25,JAC122,JAC17,JAC78,JAC68,JAC72,JAC58,JAC86,JAC90,JAC74L,JAC6,JAC110	Yes	No
Hexanal (Si-Contam)_Adms	31.2	JAC88,JAC75,JAC61A,JAC95,JAC25,JAC68,JAC81,JAC30L,JAC90,JAC74L,JAC6,JAC35	Yes	Yes

**(4a*S*,5*S*,8a*S*)-8Abeta-Formyl-5Beta-Methyl-5Alpha-(4-Methyl-3-Pentenyl)-3,4,4A,5,6,7,8,8A-Octahydronaphthalen-1(2H)-One**	30.87	JAC96,JAC88,JAC55,JAC75,JAC54,JAC61A,JAC25,JAC122,JAC68,JAC72,JAC81,JAC30L,JAC74L,JAC6,JAC35	No	No
**Terpenes and Terpenoids**				
**2-Octene, 2,6-Dimethyl-**	7.95	JAC33,JAC60,JAC87,JAC102,JAC96,JAC88,JAC55,JAC99,JAC75,JAC47,JAC54,JAC31,JAC61A,JAC45,JAC64,JAC103,JAC74C,JAC48,JAC95,JAC25,JAC101,JAC122,JAC17,JAC78,JAC68,JAC72,JAC81,JAC30L,JAC76,JAC86,JAC90,JAC74L,JAC6,JAC110,JAC24,JAC35	Yes	No
**Perillyl Isobutyrate**	10.08	JAC33,JAC60,JAC87,JAC102,JAC96,JAC88,JAC55,JAC99,JAC75,JAC47,JAC54,JAC31,JAC61A,JAC45,JAC64,JAC103,JAC74C,JAC48,JAC95,JAC25,JAC101,JAC17,JAC78,JAC68,JAC72,JAC81,JAC30L,JAC76,JAC58,JAC86,JAC90,JAC74L,JAC6,JAC110,JAC24,JAC35	No	No
Isopulegol(Equatorial)	11.82	JAC33,JAC60,JAC87,JAC102,JAC96,JAC88,JAC55,JAC99,JAC75,JAC47,JAC54,JAC31,JAC61A,JAC45,JAC64,JAC103,JAC74C,JAC48,JAC95,JAC25,JAC101,JAC122,JAC17,JAC78,JAC68,JAC72,JAC81,JAC30L,JAC76,JAC58,JAC86,JAC90,JAC74L,JAC6,JAC110,JAC24,JAC35	Yes	Yes
**1,1-Bis(4,4-Dimethyl-2,6-Dioxocyclohexyl)Ethane**	12.48	JAC60,JAC87,JAC102,JAC96,JAC88,JAC55,JAC75,JAC47,JAC54,JAC61A,JAC45,JAC95,JAC25,JAC101,JAC122,JAC17,JAC78,JAC68,JAC72,JAC81,JAC30L,JAC58,JAC90,JAC74L,JAC6,JAC110,JAC35	No	No

Cyclohexanol, 5-Methyl-2-(1-Methylethyl)-	14.07	JAC33,JAC60,JAC87,JAC102,JAC96,JAC88,JAC55,JAC99,JAC75,JAC47,JAC54,JAC31,JAC61A,JAC45,JAC64,JAC103,JAC74C,JAC95,JAC25,JAC101,JAC122,JAC17,JAC78,JAC68,JAC72,JAC81,JAC30L,JAC76,JAC58,JAC86,JAC74L,JAC6,JAC110,JAC24,JAC35	Yes	Yes
L-Menthol	14.16	JAC60,JAC87,JAC102,JAC96,JAC88,JAC55,JAC99,JAC75,JAC47,JAC54,JAC61A,JAC45,JAC64,JAC48,JAC25,JAC122,JAC17,JAC68,JAC72,JAC81,JAC30L,JAC76,JAC86,JAC90,JAC74L,JAC6,JAC110,JAC24	Yes	Yes
**(7S)-(-)-10,10-Di-Me-5-Thia-4-Azatricyclo[5.2.1.0-3,7]Dec-3-Ene-5,5-Dioxide**	15.71	JAC33,JAC60,JAC87,JAC102,JAC96,JAC88,JAC55,JAC99,JAC75,JAC47,JAC54,JAC31,JAC45,JAC64,JAC103,JAC74C,JAC48,JAC95,JAC25,JAC101,JAC122,JAC17,JAC78,JAC72,JAC81,JAC30L,JAC76,JAC58,JAC86,JAC90,JAC74L,JAC6,JAC110,JAC24,JAC35	No	No
Ylangene (Alpha-)	17.69	JAC33,JAC60,JAC87,JAC102,JAC96,JAC88,JAC55,JAC99,JAC75,JAC47,JAC54,JAC31,JAC61A,JAC45,JAC64,JAC103,JAC74C,JAC48,JAC95,JAC25,JAC101,JAC122,JAC17,JAC78,JAC68,JAC72,JAC81,JAC30L,JAC76,JAC58,JAC86,JAC90,JAC74L,JAC6,JAC110,JAC24,JAC35	Yes	Yes
Cubebene (Alpha-)	17.77	JAC33,JAC60,JAC87,JAC102,JAC96,JAC88,JAC55,JAC99,JAC75,JAC47,JAC54,JAC31,JAC61A,JAC45,JAC64,JAC103,JAC74C,JAC48,JAC95,JAC25,JAC101,JAC122,JAC17,JAC78,JAC68,JAC72,JAC81,JAC30L,JAC76,JAC58,JAC86,JAC90,JAC74L,JAC6,JAC110,JAC24,JAC35	Yes	Yes
**Acetic Acid, 1,7,7-Trimethyl-**	18.23	JAC33,JAC60,JAC102,JAC96,JAC88,JAC55,JAC99,JAC75,JAC47,JAC31,JAC61A,J	Yes	No

Bicyclo[2.2.1]Hept-2-Yl Ester		AC45,JAC103,JAC74C,JAC48,JAC95,JAC101,JAC122,JAC17,JAC78,JAC68,JAC72,JAC30L,JAC76,JAC58,JAC86,JAC90,JAC74L,JAC6,JAC110,JAC24		
Aristolochene (4,5-Di-Epi)	18.3	JAC33,JAC60,JAC87,JAC102,JAC96,JAC88,JAC55,JAC99,JAC75,JAC47,JAC54,JAC31,JAC61A,JAC45,JAC64,JAC103,JAC48,JAC95,JAC25,JAC101,JAC122,JAC17,JAC78,JAC68,JAC72,JAC81,JAC30L,JAC76,JAC58,JAC86,JAC90,JAC74L,JAC6,JAC110,JAC24,JAC35	Yes	Yes
Sabinene	18.3	JAC87,JAC102,JAC88,JAC75,JAC47,JAC45,JAC64,JAC103,JAC48,JAC95,JAC25,JAC17,JAC72,JAC81,JAC58,JAC86,JAC90,JAC74L,JAC24	Yes	Yes
Sesquiphellandrene (Beta-)	18.51	JAC33,JAC60,JAC87,JAC102,JAC96,JAC88,JAC55,JAC99,JAC75,JAC47,JAC54,JAC31,JAC61A,JAC45,JAC64,JAC103,JAC48,JAC95,JAC25,JAC122,JAC17,JAC78,JAC68,JAC72,JAC81,JAC30L,JAC76,JAC58,JAC86,JAC90,JAC74L,JAC6,JAC110,JAC24,JAC35	Yes	Yes
(+)-Alpha-Muurolene	18.59	JAC33,JAC60,JAC87,JAC102,JAC96,JAC88,JAC55,JAC99,JAC75,JAC47,JAC54,JAC31,JAC61A,JAC45,JAC64,JAC103,JAC74C,JAC48,JAC95,JAC25,JAC101,JAC122,JAC17,JAC78,JAC68,JAC72,JAC81,JAC30L,JAC76,JAC58,JAC86,JAC90,JAC74L,JAC6,JAC110,JAC24,JAC35	Yes	Yes
**7-Isopropenyl-1,4A-Dimethyl-3,4,5,6,7,8-Hexahydro-2-Naphthalenone**	19.95	JAC96,JAC99,JAC31,JAC61A,JAC64,JAC48,JAC122,JAC68,JAC72,JAC30L,JAC90,JAC74L,JAC6,JAC24,JAC35	Yes	No
**4,10-Dimethyl-7-Isopropyl-**	21.13	JAC33,JAC60,JAC87,JAC102,JAC88,JAC55,JAC99,JAC75,JAC54,JAC64,JAC103,J	No	No

**Bicyclo(4.4.0)Deca-1,4-Diene**		AC74C,JAC95,JAC25,JAC122,JAC17,JAC78,JAC68,JAC72,JAC81,JAC76,JAC6		
Germacrone	21.18	JAC33,JAC87,JAC88,JAC55,JAC99,JAC75,JAC47,JAC54,JAC61A,JAC45,JAC64,JAC74C,JAC48,JAC95,JAC25,JAC122,JAC72,JAC81,JAC76,JAC86,JAC90,JAC74L,JAC110,JAC35	Yes	Yes
Caryophyllene (Z-)	21.27	JAC33,JAC60,JAC87,JAC102,JAC88,JAC55,JAC99,JAC75,JAC47,JAC54,JAC31,JAC61A,JAC45,JAC64,JAC103,JAC74C,JAC48,JAC95,JAC25,JAC101,JAC122,JAC17,JAC78,JAC68,JAC72,JAC81,JAC30L,JAC76,JAC58,JAC90,JAC74L,JAC6,JAC110,JAC24,JAC35	Yes	Yes
Eremophilene	21.27	JAC33,JAC60,JAC87,JAC102,JAC96,JAC88,JAC55,JAC99,JAC75,JAC47,JAC54,JAC31,JAC61A,JAC45,JAC64,JAC103,JAC74C,JAC48,JAC95,JAC25,JAC101,JAC122,JAC17,JAC78,JAC68,JAC72,JAC81,JAC30L,JAC76,JAC58,JAC86,JAC90,JAC74L,JAC6,JAC110,JAC35	Yes	Yes
Beta-Gurjunene	21.55	JAC33,JAC60,JAC87,JAC96,JAC88,JAC55,JAC99,JAC75,JAC47,JAC54,JAC31,JAC61A,JAC45,JAC64,JAC103,JAC74C,JAC48,JAC95,JAC25,JAC101,JAC122,JAC17,JAC78,JAC68,JAC72,JAC81,JAC30L,JAC76,JAC58,JAC86,JAC90,JAC74L,JAC6,JAC35	Yes	Yes
**3-Isopropyl-6,10-Dimethylundecane-2-ol**	28.79	JAC33,JAC87,JAC102,JAC96,JAC88,JAC55,JAC99,JAC75,JAC47,JAC54,JAC31,JAC61A,JAC45,JAC64,JAC103,JAC48,JAC95,JAC25,JAC122,JAC17,JAC78,JAC68,JAC72,JAC81,JAC30L,JAC76,JAC58,JAC86,JAC90,JAC74L,JAC6,JAC110,JAC24,JAC35	No	No

**Diverse functional groups**				
**Octadecyl Bromide**	4.33	JAC33,JAC60,JAC87,JAC102,JAC96,JAC88,JAC55,JAC99,JAC75,JAC54,JAC31,JAC61A,JAC64,JAC103,JAC74C,JAC48,JAC95,JAC25,JAC101,JAC122,JAC17,JAC78,JAC68,JAC72,JAC81,JAC30L,JAC76,JAC58,JAC90,JAC74L,JAC6,JAC110,JAC35	Yes	No
Dramamine	4.53	JAC33,JAC60,JAC87,JAC102,JAC96,JAC88,JAC99,JAC75,JAC47,JAC54,JAC31,JAC61A,JAC45,JAC64,JAC103,JAC74C,JAC48,JAC95,JAC25,JAC101,JAC122,JAC17,JAC78,JAC68,JAC72,JAC81,JAC30L,JAC76,JAC58,JAC86,JAC90,JAC6,JAC110,JAC24,JAC35	Yes	Yes
**Behenic Amide**	4.86	JAC33,JAC60,JAC87,JAC102,JAC96,JAC88,JAC55,JAC54,JAC31,JAC61A,JAC74C,JAC48,JAC95,JAC25,JAC101,JAC122,JAC17,JAC78,JAC68,JAC72,JAC81,JAC30L,JAC58,JAC86,JAC74L,JAC110,JAC24,JAC35	No	No
**4-Chloro-2-Aminopyrimidine**	5.33	JAC33,JAC60,JAC102,JAC96,JAC88,JAC75,JAC54,JAC61A,JAC64,JAC103,JAC95,JAC25,JAC17,JAC78,JAC68,JAC72,JAC81,JAC30L,JAC76,JAC86,JAC90,JAC74L,JAC110	Yes	No
**Pentadecafluorooctanoic Acid, Pentyl Ester**	5.33	JAC33,JAC60,JAC87,JAC102,JAC88,JAC55,JAC99,JAC47,JAC54,JAC31,JAC45,JAC64,JAC103,JAC74C,JAC48,JAC25,JAC101,JAC122,JAC17,JAC78,JAC68,JAC72,JAC81,JAC30L,JAC76,JAC58,JAC86,JAC90,JAC6,JAC110,JAC24	No	No
**Hexenyl 3-Methyl Butanoate (*3Z*-)**	5.66	JAC33,JAC60,JAC87,JAC102,JAC96,JAC88,JAC55,JAC99,JAC75,JAC54,JAC31,JAC61A,JAC45,JAC64,JAC103,JAC48,JAC9	Yes	No

		5,JAC25,JAC122,JAC78,JAC68,JAC72,JAC81,JAC30L,JAC76,JAC58,JAC86,JAC90,JAC74L,JAC6,JAC110,JAC24,JAC35		
Heptanone (2-)	5.94	JAC33,JAC60,JAC87,JAC102,JAC96,JAC88,JAC55,JAC75,JAC47,JAC54,JAC31,JAC61A,JAC45,JAC64,JAC103,JAC74C,JAC48,JAC95,JAC25,JAC17,JAC78,JAC68,JAC72,JAC81,JAC30L,JAC58,JAC86,JAC90,JAC74L,JAC6,JAC110,JAC24,JAC35	Yes	Yes
**3-Methyl-P-Anisaldehyde**	6.04	JAC33,JAC60,JAC87,JAC102,JAC96,JAC88,JAC55,JAC99,JAC75,JAC47,JAC54,JAC31,JAC61A,JAC45,JAC64,JAC103,JAC74C,JAC48,JAC95,JAC25,JAC101,JAC122,JAC17,JAC78,JAC68,JAC72,JAC81,JAC30L,JAC76,JAC58,JAC86,JAC90,JAC74L,JAC6,JAC110,JAC24,JAC35	Yes	No
(+)-*N*-Benzyl-.Alpha.-Phenethylamine	6.25	JAC33,JAC60,JAC87,JAC102,JAC96,JAC88,JAC55,JAC99,JAC75,JAC47,JAC54,JAC31,JAC61A,JAC45,JAC64,JAC103,JAC74C,JAC48,JAC95,JAC25,JAC101,JAC122,JAC17,JAC78,JAC68,JAC72,JAC81,JAC30L,JAC76,JAC58,JAC86,JAC90,JAC74L,JAC6,JAC110,JAC24,JAC35	Yes	Yes
4-Piperidinemethanamine	6.31	JAC33,JAC87,JAC102,JAC96,JAC88,JAC55,JAC99,JAC75,JAC47,JAC54,JAC31,JAC45,JAC64,JAC103,JAC74C,JAC48,JAC95,JAC25,JAC101,JAC17,JAC78,JAC68,JAC81,JAC30L,JAC76,JAC58,JAC86,JAC90,JAC74L,JAC6,JAC110,JAC24,JAC35	No	Yes
**Pentadecafluorooctanoic Acid, Isobutyl Ester**	7.11	JAC60,JAC87,JAC102,JAC96,JAC88,JAC55,JAC99,JAC75,JAC47,JAC31,JAC61A,JAC45,JAC64,JAC103,JAC74C,JAC95,JAC25,JAC101,JAC122,JAC17,JAC78,JAC72,JAC81,JAC30L,JAC76,JAC58,JAC90,JAC6,JAC110,JAC35	No	No

**6-Iodo-2-Picolin-5-ol**	7.25	JAC87,JAC96,JAC88,JAC55,JAC75,JAC54,JAC31,JAC61A,JAC103,JAC48,JAC95,JAC25,JAC122,JAC68,JAC72,JAC81,JAC30L,JAC86,JAC90,JAC74L,JAC6,JAC110,JAC24,JAC35	No	No
**2-(4-Hydroxyphenyl)Ethanoic Acid**	7.32	JAC33,JAC60,JAC102,JAC96,JAC99,JAC47,JAC31,JAC45,JAC64,JAC74C,JAC48,JAC101,JAC17,JAC78,JAC76,JAC58,JAC86,JAC24	Yes	No
**Pentadecafluorooctanoic Acid, 4-Methyl-2-Pentyl Ester**	7.4	JAC33,JAC60,JAC102,JAC88,JAC55,JAC99,JAC75,JAC54,JAC45,JAC64,JAC74C,JAC95,JAC25,JAC101,JAC122,JAC17,JAC78,JAC68,JAC72,JAC81,JAC74L,JAC110,JAC24,JAC35	No	No
**Pentadecafluorooctanoic Acid, Undec-2-En-1-Yl Ester**	7.61	JAC33,JAC60,JAC87,JAC102,JAC96,JAC88,JAC55,JAC99,JAC75,JAC47,JAC54,JAC31,JAC61A,JAC45,JAC64,JAC103,JAC74C,JAC48,JAC95,JAC25,JAC101,JAC122,JAC17,JAC78,JAC68,JAC72,JAC81,JAC30L,JAC76,JAC58,JAC90,JAC74L,JAC6,JAC110,JAC24,JAC35	No	No
**Pentadecafluorooctanoic Acid, Dodecyl Ester**	7.79	JAC33,JAC60,JAC102,JAC96,JAC88,JAC55,JAC99,JAC75,JAC47,JAC54,JAC31,JAC45,JAC64,JAC103,JAC74C,JAC48,JAC25,JAC101,JAC122,JAC17,JAC78,JAC68,JAC72,JAC81,JAC30L,JAC76,JAC58,JAC86,JAC90,JAC74L,JAC6,JAC110,JAC24,JAC35	Yes	No
**Pentadecafluorooctanoic Acid, Hexyl Ester**	7.79	JAC33,JAC87,JAC102,JAC88,JAC75,JAC47,JAC31,JAC61A,JAC45,JAC64,JAC103,JAC74C,JAC48,JAC95,JAC25,JAC17,JAC78,JAC72,JAC30L,JAC76,JAC58,JAC90,JAC24,JAC35	No	No
Levoglucosan	7.95	JAC33,JAC60,JAC87,JAC102,JAC96,JAC88,JAC55,JAC99,JAC54,JAC31,JAC61A,J	Yes	Yes

		AC45,JAC64,JAC103,JAC74C,JAC48,JAC95,JAC25,JAC101,JAC122,JAC17,JAC78,JAC68,JAC81,JAC30L,JAC76,JAC58,JAC86,JAC90,JAC74L,JAC6,JAC110,JAC24,JAC35		
Dotriacontyl Isobutyl Ether	8.36	JAC60,JAC87,JAC102,JAC96,JAC88,JAC55,JAC99,JAC75,JAC47,JAC54,JAC31,JAC61A,JAC64,JAC103,JAC74C,JAC48,JAC95,JAC101,JAC122,JAC78,JAC72,JAC81,JAC30L,JAC76,JAC58,JAC86,JAC90,JAC74L,JAC110,JAC24,JAC35	Yes	Yes
1,2-Benzenedicarboxaldehyde	8.46	JAC33,JAC60,JAC87,JAC102,JAC96,JAC88,JAC55,JAC99,JAC75,JAC47,JAC54,JAC31,JAC61A,JAC45,JAC64,JAC103,JAC74C,JAC48,JAC95,JAC25,JAC101,JAC122,JAC17,JAC78,JAC68,JAC72,JAC81,JAC30L,JAC76,JAC58,JAC86,JAC90,JAC74L,JAC6,JAC110,JAC24,JAC35	Yes	Yes
**(*Z*,*Z*)-12,15-Octadecadienoic Acid Methyl Ester**	8.74	JAC33,JAC60,JAC87,JAC102,JAC96,JAC88,JAC55,JAC99,JAC75,JAC47,JAC54,JAC31,JAC61A,JAC45,JAC64,JAC103,JAC74C,JAC48,JAC95,JAC25,JAC101,JAC122,JAC17,JAC78,JAC68,JAC72,JAC81,JAC30L,JAC76,JAC58,JAC86,JAC90,JAC74L,JAC6,JAC110,JAC24,JAC35	Yes	No
Arachidonic Acid	8.77	JAC87,JAC102,JAC96,JAC88,JAC55,JAC99,JAC75,JAC47,JAC54,JAC31,JAC45,JAC64,JAC103,JAC74C,JAC48,JAC95,JAC25,JAC122,JAC17,JAC78,JAC68,JAC72,JAC81,JAC30L,JAC76,JAC58,JAC90,JAC74L,JAC6,JAC110,JAC24	Yes	Yes
Perfluoro(Methylcyclohexane)	8.77	JAC33,JAC60,JAC87,JAC102,JAC96,JAC88,JAC55,JAC99,JAC75,JAC47,JAC54,JAC31,JAC61A,JAC64,JAC103,JAC74C,JAC48,JAC95,JAC25,JAC101,JAC122,JAC17,J	Yes	Yes

		AC78,JAC68,JAC72,JAC81,JAC30L,JAC76,JAC58,JAC90,JAC74L,JAC6,JAC110,JAC24,JAC35		
**Acetaldehyde Hexyl Isobutyl Acetal**	8.82	JAC33,JAC60,JAC87,JAC102,JAC88,JAC55,JAC99,JAC75,JAC54,JAC31,JAC61A,JAC45,JAC103,JAC48,JAC95,JAC25,JAC122,JAC17,JAC78,JAC72,JAC81,JAC30L,JAC76,JAC58,JAC86,JAC90,JAC74L,JAC6,JAC110,JAC35	No	No
3,3-Diethylglutaric Acid	9.39	JAC33,JAC60,JAC87,JAC102,JAC96,JAC88,JAC55,JAC99,JAC75,JAC47,JAC54,JAC31,JAC61A,JAC45,JAC64,JAC103,JAC48,JAC95,JAC25,JAC101,JAC122,JAC17,JAC78,JAC68,JAC72,JAC81,JAC30L,JAC76,JAC58,JAC86,JAC90,JAC74L,JAC6,JAC110,JAC24,JAC35	Yes	Yes
**Pentadecafluorooctanoic Acid, Propyl Ester**	9.5	JAC33,JAC87,JAC102,JAC96,JAC88,JAC55,JAC99,JAC75,JAC47,JAC54,JAC61A,JAC45,JAC103,JAC74C,JAC48,JAC25,JAC78,JAC72,JAC81,JAC30L,JAC76,JAC58,JAC86,JAC90,JAC74L,JAC6,JAC110,JAC24,JAC35	No	No
**Cresol (Ortho-)**	9.61	JAC33,JAC60,JAC87,JAC102,JAC96,JAC55,JAC99,JAC75,JAC47,JAC54,JAC31,JAC45,JAC64,JAC103,JAC74C,JAC48,JAC95,JAC25,JAC101,JAC122,JAC17,JAC78,JAC68,JAC81,JAC76,JAC58,JAC86,JAC74L,JAC6,JAC24	Yes	No
**4-Amino-2,2,5,5-Tetramethyl-3-Imidazoline-1-Oxyl**	10.03	JAC96,JAC88,JAC55,JAC99,JAC47,JAC54,JAC31,JAC61A,JAC64,JAC103,JAC74C,JAC95,JAC101,JAC17,JAC68,JAC76,JAC74L,JAC110,JAC24,JAC35	No	No
**Benzenemethanol, 4-(1,1-Dimethylethyl)-**	10.08	JAC33,JAC60,JAC87,JAC102,JAC96,JAC88,JAC55,JAC99,JAC75,JAC54,JAC31,JAC61A,JAC45,JAC103,JAC74C,JAC95,JAC	Yes	No

		101,JAC122,JAC17,JAC78,JAC68,JAC72,JAC81,JAC30L,JAC76,JAC58,JAC86,JAC90,JAC74L,JAC6,JAC110,JAC24,JAC35		
***N*-((Methylphenylamino)Methyl)Benzamide**	10.29	JAC33,JAC60,JAC87,JAC102,JAC96,JAC88,JAC55,JAC99,JAC75,JAC47,JAC54,JAC31,JAC61A,JAC45,JAC64,JAC103,JAC74C,JAC48,JAC95,JAC25,JAC101,JAC122,JAC17,JAC78,JAC68,JAC72,JAC81,JAC30L,JAC76,JAC58,JAC86,JAC90,JAC74L,JAC6,JAC110,JAC24,JAC35	No	No
Ethyl 3-Methyl-5-Methylpyrrole-2-Carboxylate	10.55	JAC33,JAC60,JAC87,JAC96,JAC88,JAC55,JAC99,JAC75,JAC54,JAC31,JAC61A,JAC45,JAC64,JAC74C,JAC48,JAC95,JAC25,JAC101,JAC122,JAC68,JAC72,JAC81,JAC76,JAC58,JAC86,JAC90,JAC74L,JAC110,JAC24,JAC35	Yes	Yes
Suberoyl Chloride	10.77	JAC33,JAC60,JAC87,JAC102,JAC96,JAC88,JAC55,JAC99,JAC75,JAC54,JAC31,JAC61A,JAC64,JAC95,JAC25,JAC101,JAC78,JAC68,JAC72,JAC81,JAC30L,JAC76,JAC86,JAC90,JAC74L,JAC110,JAC24,JAC35	Yes	Yes
Oleic Acid	11.05	JAC33,JAC60,JAC87,JAC102,JAC96,JAC88,JAC55,JAC99,JAC75,JAC47,JAC54,JAC31,JAC61A,JAC45,JAC64,JAC103,JAC74C,JAC48,JAC95,JAC25,JAC101,JAC122,JAC17,JAC78,JAC68,JAC72,JAC81,JAC30L,JAC76,JAC58,JAC86,JAC90,JAC74L,JAC6,JAC110,JAC24,JAC35	Yes	Yes
**1,3-Dibenzoyl-4-Oxo-2-Thioxoimidazolidine**	11.11	JAC33,JAC60,JAC87,JAC102,JAC96,JAC88,JAC55,JAC99,JAC75,JAC47,JAC54,JAC31,JAC61A,JAC45,JAC64,JAC103,JAC74C,JAC48,JAC95,JAC25,JAC101,JAC122,JAC17,JAC78,JAC68,JAC72,JAC81,JAC3	No	No

		0L,JAC76,JAC58,JAC86,JAC90,JAC74L,JAC6,JAC110,JAC24		
N-.Alpha.-Benzoyl-L-Arginine	11.11	JAC60,JAC87,JAC96,JAC88,JAC55,JAC99,JAC75,JAC47,JAC54,JAC31,JAC61A,JAC45,JAC64,JAC103,JAC74C,JAC48,JAC95,JAC25,JAC101,JAC122,JAC78,JAC68,JAC72,JAC81,JAC30L,JAC76,JAC58,JAC86,JAC90,JAC74L,JAC6,JAC110,JAC24,JAC35	Yes	Yes
**1H,1H,2H-Perfluoro-1-Octene**	11.15	JAC33,JAC60,JAC87,JAC102,JAC88,JAC55,JAC99,JAC75,JAC47,JAC54,JAC31,JAC45,JAC64,JAC103,JAC74C,JAC48,JAC95,JAC25,JAC101,JAC17,JAC78,JAC68,JAC81,JAC76,JAC58,JAC86,JAC6,JAC110,JAC24	Yes	No
Decanedioyl Dichloride	11.78	JAC102,JAC47,JAC45,JAC64,JAC103,JAC74C,JAC48,JAC25,JAC101,JAC17,JAC78,JAC68,JAC81,JAC76,JAC58,JAC86,JAC90,JAC6	Yes	Yes
**Benzeneacetic Acid, .Alpha.-Oxo-, Methyl Ester**	11.91	JAC33,JAC87,JAC96,JAC88,JAC55,JAC99,JAC75,JAC54,JAC61A,JAC45,JAC64,JAC74C,JAC48,JAC95,JAC25,JAC101,JAC122,JAC78,JAC68,JAC72,JAC81,JAC30L,JAC76,JAC90,JAC74L,JAC6,JAC110,JAC35	No	No
Benzoic Acid Mono-Tms	11.96	JAC33,JAC60,JAC87,JAC102,JAC96,JAC88,JAC55,JAC99,JAC75,JAC47,JAC54,JAC31,JAC61A,JAC45,JAC64,JAC103,JAC74C,JAC48,JAC95,JAC25,JAC101,JAC122,JAC17,JAC78,JAC68,JAC72,JAC81,JAC30L,JAC76,JAC58,JAC86,JAC90,JAC74L,JAC6,JAC110,JAC24,JAC35	Yes	Yes
**Carbonochloridic Acid, Heptyl Ester**	12.19	JAC33,JAC60,JAC87,JAC102,JAC96,JAC88,JAC55,JAC99,JAC75,JAC47,JAC54,JAC31,JAC61A,JAC45,JAC64,JAC103,JAC7	Yes	No

		4C,JAC48,JAC95,JAC25,JAC101,JAC122,JAC17,JAC78,JAC68,JAC72,JAC81,JAC30L,JAC76,JAC58,JAC86,JAC90,JAC74L,JAC6,JAC110,JAC24,JAC35		
**Benzenamine, 2,4-Dimethoxy-**	13.66	JAC33,JAC60,JAC87,JAC96,JAC99,JAC47,JAC31,JAC45,JAC64,JAC103,JAC74C,JAC48,JAC95,JAC25,JAC101,JAC122,JAC17,JAC78,JAC68,JAC81,JAC30L,JAC76,JAC58,JAC86,JAC110,JAC35	No	No
2,5-Dimethylbenzophenone	13.72	JAC33,JAC60,JAC87,JAC96,JAC88,JAC55,JAC99,JAC75,JAC47,JAC54,JAC31,JAC61A,JAC103,JAC74C,JAC48,JAC95,JAC101,JAC122,JAC68,JAC72,JAC81,JAC30L,JAC76,JAC86,JAC90,JAC74L,JAC6,JAC110	Yes	Yes
O-Decylhydroxylamine	13.9	JAC33,JAC60,JAC87,JAC102,JAC96,JAC88,JAC55,JAC99,JAC75,JAC54,JAC31,JAC61A,JAC103,JAC48,JAC95,JAC25,JAC122,JAC17,JAC78,JAC68,JAC72,JAC81,JAC30L,JAC76,JAC58,JAC86,JAC90,JAC74L,JAC6,JAC110,JAC35	Yes	Yes
Para-Bromotoluene	13.97	JAC33,JAC60,JAC87,JAC102,JAC96,JAC88,JAC55,JAC99,JAC75,JAC47,JAC54,JAC31,JAC61A,JAC45,JAC64,JAC103,JAC74C,JAC48,JAC95,JAC25,JAC101,JAC122,JAC17,JAC78,JAC68,JAC72,JAC81,JAC30L,JAC76,JAC58,JAC86,JAC90,JAC74L,JAC6,JAC110,JAC24,JAC35	Yes	Yes
**2'-Ethylpropiophenone**	14.16	JAC33,JAC60,JAC87,JAC102,JAC96,JAC88,JAC55,JAC99,JAC75,JAC47,JAC54,JAC31,JAC45,JAC64,JAC103,JAC74C,JAC48,JAC25,JAC101,JAC17,JAC78,JAC72,JAC81,JAC30L,JAC76,JAC58,JAC86,JAC6,JAC110,JAC24	Yes	No

**Diazene, Bis[4-(Hexyloxy)Phenyl]-, 1-Oxide**	14.45	JAC33,JAC60,JAC87,JAC102,JAC96,JAC88,JAC55,JAC99,JAC75,JAC54,JAC31,JAC61A,JAC45,JAC64,JAC103,JAC74C,JAC48,JAC95,JAC25,JAC101,JAC122,JAC17,JAC78,JAC68,JAC72,JAC81,JAC30L,JAC76,JAC58,JAC86,JAC90,JAC74L,JAC6,JAC110,JAC35	No	No
**Pentadecafluorooctanoic Acid, 2-Ethylhexyl Ester**	14.65	JAC33,JAC60,JAC87,JAC96,JAC88,JAC55,JAC99,JAC75,JAC54,JAC61A,JAC45,JAC103,JAC74C,JAC48,JAC95,JAC25,JAC101,JAC122,JAC17,JAC78,JAC68,JAC72,JAC81,JAC30L,JAC76,JAC58,JAC86,JAC90,JAC74L,JAC6,JAC110,JAC35	No	No
**Pentadecafluorooctanoic Acid, Heptyl Ester**	14.84	JAC33,JAC60,JAC87,JAC102,JAC96,JAC88,JAC55,JAC99,JAC75,JAC47,JAC54,JAC31,JAC61A,JAC64,JAC103,JAC74C,JAC48,JAC95,JAC25,JAC101,JAC122,JAC17,JAC78,JAC68,JAC72,JAC81,JAC30L,JAC76,JAC58,JAC86,JAC90,JAC74L,JAC6,JAC110,JAC24,JAC35	No	No
Beta-Cyclocitral	15.24	JAC33,JAC87,JAC96,JAC88,JAC55,JAC99,JAC75,JAC47,JAC54,JAC31,JAC61A,JAC45,JAC64,JAC103,JAC74C,JAC48,JAC95,JAC25,JAC101,JAC122,JAC68,JAC72,JAC81,JAC30L,JAC76,JAC86,JAC90,JAC74L,JAC6,JAC110,JAC35	Yes	Yes
**1-Phenyl-1-Nonyne**	15.5	JAC33,JAC60,JAC87,JAC96,JAC88,JAC55,JAC99,JAC47,JAC54,JAC31,JAC61A,JAC45,JAC64,JAC103,JAC74C,JAC48,JAC95,JAC25,JAC101,JAC122,JAC17,JAC78,JAC68,JAC72,JAC81,JAC30L,JAC76,JAC58,JAC86,JAC90,JAC74L,JAC6,JAC110,JAC24,JAC35	Yes	No
Allylbenzene	15.75	JAC33,JAC60,JAC87,JAC102,JAC88,JAC55,JAC99,JAC75,JAC47,JAC54,JAC31,JA	Yes	Yes

		C61A,JAC45,JAC64,JAC103,JAC74C,JAC48,JAC95,JAC25,JAC101,JAC122,JAC17,JAC78,JAC68,JAC72,JAC30L,JAC76,JAC58,JAC86,JAC6,JAC24,JAC35		
**Benzoic Acid, 2-Acetylhydrazide**	15.75	JAC60,JAC87,JAC102,JAC96,JAC88,JAC55,JAC99,JAC75,JAC54,JAC61A,JAC45,JAC64,JAC74C,JAC48,JAC95,JAC25,JAC101,JAC122,JAC78,JAC68,JAC72,JAC30L,JAC76,JAC90,JAC74L,JAC6,JAC110,JAC35	No	No
**Ethyl Acetophenone (P-)**	15.75	JAC87,JAC96,JAC88,JAC55,JAC54,JAC61A,JAC103,JAC74C,JAC95,JAC25,JAC122,JAC78,JAC68,JAC81,JAC90,JAC74L,JAC6,JAC110,JAC35	Yes	No
**Behenyl Chloride**	16.45	JAC33,JAC60,JAC87,JAC102,JAC96,JAC88,JAC55,JAC99,JAC75,JAC47,JAC54,JAC31,JAC61A,JAC45,JAC64,JAC103,JAC74C,JAC48,JAC95,JAC25,JAC101,JAC122,JAC17,JAC78,JAC68,JAC72,JAC81,JAC30L,JAC76,JAC58,JAC86,JAC90,JAC74L,JAC6,JAC110,JAC24,JAC35	Yes	No
**Nonyl Tetradecyl Ether**	16.58	JAC33,JAC60,JAC87,JAC102,JAC96,JAC88,JAC55,JAC75,JAC54,JAC31,JAC61A,JAC45,JAC64,JAC48,JAC95,JAC25,JAC122,JAC17,JAC78,JAC68,JAC81,JAC30L,JAC58,JAC90,JAC74L,JAC6,JAC110,JAC24,JAC35	No	No
**1-Bromo-8-Tetrahydropyranyloxy octane**	16.81	JAC33,JAC60,JAC87,JAC102,JAC96,JAC88,JAC55,JAC99,JAC75,JAC54,JAC31,JAC61A,JAC45,JAC64,JAC103,JAC48,JAC95,JAC25,JAC101,JAC122,JAC17,JAC78,JAC68,JAC72,JAC81,JAC30L,JAC76,JAC58,JAC86,JAC90,JAC74L,JAC6,JAC110,JAC24,JAC35	No	No

**4-Benzylaminoindole**	17.04	JAC33,JAC60,JAC87,JAC102,JAC96,JAC88,JAC55,JAC99,JAC75,JAC47,JAC54,JAC31,JAC61A,JAC45,JAC64,JAC103,JAC74C,JAC48,JAC95,JAC25,JAC101,JAC122,JAC17,JAC78,JAC68,JAC72,JAC81,JAC30L,JAC76,JAC58,JAC86,JAC90,JAC74L,JAC6,JAC110,JAC24,JAC35	Yes	No
Nonalactone (Gamma-)	17.41	JAC33,JAC60,JAC87,JAC102,JAC96,JAC88,JAC55,JAC99,JAC75,JAC54,JAC31,JAC61A,JAC64,JAC103,JAC48,JAC95,JAC25,JAC122,JAC17,JAC78,JAC68,JAC72,JAC81,JAC30L,JAC76,JAC58,JAC86,JAC90,JAC74L,JAC6,JAC110,JAC24,JAC35	Yes	Yes
**Guanidine, N,N'-Bis(2-Methylphenyl)-**	17.86	JAC33,JAC60,JAC87,JAC102,JAC96,JAC88,JAC55,JAC99,JAC75,JAC47,JAC54,JAC31,JAC61A,JAC45,JAC64,JAC103,JAC74C,JAC48,JAC95,JAC25,JAC122,JAC17,JAC78,JAC68,JAC72,JAC81,JAC30L,JAC76,JAC58,JAC86,JAC90,JAC74L,JAC6,JAC110,JAC35	No	No
**Heptane, 3-[(Ethenyloxy)Methyl]-**	18.2	JAC33,JAC60,JAC87,JAC102,JAC96,JAC88,JAC55,JAC99,JAC75,JAC47,JAC54,JAC31,JAC61A,JAC45,JAC64,JAC103,JAC74C,JAC48,JAC95,JAC25,JAC101,JAC122,JAC17,JAC78,JAC68,JAC72,JAC81,JAC30L,JAC76,JAC58,JAC86,JAC90,JAC74L,JAC6,JAC110,JAC24,JAC35	Yes	No
**(*Z*,*Z*,*Z*)-6,9,15-Octadecatrienoic Acid Methyl Ester**	18.33	JAC33,JAC87,JAC88,JAC55,JAC75,JAC54,JAC61A,JAC64,JAC103,JAC74C,JAC48,JAC95,JAC25,JAC81,JAC76,JAC58,JAC86,JAC74L,JAC110,JAC35	Yes	No
**3Beta-Acetoxy-20-Hydroxy-5Alpha-Cevan-6-One**	18.38	JAC33,JAC60,JAC87,JAC102,JAC96,JAC88,JAC55,JAC99,JAC75,JAC47,JAC54,JAC31,JAC61A,JAC45,JAC64,JAC103,JAC74C,JAC48,JAC95,JAC25,JAC101,JAC122,	No	No

		JAC17,JAC78,JAC68,JAC72,JAC81,JAC30L,JAC76,JAC58,JAC86,JAC90,JAC74L,JAC6,JAC110,JAC24,JAC35		
(*Z*,*Z*,*Z*)-9,12,15-Octadecatrienoic Acid Methyl Ester	18.59	JAC33,JAC87,JAC96,JAC88,JAC55,JAC99,JAC75,JAC47,JAC54,JAC31,JAC61A,JAC45,JAC64,JAC103,JAC74C,JAC95,JAC25,JAC122,JAC17,JAC68,JAC72,JAC81,JAC30L,JAC76,JAC58,JAC86,JAC90,JAC6,JAC110,JAC24,JAC35	Yes	Yes
**Butanal, 3-Methyl-2-Methylene-, (1-Methylethyl)Hydrazone**	19.1	JAC33,JAC87,JAC102,JAC88,JAC55,JAC99,JAC75,JAC47,JAC54,JAC31,JAC61A,JAC45,JAC64,JAC103,JAC48,JAC95,JAC25,JAC101,JAC122,JAC17,JAC78,JAC81,JAC76,JAC58,JAC86,JAC90,JAC74L,JAC110,JAC24,JAC35	No	No
1-Chloroeicosane	19.3	JAC33,JAC60,JAC102,JAC96,JAC88,JAC99,JAC75,JAC47,JAC54,JAC31,JAC61A,JAC45,JAC64,JAC103,JAC74C,JAC48,JAC95,JAC25,JAC101,JAC122,JAC17,JAC78,JAC68,JAC72,JAC81,JAC30L,JAC76,JAC58,JAC86,JAC6,JAC110,JAC24,JAC35	Yes	Yes
**Propanoic Acid, 2-Methyl-, 1-(1,1-Dimethylethyl)-2-Methyl-1,3-Propanediyl Ester**	19.46	JAC33,JAC60,JAC87,JAC102,JAC96,JAC88,JAC55,JAC99,JAC75,JAC54,JAC31,JAC61A,JAC45,JAC64,JAC103,JAC74C,JAC48,JAC95,JAC25,JAC101,JAC122,JAC17,JAC78,JAC68,JAC72,JAC81,JAC30L,JAC76,JAC58,JAC86,JAC90,JAC74L,JAC6,JAC110,JAC24,JAC35	Yes	No
**Malonic Acid, Di(4-Heptyl) Ester**	19.95	JAC33,JAC60,JAC87,JAC96,JAC88,JAC55,JAC99,JAC75,JAC47,JAC54,JAC31,JAC61A,JAC45,JAC64,JAC103,JAC74C,JAC48,JAC95,JAC25,JAC101,JAC122,JAC17,JAC78,JAC68,JAC72,JAC81,JAC30L,JAC58,JAC90,JAC74L,JAC6,JAC110	No	No

**Heptyl Tetradecyl Ether**	20.3	JAC33,JAC60,JAC87,JAC102,JAC96,JAC88,JAC55,JAC99,JAC75,JAC47,JAC54,JAC31,JAC61A,JAC45,JAC64,JAC103,JAC74C,JAC48,JAC95,JAC25,JAC101,JAC122,JAC17,JAC78,JAC68,JAC72,JAC81,JAC30L,JAC76,JAC58,JAC86,JAC90,JAC74L,JAC6,JAC110,JAC24,JAC35	Yes	No
**(*Z*,*Z*,*Z*)-6,9,12-Octadecatrienoic Acid Methyl Ester**	20.35	JAC33,JAC102,JAC99,JAC47,JAC45,JAC64,JAC103,JAC48,JAC101,JAC17,JAC72,JAC76,JAC86,JAC110,JAC24	Yes	No
**(*Z*,*Z*,*Z*,*Z*)-6,9,12,15-Octadecatetraenoic Acid Methyl Ester**	20.38	JAC60,JAC102,JAC88,JAC55,JAC75,JAC74C,JAC25,JAC101,JAC122,JAC78,JAC68,JAC81,JAC90,JAC6,JAC110,JAC35	Yes	No
4-Tert-Butylpyrocatechol	20.57	JAC60,JAC88,JAC55,JAC54,JAC61A,JAC74C,JAC48,JAC95,JAC17,JAC72,JAC74L,JAC110,JAC24	Yes	Yes
**4-(Decyloxy)Benzaldehyde**	20.87	JAC60,JAC87,JAC96,JAC88,JAC55,JAC99,JAC75,JAC47,JAC54,JAC61A,JAC45,JAC64,JAC103,JAC74C,JAC48,JAC95,JAC122,JAC68,JAC72,JAC30L,JAC76,JAC86,JAC90,JAC74L,JAC6,JAC110,JAC24,JAC35	Yes	No
**1-Chloromethyl-3,5-Bis(1,1-Dimethylethyl)Benzene**	20.98	JAC33,JAC60,JAC87,JAC102,JAC96,JAC88,JAC55,JAC99,JAC75,JAC47,JAC54,JAC31,JAC61A,JAC45,JAC64,JAC103,JAC74C,JAC48,JAC95,JAC101,JAC122,JAC17,JAC78,JAC68,JAC72,JAC81,JAC30L,JAC76,JAC58,JAC86,JAC90,JAC74L,JAC6,JAC110,JAC24,JAC35	No	No
**Methyl 3,4-O-Isopropylidene-Beta-D-Fucopyranoside**	21.06	JAC33,JAC60,JAC87,JAC96,JAC88,JAC55,JAC99,JAC75,JAC31,JAC45,JAC64,JAC103,JAC74C,JAC48,JAC95,JAC25,JAC122,JAC17,JAC78,JAC68,JAC72,JAC81,JAC76,JAC58,JAC86,JAC90,JAC74L,JAC6,JAC110,JAC24,JAC35	No	No

9,12-Octadecadienoyl Chloride, (*Z*,*Z*)-	21.34	JAC33,JAC60,JAC87,JAC88,JAC99,JAC75,JAC47,JAC54,JAC45,JAC64,JAC103,JAC95,JAC17,JAC78,JAC76,JAC86,JAC6,JAC24	Yes	Yes
**Decyl Octyl Ether**	21.41	JAC33,JAC60,JAC87,JAC102,JAC96,JAC88,JAC55,JAC99,JAC75,JAC47,JAC54,JAC61A,JAC64,JAC103,JAC48,JAC95,JAC25,JAC101,JAC122,JAC17,JAC78,JAC68,JAC72,JAC81,JAC30L,JAC76,JAC58,JAC86,JAC90,JAC74L,JAC6,JAC110,JAC24,JAC35	No	No
1,9-Dichlorononane	22.19	JAC33,JAC60,JAC87,JAC102,JAC96,JAC88,JAC55,JAC99,JAC75,JAC47,JAC54,JAC31,JAC45,JAC64,JAC103,JAC48,JAC25,JAC101,JAC122,JAC17,JAC78,JAC72,JAC30L,JAC76,JAC58,JAC86,JAC90,JAC74L,JAC6,JAC24	Yes	Yes
Cyclohexasiloxane, Dodecamethyl-	22.71	JAC60,JAC87,JAC96,JAC88,JAC55,JAC99,JAC75,JAC54,JAC61A,JAC64,JAC103,JAC74C,JAC48,JAC95,JAC25,JAC122,JAC68,JAC72,JAC81,JAC30L,JAC76,JAC86,JAC90,JAC74L,JAC6,JAC110,JAC35	Yes	Yes
**(Alpha,Alpha,Alpha-Trifluoro-Para-Tolyl)Acetic Acid**	23.89	JAC33,JAC60,JAC87,JAC96,JAC88,JAC55,JAC99,JAC75,JAC47,JAC54,JAC31,JAC61A,JAC45,JAC64,JAC103,JAC74C,JAC48,JAC95,JAC25,JAC101,JAC122,JAC17,JAC78,JAC68,JAC72,JAC81,JAC30L,JAC76,JAC86,JAC90,JAC74L,JAC6,JAC110,JAC24,JAC35	No	No
Eicosane, 1-Iodo-	24.1	JAC60,JAC87,JAC102,JAC96,JAC88,JAC55,JAC99,JAC75,JAC47,JAC54,JAC61A,JAC103,JAC48,JAC95,JAC25,JAC101,JAC122,JAC17,JAC78,JAC68,JAC72,JAC81,JAC30L,JAC76,JAC58,JAC90,JAC74L,JAC6,JAC110,JAC35	Yes	Yes

1-Adamantaneacetic Acid	24.38	JAC33,JAC60,JAC87,JAC102,JAC96,JAC88,JAC55,JAC99,JAC75,JAC47,JAC54,JAC31,JAC61A,JAC45,JAC64,JAC103,JAC74C,JAC48,JAC95,JAC25,JAC101,JAC122,JAC17,JAC78,JAC68,JAC72,JAC81,JAC30L,JAC58,JAC86,JAC90,JAC74L,JAC6,JAC110,JAC24,JAC35	Yes	Yes
Butane, 1-Iodo-3-Methyl-	24.56	JAC33,JAC60,JAC87,JAC102,JAC96,JAC55,JAC99,JAC75,JAC54,JAC31,JAC61A,JAC45,JAC64,JAC103,JAC74C,JAC48,JAC95,JAC25,JAC101,JAC122,JAC17,JAC78,JAC68,JAC72,JAC81,JAC30L,JAC76,JAC58,JAC86,JAC90,JAC74L,JAC6,JAC110,JAC24,JAC35	Yes	Yes
1-Iodooctane	24.74	JAC33,JAC60,JAC87,JAC102,JAC99,JAC75,JAC47,JAC31,JAC45,JAC64,JAC103,JAC74C,JAC48,JAC95,JAC25,JAC101,JAC122,JAC17,JAC78,JAC68,JAC30L,JAC76,JAC58,JAC86,JAC6,JAC24,JAC35	Yes	Yes
Cis-1-Chloro-9-Octadecene	24.92	JAC33,JAC60,JAC87,JAC102,JAC96,JAC88,JAC55,JAC99,JAC75,JAC47,JAC54,JAC31,JAC61A,JAC45,JAC64,JAC103,JAC74C,JAC48,JAC95,JAC25,JAC101,JAC122,JAC17,JAC78,JAC68,JAC72,JAC81,JAC30L,JAC76,JAC58,JAC86,JAC90,JAC74L,JAC6,JAC110,JAC24,JAC35	Yes	Yes
Distearyl Thiodipropionate	25.76	JAC33,JAC60,JAC87,JAC102,JAC96,JAC55,JAC99,JAC75,JAC47,JAC31,JAC45,JAC64,JAC103,JAC74C,JAC101,JAC122,JAC17,JAC78,JAC68,JAC76,JAC86,JAC90,JAC6,JAC110,JAC24,JAC35	Yes	Yes
Docosanoic Anhydride	26.67	JAC33,JAC60,JAC87,JAC102,JAC96,JAC88,JAC55,JAC99,JAC75,JAC47,JAC54,JAC31,JAC61A,JAC45,JAC64,JAC103,JAC74C,JAC48,JAC95,JAC25,JAC101,JAC122,	Yes	Yes

		JAC17,JAC78,JAC68,JAC72,JAC81,JAC30L,JAC76,JAC58,JAC86,JAC90,JAC74L,JAC6,JAC110,JAC24,JAC35		
**Docosyl Pentyl Ether**	26.8	JAC33,JAC60,JAC87,JAC102,JAC96,JAC88,JAC55,JAC99,JAC75,JAC47,JAC54,JAC31,JAC61A,JAC45,JAC64,JAC103,JAC74C,JAC48,JAC95,JAC25,JAC101,JAC122,JAC17,JAC78,JAC68,JAC72,JAC81,JAC30L,JAC76,JAC58,JAC86,JAC90,JAC74L,JAC6,JAC110,JAC24,JAC35	No	No
**Pentadecafluorooctanoic Acid, Undecyl Ester**	26.93	JAC33,JAC60,JAC87,JAC96,JAC88,JAC55,JAC99,JAC75,JAC47,JAC54,JAC31,JAC61A,JAC45,JAC64,JAC103,JAC74C,JAC48,JAC95,JAC25,JAC101,JAC122,JAC17,JAC78,JAC68,JAC72,JAC81,JAC30L,JAC76,JAC58,JAC86,JAC74L,JAC6,JAC110,JAC24,JAC35	No	No
2-Bromo Dodecane	27.71	JAC87,JAC96,JAC88,JAC55,JAC99,JAC75,JAC54,JAC61A,JAC45,JAC95,JAC25,JAC101,JAC122,JAC78,JAC68,JAC72,JAC81,JAC30L,JAC76,JAC86,JAC90,JAC74L,JAC6,JAC110,JAC35	Yes	Yes
Maltitol	28.08	JAC33,JAC60,JAC87,JAC102,JAC88,JAC55,JAC99,JAC47,JAC54,JAC31,JAC61A,JAC45,JAC64,JAC103,JAC74C,JAC48,JAC25,JAC101,JAC17,JAC78,JAC81,JAC30L,JAC76,JAC58,JAC86,JAC110,JAC24	Yes	Yes
**Cyclobutanecarboxamide, *N*-Octyl-**	28.71	JAC33,JAC88,JAC99,JAC47,JAC31,JAC64,JAC103,JAC74C,JAC48,JAC25,JAC122,JAC17,JAC72,JAC76,JAC86,JAC90,JAC6,JAC24	No	No
**2-(3-Benzoylphenyl)Propionic Acid Trimethylsilyl Ester**	29.04	JAC60,JAC87,JAC102,JAC96,JAC88,JAC99,JAC54,JAC64,JAC103,JAC95,JAC101,JAC17,JAC78,JAC68,JAC30L,JAC76,JAC90,JAC35	No	No

**Oct-3-Enoylamide, *N*-Methyl-*N*-Pentyl-**	29.42	JAC60,JAC87,JAC102,JAC96,JAC88,JAC55,JAC99,JAC75,JAC47,JAC54,JAC31,JAC61A,JAC45,JAC64,JAC74C,JAC48,JAC95,JAC25,JAC101,JAC122,JAC17,JAC78,JAC68,JAC72,JAC81,JAC30L,JAC76,JAC58,JAC86,JAC90,JAC74L,JAC6,JAC110,JAC24,JAC35	No	No
Perfluorotributylamine	30.36	JAC87,JAC96,JAC88,JAC55,JAC54,JAC61A,JAC95,JAC25,JAC122,JAC68,JAC72,JAC81,JAC30L,JAC6,JAC110,JAC35	Yes	Yes
Violuric Acid	30.72	JAC87,JAC96,JAC88,JAC55,JAC75,JAC61A,JAC95,JAC25,JAC122,JAC68,JAC72,JAC90,JAC74L,JAC6,JAC110,JAC35	Yes	Yes
**1-Octanamine, *N*-Methyl-**	31.53	JAC88,JAC55,JAC75,JAC54,JAC61A,JAC122,JAC68,JAC72,JAC81,JAC30L,JAC74L,JAC6,JAC110,JAC35	No	No
**Perfluoro(2-Methylpentane)**	31.53	JAC87,JAC96,JAC88,JAC75,JAC54,JAC61A,JAC95,JAC25,JAC122,JAC68,JAC72,JAC30L,JAC74L,JAC6,JAC110	Yes	No
Perfluoro-1,1-Dimethylcyclopentane	31.66	JAC87,JAC96,JAC88,JAC55,JAC61A,JAC95,JAC25,JAC122,JAC72,JAC81,JAC30L,JAC74L,JAC6,JAC110	Yes	Yes
**Disulfide, Di-Tert-Dodecyl**	31.77	JAC87,JAC96,JAC88,JAC55,JAC75,JAC54,JAC61A,JAC95,JAC25,JAC122,JAC68,JAC72,JAC81,JAC30L,JAC90,JAC74L,JAC6,JAC110,JAC35	Yes	No

* VOCs have not been reported from *Streptomyces* previously are highlighted in bold.

Table S5. Bacterially derived compounds annotated in pooled culture VOC analysis of the 37 *Streptomyces* isolates using the conventional method.

VOC	Retention Time (min)	Confidence in annotion	Annotated in (isolates)
**Alcohols (28)**			
3-Pentanol, 3-methyl-	4.036	Unknown	JAC99, JAC48
(*S*)-3,4-Dimethylpentanol	4.129	Unknown	JAC122
2-Buten-1-ol, 2-methyl-	4.316	Unknown	JAC74C
Cyclopentanol, 1-methyl-	4.676	Low	JAC99, JAC103, JAC76, JAC48
meso-3,4-Hexanediol	4.724	Unknown	JAC30L
(*S*)-3,4-Dimethylpentanol	4.744	Unknown	JAC87
2-Methyl-2,3-pentanediol	4.757	Unknown	JAC17
2-Hexanol	4.766	Unknown	JAC88
DL-2,3-Butanediol	4.798	High	JAC17, JAC47
DL-2,3-Butanediol	4.798	Unknown	JAC103
1-Hexanol	6.223	High	JAC60, JAC61A
4-Octanol, 2-methyl-	6.918	Unknown	JAC99
Ether, 3-butenyl propyl	7.524	Unknown	JAC6, JAC88, JAC55
1-Octen-3-ol	8.963	High	JAC74C
3-Octanol	9.342	Unknown	JAC122
(*S*)-3-Ethyl-4-methylpentanol	9.462	Unknown	JAC25, JAC88, JAC61A
Benzyl alcohol	10.29	High	JAC17, JAC58, JAC25, JAC122, JAC30L

1-Octanol	11.282	High	JAC99, JAC101, JAC64
DL-4,5-Octanediol	11.475	Unknown	JAC17
1-Octanol, 2-butyl-	12.098	Unknown	JAC35
Phenylethyl Alcohol	12.432	High	JAC17, JAC31, JAC86, JAC101, JAC33, JAC58, JAC74C, JAC25, JAC110, JAC74L, JAC30L
trans-1,10-Dimethyl-trans-9-decalinol	18.385	Unknown	JAC24, JAC60, JAC58, JAC45
5,10-Pentadecadiyn-1-ol	18.969	Unknown	JAC81
1-Dodecanol	19.054	High	JAC68
5,10-Pentadecadiyn-1-ol	21.247	Unknown	JAC76, JAC90
1-Allyl-cyclohexane-1,2-diol	21.544	Unknown	JAC81
Cyclohexanol, 1-ethyl-2,2-dimethyl-6-methylene-	22.219	Unknown	JAC81
5,10-Pentadecadien-1-ol, (*Z*,*Z*)-	25.467	Unknown	JAC102
**Aldehydes (4)**			
Acetamidoacetaldehyde	7.523	Unknown	JAC110, JAC74L
(1,2,2-trimethyl-3-cyclopenten-1-yl)acetaldehyde	15.315	Unknown	JAC81
Phenylacetaldehyde N-methyl-N-formylhydrazone	16.707	Unknown	JAC25
Dodecanal	18.199	Unknown	JAC24, JAC33
**Esters (17)**			
Propanedioic acid, (bromomethyl)methyl-, bis(1,1-dimethylethyl) ester	4.019	Unknown	JAC61A

Propanoic acid, 2-methyl-, 2-ethyl-1-propyl-1,3-propanediyl ester	4.273	Unknown	JAC64
Butanoic acid, 2-methyl-, methyl ester	4.359	Unknown	JAC45, JAC47
Propanoic acid, 2,2-dimethyl-, 2,4-dinitrophenyl ester	4.361	Unknown	JAC60, JAC64
Sulfurous acid, isobutyl pentyl ester	4.485	Unknown	JAC103
Methyl 2-methoxypropenoate	4.824	Unknown	JAC30L
Propanoic acid, 2,2-dimethyl-, 2,4-dinitrophenyl ester	5.488	Unknown	JAC45
2-Butenoic acid, 2-methyl-, methyl ester	6.158	Unknown	JAC74C
Vinyl butyrate	6.751	Unknown	JAC61A
Carbonic acid, bis(1-methylethyl) ester	6.791	Unknown	JAC33
Methyl 2-methylhexanoate	8.537	High	JAC45, JAC64
Formic acid, heptyl ester	8.714	Unknown	JAC99
Propanoic acid, 3-chloro-, 4-formylphenyl ester	10.547	Unknown	JAC54
Formic acid, 2-ethylhexyl ester	11.49	Unknown	JAC99, JAC101, JAC64
6-Octen-1-ol, 3,7-dimethyl-, formate	15.123	Unknown	JAC74C
2,4-Furandicarboxylic acid, dimethyl ester	17.364	High	JAC74C
n-Heptyl methylphosphonofluoridate	18.818	Unknown	JAC78, JAC95
n-Hexyl methylphosphonofluoridate	18.841	Unknown	JAC86
Dodecanoic acid, methyl ester	19.695	High	JAC35
Benzoic acid, 4-ethoxy-, ethyl ester	19.728	Unknown	JAC88

Pentanoic acid, 2,2,4-trimethyl-3-carboxyisopropyl, isobutyl ester	20.535	High	JAC122
Hexanethioic acid, *S*-methyl ester	21.058	Unknown	JAC81
Methyl tetradecanoate	22.094	High	JAC35, JAC110, JAC74L
Hexanedioic acid, mono(2-ethylhexyl)ester	28.637	Unknown	JAC81
Phthalic acid, di(hept-3-yl) ester	30.506	Unknown	JAC81
**Hydrocarbons (30)**			
Cyclohexane, 1,1,2-trimethyl-	6.691	Low	JAC99
4-Octene, 2,3,7-trimethyl-, [*S*-(*E*)]-	9.601	Unknown	JAC25
4-Nonene, 3-methyl-, (*Z*)-	10.026	Unknown	JAC31
Cyclopropane, 1-butyl-2-(2-methylpropyl)-	10.337	Unknown	JAC87
6-Dodecene, (*E*)-	12.024	Unknown	JAC87
Undecane	12.099	High	JAC95, JAC6, JAC122
Bicyclo[3.2.0]hepta-2,6-diene	12.4	Unknown	JAC90
Cyclopropane, pentyl-	12.917	High	JAC99, JAC101
Cyclopropane, 1-butyl-2-(2-methylpropyl)-	13.103	Unknown	JAC86
1,3-Methanopentalene, octahydro-	14.053	Unknown	JAC45, JAC64
Dodecane	14.56	High	JAC95, JAC6, JAC122, JAC68
3-Hexadecene, (*Z*)-	16.299	Unknown	JAC122
1-Tridecene	16.3	Unknown	JAC6
6-Tridecene, (*Z*)-	16.301	Unknown	JAC95, JAC68
Tricyclo[4.1.0.0(2,7)]heptane	16.311	Unknown	JAC47

heptylcyclohexane	17.204	High	JAC95, JAC6, JAC122, JAC68
1,4-Dimethyladamantane	18.726	Unknown	JAC95
3-Heptyne, 5-methyl-	18.726	Unknown	JAC25, JAC81
Cyclopentane, nonyl-	18.755	Unknown	JAC95, JAC6, JAC122
4,7-Methanoazulene, 1,2,3,4,5,6,7,8-octahydro-1,4,9,9-tetramethyl-, [1*S*-(1.alpha.,4.alpha.,7.alpha.)]-	18.812	Unknown	JAC81
trans,trans- and trans,cis-1,8-Dimethylspiro[5.5]undecane	18.95	Unknown	JAC86
7-Tetradecene	19.307	Low	JAC35
6-Tridecene	19.307	Unknown	JAC88
n-Heptadecylcyclohexane	20.143	Unknown	JAC95, JAC6, JAC122, JAC68
Cetene	20.633	High	JAC47
3,4-Nonadien-6-yne, 5-ethyl-3-methyl-	21.482	Unknown	JAC81
1-Nonadecene	21.757	Low	JAC110, JAC74L
10-Heneicosene (c,t)	21.758	Unknown	JAC25
Cyclohexene, 1,5,5-trimethyl-6-(2-propenylidene)-	21.921	Unknown	JAC81
5,6-Decadien-3-yne, 5,7-diethyl-	24.274	Unknown	JAC35
**Ketones (14)**			
Cyclobutanone, 2,3-dimethyl-, trans-	4.061	Unknown	JAC88, JAC30L
2-Hexanone, 6-methoxy-	4.29	Unknown	JAC6
3-Hexanone	4.493	Low	JAC99, JAC76, JAC48, JAC6

2-Hexanone	4.523	Unknown	JAC6
2-Hydroxy-3-pentanone	4.856	Unknown	JAC88, JAC30L
3-Hexanone, 5-methyl-	5.486	Low	JAC64
(*R*)-(+)-3-Methylcyclopentanone	5.742	High	JAC6
Butanedioic acid, phenyl-	6.733	Unknown	JAC88
3-Octanone	9.038	High	JAC122
2-Hydroxy-5-ethyl-5-methylcyclopent-2-en-1-one	15.944	Unknown	JAC81
Bicyclo[3.2.0]hept-2-en-6-one, 7-chloro-	16.313	Unknown	JAC45, JAC64
2H-Benzocyclohepten-2-one, decahydro-4a-methyl-, trans-	19.147	Unknown	JAC25
Dichlorphen, O,O'-bis(4-fluoro-2-trifluoromethylbenzoyl)-	19.496	Unknown	JAC61A
Benzoic acid, 3-fluoro-, 2-oxo-2-phenylethyl ester	21.976	Unknown	JAC25
**Terpene and terpenoid (27)**			
Tricyclo[2.2.1.0(1,4)]heptan-2-one, 6-nitro-	9.615	Unknown	JAC58
2-Methyl-2-bornene	9.964	High	JAC25
Tricyclo[3.2.1.0(2,4)]octane, 8-methylene-, (1.alpha.,2.alpha.,4.alpha.,5.alpha.)-	10.075	Unknown	JAC35
2-Methylisoborneol	14.38	High	JAC102, JAC99, JAC31, JAC101, JAC60, JAC78, JAC25, JAC72, JAC55, JAC54, JAC61A, JAC90
(2*S*,4*R*)-p-Mentha-[1(7),8]-diene 2-hydroperoxide	15.292	Unknown	JAC86

Bicyclo[3.1.1]heptane, 6,6-dimethyl-2-methylene-, (1*S*)-	15.528	Unknown	JAC122
2,6-Octadienoic acid, 3,7-dimethyl-, methyl ester	16.82	High	JAC74C
1H-Benzocycloheptene, 2,4a,5,6,7,8,9,9a-octahydro-3,5,5-trimethyl-9-methylene-, (4a*S*-cis)-	18.341	High	JAC102
Geosmin	18.356	High	JAC102, JAC31, JAC86, JAC101, JAC33, JAC76, JAC47, JAC64, JAC78, JAC74C, JAC25, JAC95, JAC35, JAC88, JAC55, JAC110, JAC74L, JAC54, JAC61A, JAC75, JAC90, JAC87, JAC81
1-Methylene-2b-hydroxymethyl-3,3-dimethyl-4b-(3-methylbut-2-enyl)-cyclohexane	18.507	Unknown	JAC81
Dodecane, 2,6,10-trimethyl-	18.875	Low	JAC6, JAC68, JA95
1,6-Cyclodecadiene, 1-methyl-5-methylene-8-(1-methylethyl)-, [S-(*E*,*E*)]-	19.27	High	JAC25, JAC81
Cyclohexanemethanol, 4-ethenyl-.alpha.,.alpha.,4-trimethyl-3-(1-methylethenyl)-, [1*R*-(1.alpha.,3.alpha.,4.beta.)]-	20.162	High	JAC74C
Cyclohexanol, 4-ethyl-4-methyl-3-(1-methylethyl)-, (1.alpha.,3.alpha.,4.beta.)-	20.279	Unknown	JAC87
2,7-Octadiene-1,6-diol, 2,6-dimethyl-, (*E*)-	20.454	Unknown	JAC81
Naphthalene, 1,2,4a,5,6,8a-hexahydro-4,7-dimethyl-1-(1-methylethyl)-	20.502	Unknown	JAC25
Dihydro-cis-.alpha.-copaene-8-ol	20.549	Unknown	JAC24, JAC86, JAC95, JAC87

epi-cubenol	21.128	High	JAC25, JAC95, JAC90, JAC87
1-Naphthalenol, 1,2,3,4,4a,7,8,8a-octahydro-1,6-dimethyl-4-(1-methylethyl)-, [1*R*-(1.alpha.,4.beta.,4a.beta.,8a.beta.)]-(Cadinol)	21.182	High	JAC17, JAC102, JAC24, JAC86
Alloaromadendrene	21.265	Low	JAC88, JAC75, JAC87
Dihydro-cis-.alpha.-copaene-8-ol	21.267	Unknown	JAC95
cubenol	21.277	High	JAC47, JAC25, JAC110, JAC74L, JAC81
2-Naphthalenemethanol, decahydro-α,α,4a-trimethyl-8-methylene-, [2*R*-(2α,4aα,8aβ)]-	21.32	High	JAC86, JAC101, JAC33, JAC76, JAC74C
7-epi-.alpha.-selinene	21.544	Low	JAC95, JAC87
7-epi-.alpha.-eudesmol	21.599	Low	JAC86
10-epi-.gamma.-Eudesmol	21.655	Low	JAC31
**Diverse functional groups (43)**			
Butane, 2-azido-2,3,3-trimethyl-	4.022	Unknown	JAC72, JAC75, JAC81
t-Butyl cyclopentaneperoxycarboxylate	4.026	Unknown	JAC96
t-Butyl cyclopentaneperoxycarboxylate	4.057	Unknown	JAC96
1-n-Butoxy-2,3-dimethyldiaziridine	4.065	Unknown	JAC75
1-Heptene, 3-methoxy-	4.268	Unknown	JAC74C
Toluene	4.322	Low	JAC103
(2,3,3-Trimethyloxiranyl)methanol	4.714	Unknown	JAC88
2-Furanmethanol	5.826	Unknown	JAC30L
Hexanenitrile	6.403	High	JAC60, JAC78, JAC61A

Pyrazine, 2,5-dimethyl-	7.23	High	JAC17, JAC24, JAC31, JAC33, JAC47, JAC64, JAC78, JAC74C, JAC25, JAC35, JAC96, JAC110, JAC74L, JAC61A, JAC30L, JAC87, JAC81
1,6:3,4-Dianhydro-2-deoxy-.beta.-d-ribo-hexopyranose	8.253	Unknown	JAC88
Dimethyl trisulfide	8.636	Unknown	JAC90
3(2H)-Thiophenone, dihydro-2-methyl-	9.139	Unknown	JAC96
Ethanol, 2-(2-ethoxyethoxy)-	9.425	High	JAC17
6-Azabicyclo[3,2,0]heptan-7-one	10.079	Unknown	JAC61A
Benzoyl bromide	11.954	Unknown	JAC86, JAC76
Hydrazine, (phenylmethyl)-	12.396	Unknown	JAC55
Iron, tricarbonyl[(2,3,4,5-.eta.)-2,4-cycloheptadien-1-ol]-	13.856	Unknown	JAC45
Benzene, (iodomethyl)-	13.969	Unknown	JAC30L
Benzene, [(methylsulfonyl)methyl]-	14.023	Unknown	JAC58
Iron, tricarbonyl[(2,3,4,5-.eta.)-2,4-cycloheptadien-1-ol]-	14.818	Unknown	JAC45
Sydnone, 3-(phenylmethyl)-	14.82	Unknown	JAC64
Benzothiazole	15.082	Unknown	JAC61A
Tricyclo[3.3.1.1(3,7)]decanone, 4-iodo-, (1.alpha.,3.beta.,4.beta.,5.alpha.,7.beta.)-	15.236	Unknown	JAC25, JAC81
Phenol, 4-[2-(methylamino)ethyl]-	15.317	Unknown	JAC25
(3*R*,2*E*)-2-(Hexadec-15-ynylidene)-3-hydroxy-4-methylenebutanolide	18.654	Unknown	JAC81

Cyclobutaneacetonitrile, 1-methyl-2-(1-methylethylidene)-	18.78	Unknown	JAC86
Pyrrol-2(5H)-one, 4-acetyl-3-hydroxy-5-(3-nitrophenyl)-1-[2-(1-piperazinyl)ethyl]-	18.795	Unknown	JAC87
3-Isopropoxy-1,1,1,7,7,7-hexamethyl-3,5,5-tris(trimethylsiloxy)tetrasiloxane	18.96	Unknown	JAC87
tert-Butyl 3-methylbutyl disulfide	18.977	Unknown	JAC33
1-Hexyl-2-nitrocyclohexane	20.947	Unknown	JAC74C
β-Vatirenene	21.177	Unknown	JAC72, JAC75, JAC81
10-Methylundecan-4-olide	21.22	Low	JAC74C
10-Methyldodecan-5-olide	22.831	Low	JAC45
1-Nitrosoadamantane	24.376	Unknown	JAC35

Table S6. VOCs annotated with high confidence in the pooled culture screen using conventional method.

VOCs*	Retention time (min)	Annotated in (isolates)	Reported in plants	Reported in *Streptomyces*
**Alcohols**				
**DL-2,3-Butanediol**	4.798	JAC17, JAC47	No	No
1-Hexanol	6.223	JAC60, JAC61A	Yes	Yes
**1-Octen-3-ol**	8.963	JAC74C	Yes	No
Benzyl alcohol	10.29	JAC17, JAC58, JAC25, JAC122, JAC30L	Yes	Yes
1-Octanol	11.282	JAC99, JAC101, JAC64	Yes	Yes
Phenylethyl Alcohol	12.432	JAC17, JAC31, JAC86, JAC101, JAC33, JAC58, JAC74C, JAC25, JAC110, JAC74L, JAC30L	Yes	Yes
**1-Dodecanol**	19.054	JAC68	No	No
**Esters**				
**Methyl 2-methylhexanoate**	8.537	JAC45, JAC64	No	No
**2,4-Furandicarboxylic acid, dimethyl ester**	17.364	JAC74C	No	No
Dodecanoic acid, methyl ester	19.695	JAC35	Yes	Yes
**Pentanoic acid, 2,2,4-trimethyl-3-carboxyisopropyl, isobutyl ester**	20.535	JAC122	No	No
**Methyl tetradecanoate**	22.094	JAC35, JAC110, JAC74L	Yes	No
**Hydrocarbons**				
**Undecane**	12.099	JAC95, JAC6, JAC122	Yes	No
**Cyclopropane, pentyl-**	12.917	JAC99, JAC101	Yes	No
Dodecane	14.56	JAC95, JAC6, JAC122, JAC68	Yes	Yes
**Heptylcyclohexane**	17.204	JAC95, JAC6, JAC122, JAC68	Yes	No
**Cetene**	20.633	JAC47	No	No
**Ketones**				
**(*R*)-(+)-3-Methylcyclopentanone**	5.742	JAC6	No	No
**3-Octanone**	9.038	JAC122	No	No

**Terpenes and terpenoids**				
2-Methyl-2-bornene	9.964	JAC25	No	Yes
2-Methylisoborneol	14.38	JAC102, JAC99, JAC31, JAC101, JAC60, JAC78, JAC25, JAC72, JAC55, JAC54, JAC61A, JAC90	No	Yes
**2,6-Octadienoic acid, 3,7-dimethyl-, methyl ester**	16.82	JAC74C	No	No
1H-Benzocycloheptene, 2,4a,5,6,7,8,9,9a-octahydro-3,5,5-trimethyl-9-methylene-, (4a*S*-cis)-	18.341	JAC102	Yes	Yes
Geosmin	18.356	JAC102, JAC31, JAC86, JAC101, JAC33, JAC76, JAC47, JAC64, JAC78, JAC74C, JAC25, JAC95, JAC35, JAC88, JAC55, JAC110, JAC74L, JAC54, JAC61A, JAC75, JAC90, JAC87, JAC81	No	Yes
1,6-Cyclodecadiene, 1-methyl-5-methylene-8-(1-methylethyl)-, [*S*-(*E*,*E*)]-	19.27	JAC25, JAC81	Yes	Yes
Cyclohexanemethanol, 4-ethenyl-.alpha.,.alpha.,4-trimethyl-3-(1-methylethenyl)-, [1*R*-(1.alpha.,3.alpha.,4.beta.)]-	20.162	JAC74C	No	Yes
Epi-cubenol	21.128	JAC25, JAC95, JAC90, JAC87	No	Yes
**1-Naphthalenol, 1,2,3,4,4a,7,8,8a-octahydro-1,6-dimethyl-4-(1-methylethyl)-, [1*R*-(1.alpha.,4.beta.,4a.beta.,8a.beta.)] - (Cadinol)**	21.182	JAC17, JAC102, JAC24, JAC86	Yes	No
Cubenol	21.277	JAC47, JAC25, JAC110, JAC74L, JAC81	Yes	Yes
2-Naphthalenemethanol, decahydro-α,α,4a-trimethyl-8-methylene-, [2*R*-(2α,4aα,8aβ)]-	21.32	JAC86, JAC101, JAC33, JAC76, JAC74C	Yes	Yes
**Diverse functional groups**				

**Hexanenitrile**	6.403	JAC60, JAC78, JAC61A	No	No
**Pyrazine, 2,5-dimethyl-**	7.23	JAC17, JAC24, JAC31, JAC33, JAC47, JAC64, JAC78, JAC74C, JAC25, JAC35, JAC96, JAC110, JAC74L, JAC61A, JAC30L, JAC87, JAC81	No	No
**Ethanol, 2-(2-ethoxyethoxy)-**	9.425	JAC17	No	No

* VOCs have not been reported from *Streptomyces* previously are highlighted in bold.

Table S7. Bacterially derived compounds annotated in replicate VOC analysis of the six selceted *Streptomyces* isolates using the MSHub/GNPS. VOCs annotated during both the pooled culture screen and the replicated analysis are shown in bold and labelled with an asterisk*.

Compound/VOC	Retention time (min)	Annotated in (isolates)	Media
Benzaldehyde, 3-Methoxy-4-(Phenylmethoxy)-	4.25	JAC95, JAC25, JAC45	YMS, Synthetic, SFM
2,4-Heptanedione, 6-Methyl-	4.52	JAC25, JAC74C	SFM, YMS, Synthetic
Hexenyl Butanoate (*3Z*-)	4.62	JAC74C, JAC60, JAC81, JAC25	SFM, Synthetic, YMS
Hexenyl Isobutanoate (*3Z*-)	4.62	JAC81, JAC95, JAC25	YMS, Synthetic, SFM
**Octanol (2-)***	4.76	JAC45, JAC25	YMS, Synthetic, SFM
Tetrasiloxane, Decamethyl-	4.98	JAC74C, JAC95, JAC81	SFM, Synthetic, YMS
**Nonacosane***	5.2	JAC45, JAC60, JAC81, JAC60, JAC95	YMS, Synthetic, SFM
Triacontane	5.2	JAC60, JAC25	Synthetic, SFM, YMS
**1-Octen-3-ol***	5.42	JAC45	YMS, Synthetic, SFM
Nonane, 2,2,4,4,6,8,8-Heptamethyl-	5.48	JAC60, JAC25	Synthetic, SFM, YMS
4-Piperidinone, 2,2,6,6-Tetramethyl-	5.53	JAC60, JAC74C, JAC25, JAC95	Synthetic, SFM, YMS
**Pentanone (4-OH-4-Me-2-)***	5.53	JAC81, JAC74C	YMS, Synthetic, SFM
**Hexenyl 3-Methyl Butanoate (*3Z*-)***	5.65	JAC81, JAC95, JAC25	YMS, Synthetic, SFM
Cyclohexanone, 2,6-Dimethyl-	5.75	JAC74C, JAC25, JAC81	SFM, Synthetic, YMS
**Pentadecafluorooctanoic Acid, 2-Methylpent-3-Yl Ester***	5.93	JAC60, JAC25, JAC74C, JAC95, JAC45	Synthetic, SFM, YMS
Nonadecanoic Acid Methyl Ester	5.93	JAC95, JAC60	YMS, Synthetic
Benzenemethanamine, *N*-Phenyl-	6.03	JAC74C, JAC95, JAC25, JAC45	SFM, Synthetic, YMS
Silane, Trichlorooctadecyl-	6.1	JAC25, JAC95	SFM, YMS, Synthetic
**1-Hexanol***	6.22	JAC60, JAC95, JAC81	Synthetic, SFM, YMS
1-Ethylpentyl Acetate	6.22	JAC74C, JAC25	SFM, Synthetic, YMS
**(+)-*N*-Benzyl-.Alpha.-Phenethylamine***	6.24	JAC74C, JAC25, JAC95	SFM, Synthetic, YMS
1-Hexanethiol	6.29	JAC60, JAC95	Synthetic, SFM, YMS

N-Trimethylsilylphenylacetylglycine Trimethylsilyl Ester	6.33	JAC60	Synthetic, SFM, YMS
**Carbonic Acid, Monoamide, *N*-Hexadecyl-, Allyl Ester***	6.59	JAC45	YMS, Synthetic, SFM
Nonanol	6.62	JAC60, JAC95, JAC74C	Synthetic, SFM, YMS
2,4-Dimethylcyclopentanone	6.62	JAC74C, JAC25	SFM, Synthetic, YMS
**Hexyl Acetate***	6.62	JAC95	YMS, Synthetic,
Butanamide, N,*N*-Diethyl-3-Oxo-	6.67	JAC45	YMS, Synthetic, SFM
1H-Inden-1-One, 2,3-Dihydro-	6.73	JAC74C, JAC25	SFM, Synthetic, YMS
**Behenyl Chloride***	6.96	JAC45, JAC60, JAC81, JAC95	YMS, SFM
Ethylene Glycol Monoisobutyl Ether	7.01	JAC74C, JAC25, JAC95, JAC81	SFM, Synthetic, YMS
**2-Propyl-5,5-Dimethyl-1,3-Cyclohexanedione***	7.14	JAC74C, JAC60, JAC95, JAC81, JAC45	SFM, Synthetic, YMS
2-Butyrylfuran	7.14	JAC95, JAC45	YMS, Synthetic, SFM
**Pentadecafluorooctanoic Acid, 3-Methylbut-2-Yl Ester***	7.29	JAC74C, JAC25	SFM, Synthetic, YMS
Cyclohexanol Acetate (Trans-2-Tert-Butyl)	7.4	JAC45	YMS, Synthetic, SFM
Vinyl 10-Undecenoate	7.4	JAC74C, JAC81	SFM, Synthetic, YMS
Cyclopentadecanolide	7.4	JAC95, JAC45	YMS, Synthetic, SFM
Thujene (Alpha-)	7.78	JAC45	YMS, Synthetic, SFM
Octyl P-Toluenesulfonate	7.95	JAC74C, JAC95	SFM, Synthetic, YMS
1-Hexanol, 4-Methyl-, (*S*)-	8.02	JAC45	YMS, Synthetic, SFM
**9-Octadecenoic Acid, Methyl Ester, (*E*)-***	8.12	JAC95, JAC81, JAC45	YMS, Synthetic, SFM
((5-Isopropyl-2-Methylcyclohexyl)Sulfonylmethyl)Benzene	8.26	JAC74C, JAC25	SFM, Synthetic, YMS
**Cis-1-Chloro-9-Octadecene***	8.37	JAC45, JAC74C	Synthetic, SFM
**Oleic Anhydride***	8.38	JAC60, JAC74C, JAC25	Synthetic, SFM, YMS
N,N'-Bis(4,5-Dihydro-3-Furoyl)-M-Phenylenediamine	8.54	JAC60, JAC81	Synthetic, SFM, YMS
1-Heptanol	8.66	JAC60, JAC95, JAC45	Synthetic, SFM, YMS

Cimetidine	8.73	JAC45	YMS, Synthetic, SFM
Hexanoic Acid	8.79	JAC25	Synthetic,
**Benztropine***	8.79	JAC81	YMS, Synthetic, SFM
**10-Methyl-1-Dodecanol***	8.85	JAC25, JAC45, JAC74C, JAC60, JAC81	SFM, Synthetic, YMS
N,N’-(M-Phenylene)Dibenzamide	8.87	JAC45, JAC95	YMS, Synthetic, SFM
1-Undecanol	8.92	JAC95, JAC25,	YMS, SFM
10-Hydroxydecanoic Acid	8.93	JAC60, JAC74C	Synthetic, SFM, YMS
**7-Ethyl-2-Methylundecan-1-ol***	8.93	JAC74C, JAC25	SFM, Synthetic
Ethyl 3-(6-Methoxy-3-Methyl-2-Benzofuranyl)-3-(P-Methoxyphenyl)Propionate	9.02	JAC25, JAC81, JAC95	SFM, YMS, Synthetic
**1-Octanol***	9.04	JAC25, JAC95	SFM, YMS, Synthetic
Cyanoacetic Acid, Heptyl Ester	9.13	JAC45, JAC45	YMS, Synthetic, SFM
Heptane, 2-Chloro-	9.13	JAC60, JAC25, JAC74C, JAC95, JAC45	Synthetic, SFM, YMS
Pentyl Furan (2-)	9.18	JAC60, JAC95, JAC45, , JAC25	Synthetic, SFM, YMS
Propylene Glycol	9.21	JAC45, JAC81, JAC74C	YMS, Synthetic, SFM
**3,3-Diethylglutaric Acid***	9.4	JAC60, JAC25, JAC95, JAC81, JAC45, JAC74C	Synthetic, SFM, YMS
Tetradecane	9.47	JAC25, JAC95	SFM, YMS, Synthetic
Cetotiamine	9.47	JAC60, JAC95, JAC81	Synthetic, YMS
3-Amino-4,5,6,7,8,9-Hexahydrocyclooct(C)Isoxazole	9.5	JAC45	YMS, Synthetic, SFM
4,5-Dimethyl-2-Propyloxazole	9.62	JAC25	SFM, YMS, Synthetic
2,2,5-Trimethyl-1-Phenyl-4-Hexen-1-ol	9.62	JAC60, JAC25	Synthetic, SFM, YMS
S,S'-(Thiodi-P-Phenylene) Bis(2-Methyl-2-Propenethioate)	9.62	JAC74C, JAC45	SFM, Synthetic, YMS
**2-Butylcyclopenanone***	9.67	JAC60, JAC74C, JAC81	Synthetic, SFM, YMS
2-Piperidinoethyl 3,5-Dichlorobenzoate	9.71	JAC60, JAC45	Synthetic, SFM, YMS
Nonadienal (2*E*, 6*Z*)	9.82	JAC60	Synthetic, SFM, YMS

**1,3-Propanediol, 2-Butyl-2-Ethyl-***	9.84	JAC25, JAC45, JAC74C, JAC60, JAC95, JAC81	YMS, Synthetic,
**Methacrylic Acid 2-Ethylhexyl Ester**	9.84	JAC74C, JAC25, JAC95	SFM, Synthetic, YMS
**Pentadecafluorooctanoic Acid, Heptyl Ester***	9.84	JAC95, JAC74C, JAC25, JAC45, JAC60	YMS, Synthetic, SFM
Bulnesene (Alpha-)	9.97	JAC60, JAC45, JAC81, JAC95, JAC25	Synthetic, SFM, YMS
(*S*)-(-)-(4-Isopropenyl-1-Cyclohexenyl)Methanol	10.07	JAC60, JAC95, JAC81	Synthetic, SFM, YMS
(*S*)-(+)-5-Methyl-1-Heptanol	10.14	JAC60, JAC45, JAC74C, JAC25, JAC95	Synthetic, SFM, YMS
Geraniol	10.21	JAC25, JAC45	SFM, YMS, Synthetic
**Cresol (Ortho-)***	10.27	JAC95, JAC45, JAC25	YMS, Synthetic, SFM
**Benzyl Alcohol***	10.3	JAC60, JAC25	SFM, YMS
4-Nitrobenzyl Alcohol	10.31	JAC95	YMS, Synthetic,
N*-((Methylphenylamino)Methyl)Benzamide	10.31	JAC95	YMS, Synthetic,
**Cis-1,2-Cyclohexanediol***	10.43	JAC60, JAC74C, JAC25, JAC95, JAC81, JAC45	Synthetic, SFM, YMS
Fumaric Acid, 3-Fluorophenyl Tetradecyl Ester	10.53	JAC60, JAC25	Synthetic, SFM, YMS
N-Tetracosanol-1	10.53	JAC74C, JAC81, JAC45	SFM, Synthetic, YMS
1-Methylbutyl Acetoacetate	10.58	JAC60, JAC25	Synthetic, SFM, YMS
3,4-Epoxy Menthane	10.77	JAC60, JAC74C, JAC45	Synthetic, SFM, YMS
Heptane, 2,3-Dimethyl-	10.85	JAC60, JAC25	Synthetic, SFM, YMS
Pentadecane, 2,6,10,14-Tetramethyl-	10.85	JAC74C, JAC95	SFM, Synthetic, YMS
**Cyclopentadecane***	10.89	JAC74C, JAC81	SFM, Synthetic, YMS
**Dodecane***	11	JAC60, JAC25, JAC95, JAC74C	Synthetic, SFM, YMS
**Pentadecane***	11	JAC74C, JAC45, JAC60, JAC95	SFM, Synthetic, YMS
**Oleic Acid***	11.05	JAC74C, JAC25, JAC95, JAC81, JAC45	SFM, Synthetic, YMS
Acetophenone	11.09	JAC74C, JAC81, JAC45, JAC25	SFM, Synthetic, YMS
Benzamide, *N*-(2'-Ethylphenyl)-	11.1	JAC95, JAC60	YMS, Synthetic, SFM

Decylamine, *N*-Allyl-	11.25	JAC60, JAC74C, JAC25, JAC81	Synthetic, SFM, YMS
**1-Tetradecanol***	11.29	JAC60, JAC74C, JAC25, JAC45, JAC95	Synthetic, SFM, YMS
Methyl P-Aminosalicylate	11.38	JAC60, JAC81	Synthetic, SFM, YMS
12-Methyl-1-Tetradecanol	11.42	JAC60, JAC25, JAC81	Synthetic, SFM, YMS
**7-Tetradecene***	11.61	JAC60, JAC74C, JAC95, JAC25	Synthetic, SFM, YMS
18-Nonadecenoic Acid	11.72	JAC60, JAC95	Synthetic, SFM, YMS
**Dodecyl Formate***	11.72	JAC74C, JAC25, JAC81, JAC45	SFM, Synthetic, YMS
Octane, 2,3-Dichloro-	11.86	JAC60, JAC95, JAC25, JAC81, JAC45	Synthetic, SFM, YMS
S-Benzoyl-*N*-(O-Hydroxybenzylidene)Thiohydroxylamine	11.89	JAC60, JAC81	Synthetic, SFM, YMS
Methyl Benzoate	11.89	JAC74C, JAC45, JAC25	SFM, Synthetic, YMS
Hexahydro-2H-Pyrido(1,2-A)Pyrazin-3(4H)-One	11.96	JAC60, JAC74C, JAC95, JAC25, JAC81, JAC45	Synthetic, SFM, YMS
**Undecane***	12.01	JAC81	YMS, Synthetic, SFM
L-Proline, *N*-(Cyclopentylcarbonyl)-, Undecyl Ester	12.01	JAC81, JAC45	YMS, Synthetic, SFM
Linalool	12.05	JAC74C, JAC95, JAC81, JAC45	SFM, Synthetic, YMS
Camphene_Adms	12.05	JAC81, JAC74C	YMS, Synthetic, SFM
Dodecyl Octyl Ether	12.09	JAC25, JAC45, JAC95, JAC74C	SFM, YMS, Synthetic
2,3-Dihydroxy-3,7,11,15-Tetramethylhexadecan-1-ol Nitrate	12.09	JAC60, JAC74C	Synthetic, SFM, YMS
3,5,5-Trimethylhexanol	12.19	JAC45, JAC95	YMS, Synthetic, SFM
**1,1,4-Trimethylcyclohexane***	12.19	JAC60, JAC81, JAC95	Synthetic, SFM, YMS
2-Propenoic Acid, 2-Methyl-, 1,2-Ethanediyl Ester	12.35	JAC60, JAC25	Synthetic, SFM, YMS
Didodecylphosphine Oxide	12.41	JAC74C, JAC25, JAC81	SFM, Synthetic, YMS
Cyclopentanecarboxamide, *N*-(Cyclopentylcarbonyl)-*N*-Isobutyl-	12.42	JAC45	Synthetic, SFM
**Phenyl Ethyl Alcohol***	12.42	JAC45, JAC25, JAC95	YMS, Synthetic, SFM
Heptane, 1,1’-Oxybis-	12.44	JAC60, JAC74C, JAC25, JAC95	Synthetic, SFM, YMS

**2-Tert-Butylcyclohexanone***	12.85	JAC60, JAC74C, JAC95, JAC81, JAC45	Synthetic, SFM, YMS
Propanoic Acid, 2-Methyl-, 2-Methylpentyl Ester	12.93	JAC60, JAC95, JAC81	Synthetic, SFM, YMS
Tartaric Acid-Tetra-Tms	13	JAC25, JAC60	SFM, YMS, Synthetic
2-Heptylfuran	13	JAC95	YMS, Synthetic, SFM
Cyclopentasiloxane, Decamethyl-	13.08	JAC60, JAC95, JAC81	Synthetic, SFM, YMS
2-Heptenoic Acid	13.08	JAC74C, JAC25, JAC45	SFM, Synthetic
Cyclodecanone	13.22	JAC81, JAC95, JAC25, JAC74C, JAC60, JAC45	Synthetic, SFM, YMS
Tricosane	13.28	JAC81	YMS, Synthetic, SFM
2,3-Dimethyl-3-Heptene, (*Z*)-	13.41	JAC25, JAC45	SFM, YMS, Synthetic
1-Methoxy-2-Methylbenzene	13.48	JAC45	YMS, Synthetic, SFM
**2,4,4-Trimethyl-1-Hexene***	13.57	JAC60, JAC45	Synthetic, SFM, YMS
**Docosyl Pentyl Ether***	13.69	JAC74C, JAC25	SFM, Synthetic, YMS
**Cyclohexanol, 5-Methyl-2-(1-Methylethyl)-***	14.07	JAC60, JAC74C, JAC25, JAC95, JAC81, JAC45	Synthetic, SFM, YMS
Cyclohexane, 2-Chloro-4-Methyl-1-(1-Methylethyl)-, [1S-(1.Alpha.,2.Beta.,4.Beta.)]-	14.16	JAC60, JAC95, JAC45	Synthetic, SFM, YMS
**2-Methylisoborneol***	14.35	JAC60	Synthetic, SFM, YMS
Bornyl Pentanoate	14.44	JAC81	Synthetic, SFM
N*-Octadecane	14.56	JAC60, JAC81	Synthetic, SFM, YMS
(*Z*)-2-Tridecen-1-ol	14.65	JAC60	Synthetic, SFM, YMS
Tridecanal	14.65	JAC81	YMS, Synthetic, SFM
P-Benzyloxybenzaldehyde Semicarbazone	14.79	JAC25, JAC74C	Synthetic, YMS
**1-Chloroeicosane***	14.83	JAC25, JAC45, JAC74C	SFM, YMS, Synthetic
**Ethanone, 2-Chloro-1-(2,4-Dimethylphenyl)-***	14.88	JAC95, JAC81	Synthetic, SFM
P-Methoxybenzylazidoformate	14.95	JAC60, JAC45, JAC74C	Synthetic, SFM, YMS
**2-(1-Cyclohexenyl)Cyclohexanone***	15.23	JAC74C, JAC81, JAC95	SFM, Synthetic, YMS

Carbonic Acid, Monoamide, *N*-Isobutyl-, 2-Methoxyethyl Ester	15.38	JAC25, JAC81	SFM, Synthetic
Cyclohexanecarboxylic Acid, 1-Phenyl-	15.49	JAC25, JAC74C	SFM, YMS, Synthetic
Calamenene (Cis-)	15.49	JAC74C, JAC95, JAC45, JAC25	SFM, Synthetic, YMS
(2-Hydroxy-4,5-Dimethylbenzoyl)Formic Acid	15.52	JAC74C	SFM, Synthetic
Phthalic Acid Dipropyl Ester	15.58	JAC25, JAC95	SFM, YMS
Bis(3-Methoxybenzyl) Ether	15.58	JAC45	YMS, Synthetic, SFM
**(7S)-(-)-10,10-Di-Me-5-Thia-4-Azatricyclo[5.2.1.0-3,7]Dec-3-Ene-5,5-Dioxide***	15.71	JAC81, JAC95	Synthetic, SFM
**Ethyl Acetophenone (P-)***	15.75	JAC74C, JAC81, JAC95, JAC45, JAC25	SFM, Synthetic, YMS
3,4-Dimethylbenzophenone	15.84	JAC74C, JAC45	SFM, Synthetic, YMS
Gamma-Linolenic Acid	15.84	JAC81	Synthetic, SFM
1-Hexanol, 5-Methyl-2-(1-Methylethyl)-	15.93	JAC60	SFM, YMS
**Pentadecafluorooctanoic Acid, 2-Ethylhexyl Ester***	15.93	JAC95, JAC81	YMS, Synthetic, SFM
**(*Z,Z,Z*)-6,9,15-Octadecatrienoic Acid Methyl Ester***	16.02	JAC60, JAC74C, JAC25, JAC81	Synthetic, YMS
**2’-Ethylpropiophenone***	16.15	JAC25, JAC74C, JAC81, JAC95, JAC45	SFM, YMS, Synthetic
Benzoic Acid, Heptyl Ester	16.19	JAC81	YMS, Synthetic, SFM
4-*N*-Dodecylresorcinol	16.19	JAC81, JAC60	Synthetic, SFM
4-Tert-Butylphenyl Acetate	16.26	JAC81, JAC95	Synthetic, SFM
**Tridecanol***	16.3	JAC81	YMS, Synthetic, SFM
3,4-Dimethylbenzoic Acid Trimethylsilyl Ester	16.36	JAC25	SFM, Synthetic
4-Formyl-2-Methoxytropone	16.36	JAC25	YMS, SFM
N,N’-(4-Methyl-M-Phenylene)Bisacetamide	16.64	JAC95, JAC60	YMS, Synthetic, SFM
Decyl Heptyl Ether	16.81	JAC25, JAC74C, JAC95	SFM, YMS, Synthetic

Hexane, 3,3-Dimethyl-	17.11	JAC25, JAC74C	SFM, Synthetic
Phenethyl Acetate	17.37	JAC74C	SFM, Synthetic
4’-Ethylpropiophenone	17.4	JAC25, JAC74C	SFM, Synthetic
**Cubebene (Alpha-)***	17.77	JAC95, JAC81	YMS, Synthetic, SFM
**Caryophyllene (*Z*-)***	17.93	JAC95, JAC81, JAC25	Synthetic, SFM
Guaiadiene (6,9-)	18.05	JAC60, JAC74C, JAC81, JAC95, JAC45, JAC25	Synthetic, SFM, YMS
Gamma-Muurolene	18.05	JAC74C, JAC25, JAC95, JAC81	SFM, Synthetic, YMS
Ibuprofen	18.23	JAC25, JAC74C, JAC60, JAC45	SFM, YMS, Synthetic
(1*S*,2*E*,4*E*,7*E*,11*E*)-10-Oxocembra-2,4,7,11-Tetraene	18.23	JAC95	YMS, Synthetic, SFM
Longifolene	18.3	JAC74C, JAC60, JAC45, JAC95	SFM, Synthetic
5,9,13,17-Tetramethyl 4,8,12,16-Octadecatetraenoic Acid	18.5	JAC25	Synthetic, SFM
5-Acetylsalicylamide	18.5	JAC25, JAC60, JAC95	SFM, YMS, Synthetic
2,6,10,15,19,23-Pentamethyl-2,6,18,22-Tetracosatetraen-10,15-Diol	18.51	JAC74C, JAC45	SFM, Synthetic, YMS
Beta-Caryophyllene	18.51	JAC81	YMS, Synthetic, SFM
1-Ethyl-Trans-2-Butenyl 2,4,6-Trimethylbenzoate, (.+/-.)-	18.76	JAC25, JAC74C, JAC60, JAC81	SFM, YMS, Synthetic
Eicosyl Isobutyl Ether	18.86	JAC60, JAC81	Synthetic, SFM, YMS
2,6-Di-Tert-Butyl-1,4-Benzoquinone	18.93	JAC74C, JAC45, JAC60, JAC45	SFM, Synthetic, YMS
2,5-Dimethoxy-Alpha-Methyl-Benzeneethanamine	18.99	JAC95	YMS, Synthetic,
**Carbonic Acid, 2,2,2-Trichloroethyl 2-Ethylhexyl Ester***	19.06	JAC81, JAC95	YMS, Synthetic, SFM
Cyclodecane	19.13	JAC25, JAC74C, JAC60, JAC95, JAC45	SFM, YMS, Synthetic
**Eicosane***	19.26	JAC60, JAC25	Synthetic, SFM, YMS
Bis(2-Ethylhexyl)Hydrogen Phosphate	19.63	JAC81	YMS, Synthetic, SFM
Liguloxide	19.7	JAC81, JAC95	YMS, Synthetic, SFM
**Octadecane***	19.85	JAC25, JAC60, JAC81	SFM, YMS, Synthetic
**Nonadecane***	19.86	JAC74C, JAC81	SFM, Synthetic, YMS

**Malonic Acid, Di(4-Heptyl) Ester***	20.09	JAC25, JAC60, JAC95, JAC45	SFM, YMS, Synthetic
**Heptylcyclohexane***	20.14	JAC81	YMS, Synthetic, SFM
3,7-Dimethyl-6-Octenyl 3-Methylbutyrate	20.3	JAC45	Synthetic, SFM
Diethyl Phthalate	20.54	JAC25, JAC74C, JAC81, JAC45	SFM, YMS, Synthetic
2H-Pyran-2-One, Tetrahydro-6-Nonyl-	21.06	JAC81, JAC25	YMS, Synthetic, SFM
Benzophenone	21.12	JAC74C, JAC60, JAC45	SFM, Synthetic, YMS
Androstanedione	21.27	JAC74C, JAC95	SFM, Synthetic, YMS
Cyclopentanecarboxylic Acid, 1-(4-Methylphenyl)-	21.31	JAC45, JAC74C, JAC60, JAC25, JAC95, JAC81	YMS, Synthetic, SFM
(1S,3S,4R)-(+)-Menthyl (*R*)-P-Toluenesulfinate	21.58	JAC45	YMS, Synthetic, SFM
1-Pentadecene	21.59	JAC25, JAC60, JAC45	SFM, YMS, Synthetic
Triacontane, 1-Iodo-	21.85	JAC25, JAC74C, JAC95, JAC60, JAC81	SFM, YMS, Synthetic
Benzoic Acid, 2-Ethylhexyl Ester	21.97	JAC60, JAC95	Synthetic, SFM, YMS
**Methyl Tetradecanoate***	22.09	JAC60, JAC81, JAC95	Synthetic, SFM, YMS
Tris(Trimethylsiloxy)Ethylene	22.23	JAC25, JAC74C, JAC45, JAC95	SFM, YMS
7-Methoxy-2,5-Dimethyl-1,2,3,4-Tetrahydropyrimido(1,6-A)Indole	22.41	JAC60, JAC81, JAC45	Synthetic, SFM, YMS
**Hexanoic Acid, 2-Hexenyl Ester, (*E*)-***	22.65	JAC81	YMS, Synthetic, SFM
Benzene, 1-Methoxy-3-Undecyl-	22.67	JAC45	Synthetic, SFM
Octadecanol	22.87	JAC25, JAC95, JAC81	SFM, YMS, Synthetic
**13-Methyltetradecanoic Acid Methyl Ester***	23.17	JAC95, JAC60	YMS, Synthetic, SFM
Diisobutyl Phthalate	23.57	JAC60, JAC95, JAC81	Synthetic, SFM, YMS
2-Pentadecanone	23.98	JAC25	YMS
**Methyl Palmitate***	24.21	JAC60, JAC25, JAC95, JAC81	Synthetic, SFM, YMS
15-Methylhexadecanoic Acid Methyl Ester	24.31	JAC60, JAC25	Synthetic, SFM, YMS
Fumaric Acid, 2,4,4-Trimethylpentyl 3-Methylbut-2-En-1-Yl Ester	24.69	JAC45, JAC25	YMS, Synthetic, SFM

**Disulfide, Di-Tert-Dodecyl***	25.01	JAC74C, JAC45	SFM, Synthetic, YMS
L-Arginine	25.1	JAC25	Synthetic, YMS,
N-(O-Chlorophenyl)-4,5-Dihydro-3-Furamide	25.44	JAC60	Synthetic, SFM
Pyridoxamine	25.5	JAC25, JAC60	SFM, YMS, Synthetic
2,4,6-Tris(Trimethylsiloxy)Benzoic Acid Trimethylsilyl Ester	25.99	JAC60, JAC74C	Synthetic, SFM, YMS
1,2-Benzenedicarboxylic Acid 2-Ethylhexylmethyl Ester	26.14	JAC60, JAC81, JAC95	Synthetic, SFM, YMS
**1-Bromo-8-Tetrahydropyranyloxyoctane***	26.21	JAC25, JAC81, 74C	YMS, SFM,
Benzene-1,2,4,5-Tetracarboxylic Acid Tetra(Trimethylsilyl) Ester	26.34	JAC74C, JAC45	SFM, Synthetic, YMS
**2-(3-Benzoylphenyl)Propionic Acid Trimethylsilyl Ester***	26.39	JAC60, JAC81	Synthetic, SFM, YMS
Octahydro-2H-Quinolizine	26.43	JAC60	SFM, YMS
**Undecane***	26.49	JAC25, JAC45, JAC60, JAC74C	SFM, YMS, Synthetic
**Tetracosane***	27.23	JAC25, JAC74C, JAC60, JAC81, JAC95	YMS, SFM
3-Methyladipic Acid Di-Tms	27.44	JAC25	YMS, SFM
**Heptacosane***	27.53	JAC81, JAC25	YMS, Synthetic, SFM
2,3,4-Tris(Trimethylsiloxy)Benzoic Acid Trimethylsilyl Ester	28.06	JAC25, JAC74C, JAC60, JAC81	SFM, YMS, Synthetic
**Behenic Amide***	28.27	JAC81	YMS, SFM
Hexacosane, 1-Iodo-	28.49	JAC74C, JAC25, JAC95, JAC81	SFM, Synthetic, YMS
Butyl Hexacosyl Ether	29.06	JAC45	YMS, SFM
Diamyl Phthalate	29.29	JAC74C, JAC81	SFM, Synthetic, YMS
1,2-Benzenedicarboxylic Acid, Bis(2-Methylpropyl) Ester	29.31	JAC45	YMS, Synthetic
Butoxycarbonylmethyl Butyl Phthalate	29.35	JAC25	YMS, SFM,
3-Ethyl-2,6,10-Trimethylundecane	29.57	JAC25	YMS, Synthetic,
Hexanamide, 6-Bromo-*N*-Hept-2-Yl-	29.94	JAC74C, JAC95, JAC60, JAC45	SFM, Synthetic, YMS

**1,2-Benzenedicarboxylic Acid Bis(2-Ethylhexyl) Ester***	30.59	JAC25, JAC45, JAC74C, JAC60, JAC95, JAC81	SFM, YMS, Synthetic
Dodecyl Nonyl Ether	31.11	JAC25	YMS, SFM,
N-(Aminocarbonyl)-2-Bromo-2-Ethyl-Butanamide Carbromal	31.17	JAC74C, JAC25	Synthetic, YMS
**3-Isopropyl-6,10-Dimethylundecane-2-ol***	31.78	JAC25	SFM, YMS, Synthetic

Table S8. Bacterially derived compounds annotated in replicate VOC analysis of the six selceted *Streptomyces* isolates using the conventional method. VOCs annotated during both the pooled culture screen and the replicated analysis are shown in bold and labelled with an asterisk*.

Compound/VOC	Retention time (min)	Annotated in (isolates)	Media
2-Furanol, tetrahydro-2-methyl-	4.02	JAC81, JAC95	synthetic
Pyrimidine-2,4(1H,3H)-dione, 5-amino-6-nitroso-	4.021	JAC25	SFM
Tetrahydrofuran, 2-propyl-	4.024	JAC74C	synthetic
Benzene, (propoxymethyl)-	4.231	JAC45	SFM
Diacetyl sulphide	4.313	JAC45	YMS
**Butanoic acid, 2-methyl-, methyl ester***	4.314	JAC45, JAC60	SFM, synthetic
Cyclobutanone, 2-methyl-	4.562	JAC45	SFM
Acetamide, N,N'-ethylenebis(*N*-nitro-	4.579	JAC81	YMS
**2-Methyl-2,3-pentanediol***	4.72	JAC60, JAC25	YMS, SFM
1,8-Nonanediol, 8-methyl-	4.722	JAC25, JAC60	YMS
**2-Hydroxy-3-pentanone***	4.854	JAC95, JAC60	YMS
4-Methyl-2,4-bis(4'-trimethylsilyloxyphenyl)pentene-1	4.862	JAC60	synthetic
Hexane, 3,4-bis(1,1-dimethylethyl)-2,2,5,5-tetramethyl-	4.864	JAC95	SFM
Sulfurous acid, cyclohexylmethyl heptadecyl ester	4.923	JAC25	YMS
Propanoic acid, 2-methyl-, anhydride	5.111	JAC95	YMS
1,6:3,4-Dianhydro-2-deoxy-.beta.-d-ribo-hexopyranose	5.499	JAC25	YMS
Butanoic acid, 3-methyl-	5.582	JAC95	synthetic
Butanoic acid, 3-methyl-	5.634	JAC95	synthetic
**Butane, 2-azido-2,3,3-trimethyl-***	5.683	JAC95	synthetic

Butanoic acid, 3-methyl-	6.112	JAC74C	YMS
**1-Hexanol***	6.197	JAC60	synthetic
**(*S*)-3,4-Dimethylpentanol***	6.257	JAC95	YMS
**Butanedioic acid, phenyl-***	6.73	JAC60	synthetic
Hexanoic acid, 5-methyl-, methyl ester	9.322	JAC60	SFM
**Benzyl alcohol***	10.269	JAC95	SFM
Pyrazine, 2-methyl-5-(1-methylethyl)-	10.541	JAC95	YMS
Benzoic acid, methyl ester	11.889	JAC95	SFM
**Phenylethyl Alcohol***	12.393	JAC95, JAC60	SFM, YMS
**Bicyclo[3.2.0]hepta-2,6-diene***	12.398	JAC95	YMS
4-Acetylbenzoic acid	14.294	JAC95	YMS
**2-Methylisoborneol***	14.348	JAC60, JAC25	SFM, synthetic, YMS
1H-Indene, 1-ethylideneoctahydro-7a-methyl-, cis-	15.234	JAC60, JAC25, JAC95, JAC81, JAC74C	SFM, synthetic, YMS
1H-Indene, 1-ethylideneoctahydro-7a-methyl-, (1*Z*,3aα,7aβ)-	15.316	JAC25, JAC95, JAC81	SFM, synthetic
1H-Indene, 1-ethylideneoctahydro-7a-methyl-, (1E,3aα,7aβ)-	15.626	JAC81	SFM
**(1,2,2-trimethyl-3-cyclopenten-1-yl)acetaldehyde***	15.711	JAC25, JAC95, JAC81	SFM
**Phenol, 4-[2-(methylamino)ethyl]-***	15.712	JAC74C	YMS
**2-Hydroxy-5-ethyl-5-methylcyclopent-2-en-1-one***	15.94	JAC81	SFM
Decane, 3-bromo-	16.709	JAC74C	SFM
2,6-Octadienoic acid, 3,7-dimethyl-, methyl ester	16.772	JAC74C	YMS
1,5-Cyclodecadiene, 1,5-dimethyl-8-(1-methylethenyl)-, [*S*-(*Z*,*E*)]-	17.933	JAC95, JAC74C	SFM, YMS

**Geosmin***	18.331	JAC60,JAC25, JAC95, JAC81, JAC74C	SFM, synthetic, YMS
**1-Methylene-2b-hydroxymethyl-3,3-dimethyl-4b-(3-methylbut-2-enyl)-cyclohexane***	18.421	JAC95, JAC81	SFM, synthetic, YMS
1H-3a,7-Methanoazulene, octahydro-3,8,8-trimethyl-6-methylene-, [3*R*-(3.alpha.,3a.beta.,7.beta.,8a.alpha.)]-	18.506	JAC81	SFM
4,4-Dimethyl-3-(3-methylbut-3-enylidene)-2-methylenebicyclo[4.1.0]heptane	18.578	JAC81	Synthetic, SFM
2-Methyl-3-(3-methyl-but-2-enyl)-2-(4-methyl-pent-3-enyl)-oxetane	18.724	JAC81	SFM
**3-Heptyne, 5-methyl-***	18.729	JAC95	SFM
2H-Pyran-2-one, 6-hexyltetrahydro-	18.799	JAC95	SFM
5,6-Decadien-3-yne, 5,7-diethyl-	18.811	JAC81	Synthetic, YMS
Cadina-1(10),6,8-triene	18.812	JAC81	SFM
2*R*-Acetoxymethyl-1,3,3-trimethyl-4t-(3-methyl-2-buten-1-yl)-1t-cyclohexanol	18.918	JAC81	SFM
**3-Isopropoxy-1,1,1,7,7,7-hexamethyl-3,5,5-tris(trimethylsiloxy)tetrasiloxane***	18.965	JAC81	SFM
2,3,4-Trifluorobenzoic acid, 2,6-dimethylnon-1-en-3-yn-5-yl ester	18.967	JAC81	synthetic
Cyclobutaneacetonitrile, 1-methyl-2-(1-methylethylidene)-	19.031	JAC74C	YMS
**1,6-Cyclodecadiene, 1-methyl-5-methylene-8-(1-methylethyl)-, [*S*-(*E*,*E*)]-***	19.273	JAC25, JAC95, JAC81, JAC74C	SFM, synthetic, YMS
Acetic acid, (dodecahydro-7-hydroxy-1,4b,8,8-tetramethyl-10-oxo-2(1H)-phenanthrenylidene)-,2-(dimethylamino)ethyl ester	19.482	JAC81	SFM
2H-3,9a-Methano-1-benzoxepin, octahydro-2,2,5a,9-tetramethyl-, [3*R*-(3.alpha.,5a.alpha.,9.alpha.,9a.alpha.)]-	19.699	JAC81	SFM
photocitral B	19.699	JAC81	synthetic
Cyclohexanol, 4-ethyl-4-methyl-3-(1-methylethyl)-, (1.alpha.,3.beta.,4.alpha.)-	19.704	JAC95	SFM
**1-Hexyl-2-nitrocyclohexane***	19.803	JAC95	SFM
Hexacosane	19.854	JAC95	YMS
Cyclobutaneacetonitrile, 1-methyl-2-(1-methylethylidene)-	19.855	JAC95	SFM

1,5-Cyclodecadiene, 1,5-dimethyl-8-(1-methylethenyl)-, [*S*-(*Z*,*E*)]-	20.109	JAC74C	YMS
Hexane, 2,3-dimethyl-	20.282	JAC95	SFM
5-Hepten-3-one, 5-ethyl-2-methyl-	20.451	JAC81	SFM
Caryophyllenyl alcohol	20.523	JAC95	SFM
Diethyl Phthalate	20.523	JAC25, JAC81, JAC74C	YMS
Ethaneperoxoic acid, 1-cyano-1-[2-(2-phenyl-1,3-dioxolan-2-yl)ethyl]pentyl ester	20.523	JAC74C	YMS
6,7-Dodecanedione	21.056	JAC81	SFM
2(5H)-Furanone, 5-(bromomethyl)-5-phenyl-	21.06	JAC81	synthetic
**5,6-Decadien-3-yne, 5,7-diethyl-***	21.126	JAC25	synthetic
**Naphthalene, 1,2,3,4,4a,7-hexahydro-1,6-dimethyl-4-(1-methylethyl)-***	21.128	JAC25	SFM, YMS
cis-muurola-3,5-diene	21.129	JAC25	SFM
.alfa.-Copaene	21.13	JAC95	SFM
**10-Methylundecan-4-olide***	21.155	JAC74C	Synthetic, YMS
1-Phenyl-2-methyl-oct-1-ene	21.177	JAC81	SFM, synthetic, YMS
**Dihydro-cis-.alpha.-copaene-8-ol***	21.263	JAC81, JAC25, JAC95	SFM, synthetic
Naphthalene, 1,2,3,4,4a,5,6,8a-octahydro-4a,8-dimethyl-2-(1-methylethenyl)-, [2*R*-(2.alpha.,4a.alpha.,8a.beta.)]-	21.268	JAC95	SFM
**Phenylacetaldehyde *N*-methyl-*N*-formylhydrazone***	21.391	JAC95	YMS
**7-epi-.alpha.-eudesmol***	21.547	JAC95	SFM
1-Methyl-4-isopropyl-cyclohexyl 2-hydroperfluorobutanoate	21.836	JAC45	YMS
Cyclohexanol, 1,3,3-trimethyl-2-(3-methyl-2-methylene-3-butenylidene)-, (*Z*)-	21.836	JAC60	YMS
2,5-Octadiyne, 4,4-diethyl-	21.914	JAC81	SFM, synthetic
**Cyclohexanol, 1-ethyl-2,2-dimethyl-6-methylene-***	22.103	JAC81	SFM
Heptacosane	25.901	JAC25	YMS

Hexadecanoic acid, 15-methyl-, methyl ester	26.128	JAC25	YMS
1-Hexyl-2-nitrocyclohexane	27.638	JAC25	YMS
**Hexanedioic acid, bis(2-ethylhexyl) ester***	28.636	JAC25	YMS

Table S9. Web links for MSHub/GNPS jobs generated in this study.

Job	Web link
MSHub for pooled cluture VOCs analysis of the 37 *Streptomyces* isolates	https://gnps.ucsd.edu/ProteoSAFe/status.jsp?task=8aa14b90160d482aa19de0cd20558038
GNPS library search for the 37 *Streptomyces* isolates (annotatio cosine score 0.5)	https://gnps.ucsd.edu/ProteoSAFe/status.jsp?task=2fccfed64f1047009283bb6904104202
GNPS library search for the 37 *Streptomyces* isolates (annotatio cosine score 0.65)	https://gnps.ucsd.edu/ProteoSAFe/status.jsp?task=b47661ec7ae84c1bae3c90b6e9c56764
MSHub for replicate VOCs analysis of JAC25	https://gnps.ucsd.edu/ProteoSAFe/status.jsp?task=9fefb239a3514a638afefaf3a88aa6ba
GNPS library search for JAC25	https://gnps.ucsd.edu/ProteoSAFe/status.jsp?task=3b476735d4684a3b9e09f7c95f8416eb
MSHub for replicate VOCs analysis of JAC45	https://gnps.ucsd.edu/ProteoSAFe/status.jsp?task=659ab3212752409fa093221e05203ff4
GNPS library search for JAC45	https://gnps.ucsd.edu/ProteoSAFe/status.jsp?task=e0a436259d6d430090d59af66976c134
MSHub for replicate VOCs analysis of JAC60	https://gnps.ucsd.edu/ProteoSAFe/status.jsp?task=c8f70167c87e4384968c47cccbd386b9
GNPS library search for JAC60	https://gnps.ucsd.edu/ProteoSAFe/status.jsp?task=8f88689b27d74a74b29dd3c44408b728
MSHub for replicate VOCs analysis of JAC74C	https://gnps.ucsd.edu/ProteoSAFe/status.jsp?task=595aff5b193a439583b8b14f48eb3bd0
GNPS library search for JAC74C	https://gnps.ucsd.edu/ProteoSAFe/status.jsp?task=17f939cdb9934ec1934e2a6d91e4e277
MSHub for replicate VOCs analysis of JAC81	https://gnps.ucsd.edu/ProteoSAFe/status.jsp?task=ca387e87543a4b72aa642069db5868cb

GNPS library search for JAC81	https://gnps.ucsd.edu/ProteoSAFe/status.jsp?task=81992bd8f98d4dfcb5153d5a7f4bdf00
MSHub for replicate VOCs analysis of JAC95	https://gnps.ucsd.edu/ProteoSAFe/status.jsp?task=0c731d7939c345dea0fcd88fe52c9b92
GNPS library search for JAC95	https://gnps.ucsd.edu/ProteoSAFe/status.jsp?task=15f34383681e4cb89093dc54a0cd224a

REFERENCES

1. Shi W, Li M, Wei G, Tian R, Li C, Wang B, Lin R, Shi C, Chi X, Zhou B, Gao Z. 2019. The occurrence of potato common scab correlates with the community composition and function of the geocaulosphere soil microbiome. Microbiome 7:14.

2. Nagashima K, Hisada T, Sato M, Mochizuki J. 2003. Application of new primer-enzyme combinations to terminal restriction fragment length polymorphism profiling of bacterial populations in human feces. Appl Environ Microbiol 69:1251–1262.

3. Guo Y, Zheng W, Rong X, Huang Y. 2008. A multilocus phylogeny of the *Streptomyces griseus* 16S rRNA gene clade: Use of multilocus sequence analysis for streptomycete systematics. Int J Syst Evol Microbiol 58:149–159.
